# Supplementary figures and images for: RGS10 deficiency facilitates distant metastasis by inducing epithelial–mesenchymal transition in breast cancer (part 1 of 2)
Source: eLife. 2024 Aug 15;13:RP97327. doi: 10.7554/eLife.97327 (PMC11326775; doi:10.7554/eLife.97327)

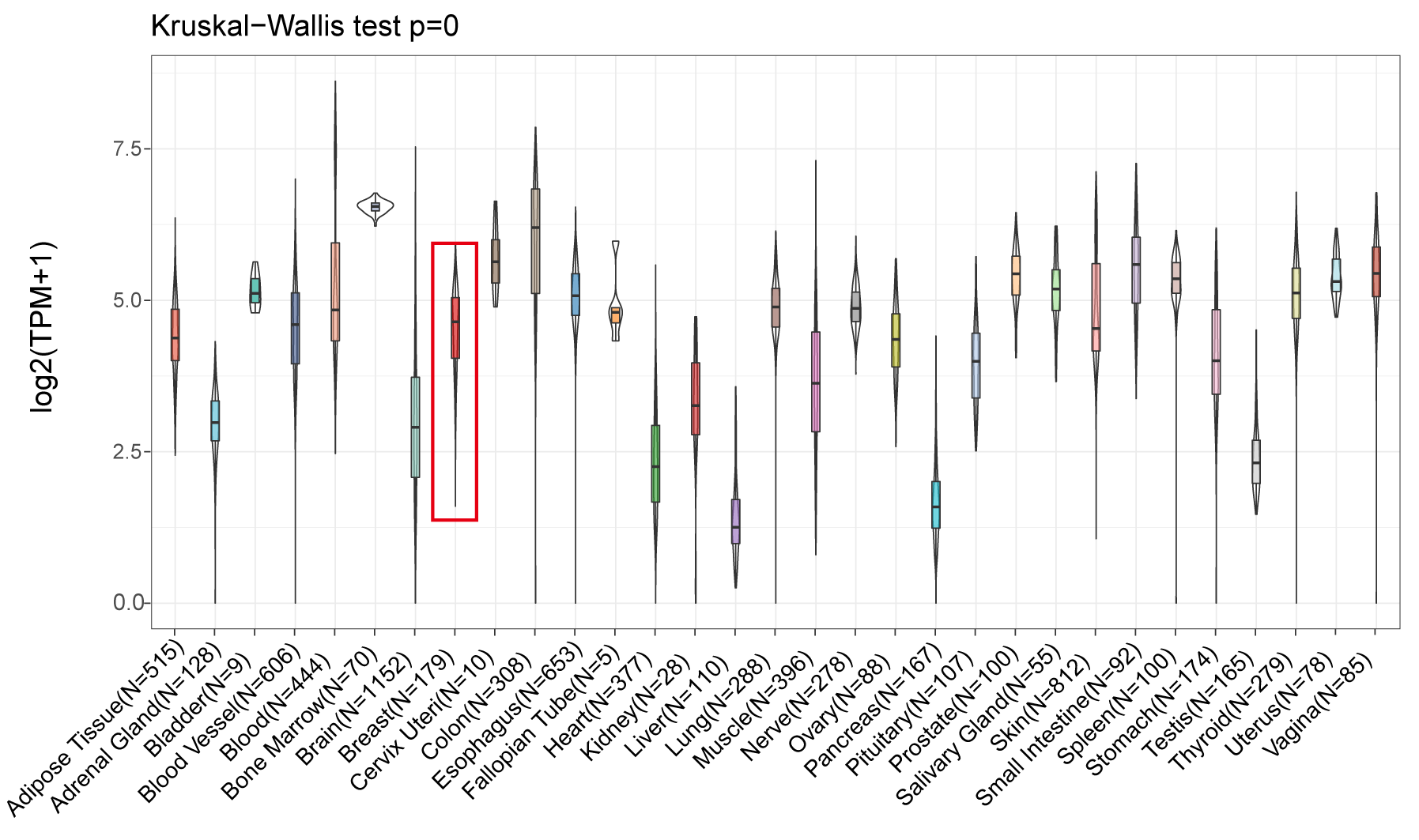

Supplement: Figure 1—source data 1. [file elife-97327-fig1-data1.zip › Figure 1-Source data 1/F1A.tif]

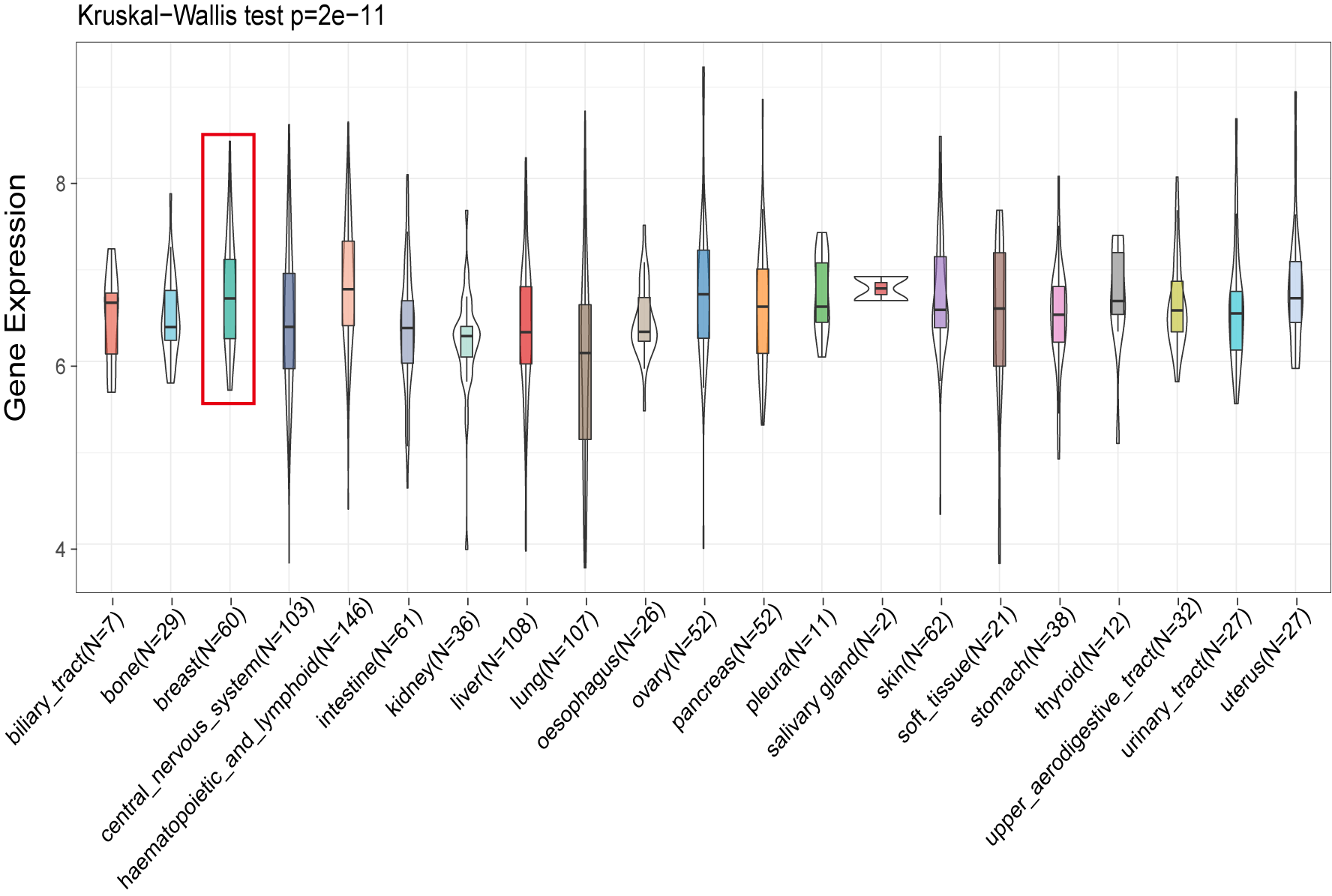

Supplement: Figure 1—source data 1. [file elife-97327-fig1-data1.zip › Figure 1-Source data 1/F1B.tif]

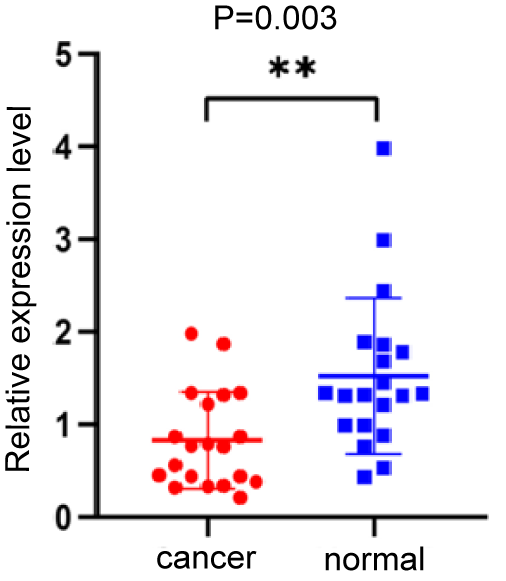

Supplement: Figure 1—source data 1. [file elife-97327-fig1-data1.zip › Figure 1-Source data 1/F1C.tif]

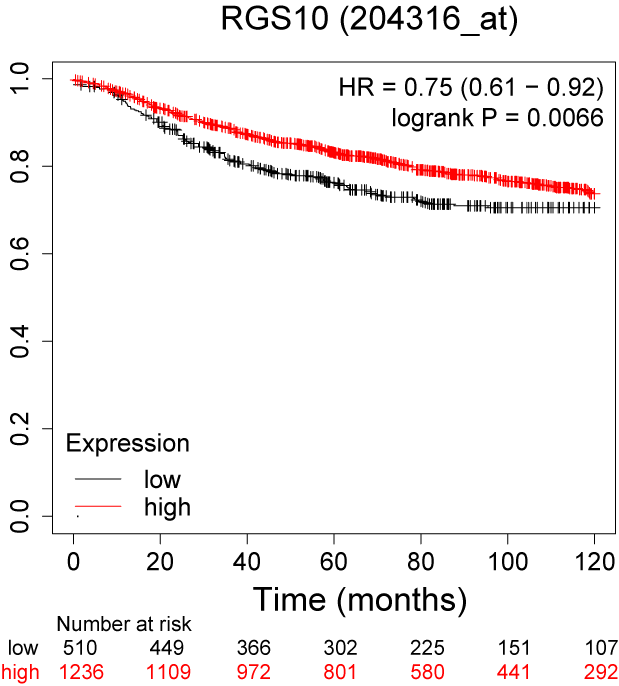

Supplement: Figure 1—source data 1. [file elife-97327-fig1-data1.zip › Figure 1-Source data 1/F1D.tif]

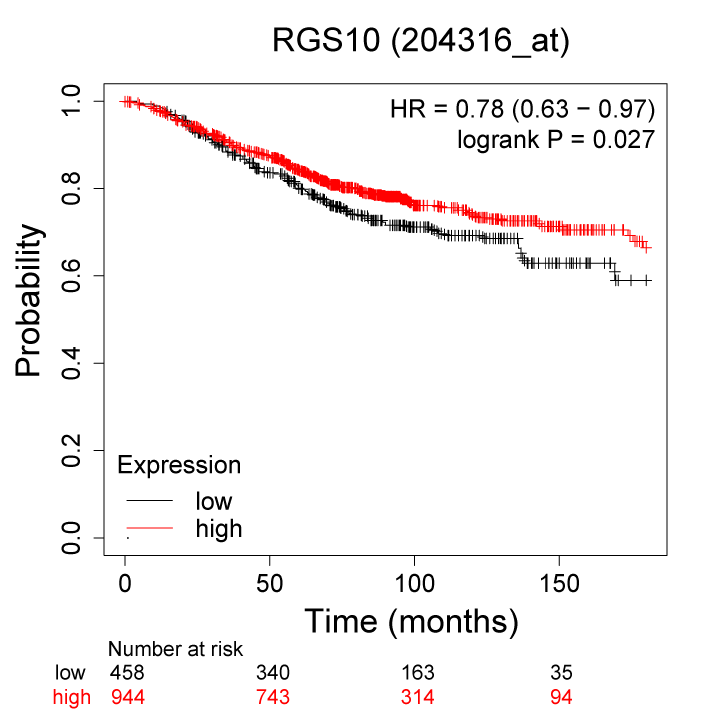

Supplement: Figure 1—source data 1. [file elife-97327-fig1-data1.zip › Figure 1-Source data 1/F1E.tif]

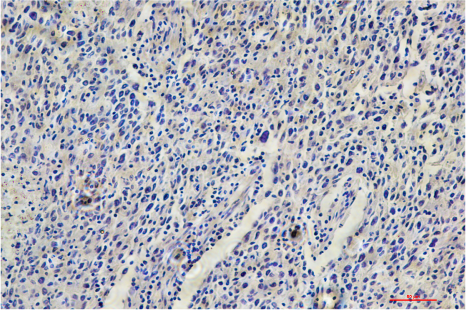

Supplement: Figure 1—source data 1. [file elife-97327-fig1-data1.zip › Figure 1-Source data 1/F1F-1.tif]

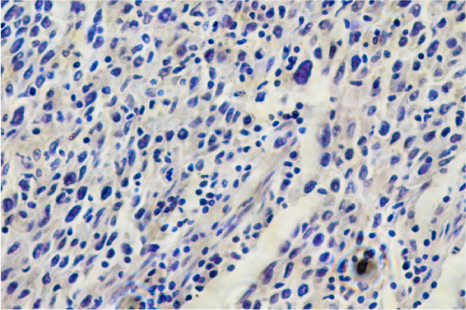

Supplement: Figure 1—source data 1. [file elife-97327-fig1-data1.zip › Figure 1-Source data 1/F1F-2.tif]

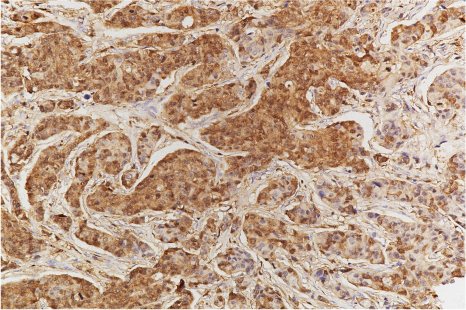

Supplement: Figure 1—source data 1. [file elife-97327-fig1-data1.zip › Figure 1-Source data 1/F1F-3.tif]

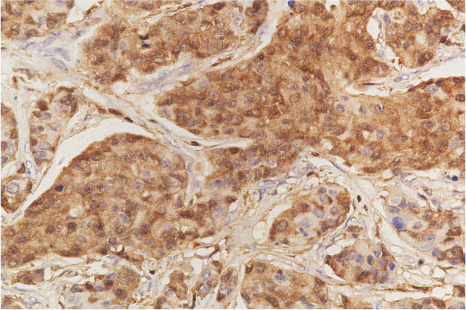

Supplement: Figure 1—source data 1. [file elife-97327-fig1-data1.zip › Figure 1-Source data 1/F1F-4.tif]

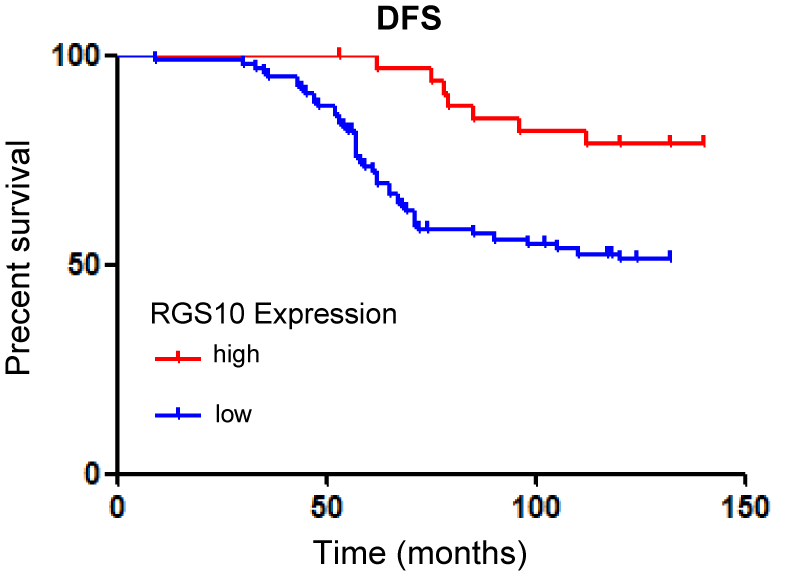

Supplement: Figure 1—source data 1. [file elife-97327-fig1-data1.zip › Figure 1-Source data 1/F1G.tif]

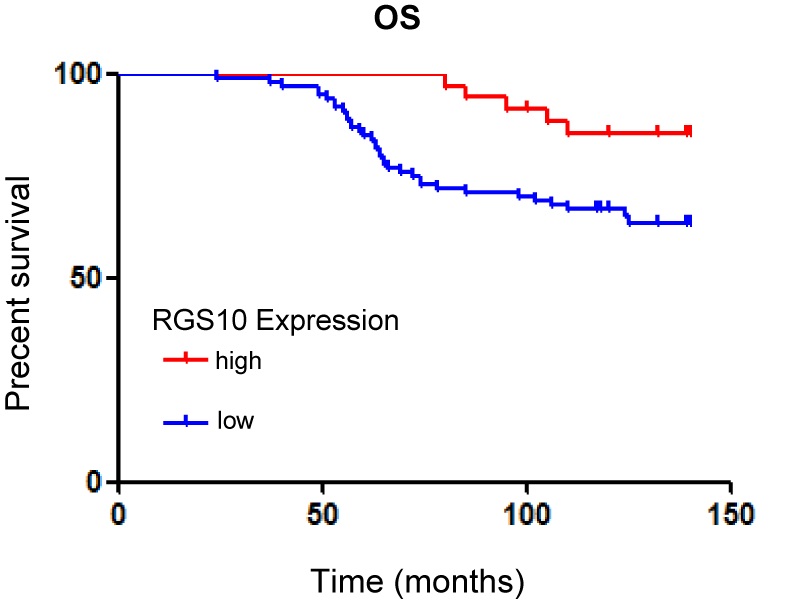

Supplement: Figure 1—source data 1. [file elife-97327-fig1-data1.zip › Figure 1-Source data 1/F1H.tif]

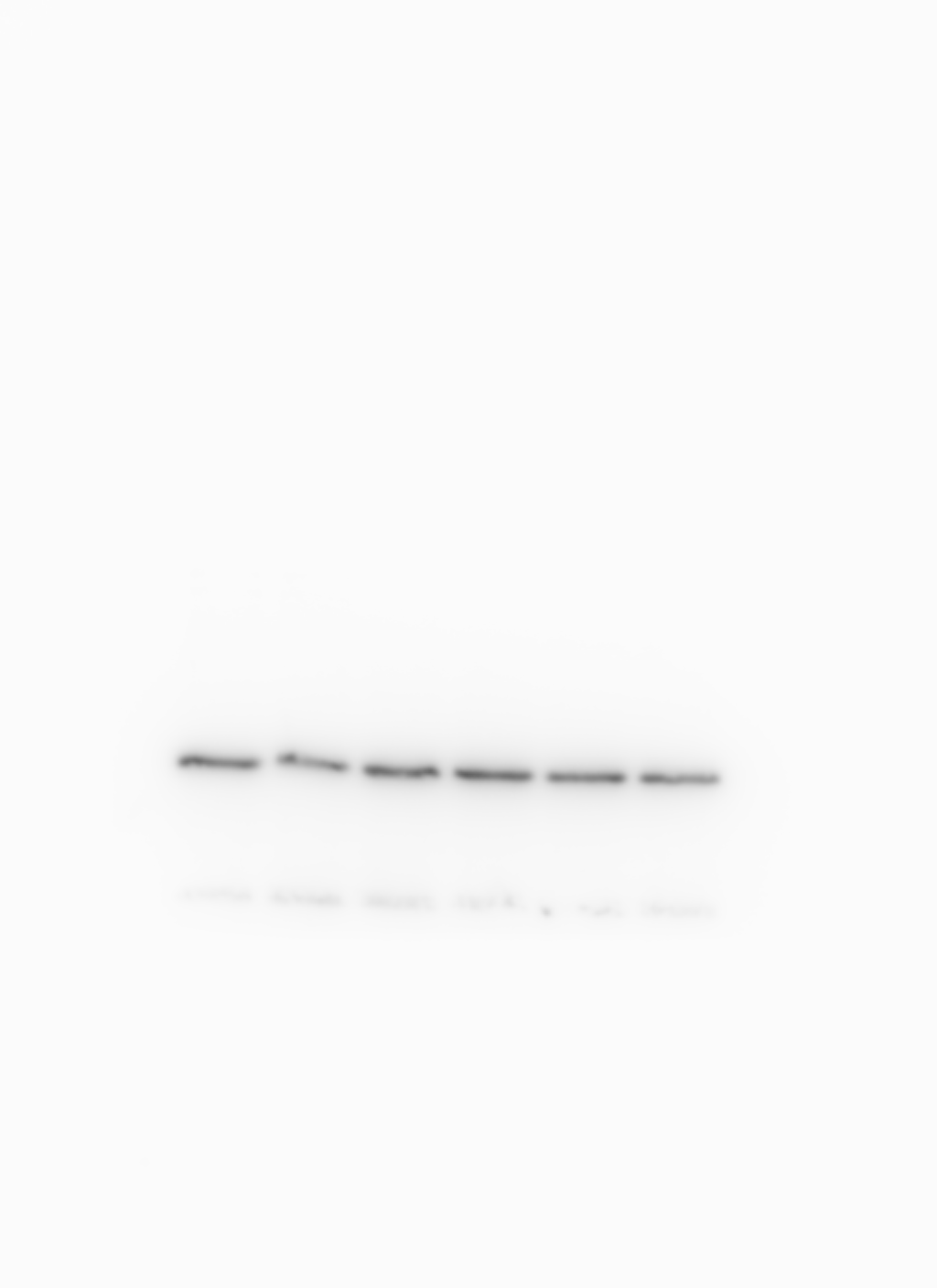

Supplement: Figure 2—source data 1. [file elife-97327-fig2-data1.zip › Source data 1/F2A-GAPDH-1.tif]

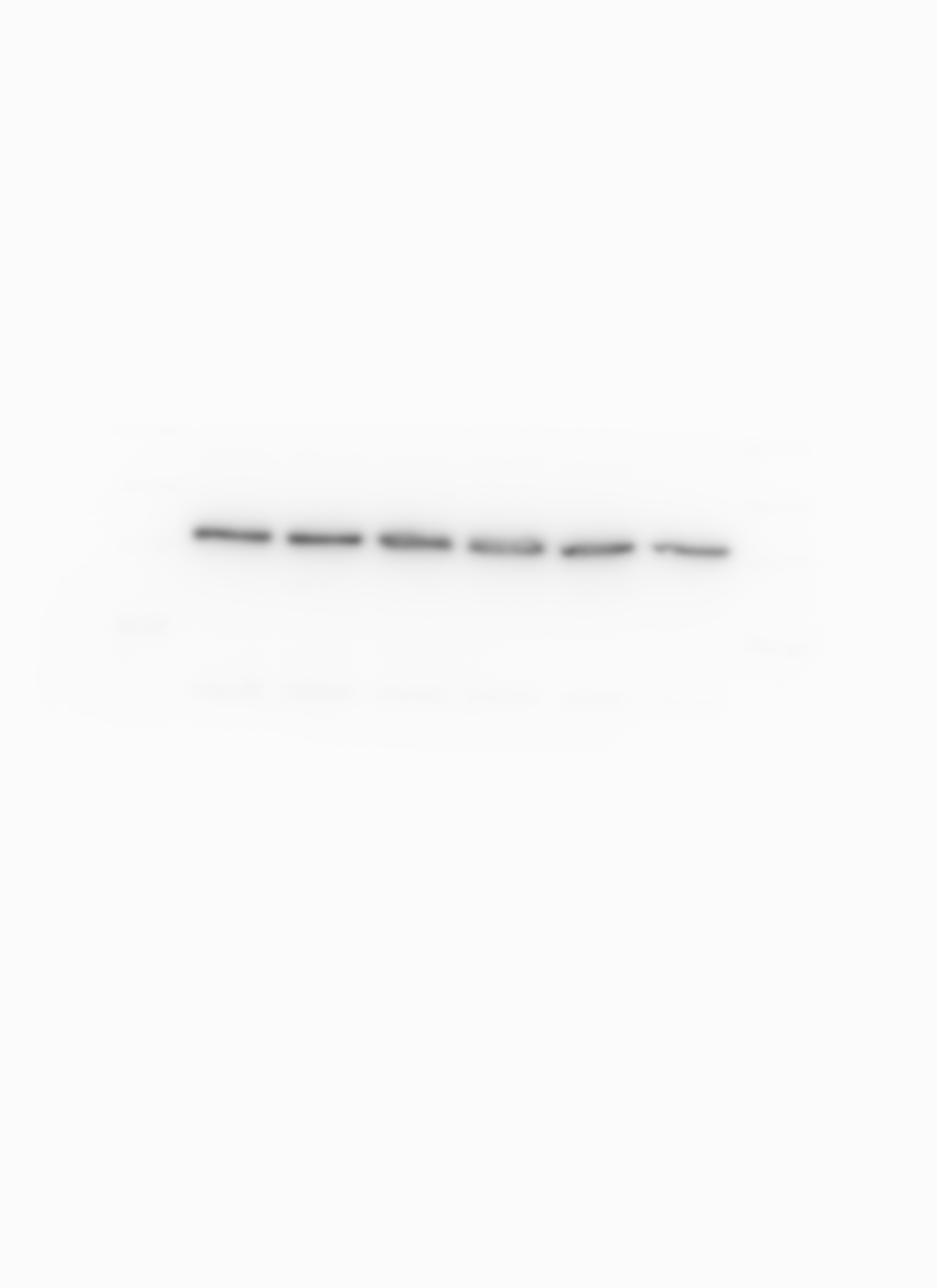

Supplement: Figure 2—source data 1. [file elife-97327-fig2-data1.zip › Source data 1/F2A-GAPDH-2.tif]

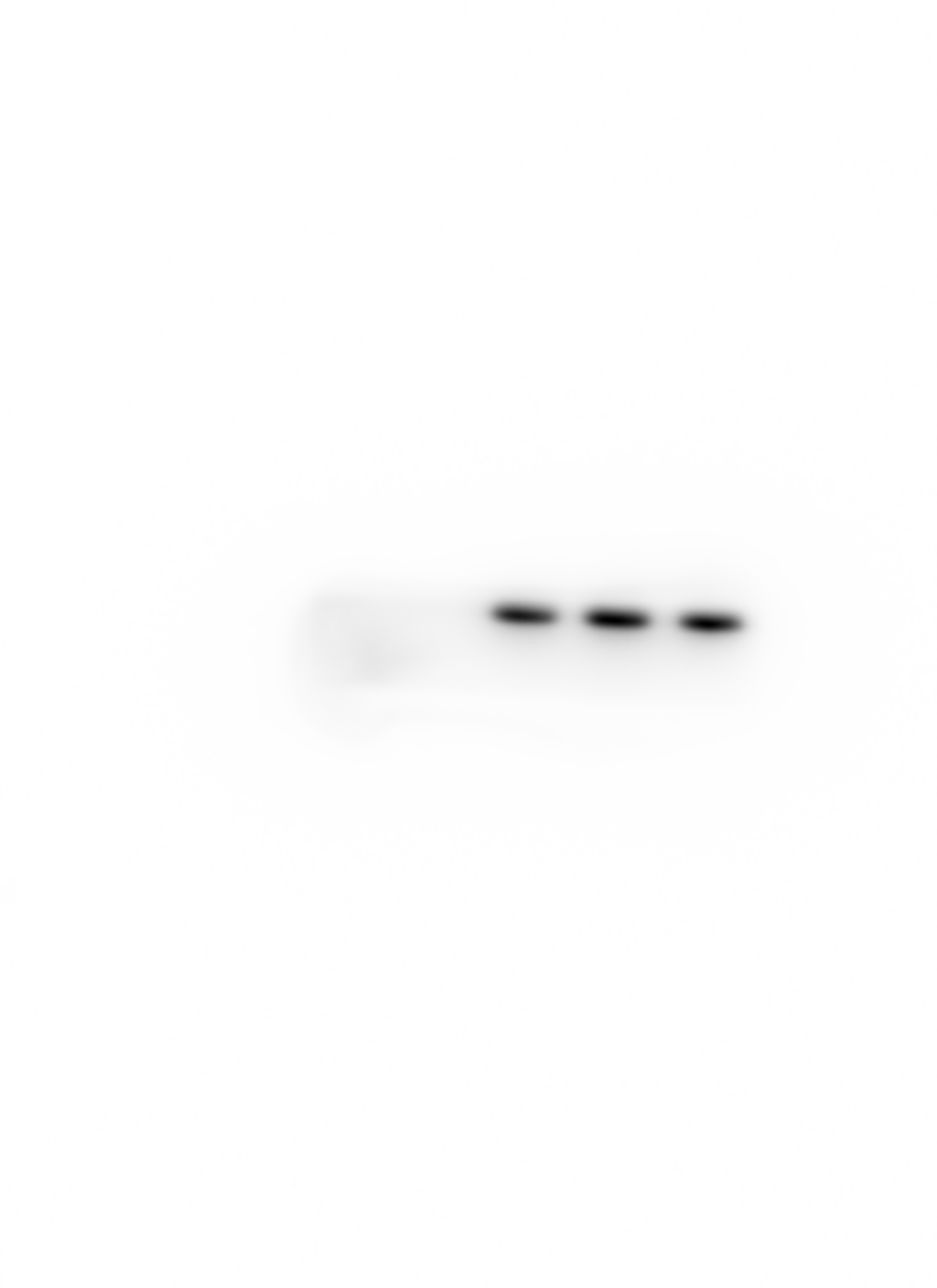

Supplement: Figure 2—source data 1. [file elife-97327-fig2-data1.zip › Source data 1/F2A-GAPDH-3.tif]

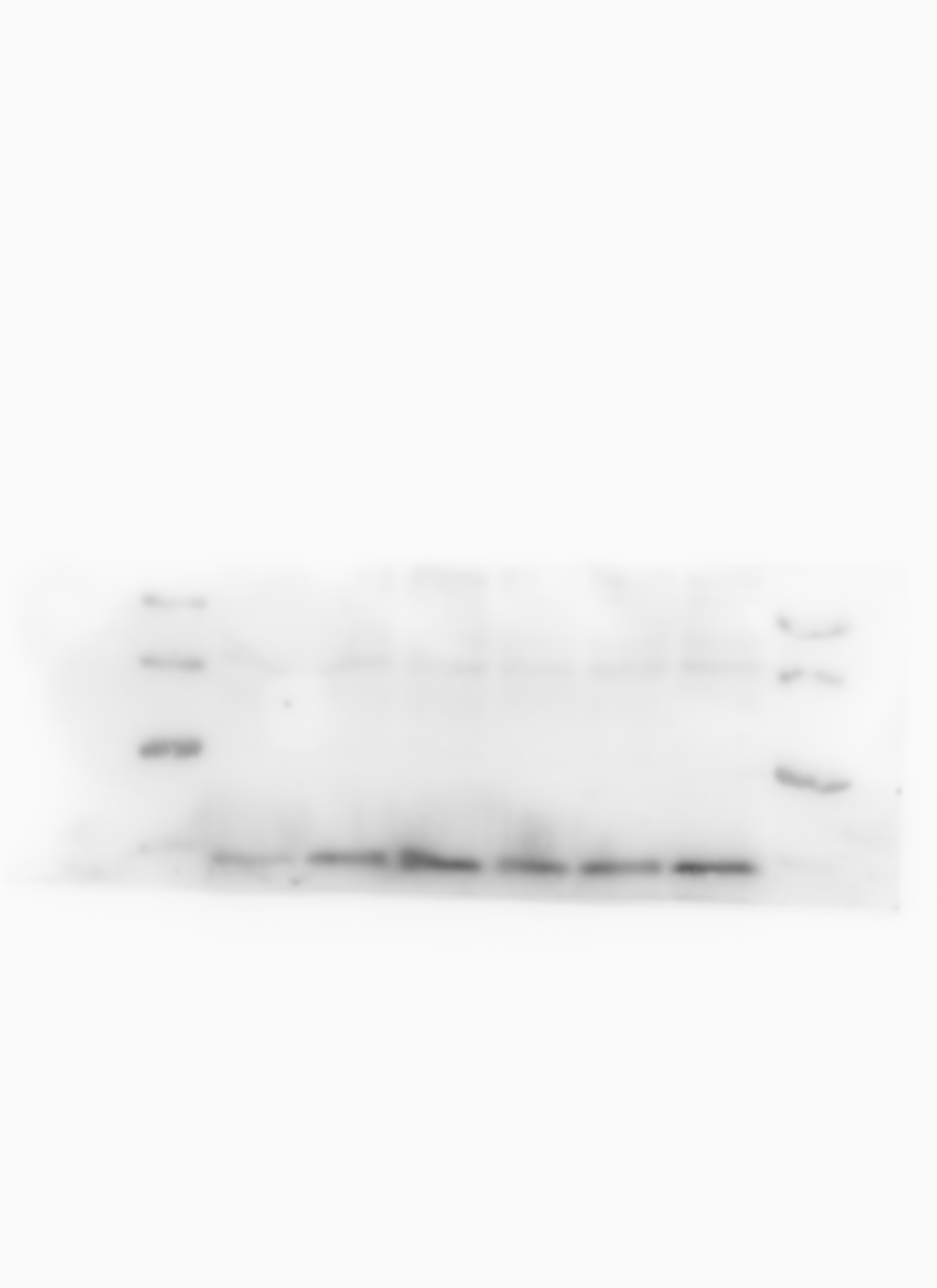

Supplement: Figure 2—source data 1. [file elife-97327-fig2-data1.zip › Source data 1/F2A-RGS10-1.tif]

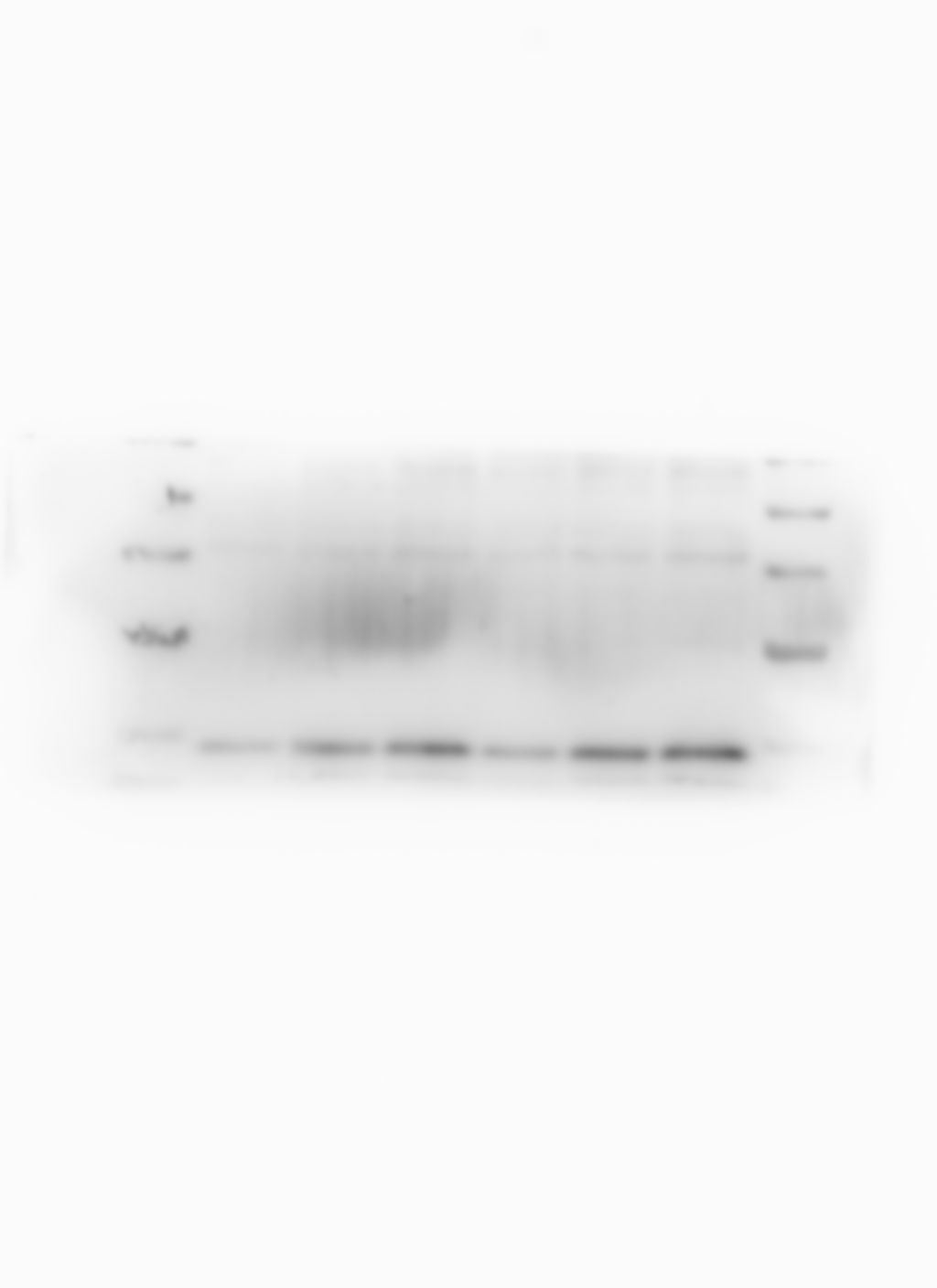

Supplement: Figure 2—source data 1. [file elife-97327-fig2-data1.zip › Source data 1/F2A-RGS10-2.tif]

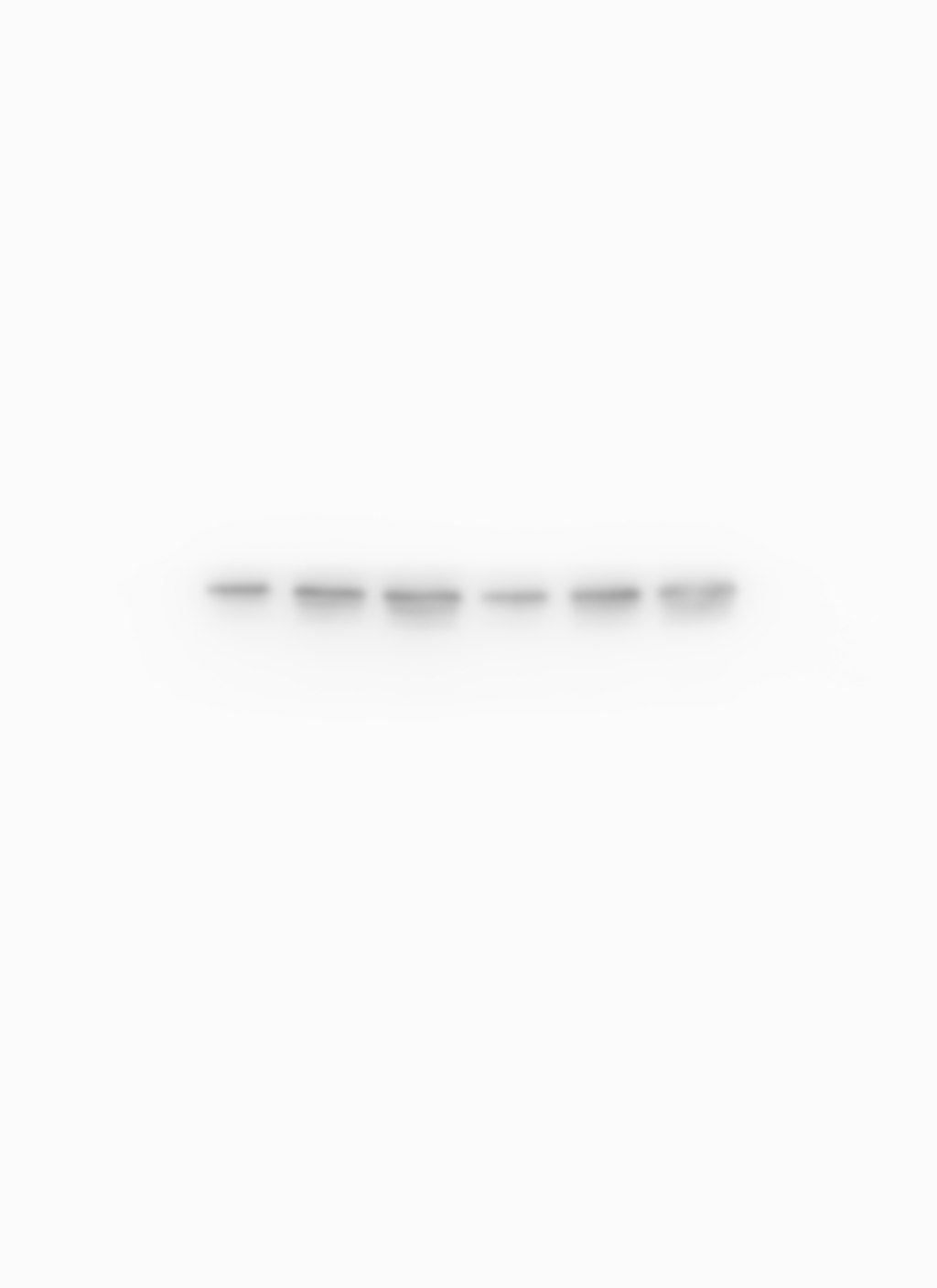

Supplement: Figure 2—source data 1. [file elife-97327-fig2-data1.zip › Source data 1/F2A-RGS10-3.tif]

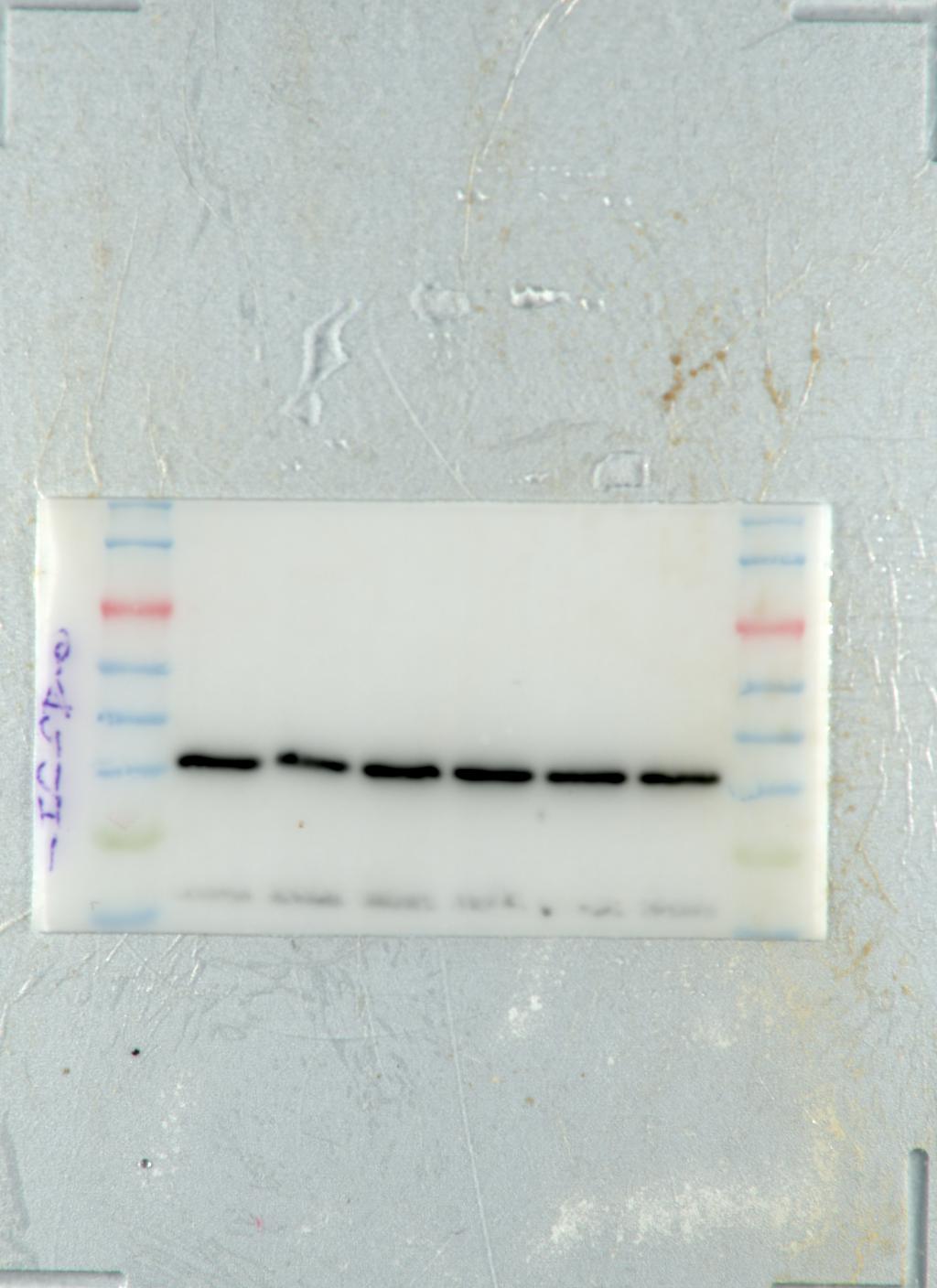

Supplement: Figure 2—source data 2. [file elife-97327-fig2-data2.zip › Source data 2/F2A-GAPDH-1-marker.jpg]

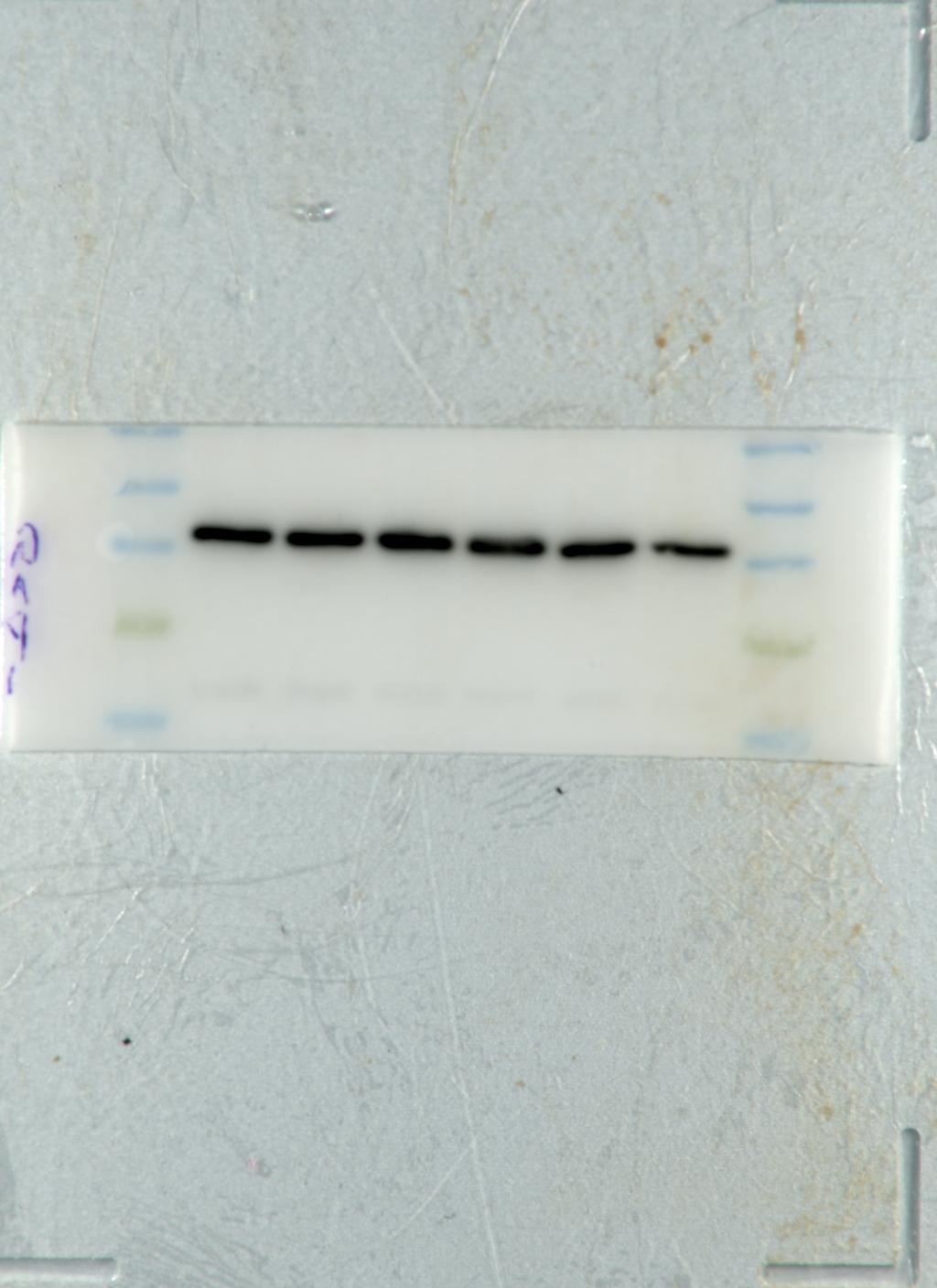

Supplement: Figure 2—source data 2. [file elife-97327-fig2-data2.zip › Source data 2/F2A-GAPDH-2 marker.jpg]

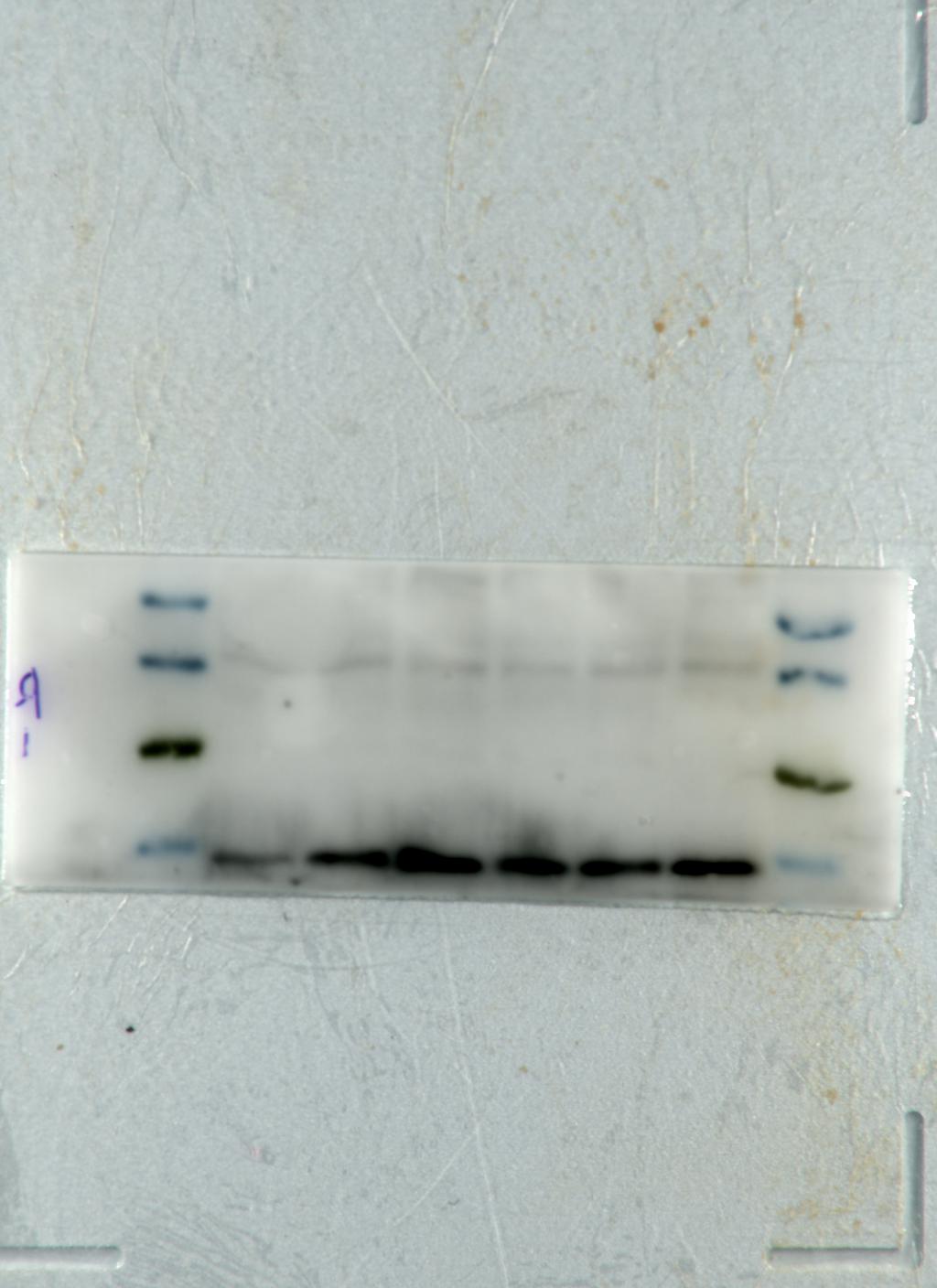

Supplement: Figure 2—source data 2. [file elife-97327-fig2-data2.zip › Source data 2/F2A-RGS10 -1+Marker.jpg]

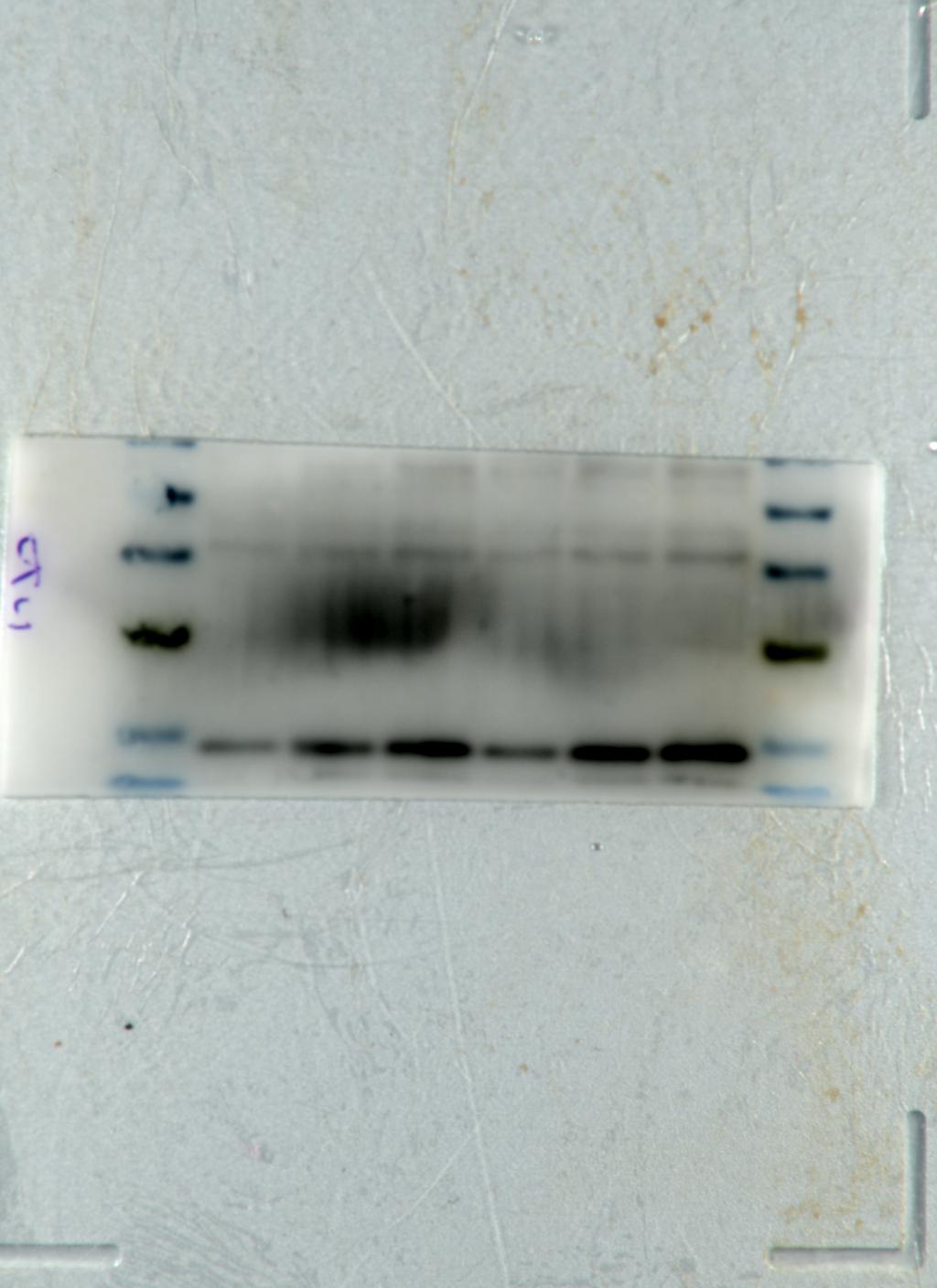

Supplement: Figure 2—source data 2. [file elife-97327-fig2-data2.zip › Source data 2/F2A-RGS10-2+Marker.jpg]

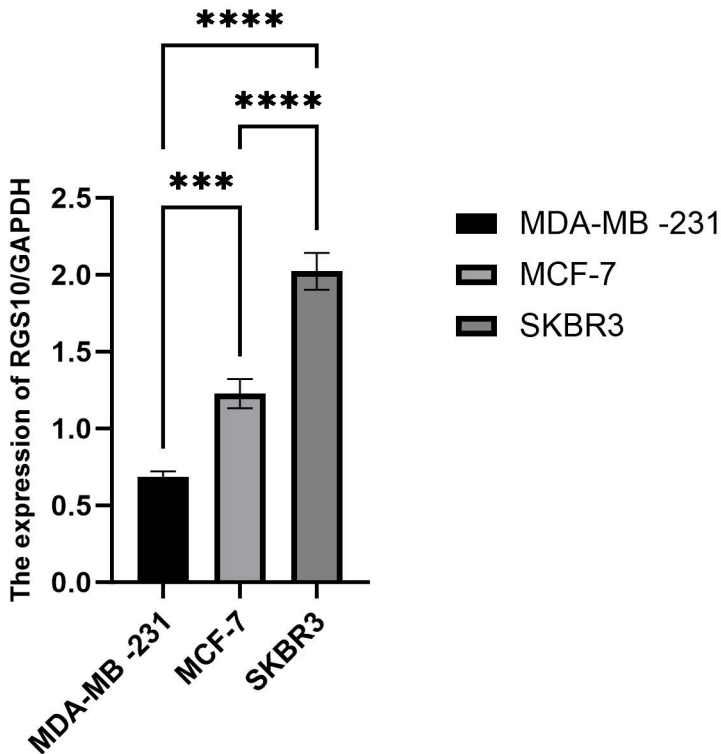

Supplement: Figure 2—source data 3. [file elife-97327-fig2-data3.pdf]

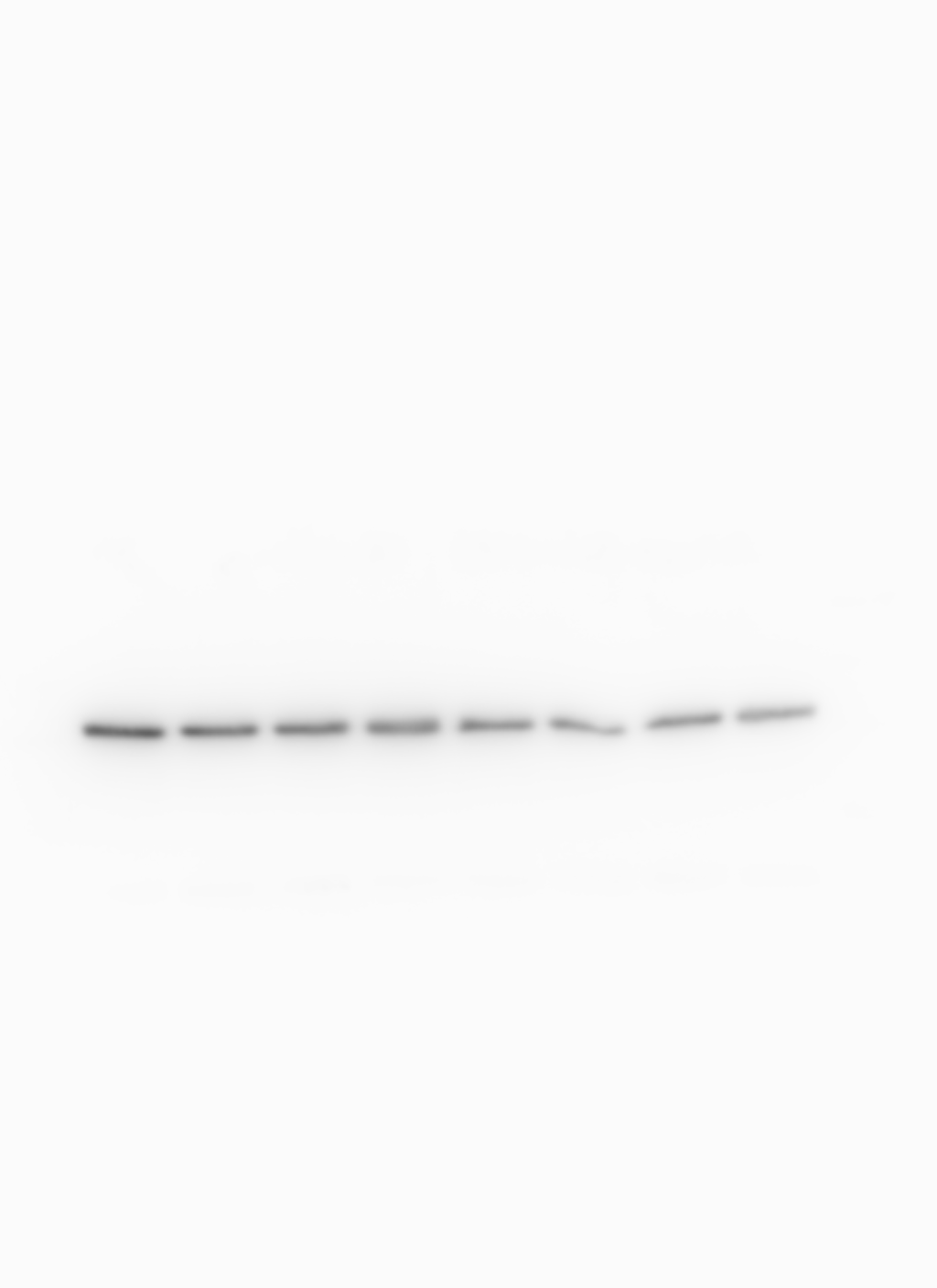

Supplement: Figure 2—source data 4. [file elife-97327-fig2-data4.zip › Source data 4/F2B-GAPDH- 2.tif]

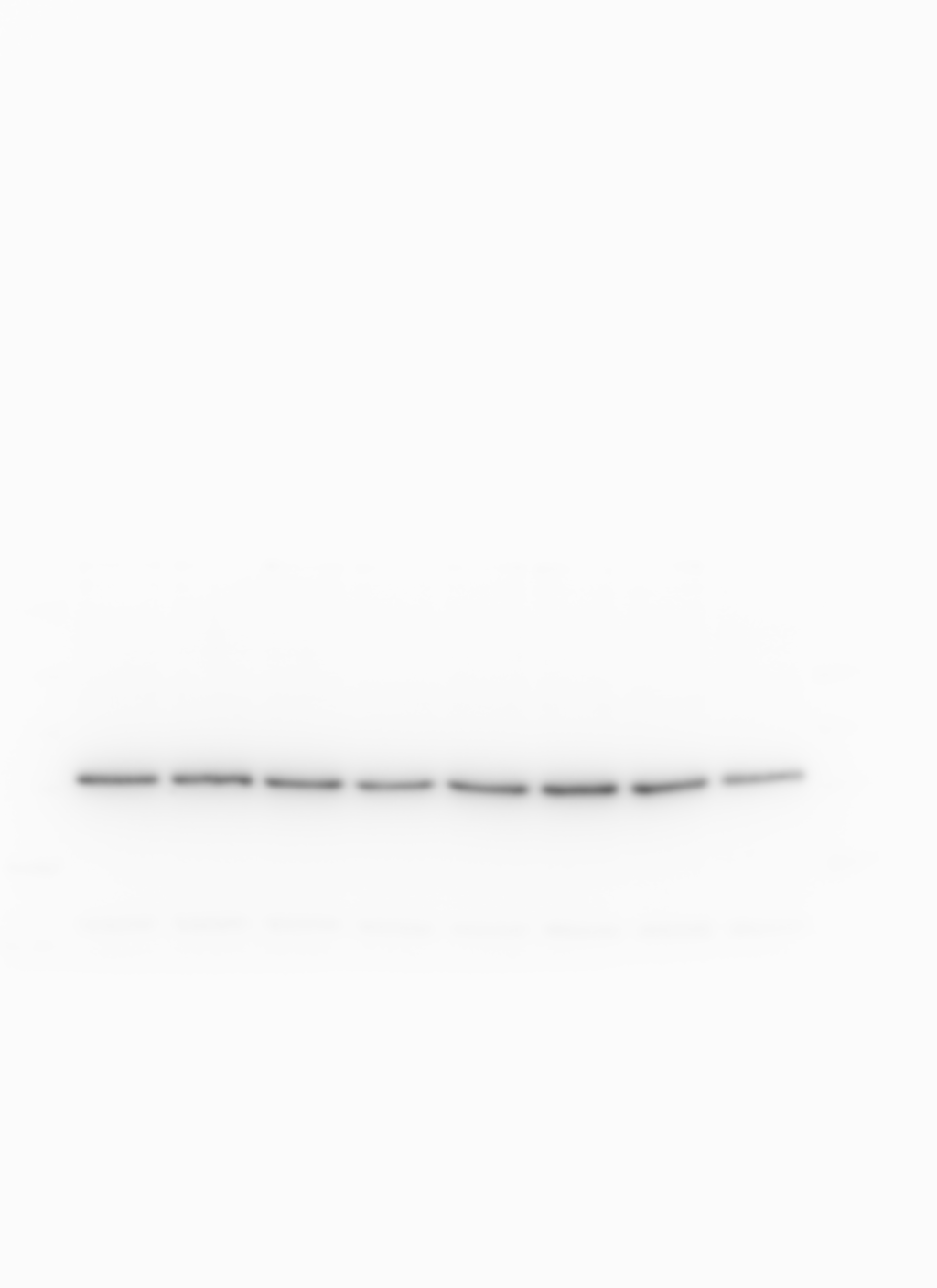

Supplement: Figure 2—source data 4. [file elife-97327-fig2-data4.zip › Source data 4/F2B-GAPDH-1 .tif]

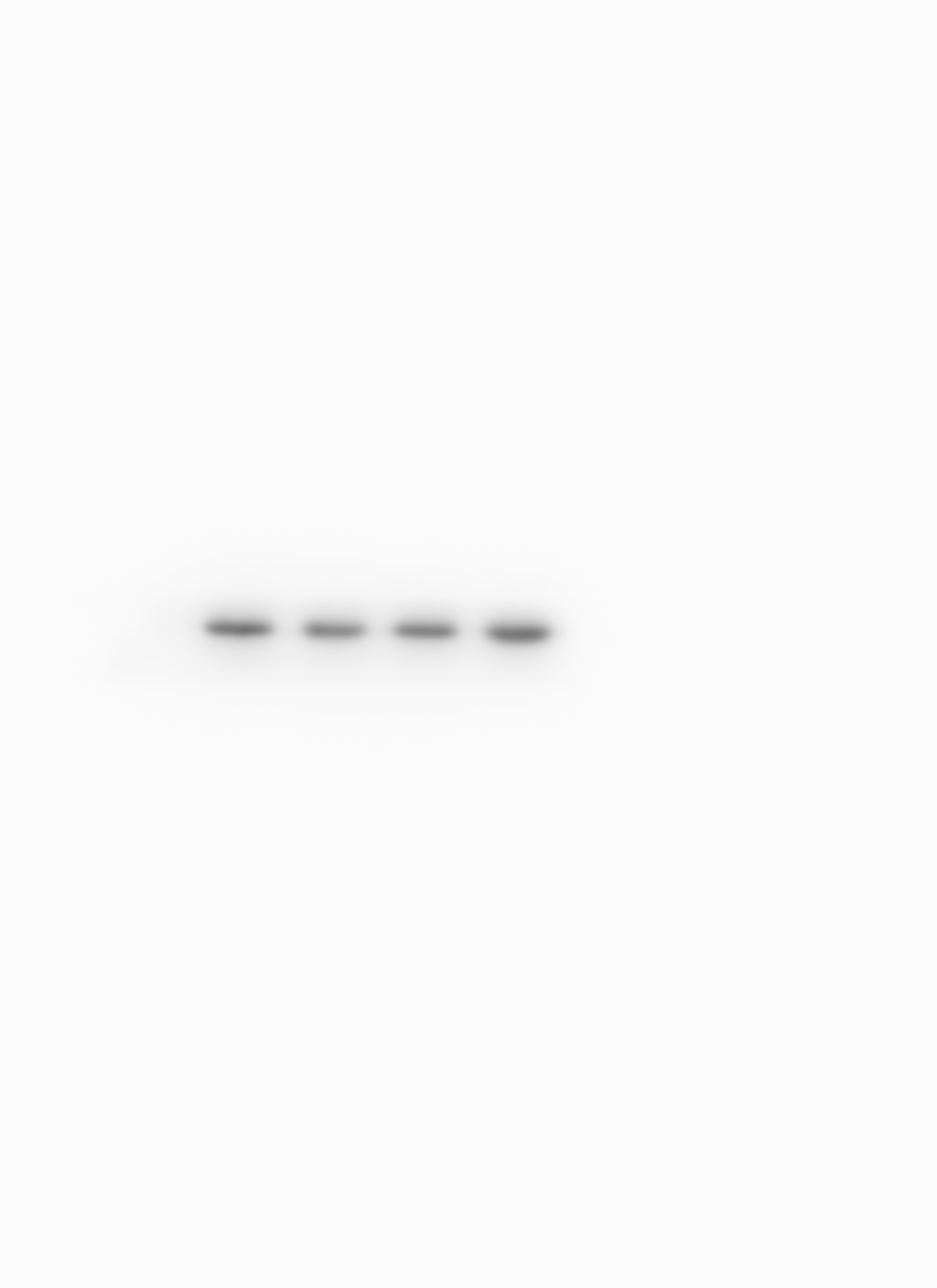

Supplement: Figure 2—source data 4. [file elife-97327-fig2-data4.zip › Source data 4/F2B-GAPDH-3.tif]

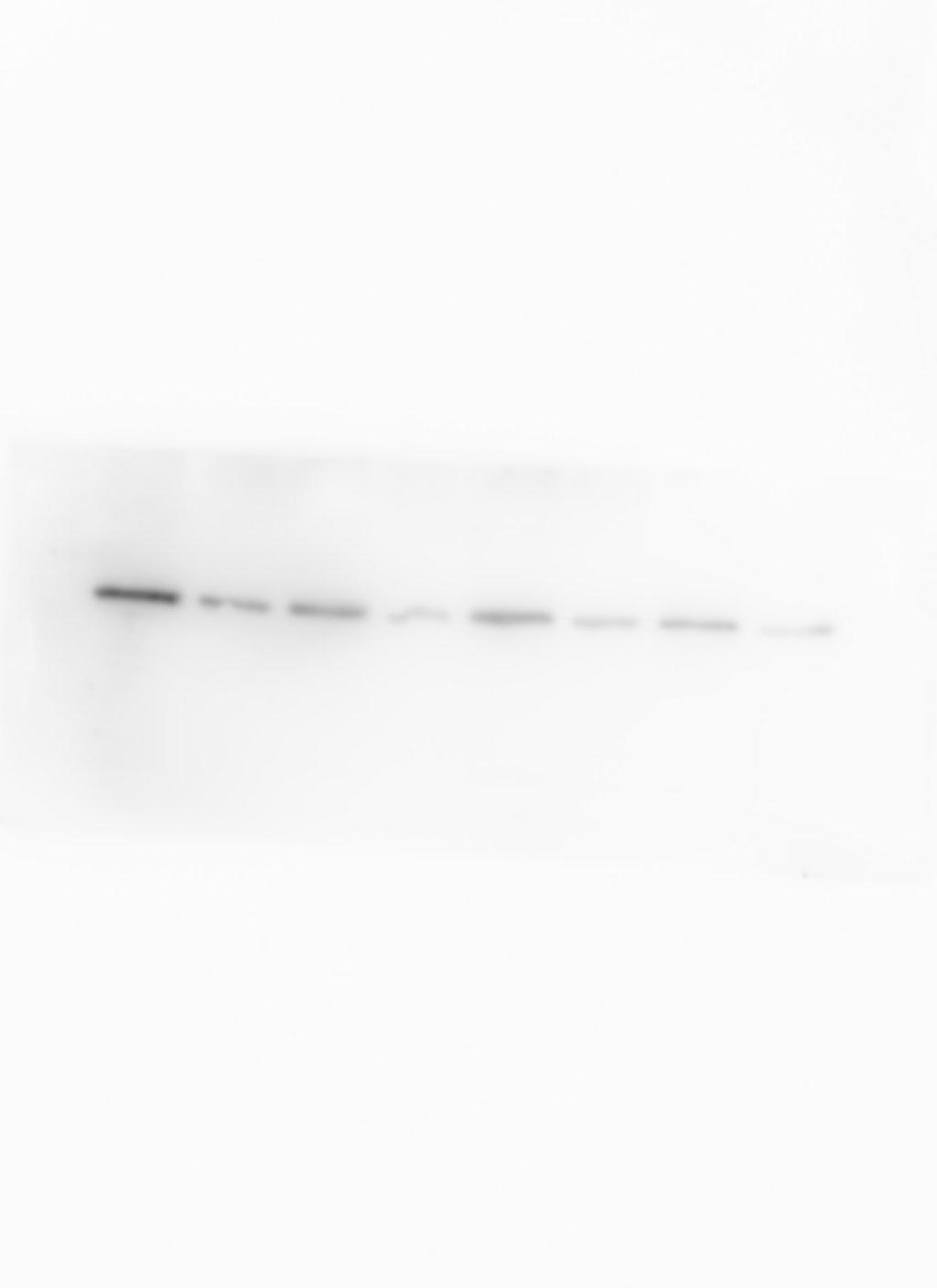

Supplement: Figure 2—source data 4. [file elife-97327-fig2-data4.zip › Source data 4/F2B-RGS10-1.tif]

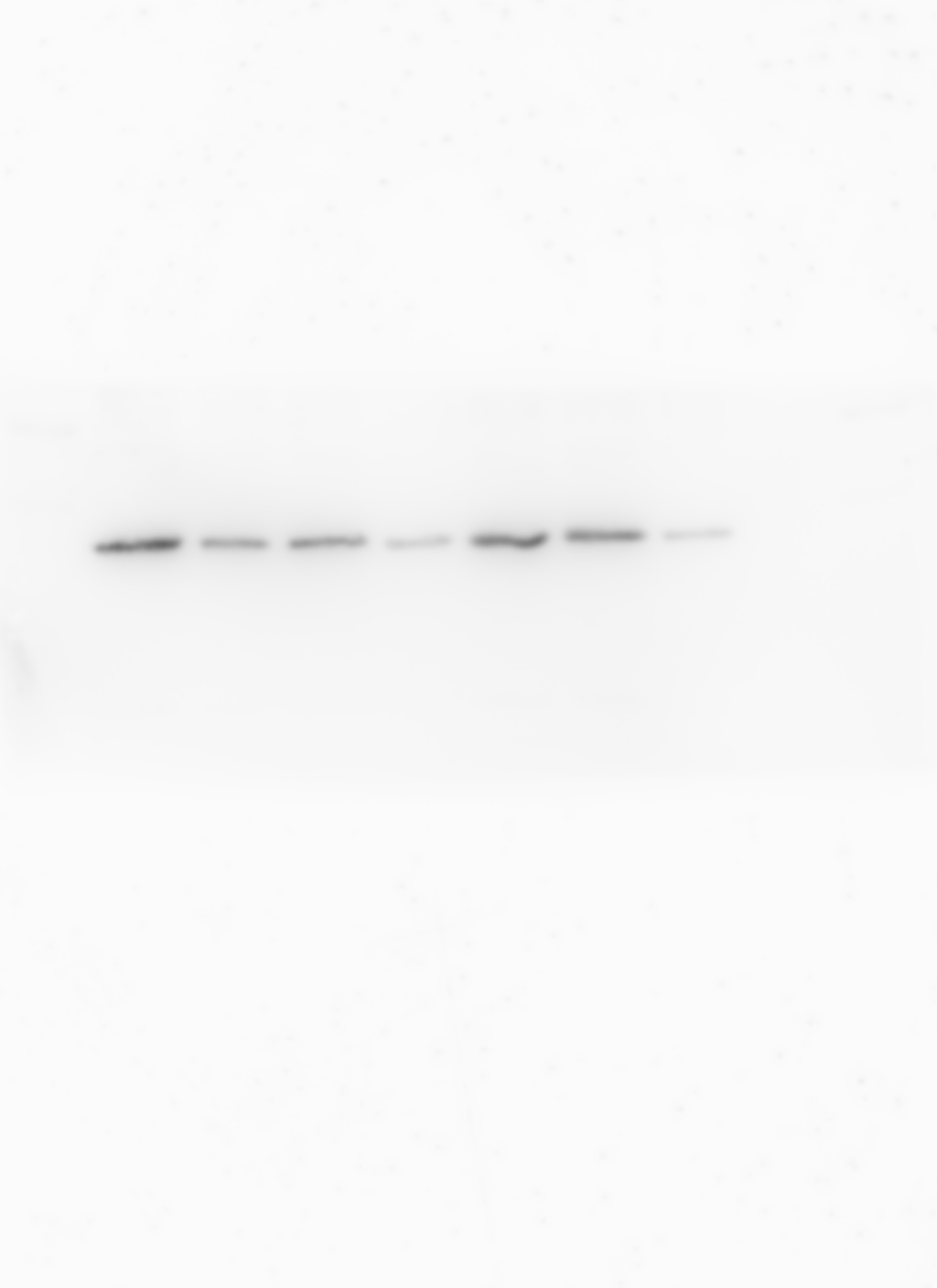

Supplement: Figure 2—source data 4. [file elife-97327-fig2-data4.zip › Source data 4/F2B-RGS10-2.tif]

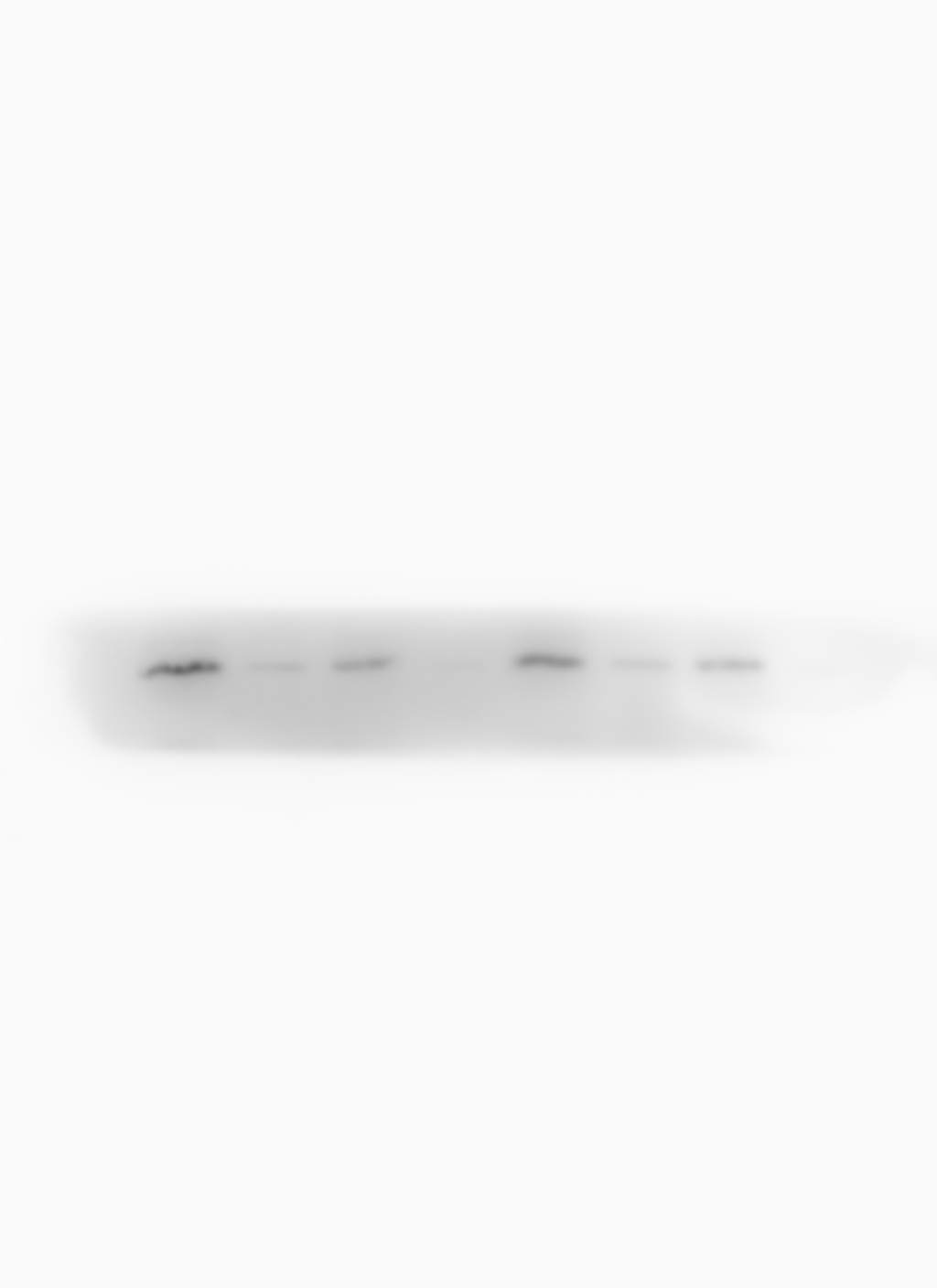

Supplement: Figure 2—source data 4. [file elife-97327-fig2-data4.zip › Source data 4/F2B-RGS10-3.tif]

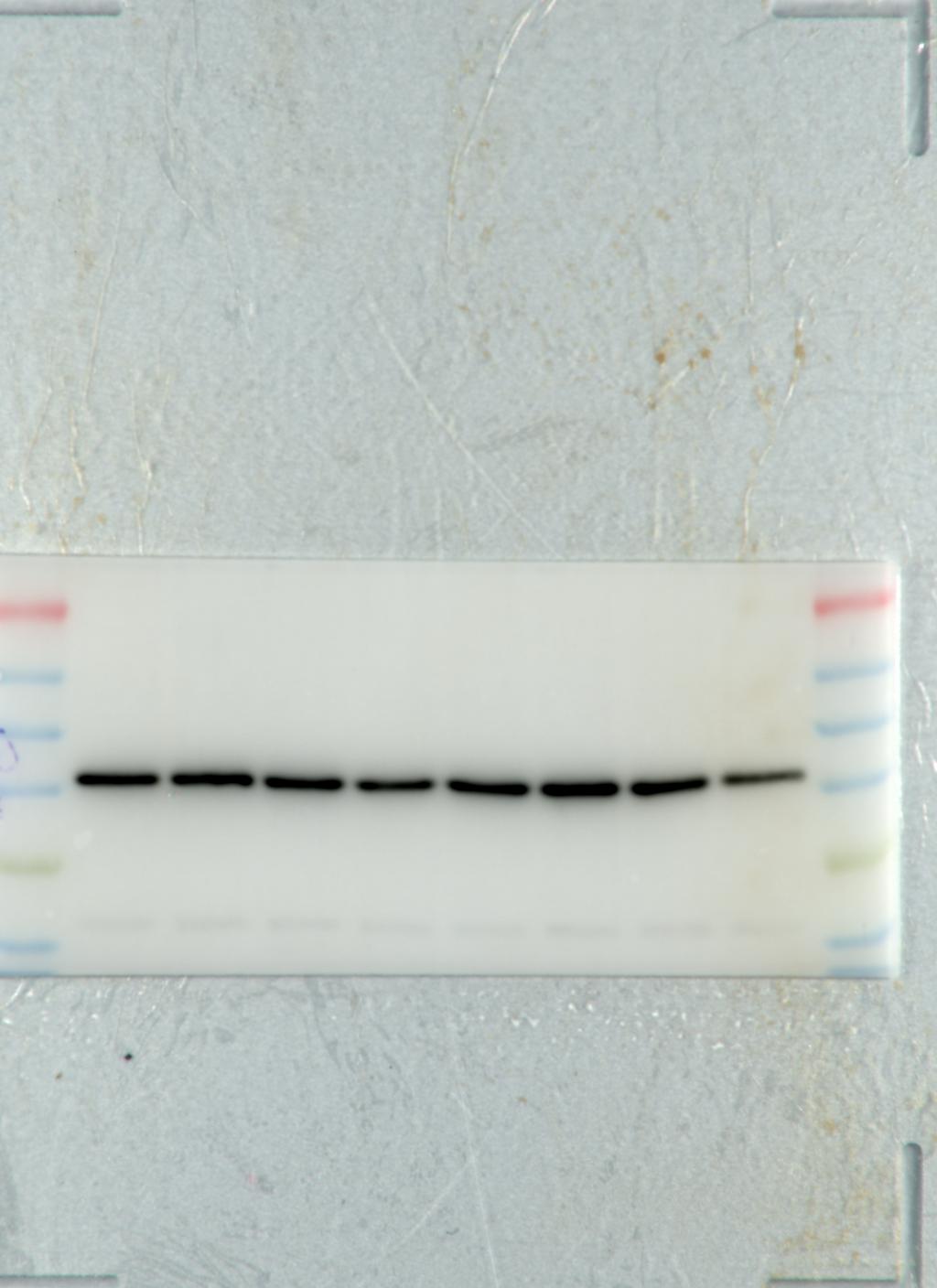

Supplement: Figure 2—source data 5. [file elife-97327-fig2-data5.zip › Source data 5/F2B-GAPDH-1 +Marker.jpg]

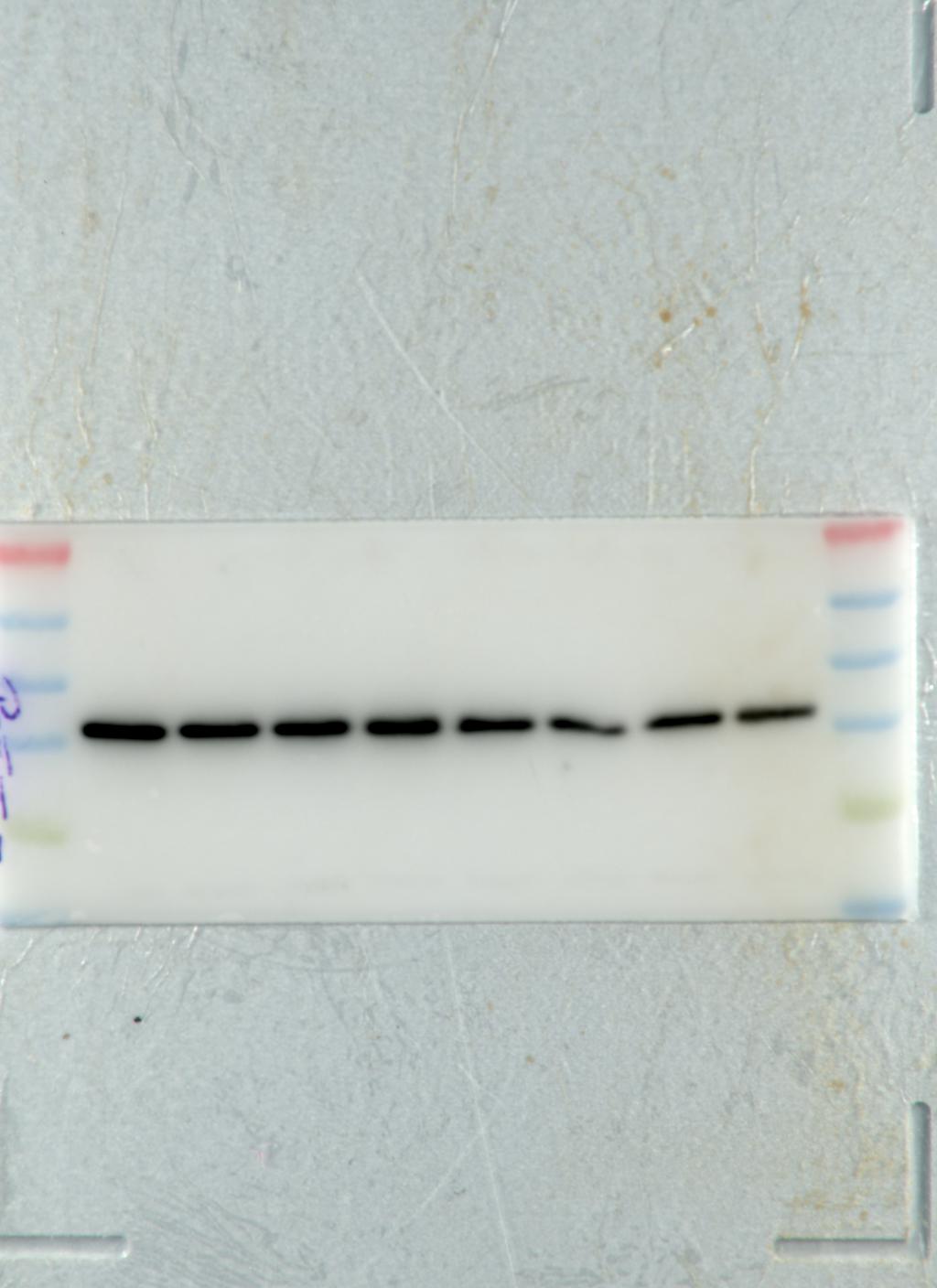

Supplement: Figure 2—source data 5. [file elife-97327-fig2-data5.zip › Source data 5/F2B-GAPDH-2+Marker.jpg]

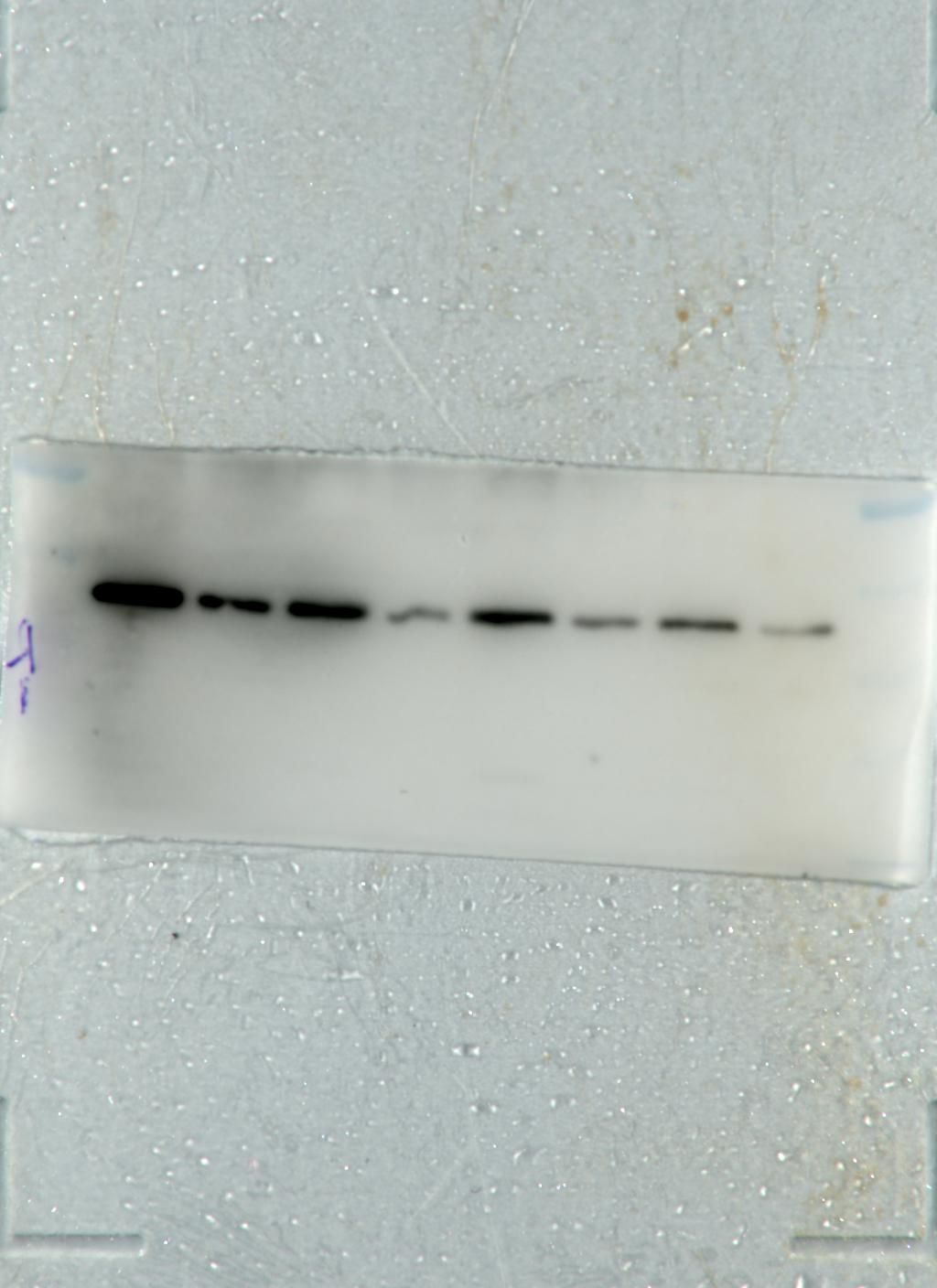

Supplement: Figure 2—source data 5. [file elife-97327-fig2-data5.zip › Source data 5/F2B-RGS10-1 +Marker.jpg]

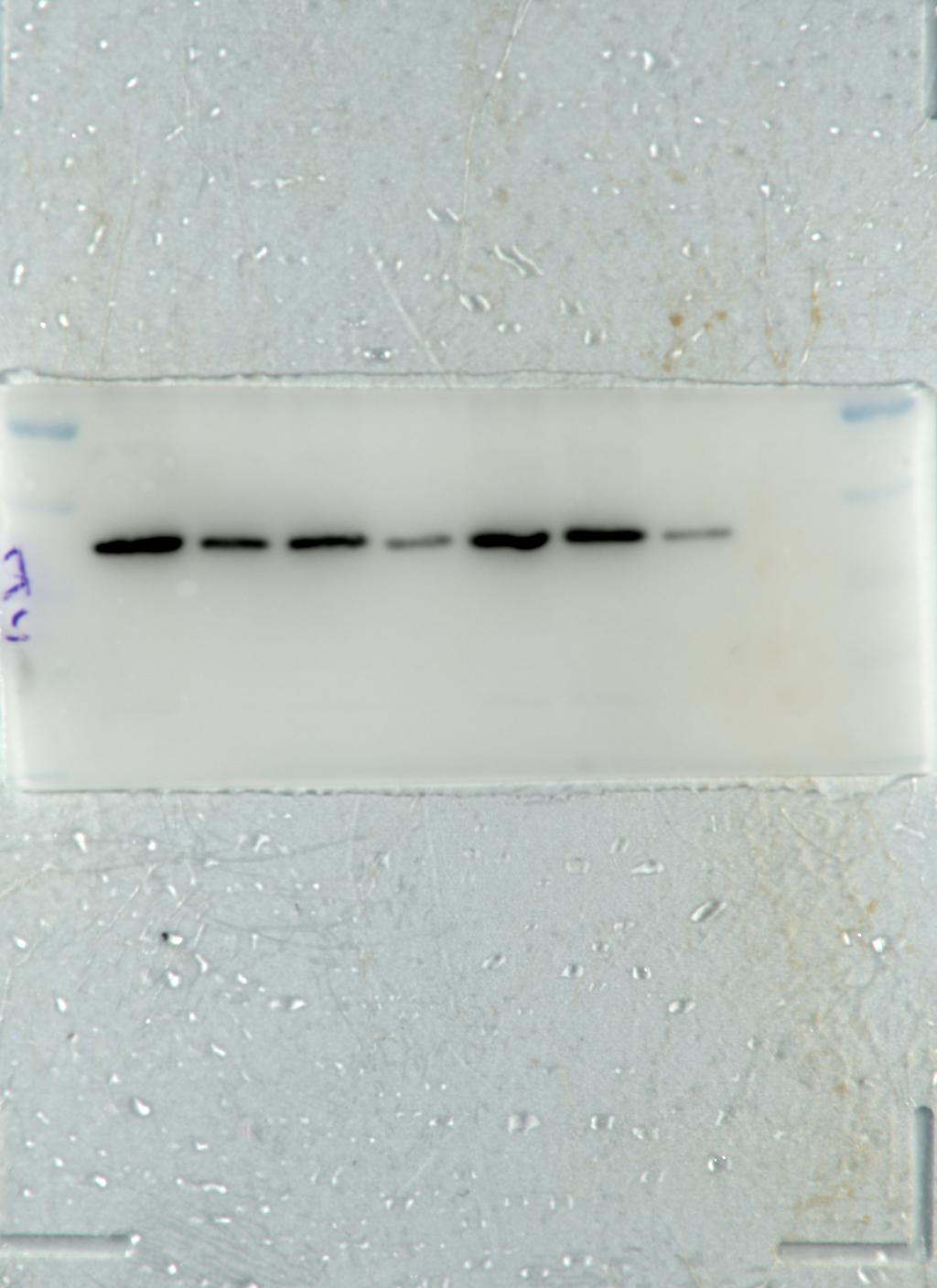

Supplement: Figure 2—source data 5. [file elife-97327-fig2-data5.zip › Source data 5/F2B-RGS10-2+Marker.jpg]

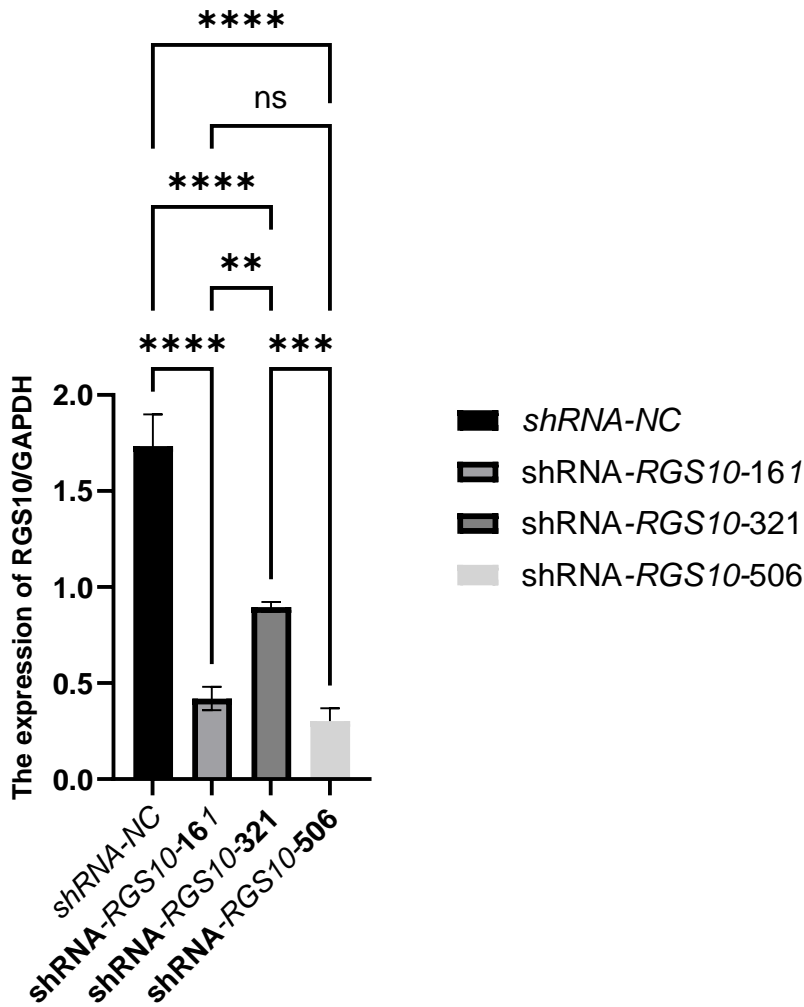

Supplement: Figure 2—source data 6. [file elife-97327-fig2-data6.pdf]

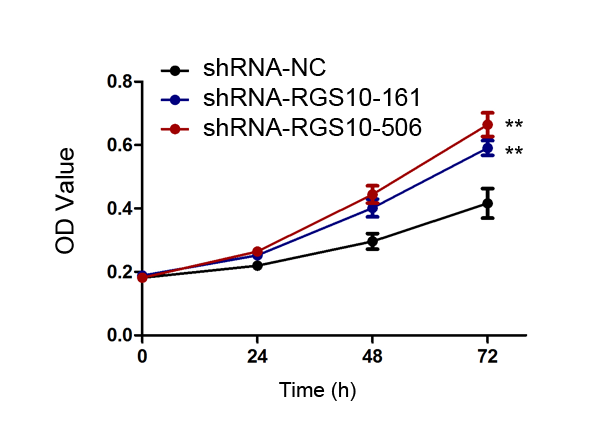

Supplement: Figure 2—source data 7. [file elife-97327-fig2-data7.zip › Source data 7/F2C-OD.tif]

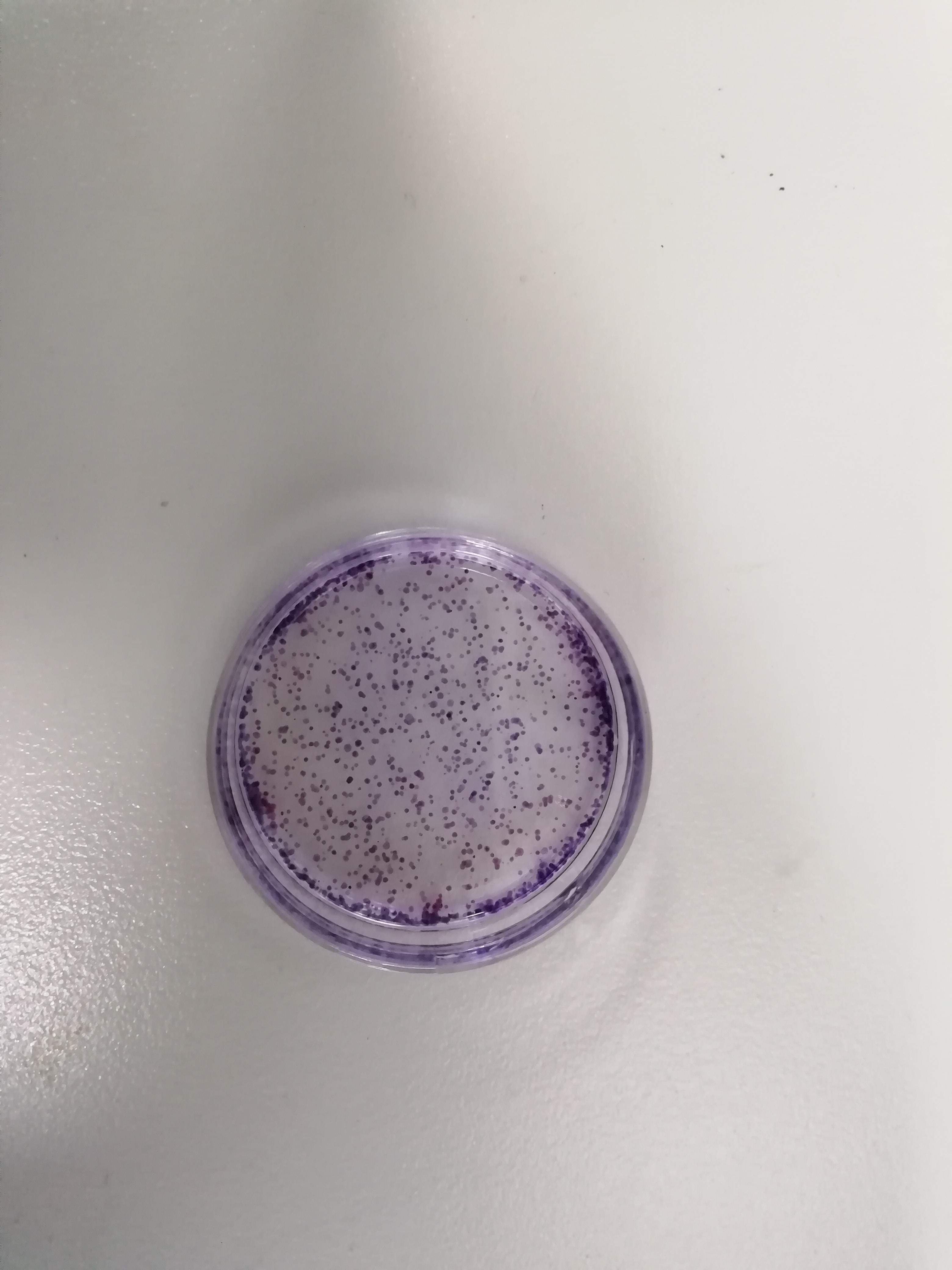

Supplement: Figure 2—source data 8. [file elife-97327-fig2-data8.zip › Source data 8/F2D-161.jpg]

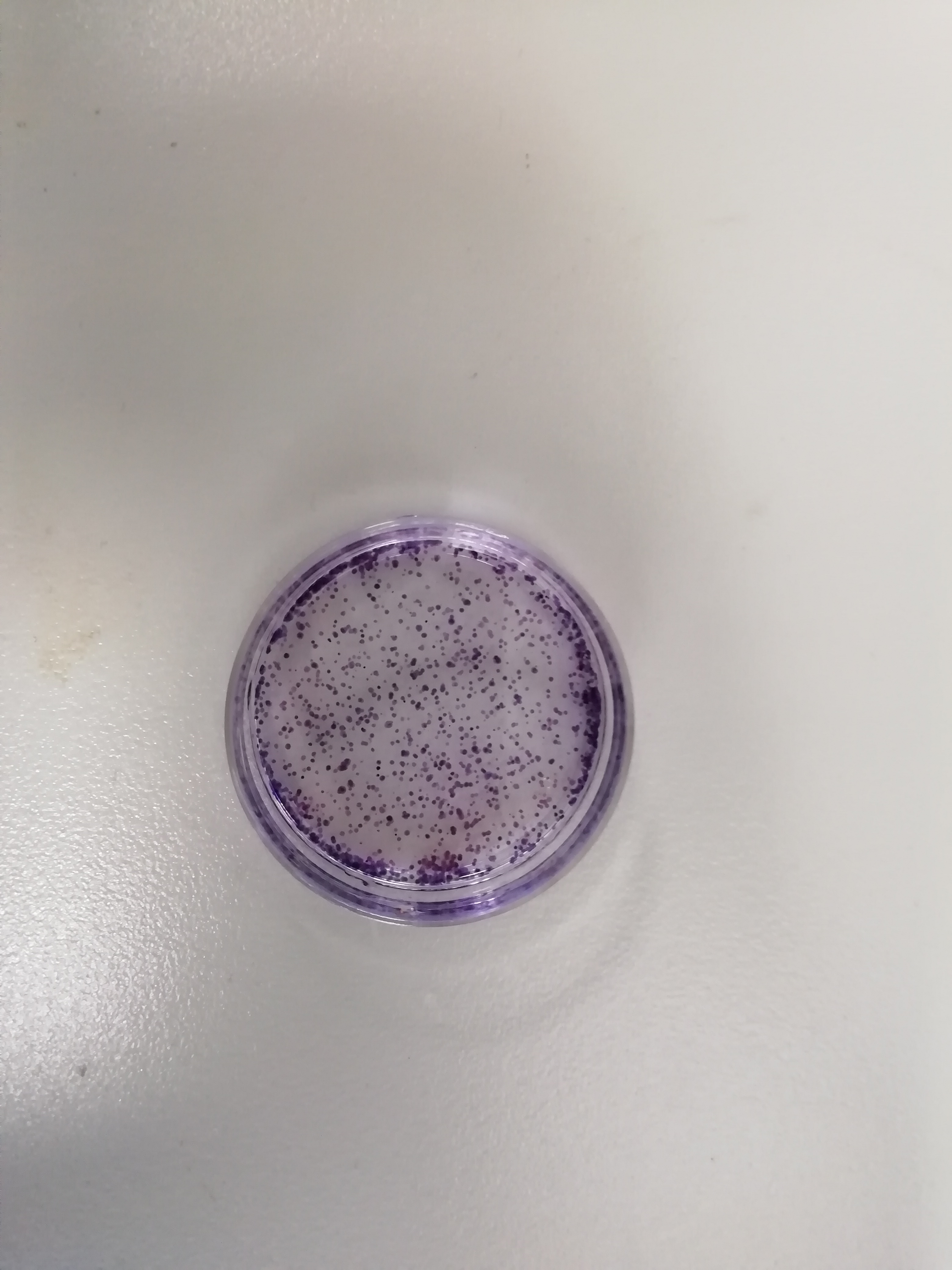

Supplement: Figure 2—source data 8. [file elife-97327-fig2-data8.zip › Source data 8/F2D-506.jpg]

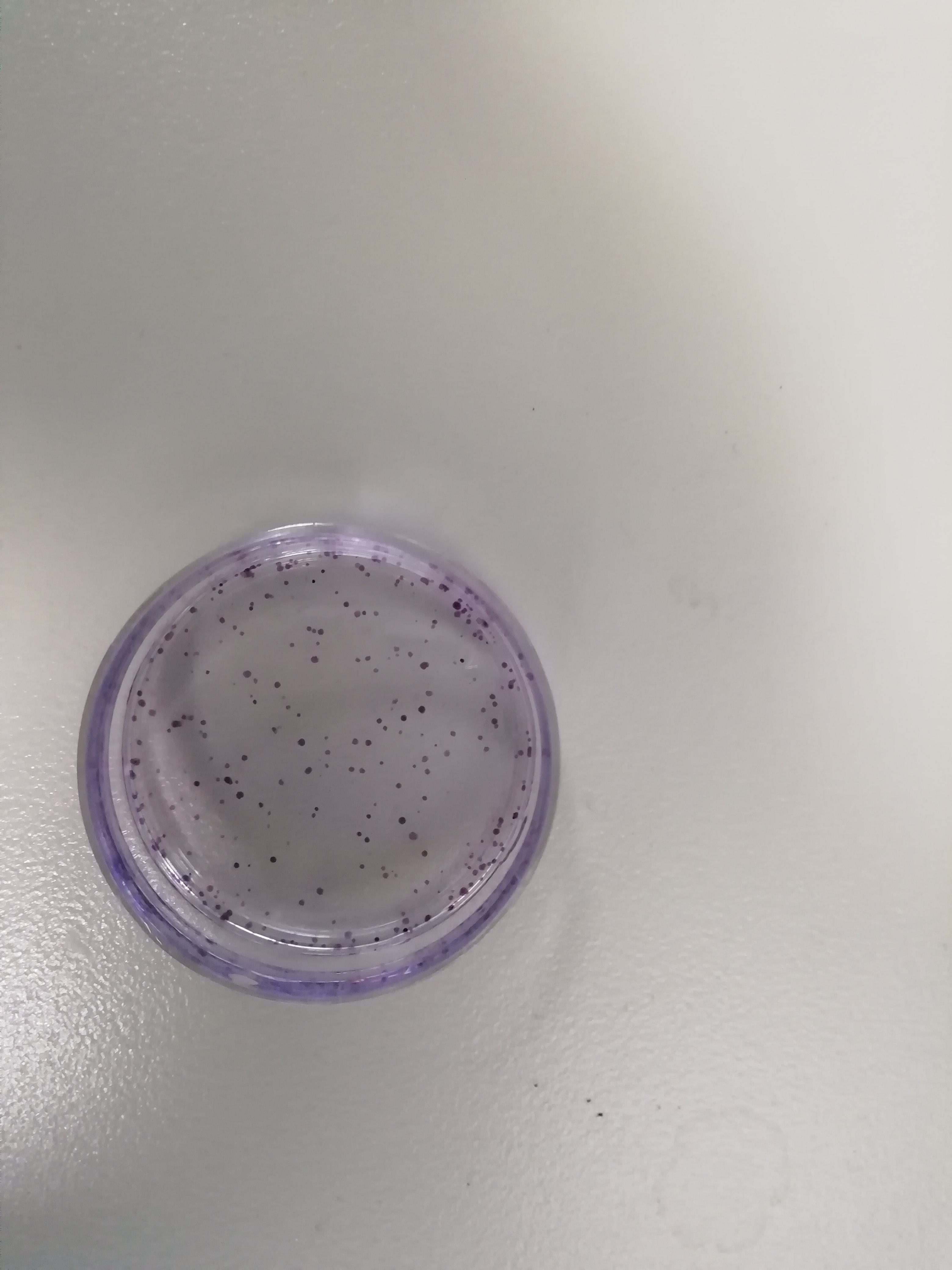

Supplement: Figure 2—source data 8. [file elife-97327-fig2-data8.zip › Source data 8/F2D-nc.jpg]

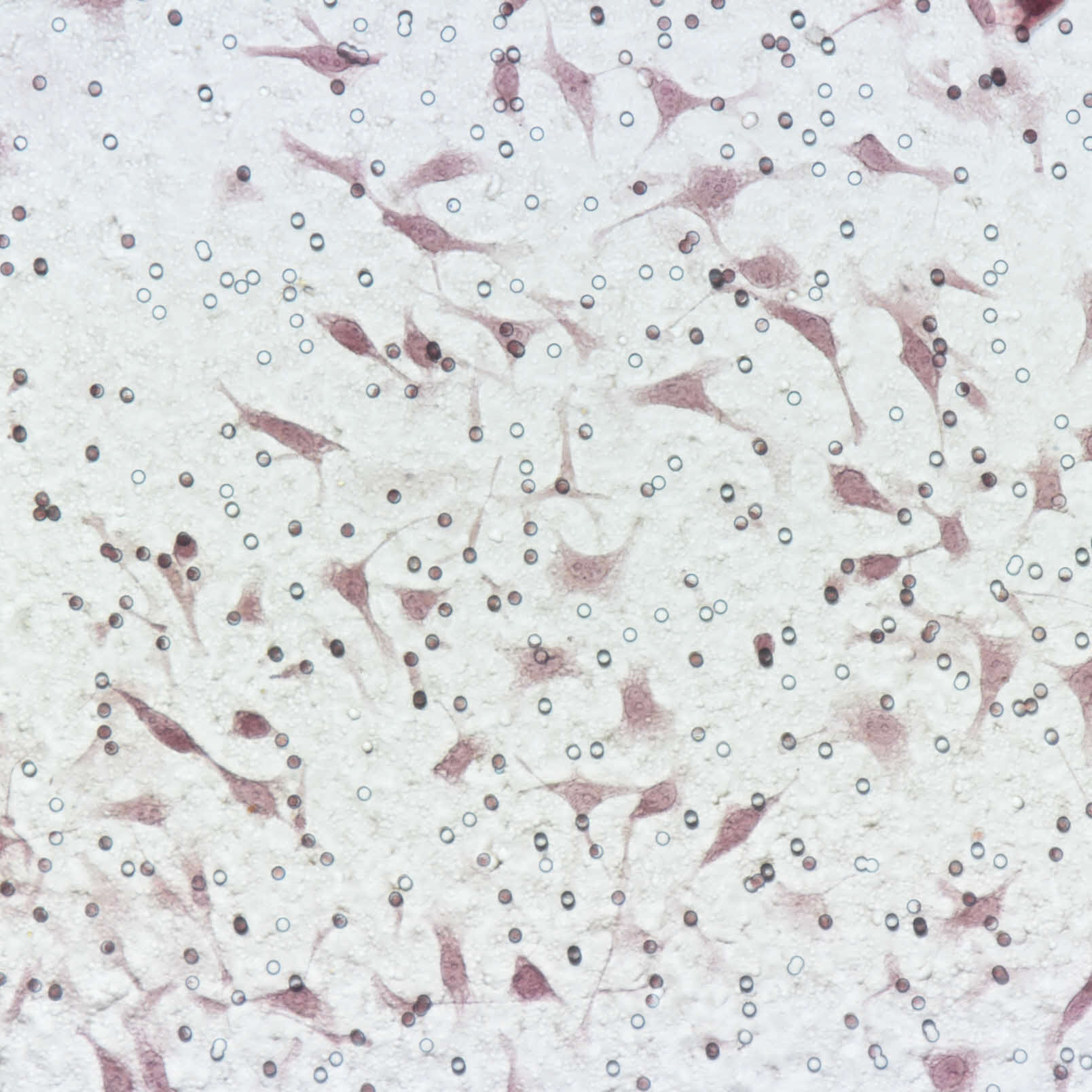

Supplement: Figure 2—source data 9. [file elife-97327-fig2-data9.zip › Source data 9/F2E-4-4-506-i.jpg]

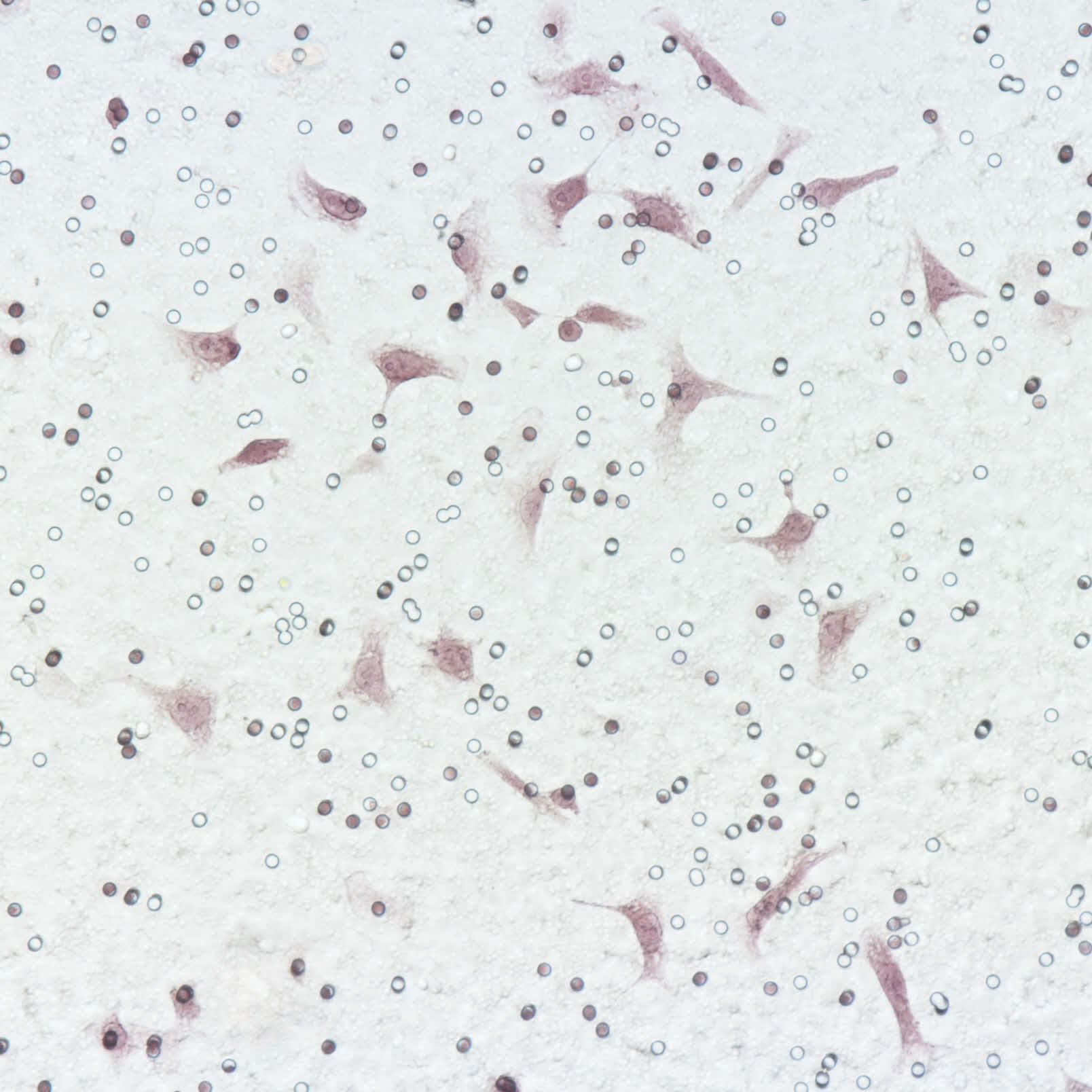

Supplement: Figure 2—source data 9. [file elife-97327-fig2-data9.zip › Source data 9/F2E-4-5-3-161-i.jpg]

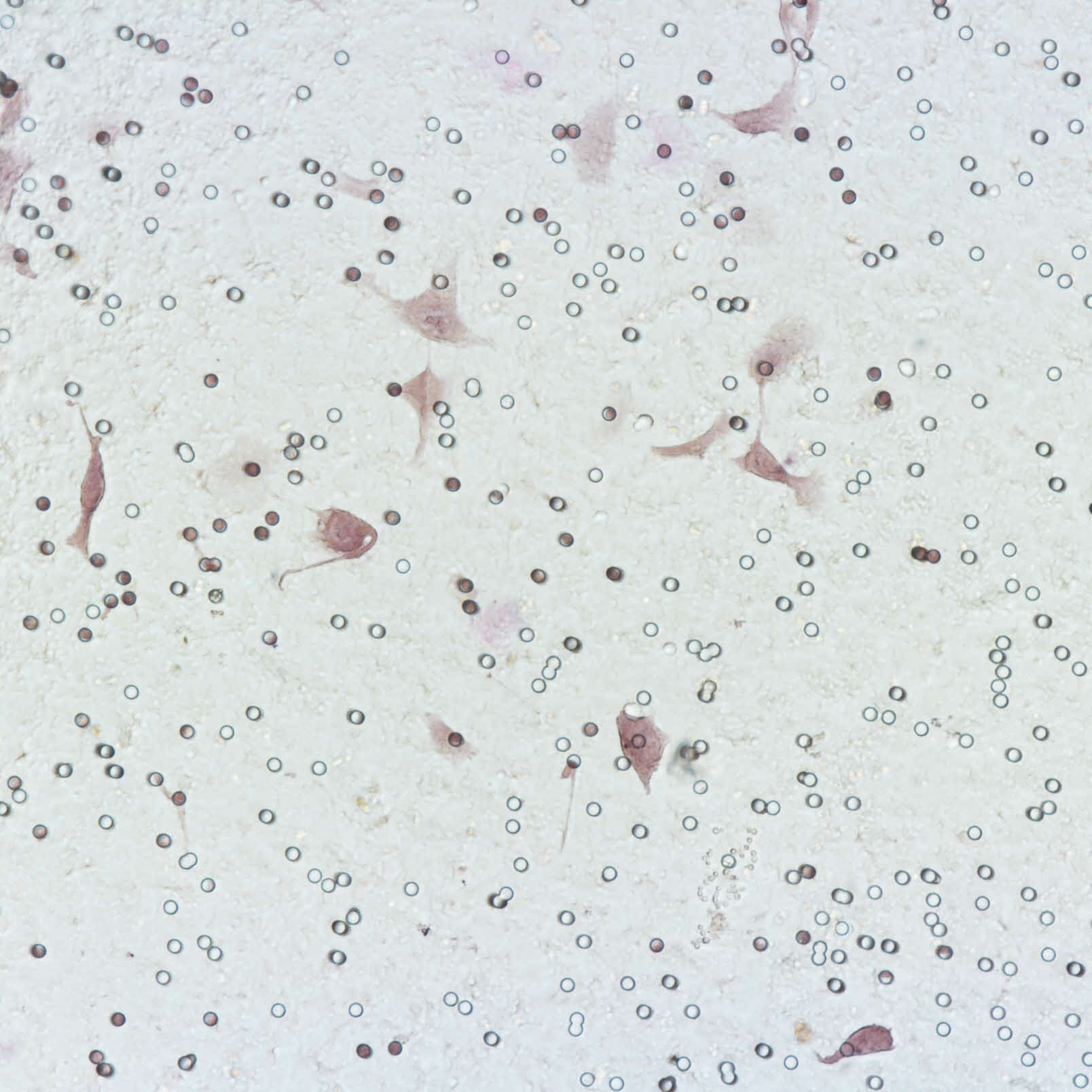

Supplement: Figure 2—source data 9. [file elife-97327-fig2-data9.zip › Source data 9/F2E-4-6-3-nc-i.jpg]

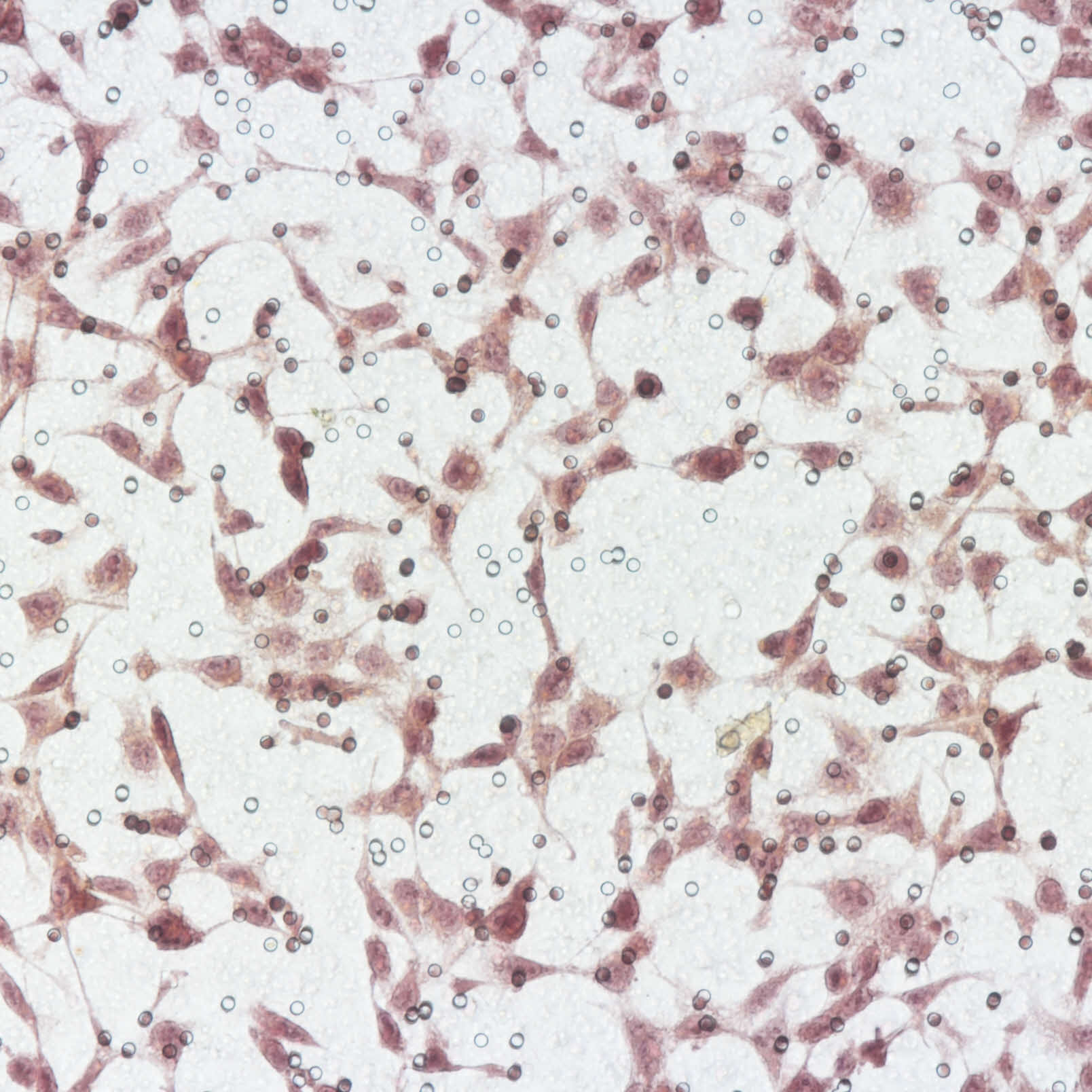

Supplement: Figure 2—source data 10. [file elife-97327-fig2-data10.zip › Source data 10/F2F-6-1-1-506-m.jpg]

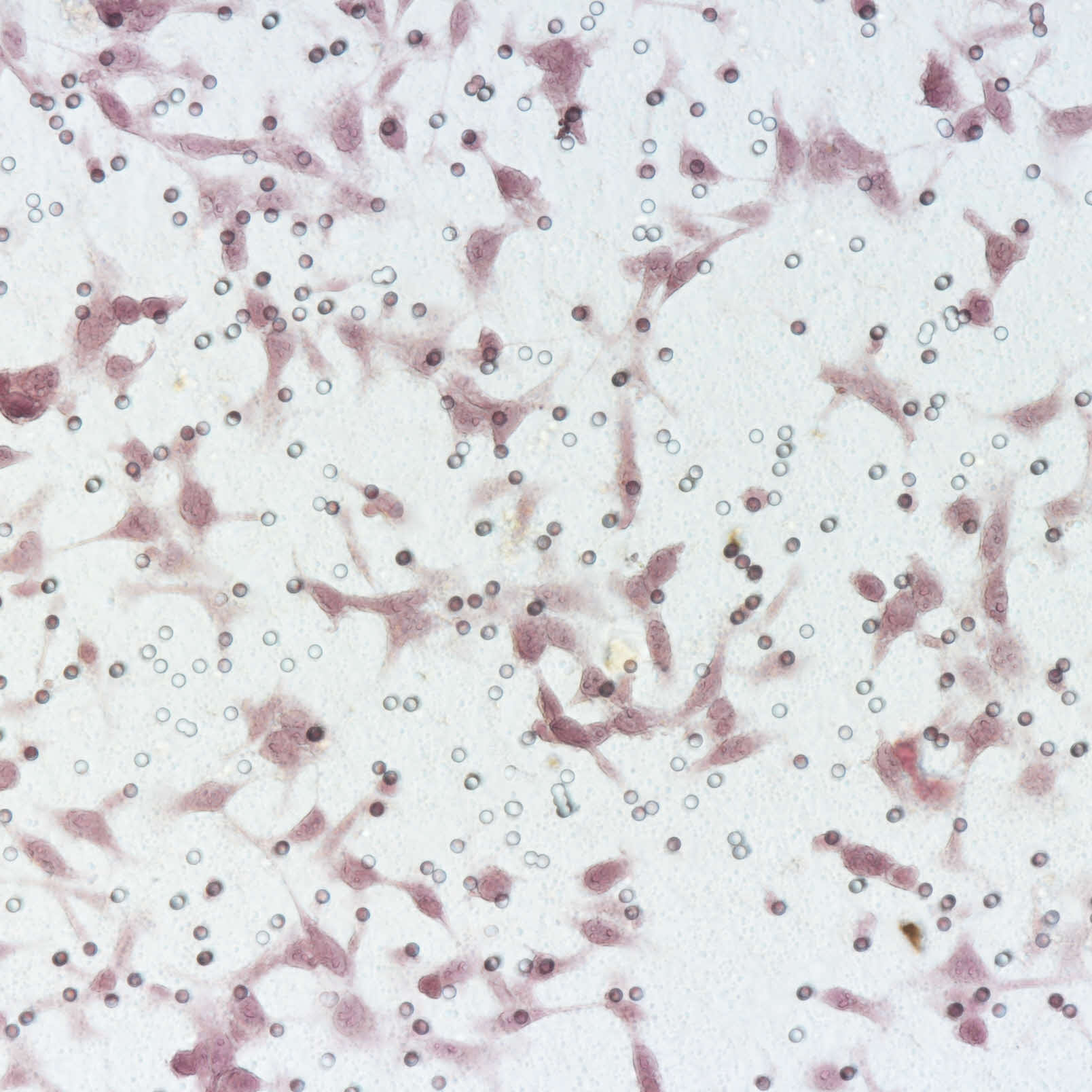

Supplement: Figure 2—source data 10. [file elife-97327-fig2-data10.zip › Source data 10/F2F-6-2-4-161-m.jpg]

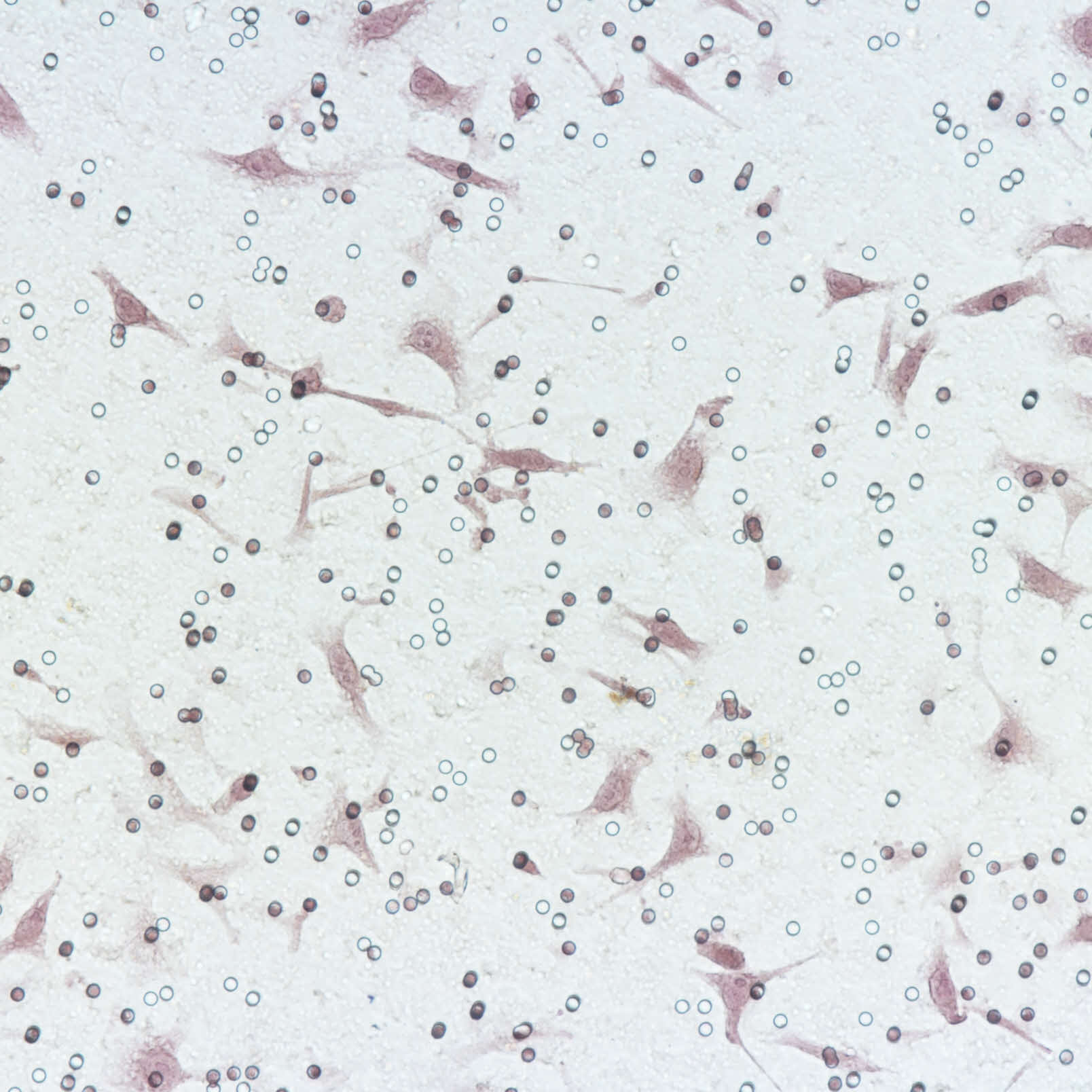

Supplement: Figure 2—source data 10. [file elife-97327-fig2-data10.zip › Source data 10/F2F-6-3-1-nc.jpg]

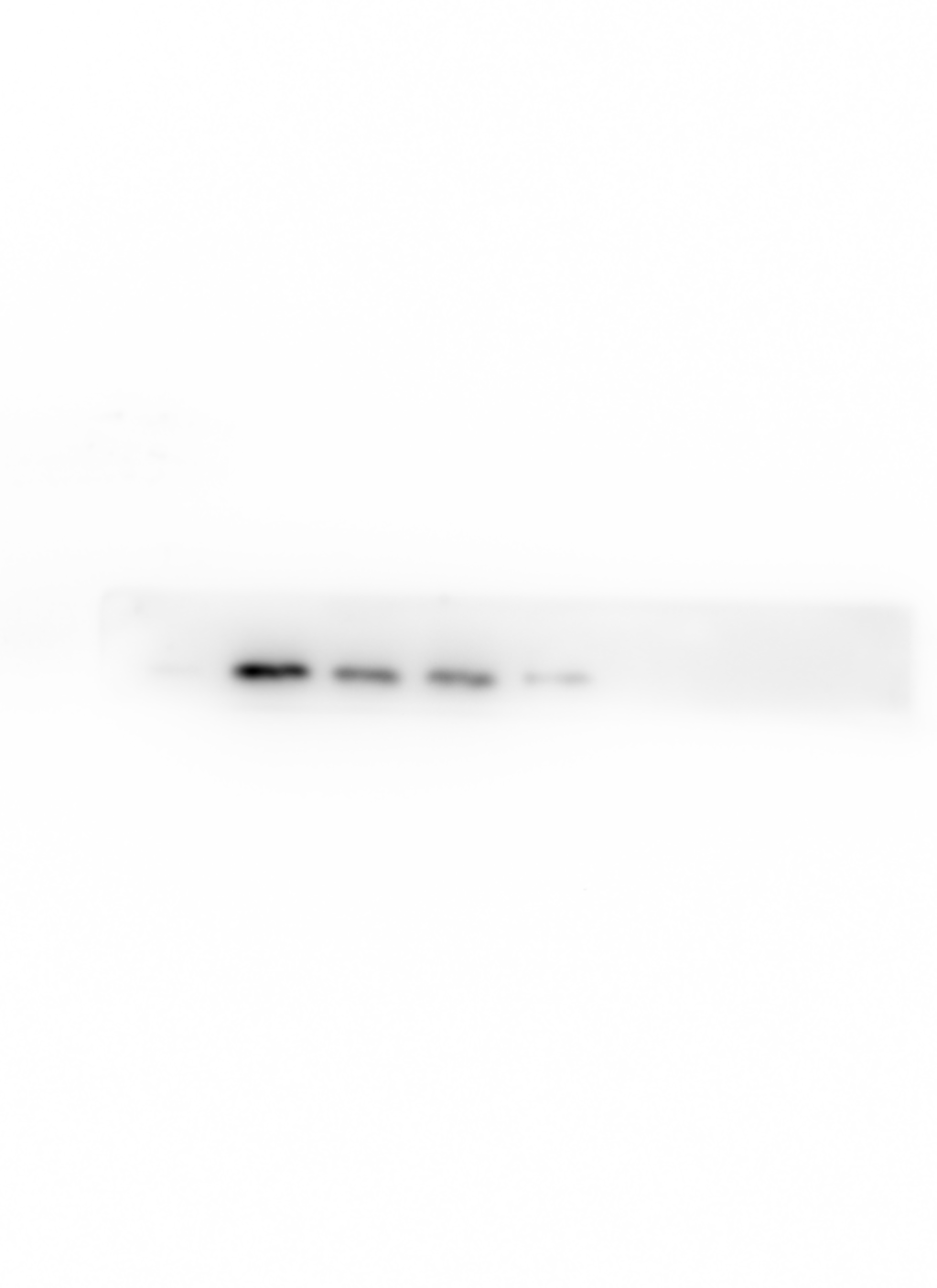

Supplement: Figure 3—source data 1. [file elife-97327-fig3-data1.zip › Figure 3-Source data 1/E-cadherin.tif]

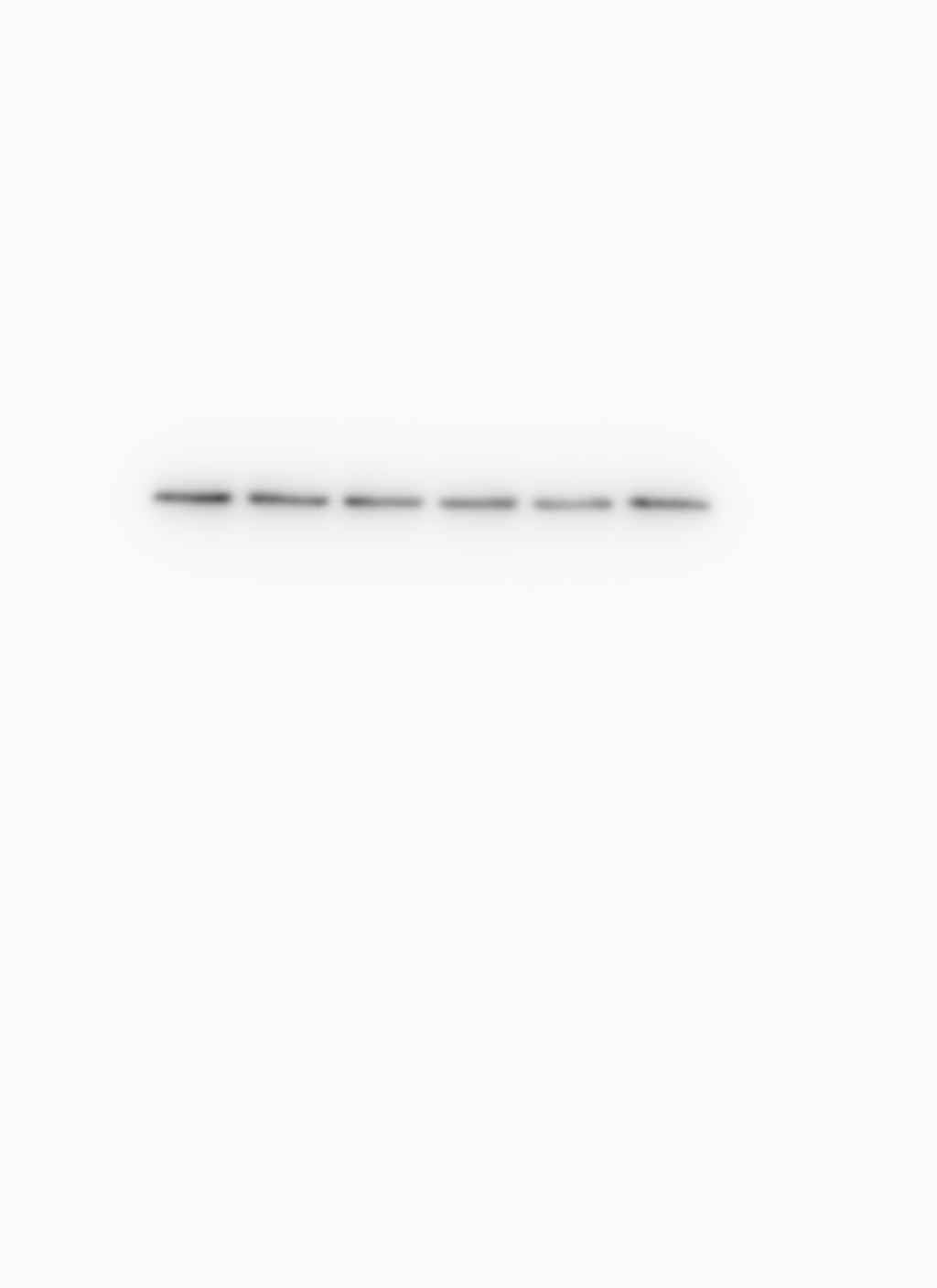

Supplement: Figure 3—source data 1. [file elife-97327-fig3-data1.zip › Figure 3-Source data 1/F3F-b-tublin-3 .tif]

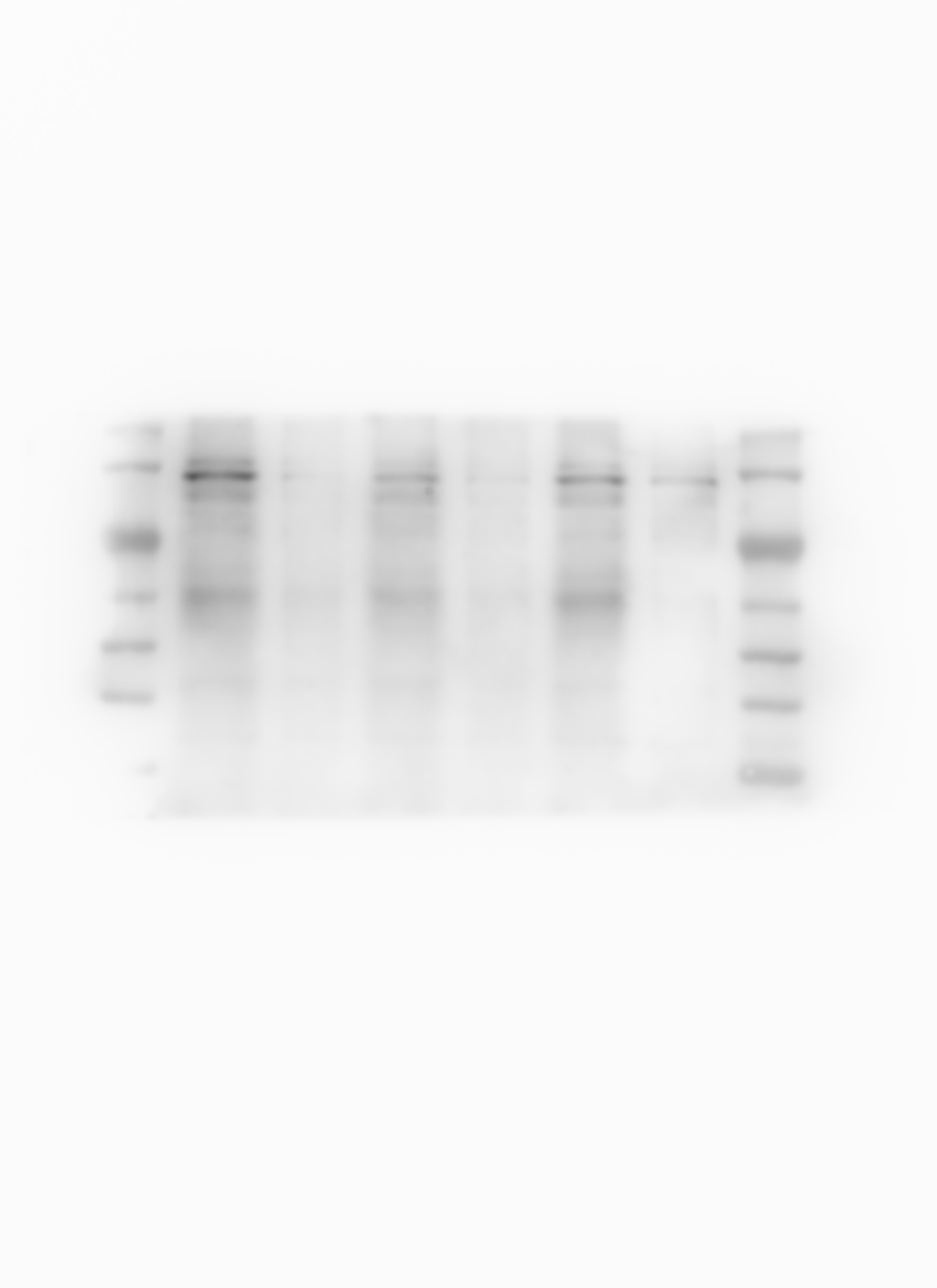

Supplement: Figure 3—source data 1. [file elife-97327-fig3-data1.zip › Figure 3-Source data 1/F3F-Eca-1.tif]

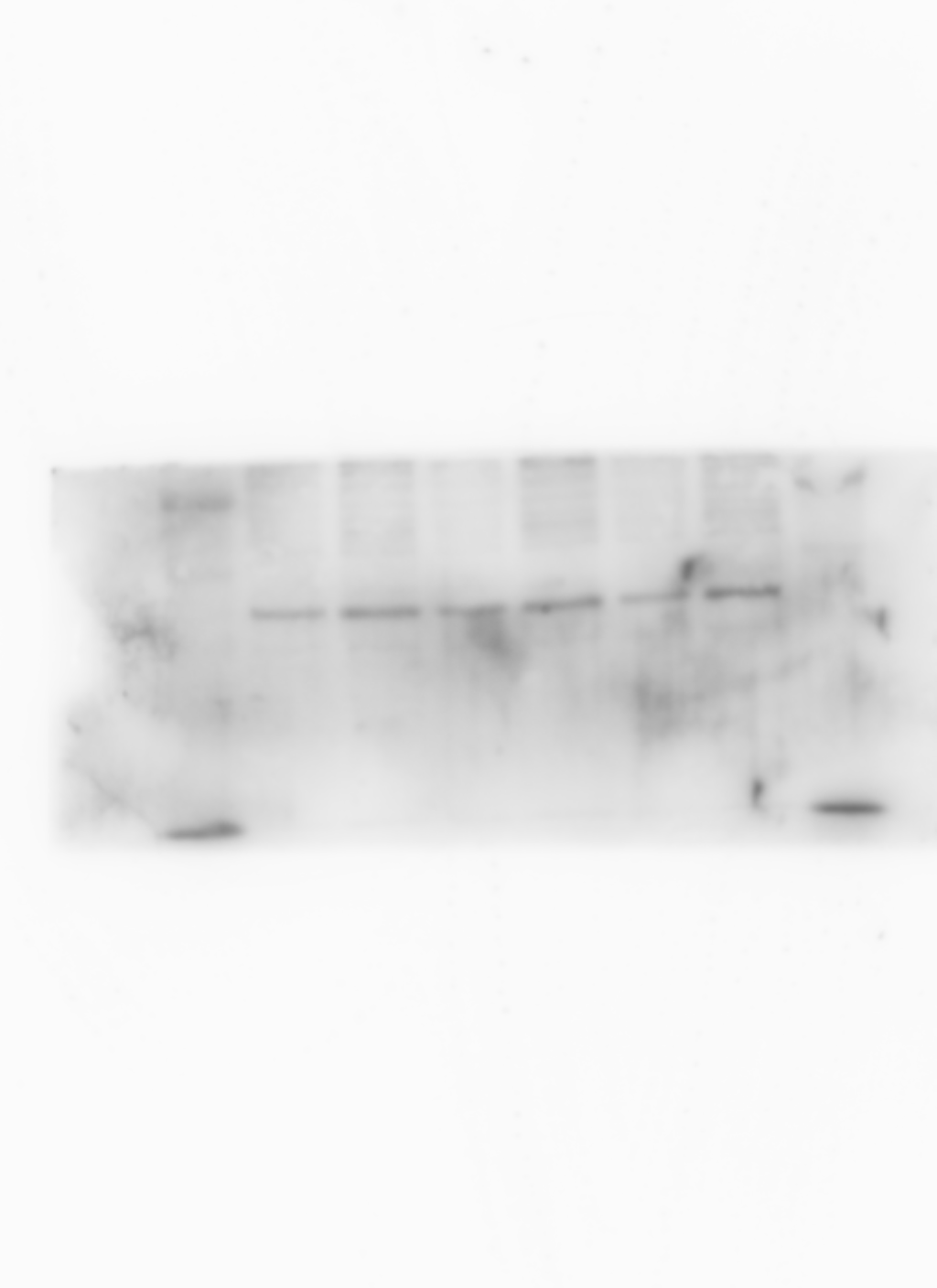

Supplement: Figure 3—source data 1. [file elife-97327-fig3-data1.zip › Figure 3-Source data 1/F3F-LCN2.tif]

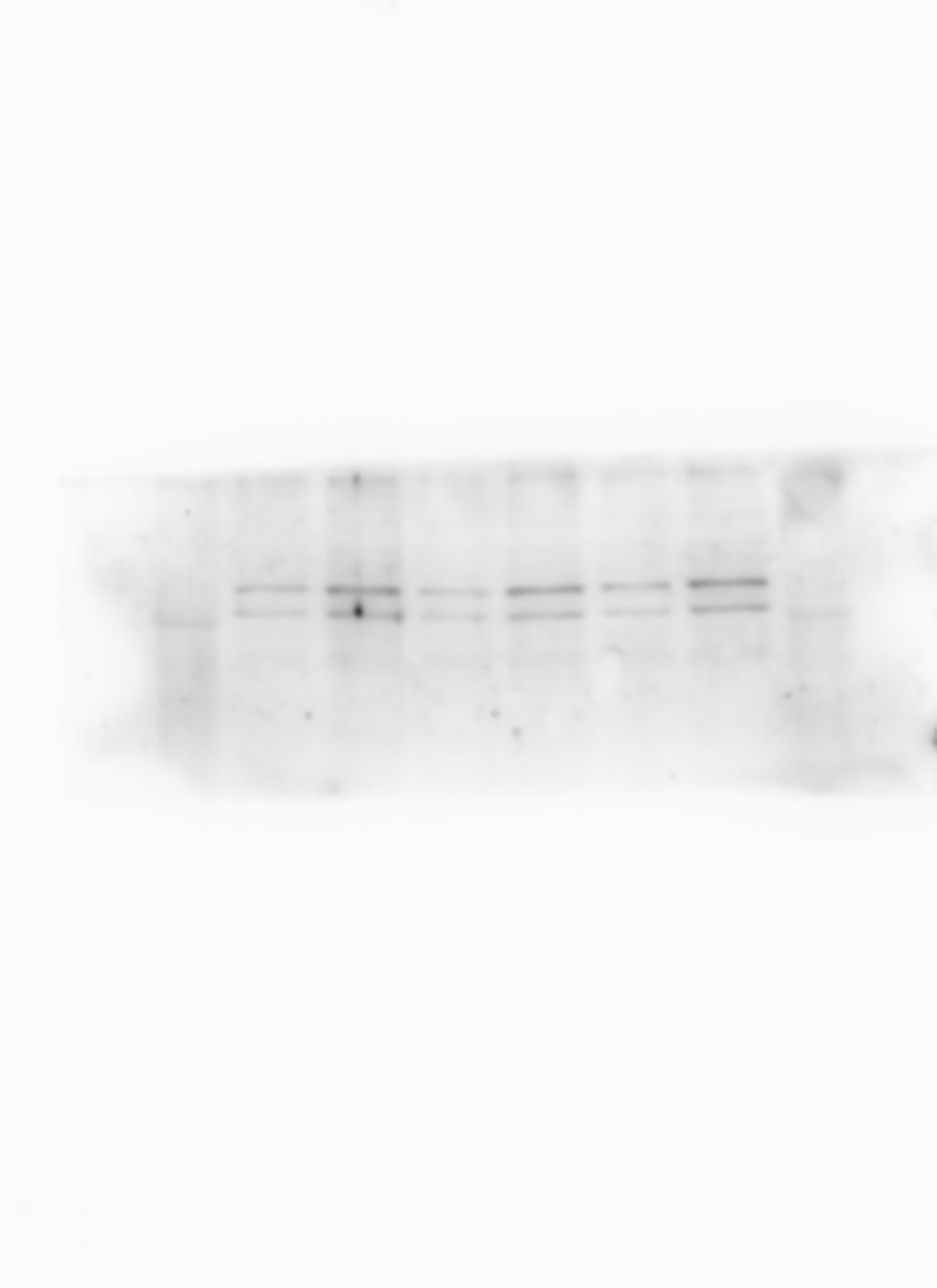

Supplement: Figure 3—source data 1. [file elife-97327-fig3-data1.zip › Figure 3-Source data 1/F3F-Vimentin .tif]

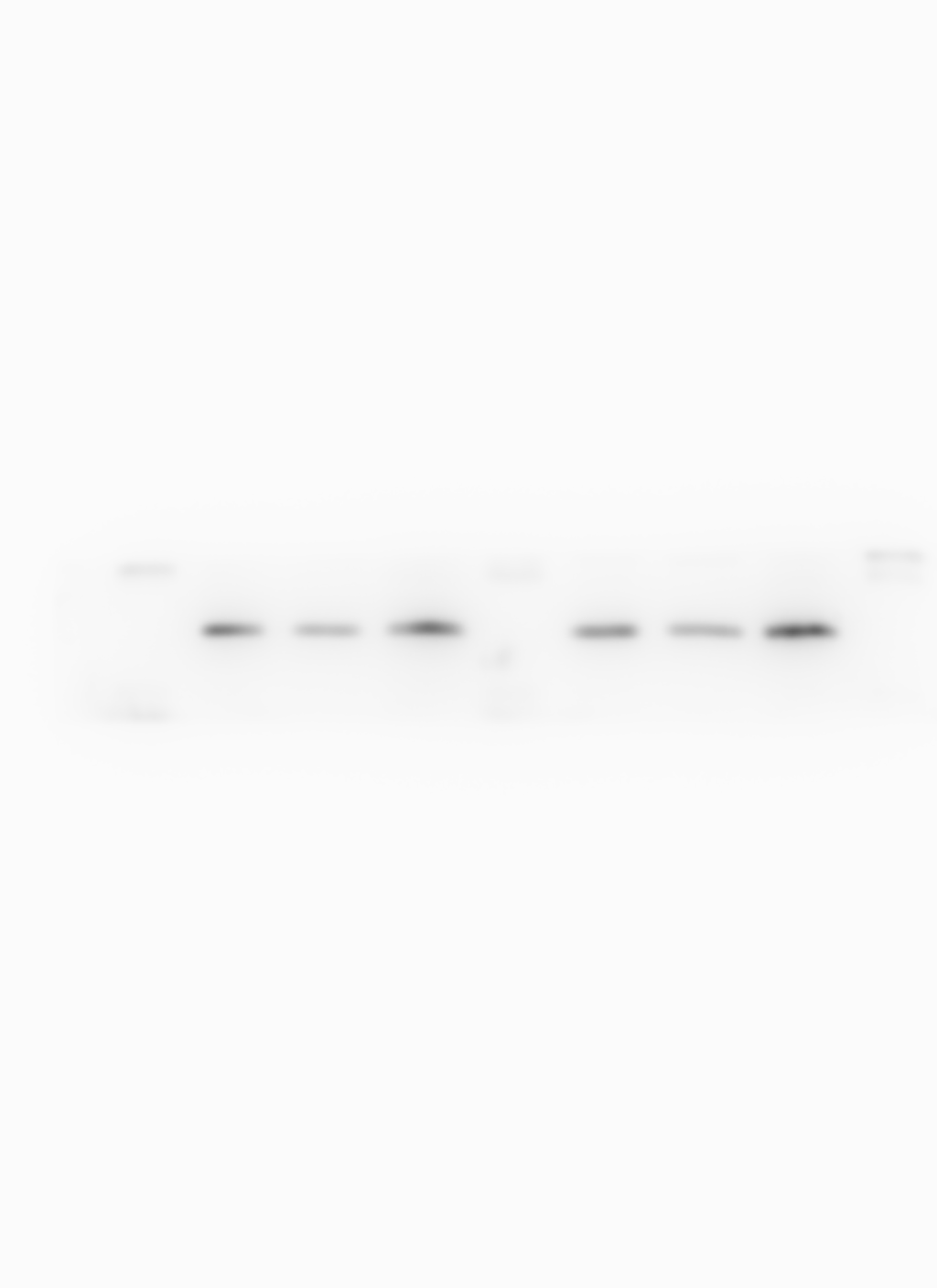

Supplement: Figure 3—source data 1. [file elife-97327-fig3-data1.zip › Figure 3-Source data 1/LCN2.tif]

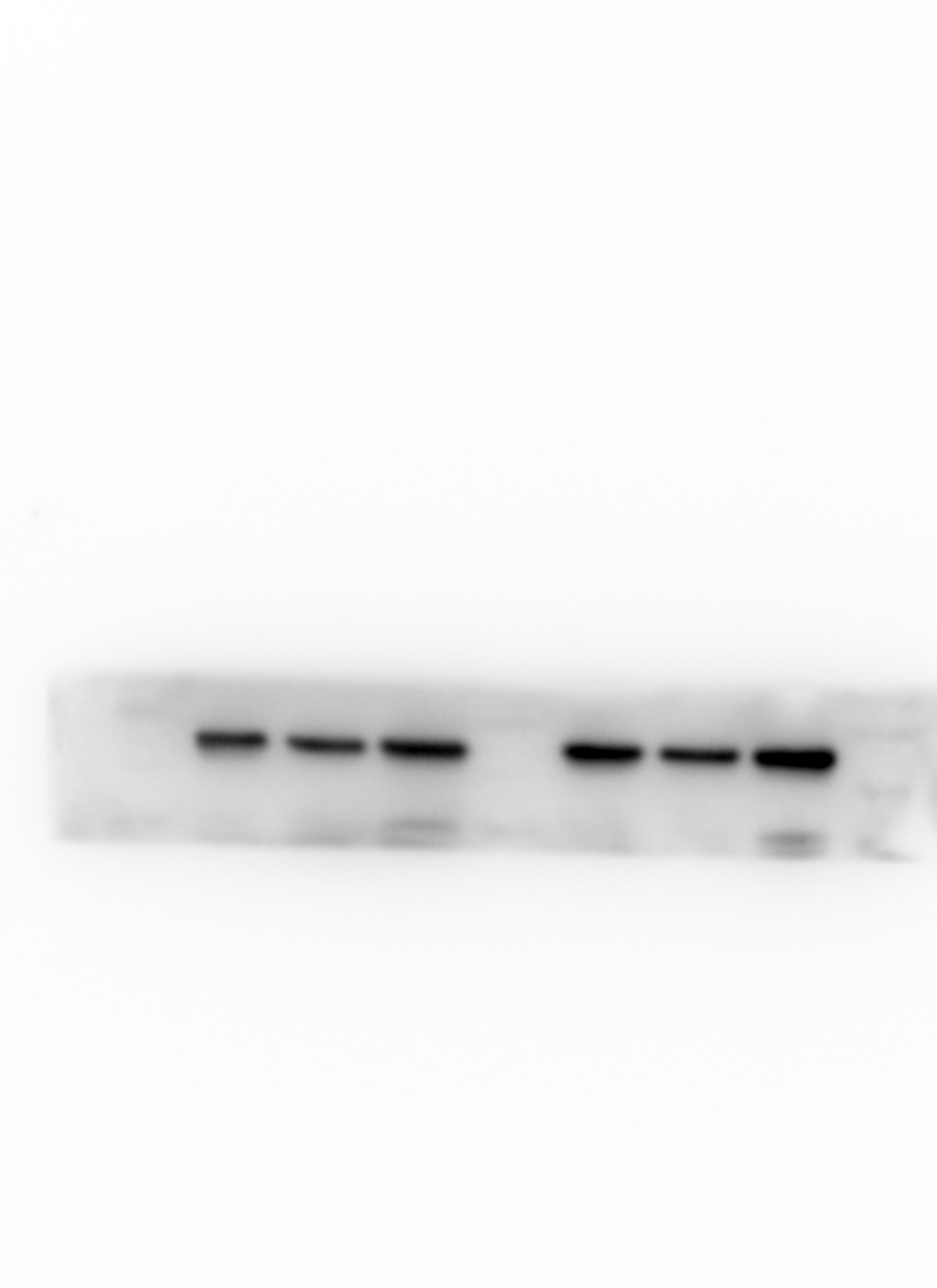

Supplement: Figure 3—source data 1. [file elife-97327-fig3-data1.zip › Figure 3-Source data 1/tubulin.tif]

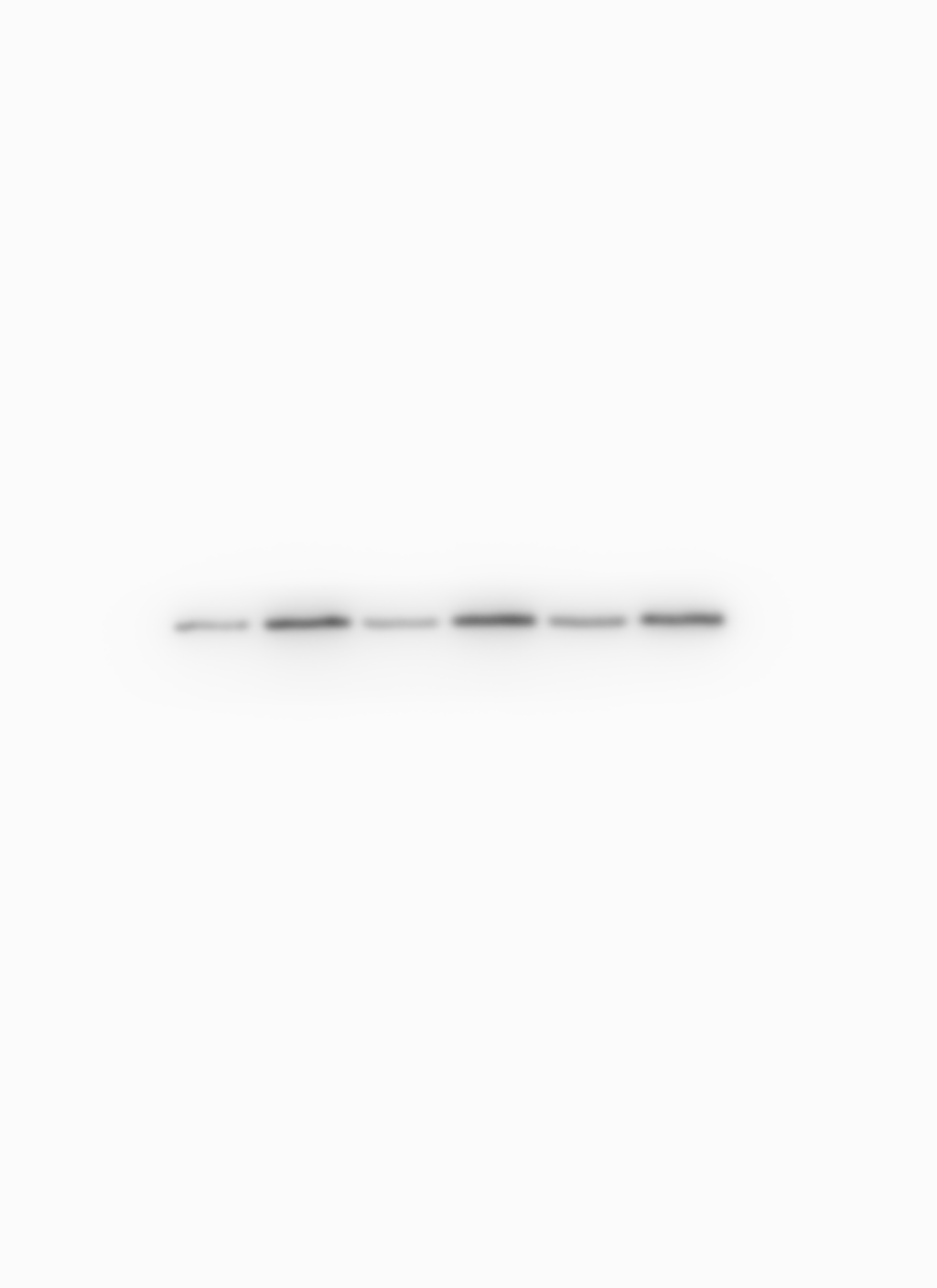

Supplement: Figure 3—source data 1. [file elife-97327-fig3-data1.zip › Figure 3-Source data 1/vimentin.tif]

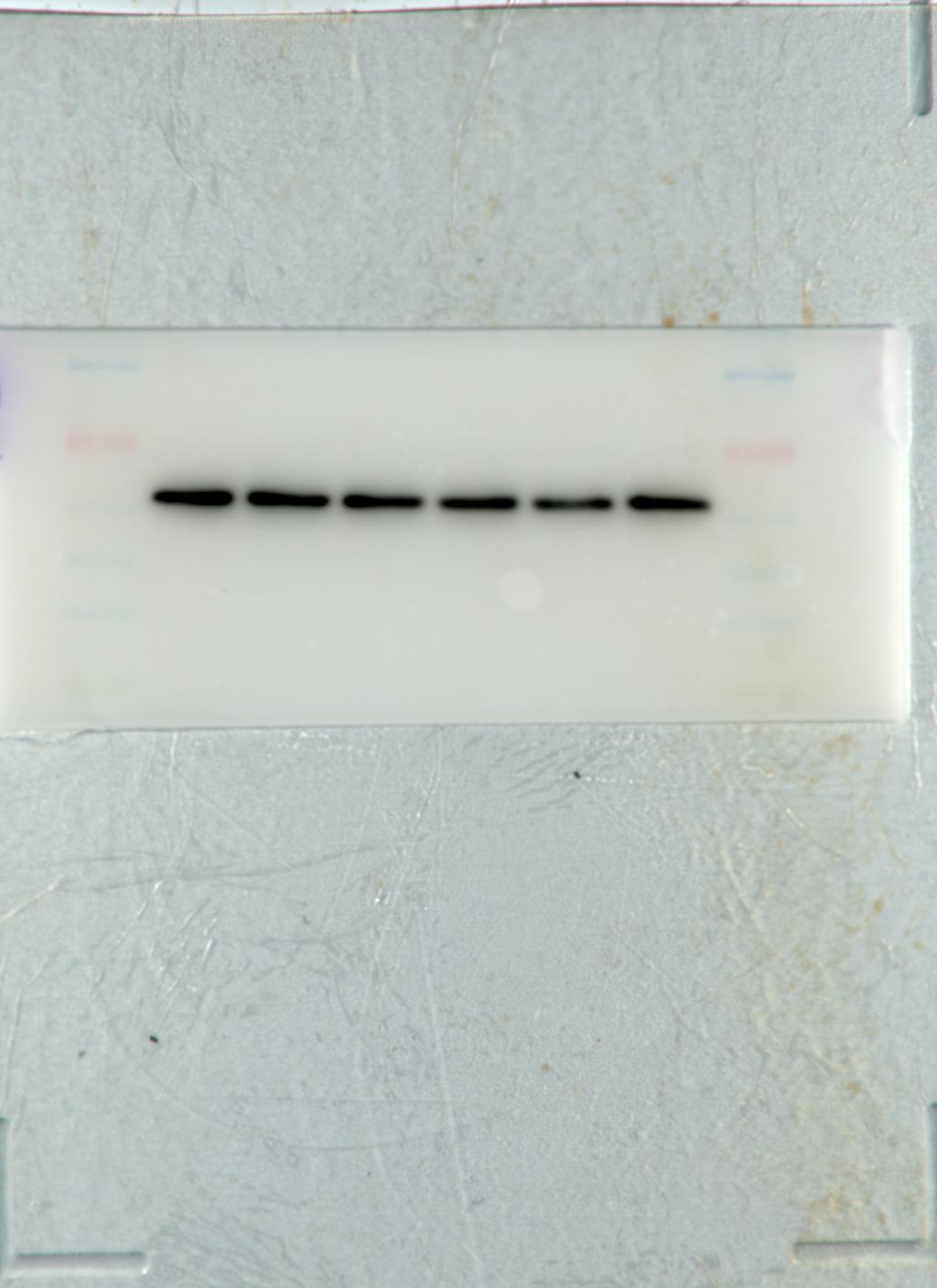

Supplement: Figure 3—source data 2. [file elife-97327-fig3-data2.zip › Figure 3-Source data 2/F3F-b-tublin-3 +Marker.jpg]

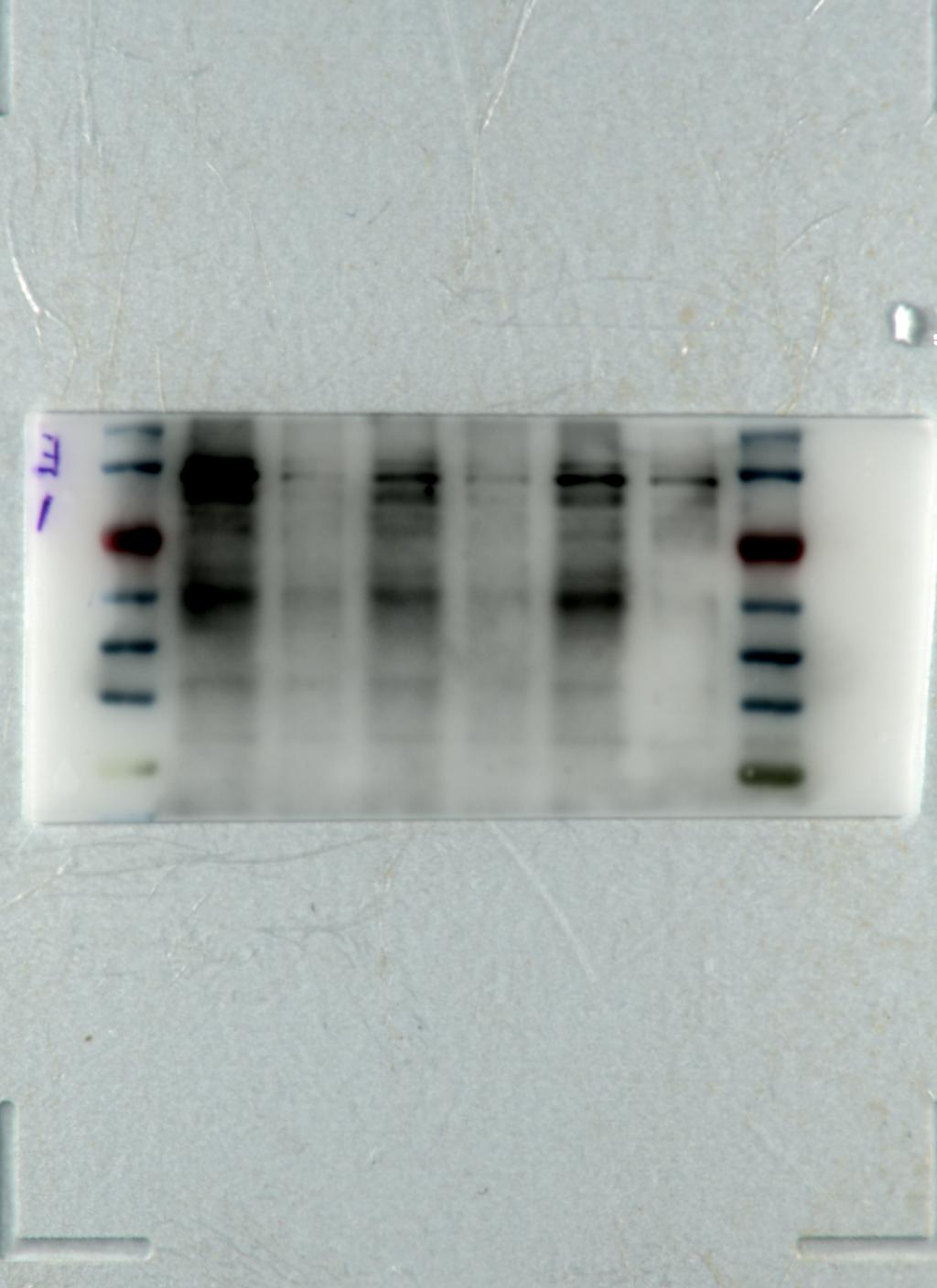

Supplement: Figure 3—source data 2. [file elife-97327-fig3-data2.zip › Figure 3-Source data 2/F3F-Eca-1+Marker.jpg]

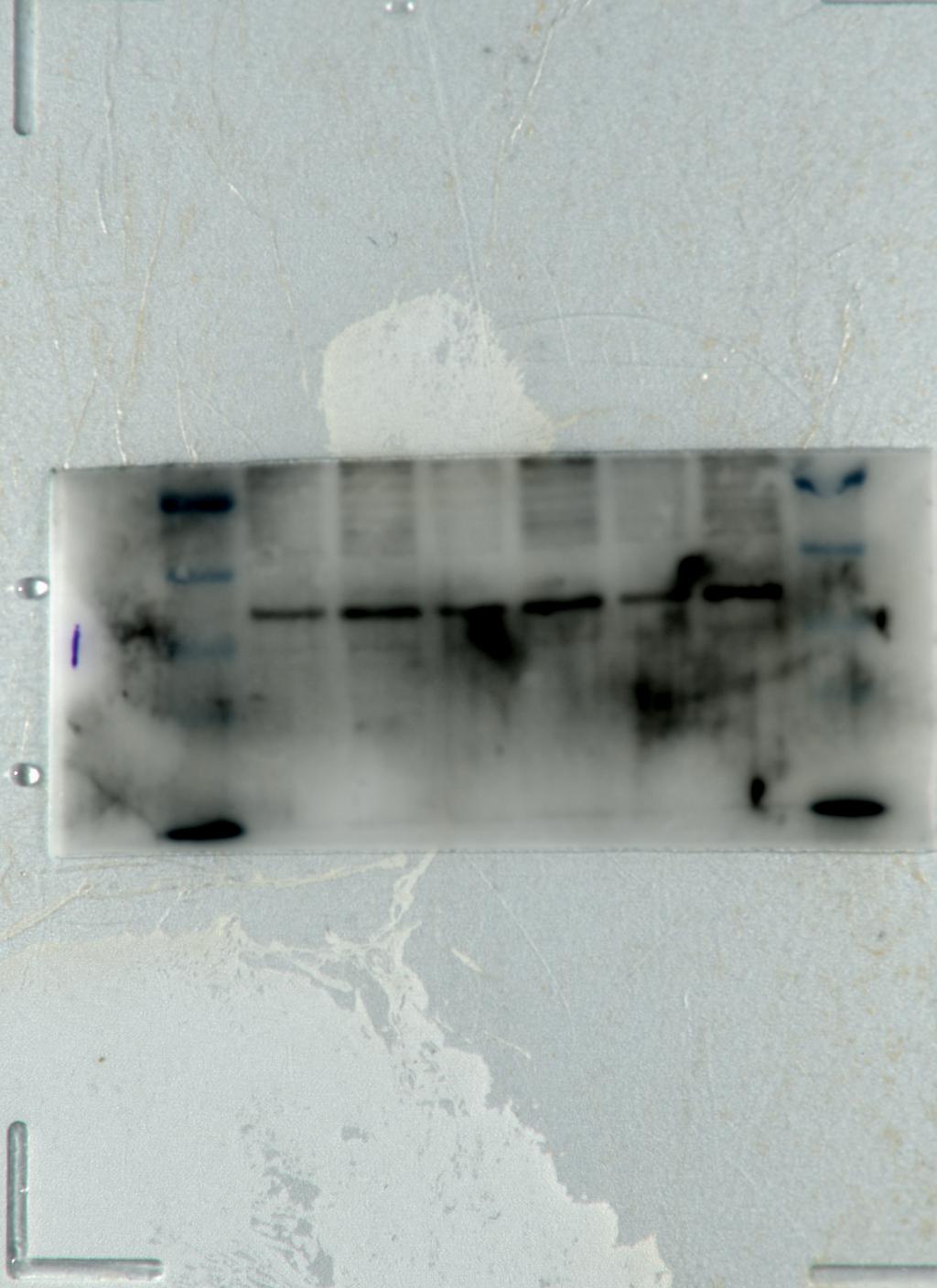

Supplement: Figure 3—source data 2. [file elife-97327-fig3-data2.zip › Figure 3-Source data 2/F3F-LCN2+Marker.jpg]

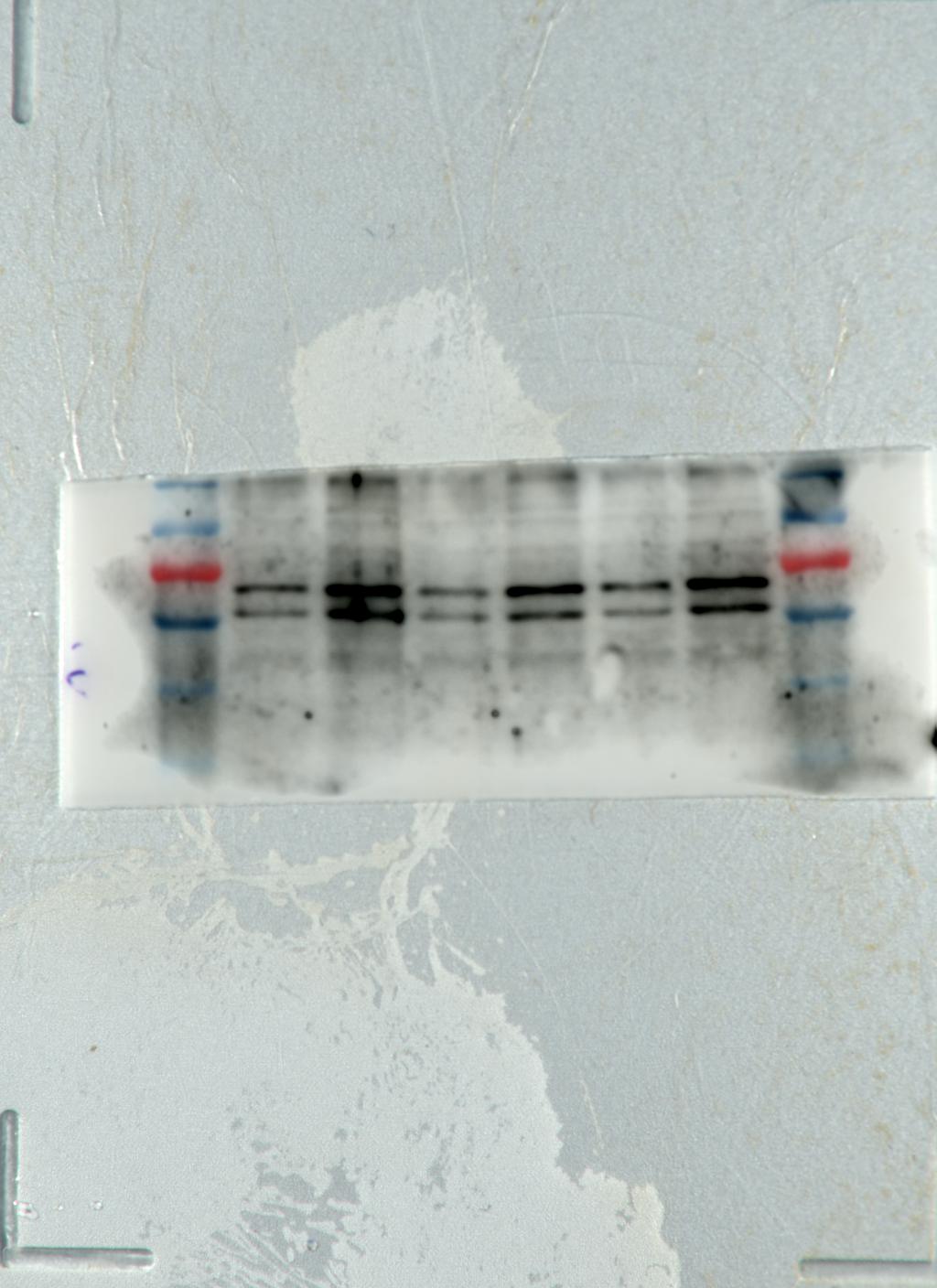

Supplement: Figure 3—source data 2. [file elife-97327-fig3-data2.zip › Figure 3-Source data 2/F3F-Vimentin+Marker.jpg]

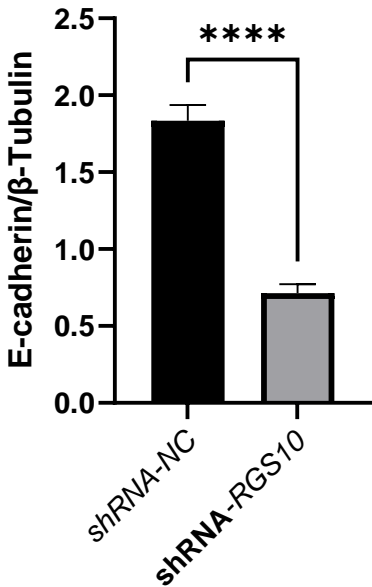

Supplement: Figure 3—source data 3. [file elife-97327-fig3-data3.pdf]

# EnhancedVolcano

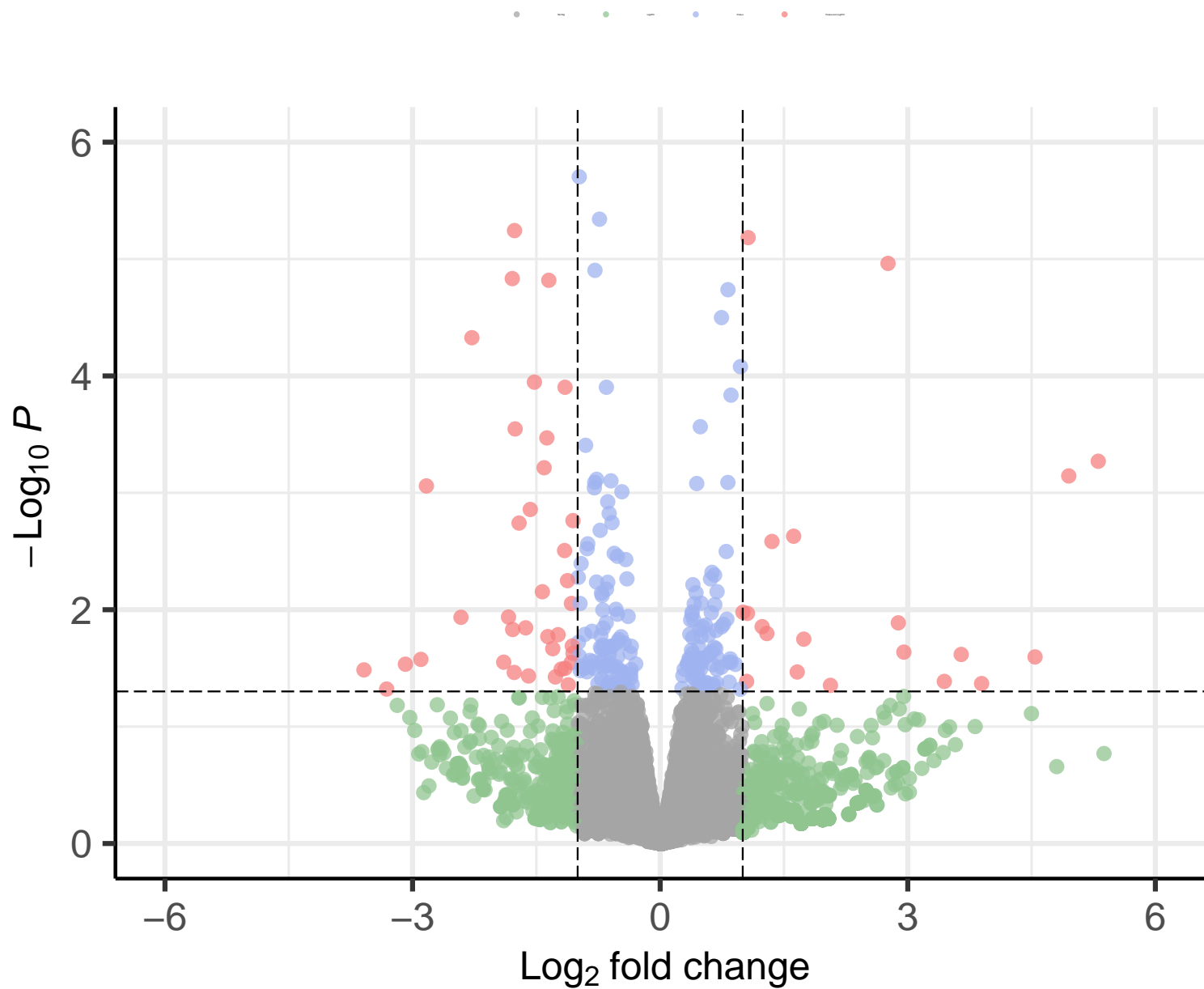

Supplement: Figure 3—source data 4. [file elife-97327-fig3-data4.zip › Figure 3-Source data 4/F3A.pdf]

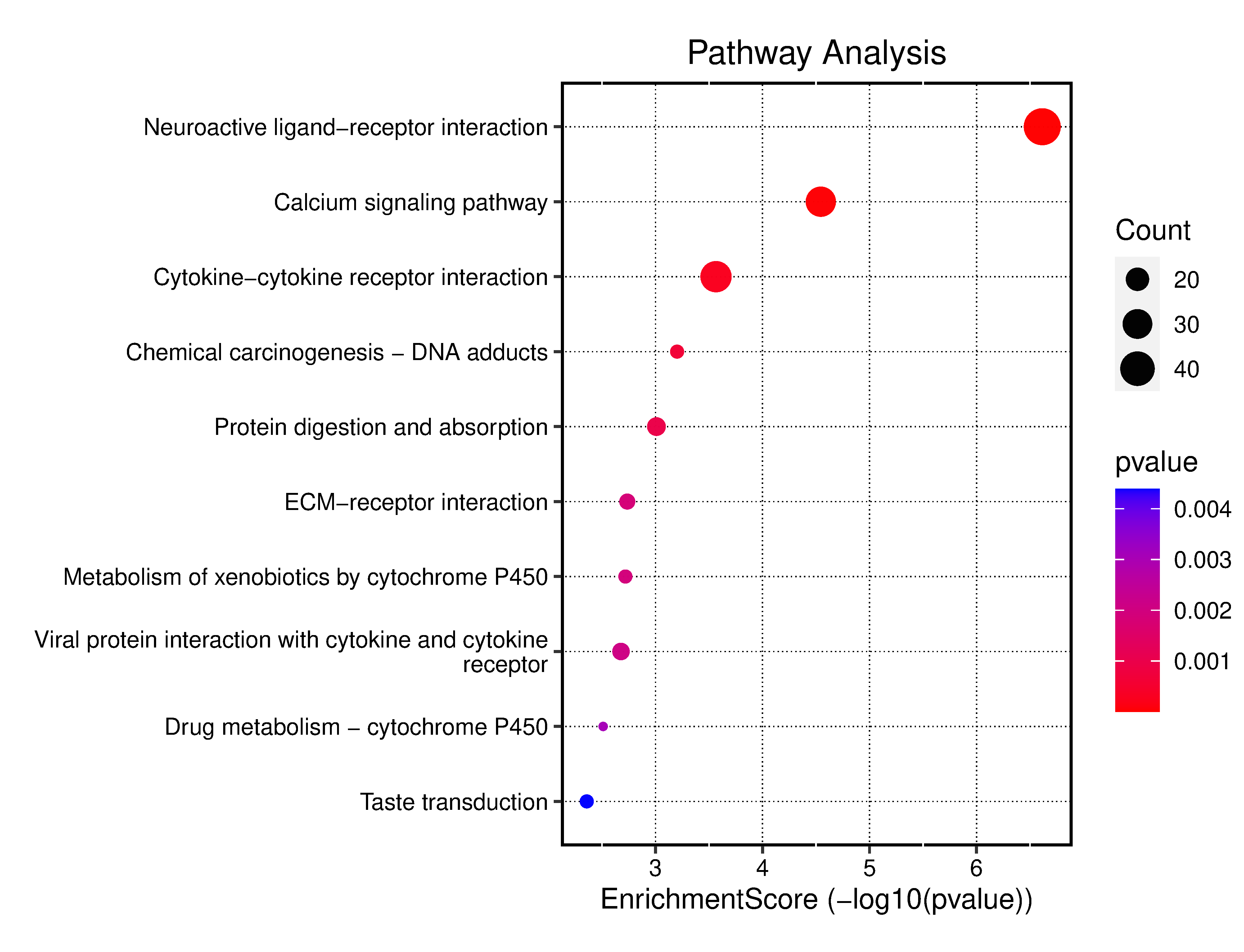

Supplement: Figure 3—source data 4. [file elife-97327-fig3-data4.zip › Figure 3-Source data 4/F3B Pathway_Enrichment_Score_dotplot.tiff]

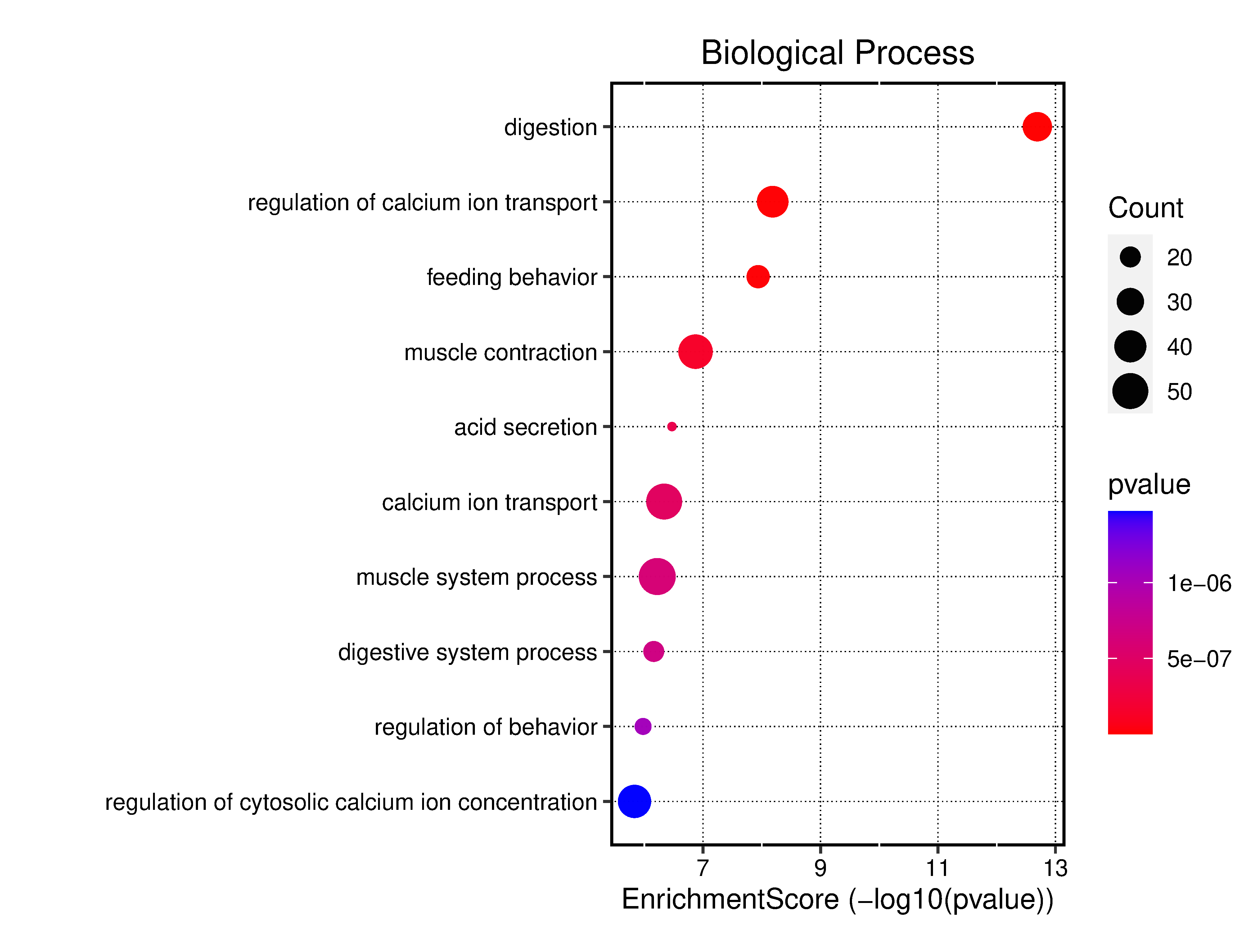

Supplement: Figure 3—source data 4. [file elife-97327-fig3-data4.zip › Figure 3-Source data 4/F3C-BP_Enrichment_Score_dotplot.tiff]

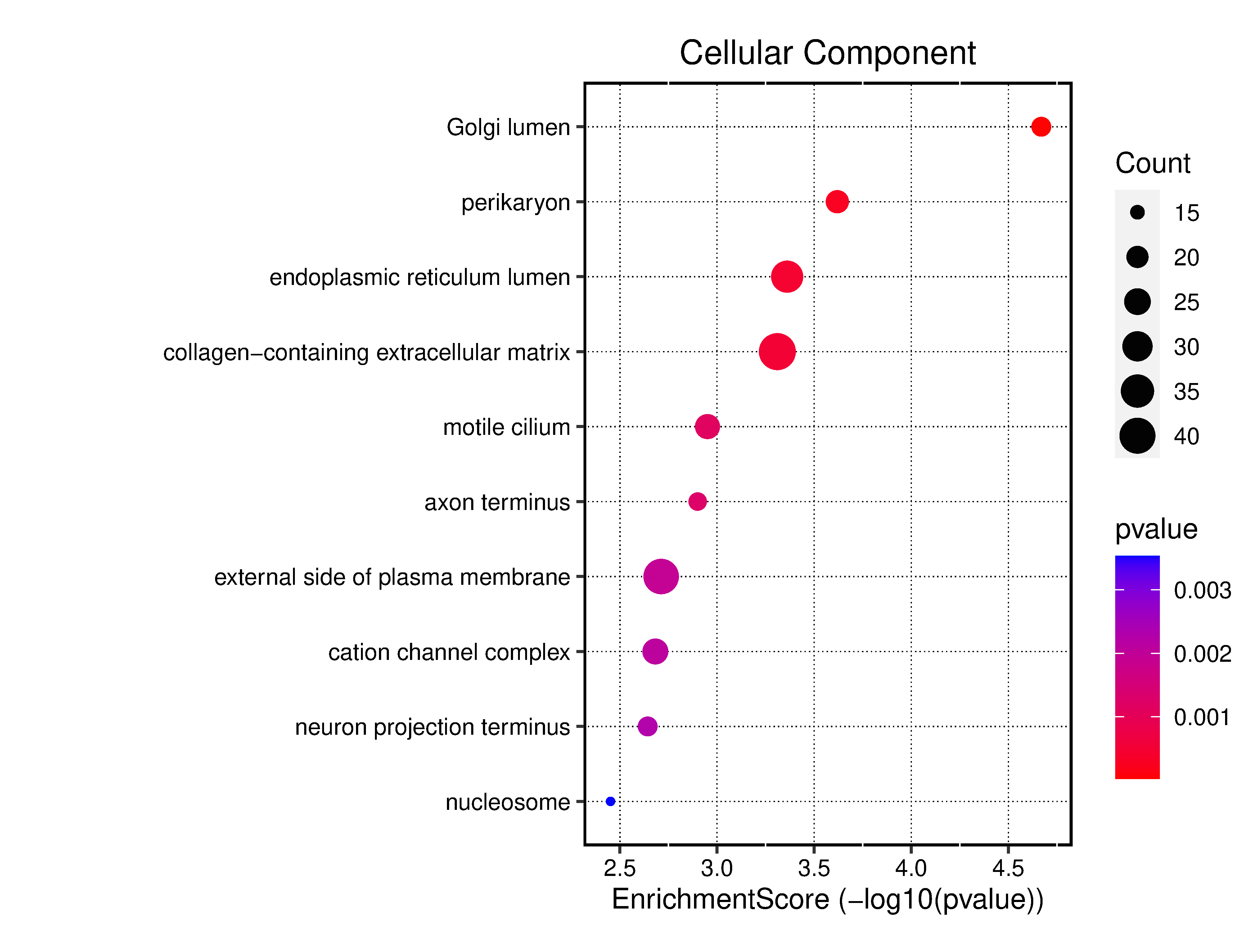

Supplement: Figure 3—source data 4. [file elife-97327-fig3-data4.zip › Figure 3-Source data 4/F3D-CC_Enrichment_Score_dotplot.tiff]

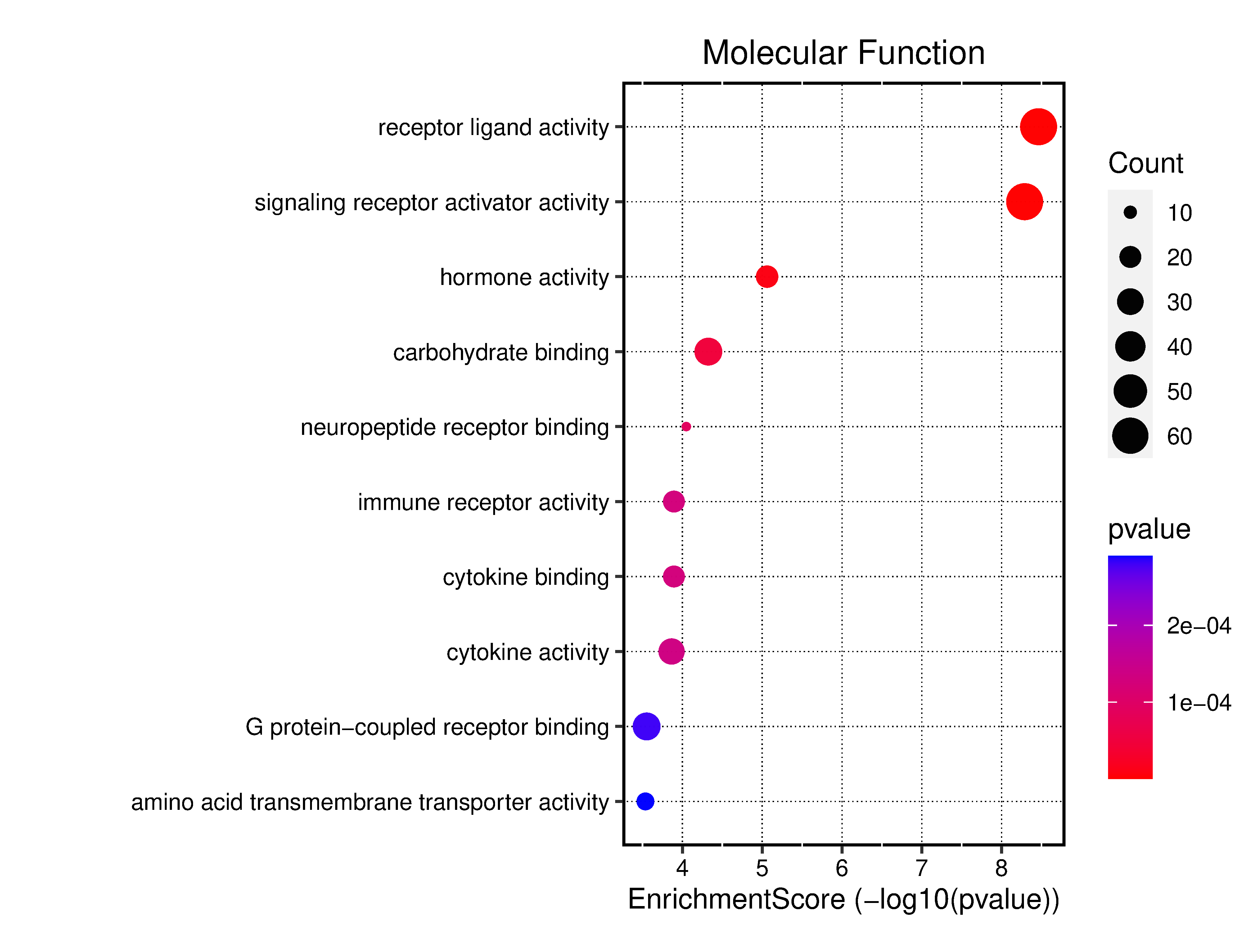

Supplement: Figure 3—source data 4. [file elife-97327-fig3-data4.zip › Figure 3-Source data 4/F3E-MF_Enrichment_Score_dotplot.tiff]

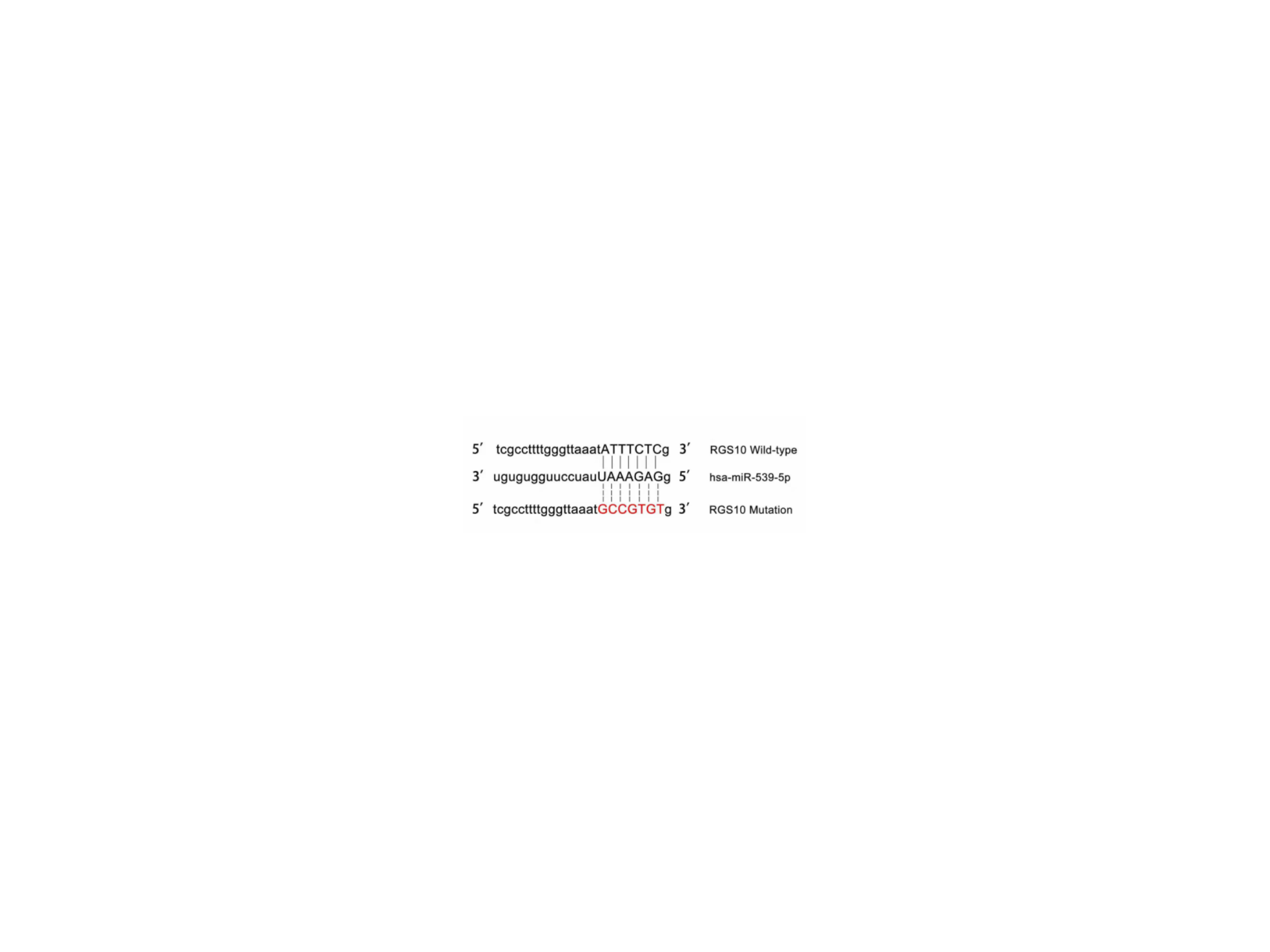

Supplement: Figure 3—source data 4. [file elife-97327-fig3-data4.zip › Figure 3-Source data 4/F3G.tif]

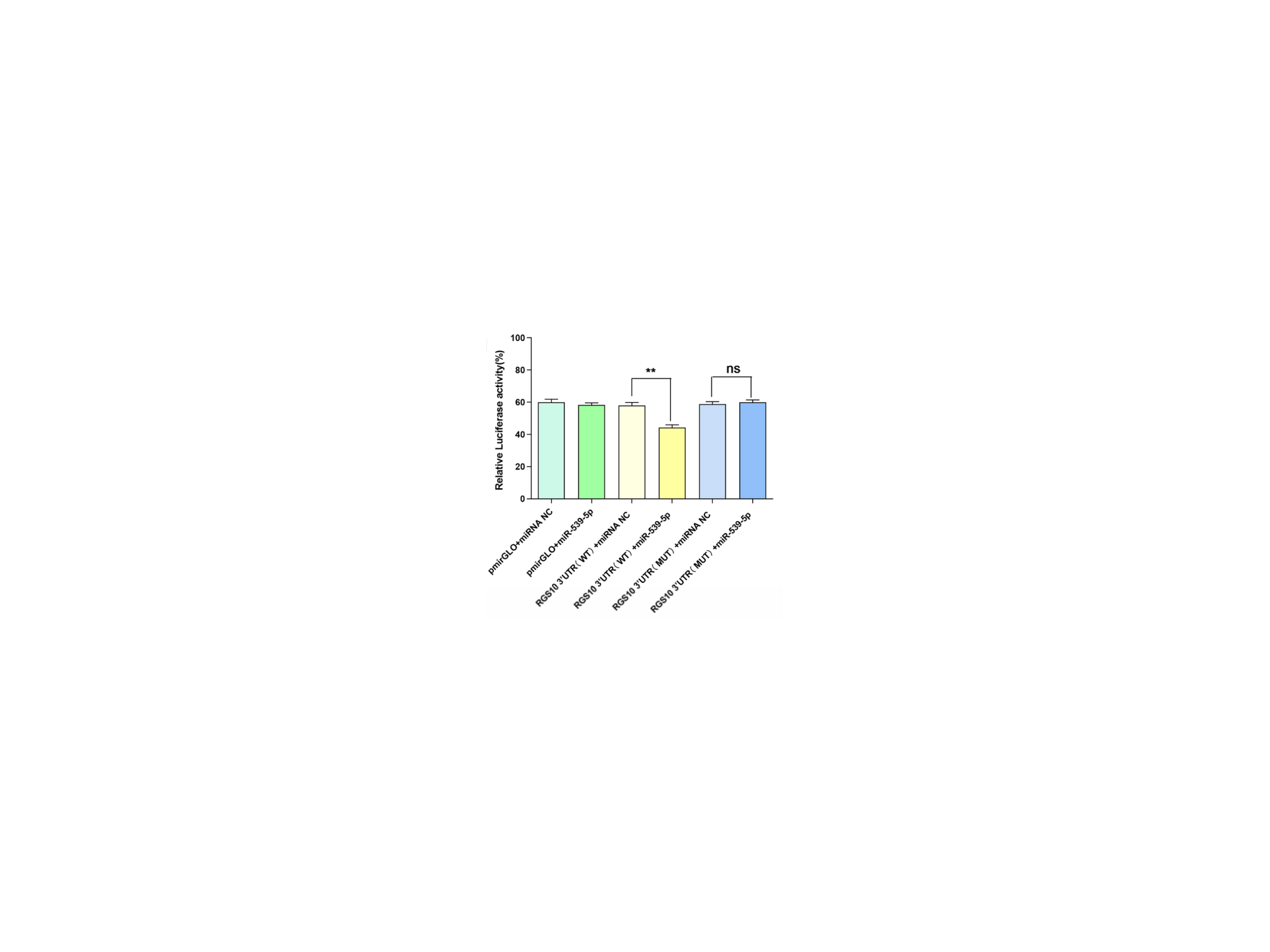

Supplement: Figure 3—source data 4. [file elife-97327-fig3-data4.zip › Figure 3-Source data 4/F3H.tif]

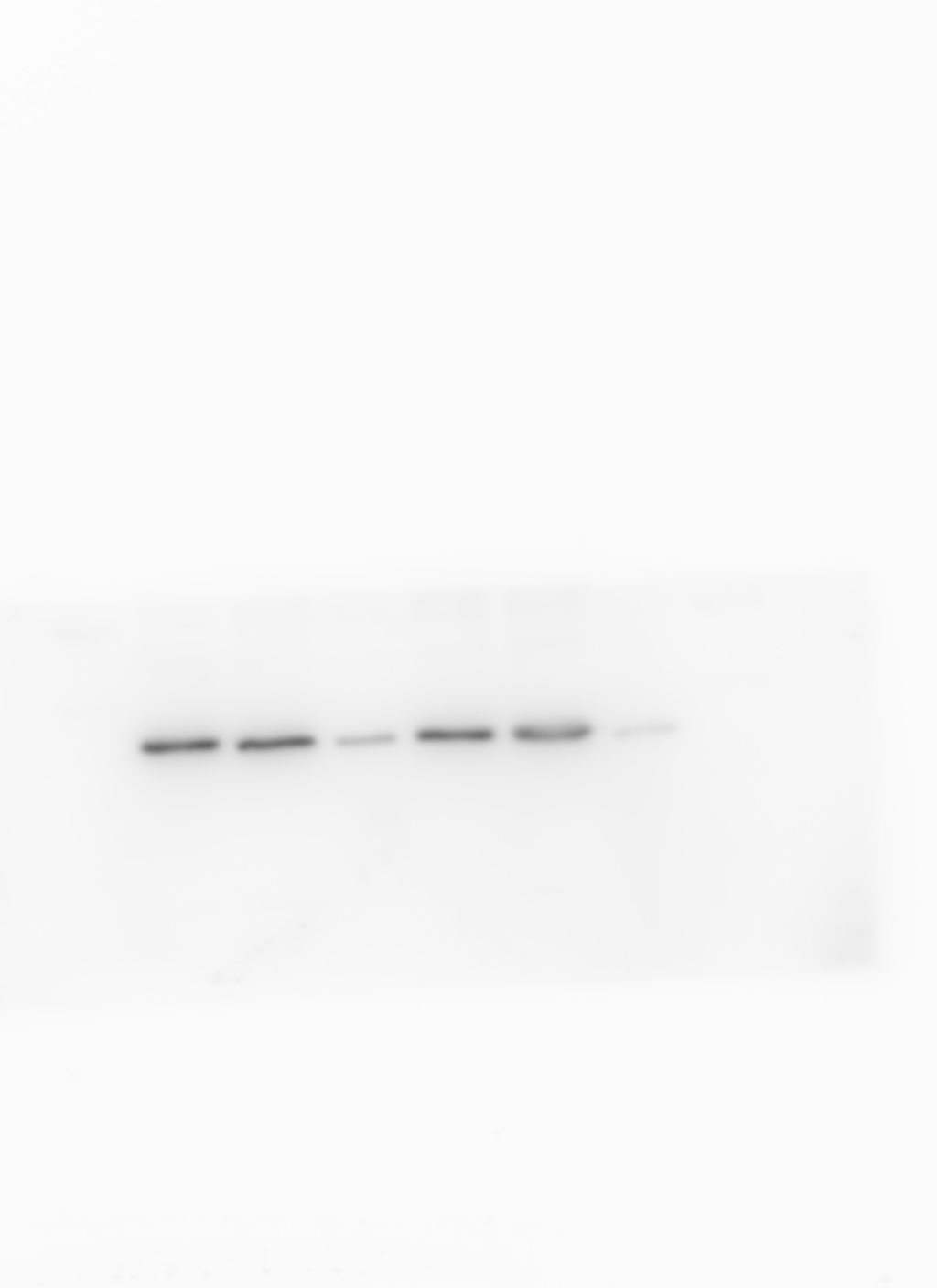

Supplement: Figure 4—source data 1. [file elife-97327-fig4-data1.zip › Figure4-Source data 1/F4C-RGS10-2 .tif]

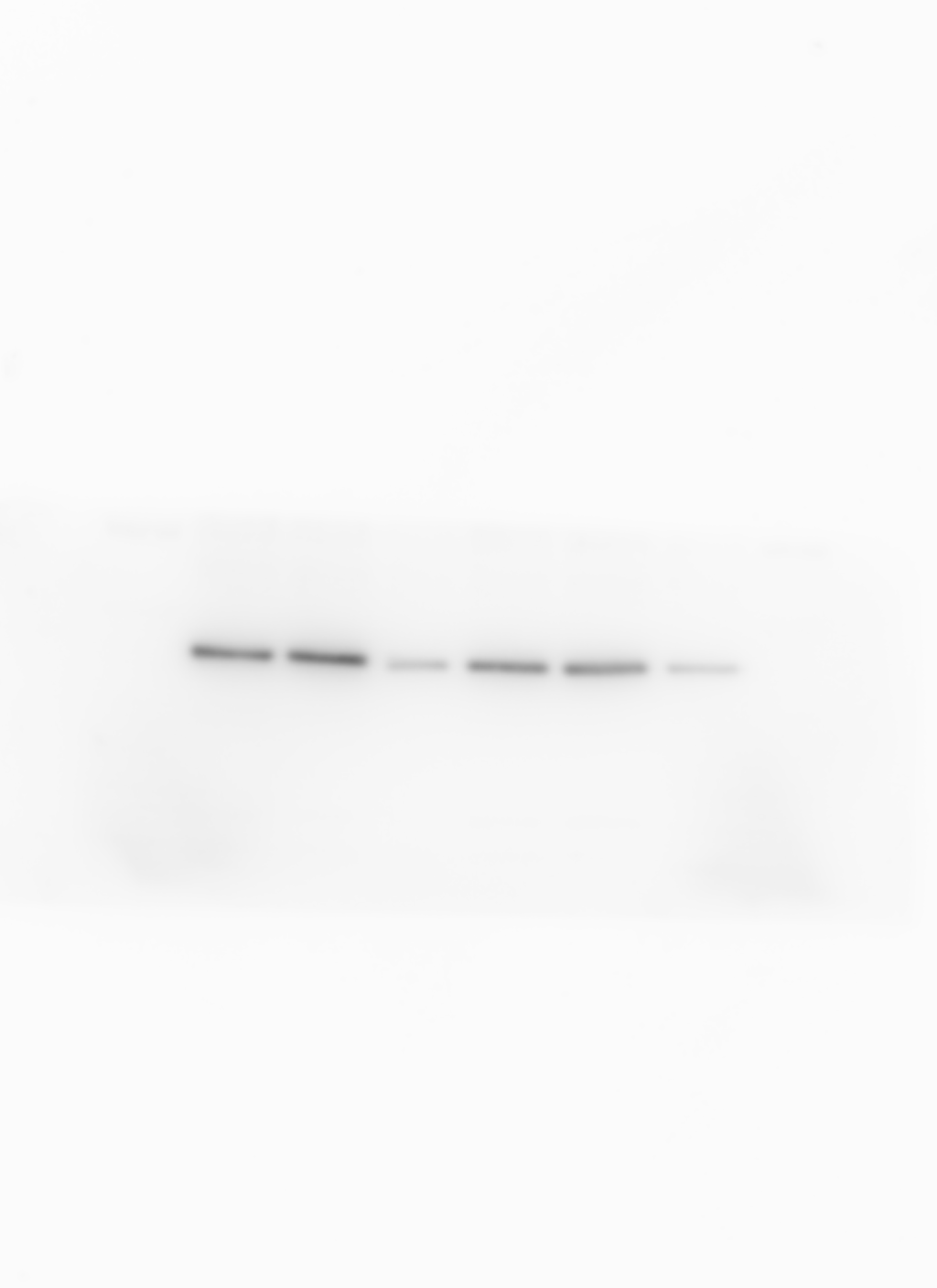

Supplement: Figure 4—source data 1. [file elife-97327-fig4-data1.zip › Figure4-Source data 1/F4C-RGS10-3.tif]

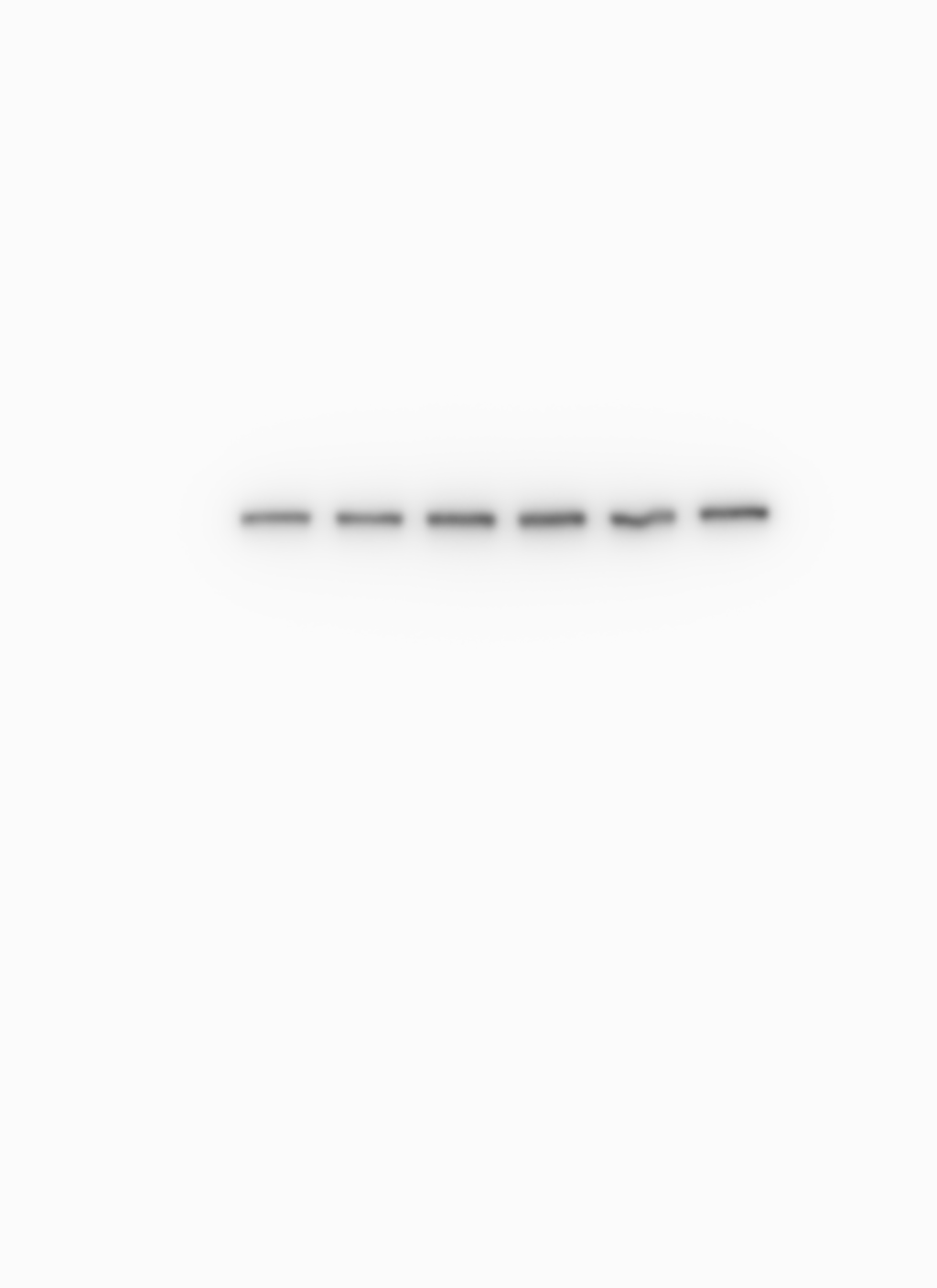

Supplement: Figure 4—source data 1. [file elife-97327-fig4-data1.zip › Figure4-Source data 1/F4C-TU .tif]

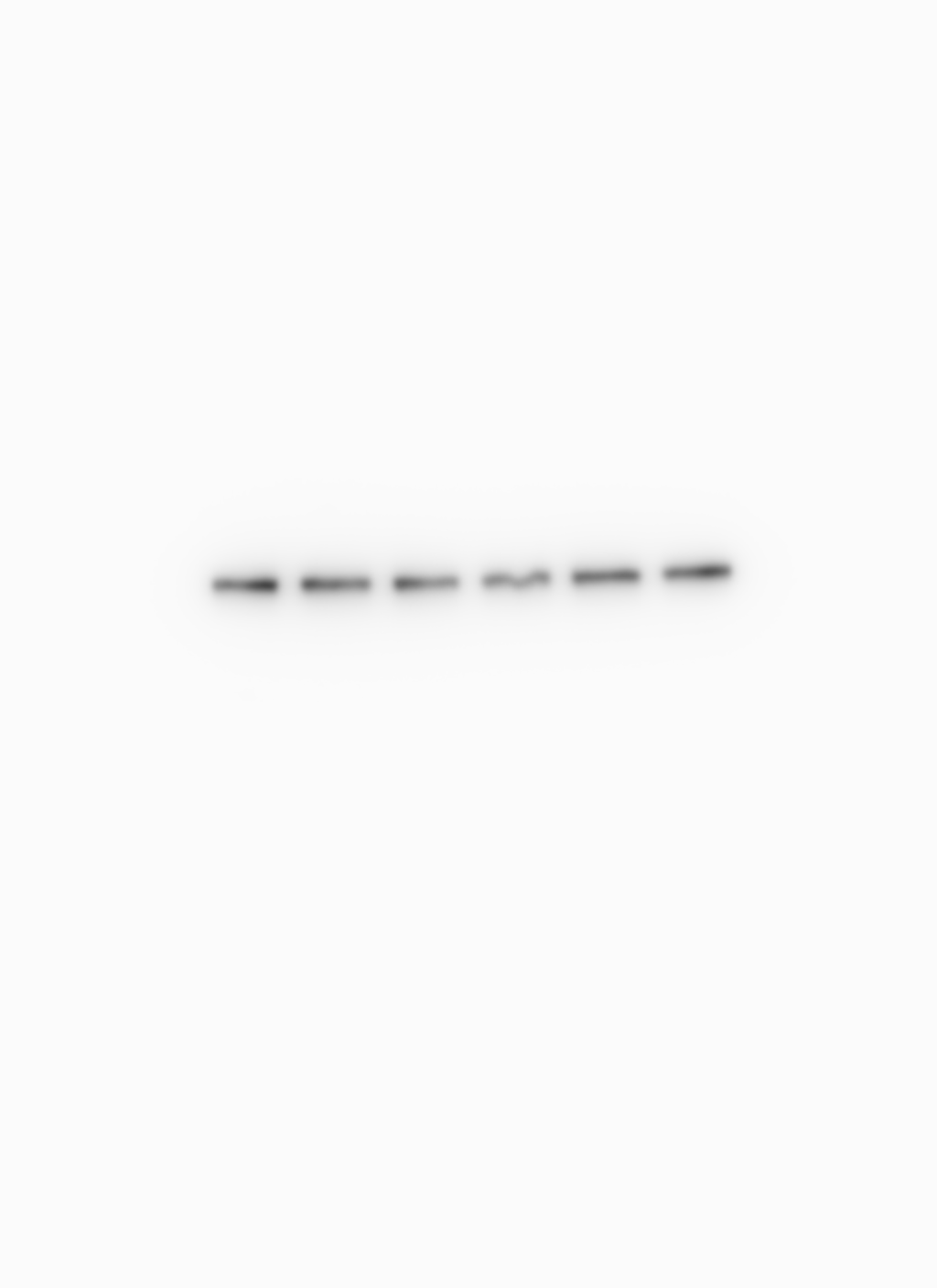

Supplement: Figure 4—source data 1. [file elife-97327-fig4-data1.zip › Figure4-Source data 1/F4C-TU-2 .tif]

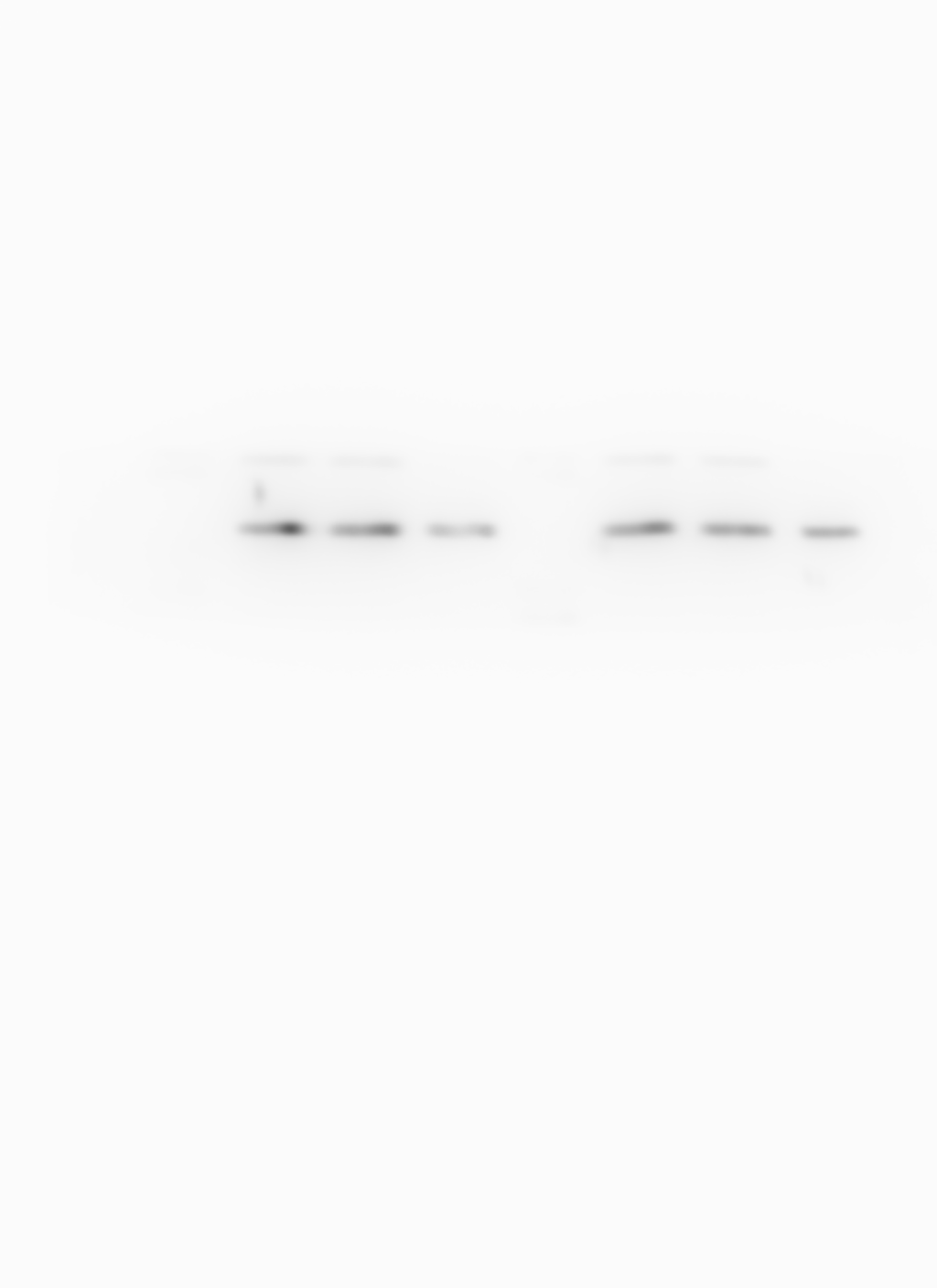

Supplement: Figure 4—source data 1. [file elife-97327-fig4-data1.zip › Figure4-Source data 1/RGS10.tif]

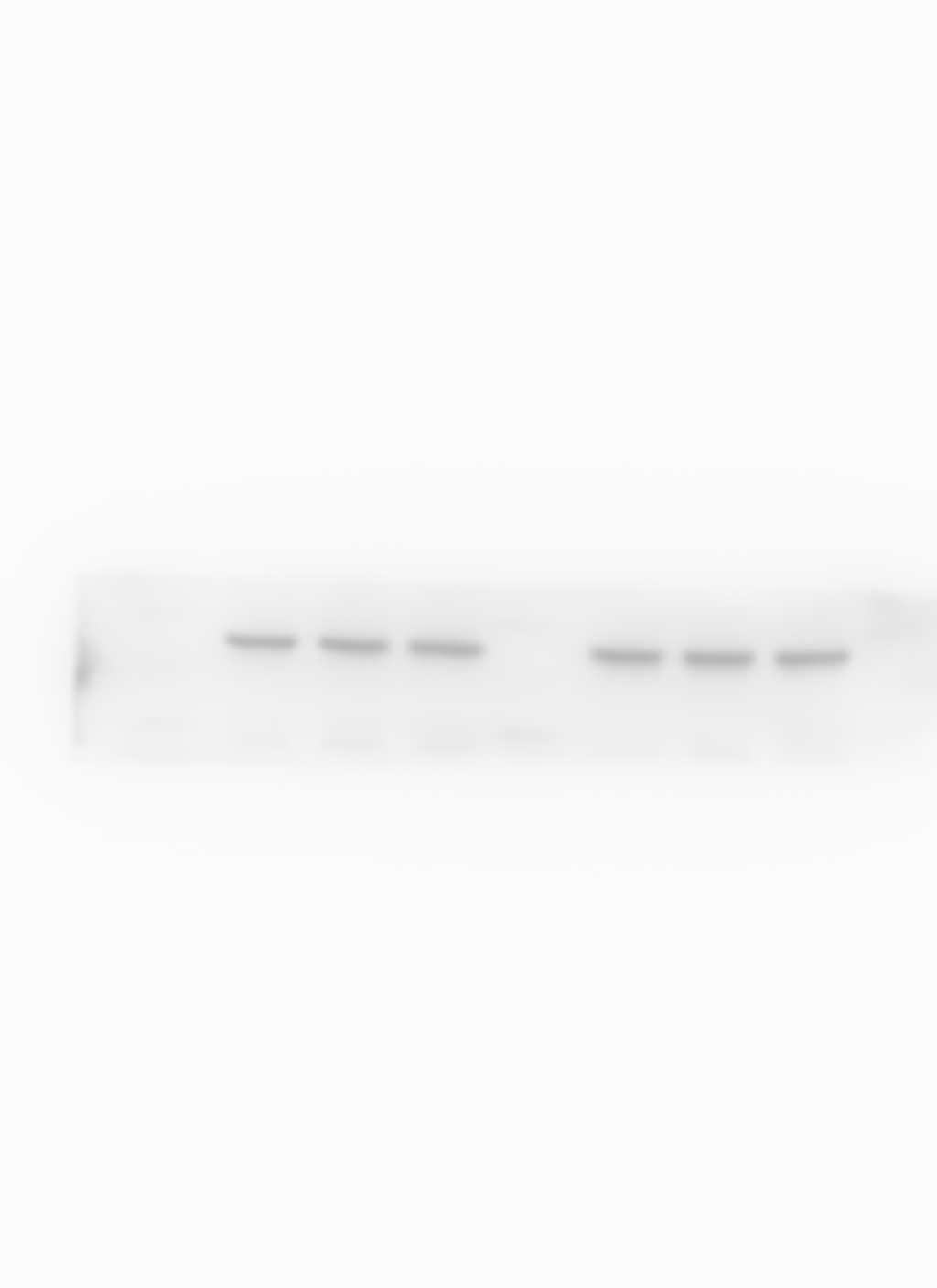

Supplement: Figure 4—source data 1. [file elife-97327-fig4-data1.zip › Figure4-Source data 1/tubulin.tif]

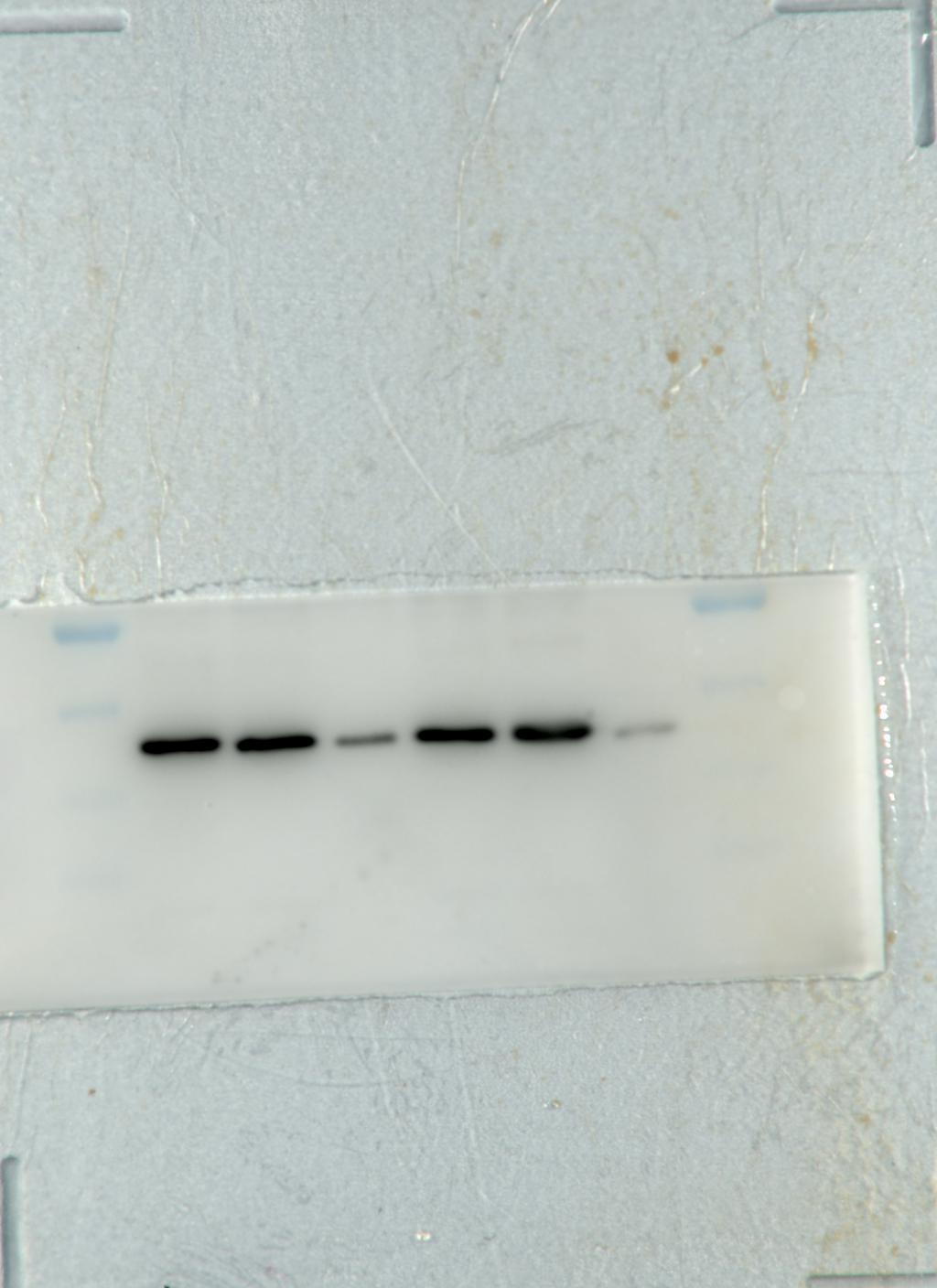

Supplement: Figure 4—source data 2. [file elife-97327-fig4-data2.zip › Figure4-Source data 2/F4C-RGS10-2 +Marker.jpg]

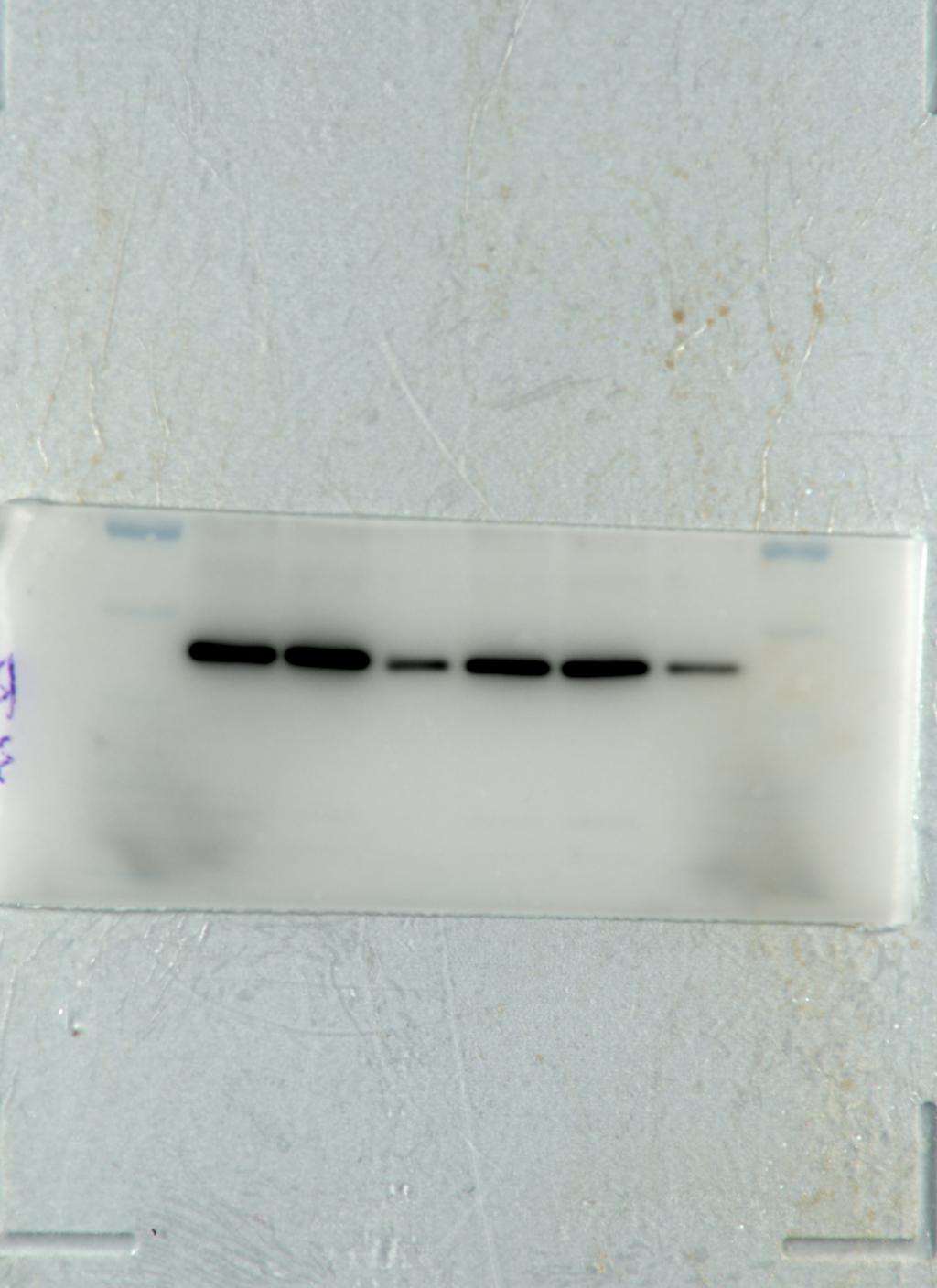

Supplement: Figure 4—source data 2. [file elife-97327-fig4-data2.zip › Figure4-Source data 2/F4C-RGS10-3+Marker.jpg]

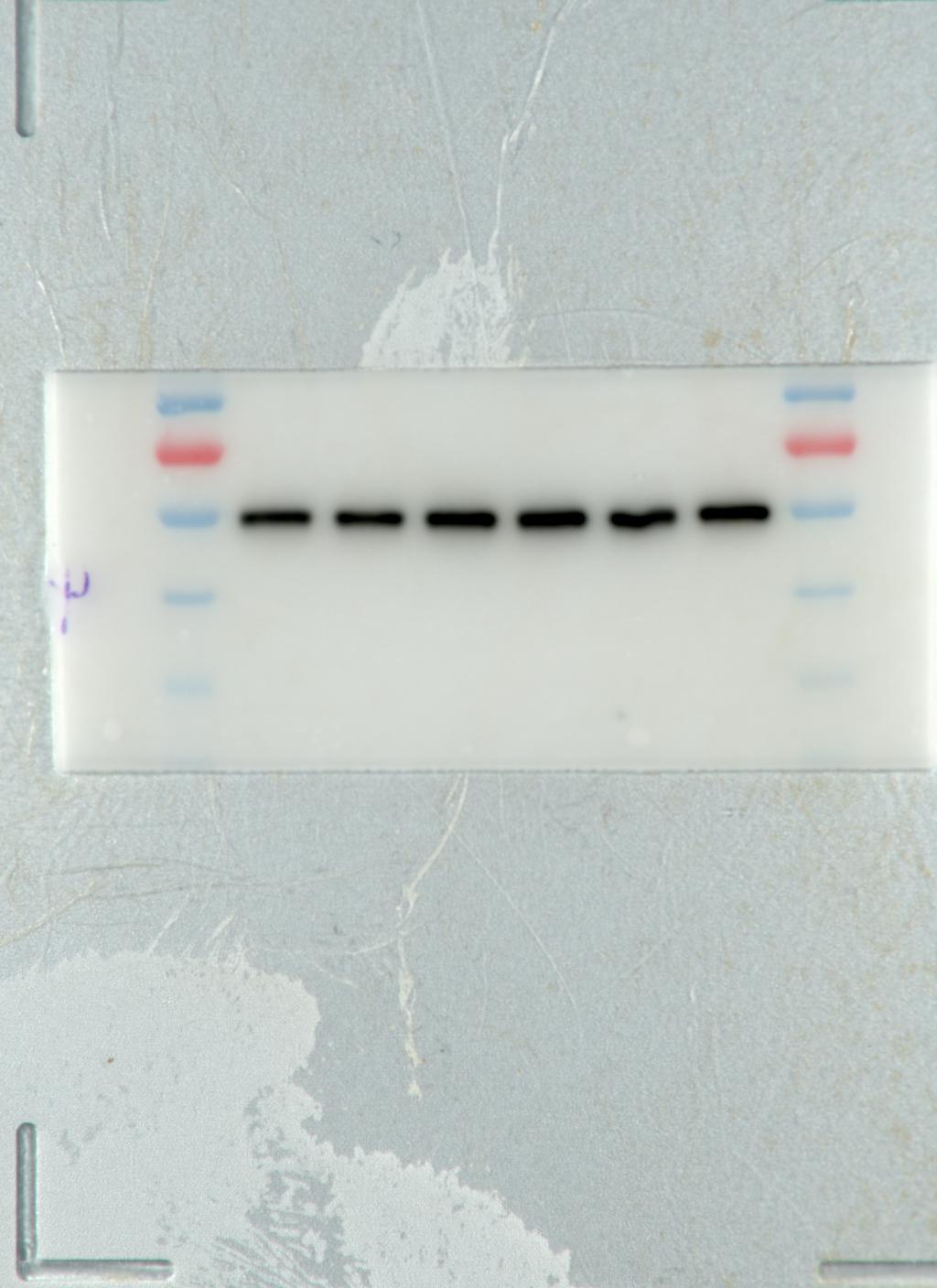

Supplement: Figure 4—source data 2. [file elife-97327-fig4-data2.zip › Figure4-Source data 2/F4C-TU +Marker.jpg]

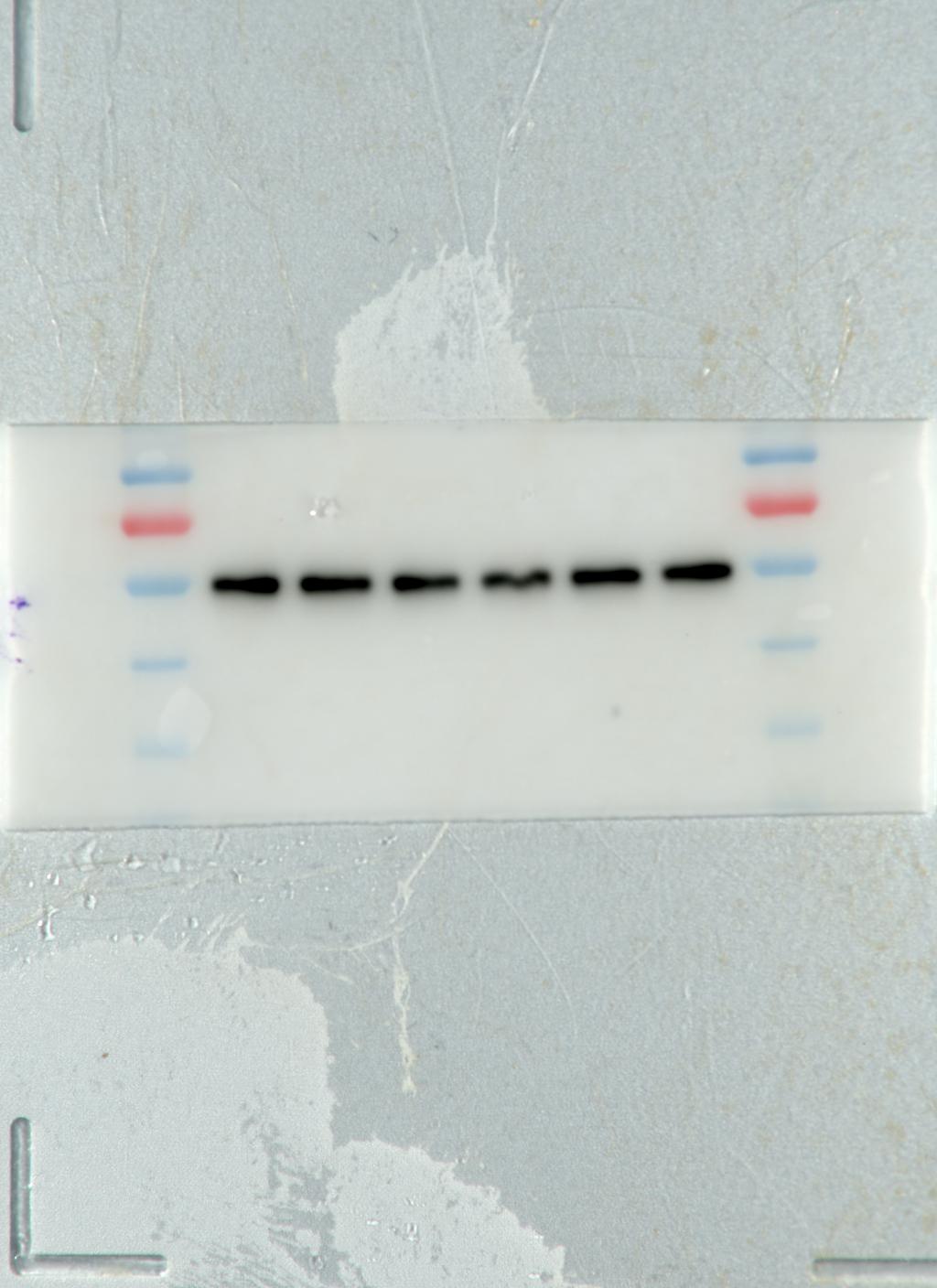

Supplement: Figure 4—source data 2. [file elife-97327-fig4-data2.zip › Figure4-Source data 2/F4C-TU-2 +Marker.jpg]

The expression of RGS10/ $\beta$ -Tubulin

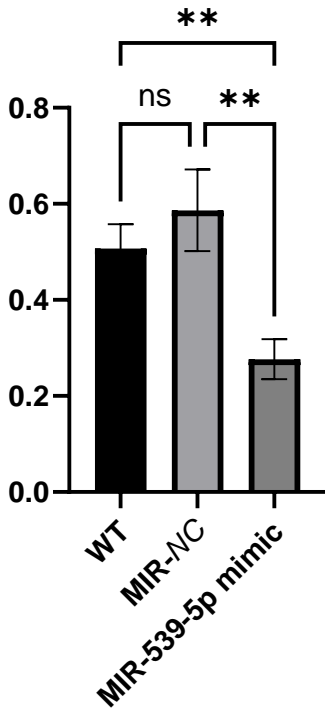

- WT
- MIR-NC
- MIR-539-5p mimic

Supplement: Figure 4—source data 3. [file elife-97327-fig4-data3.pdf]

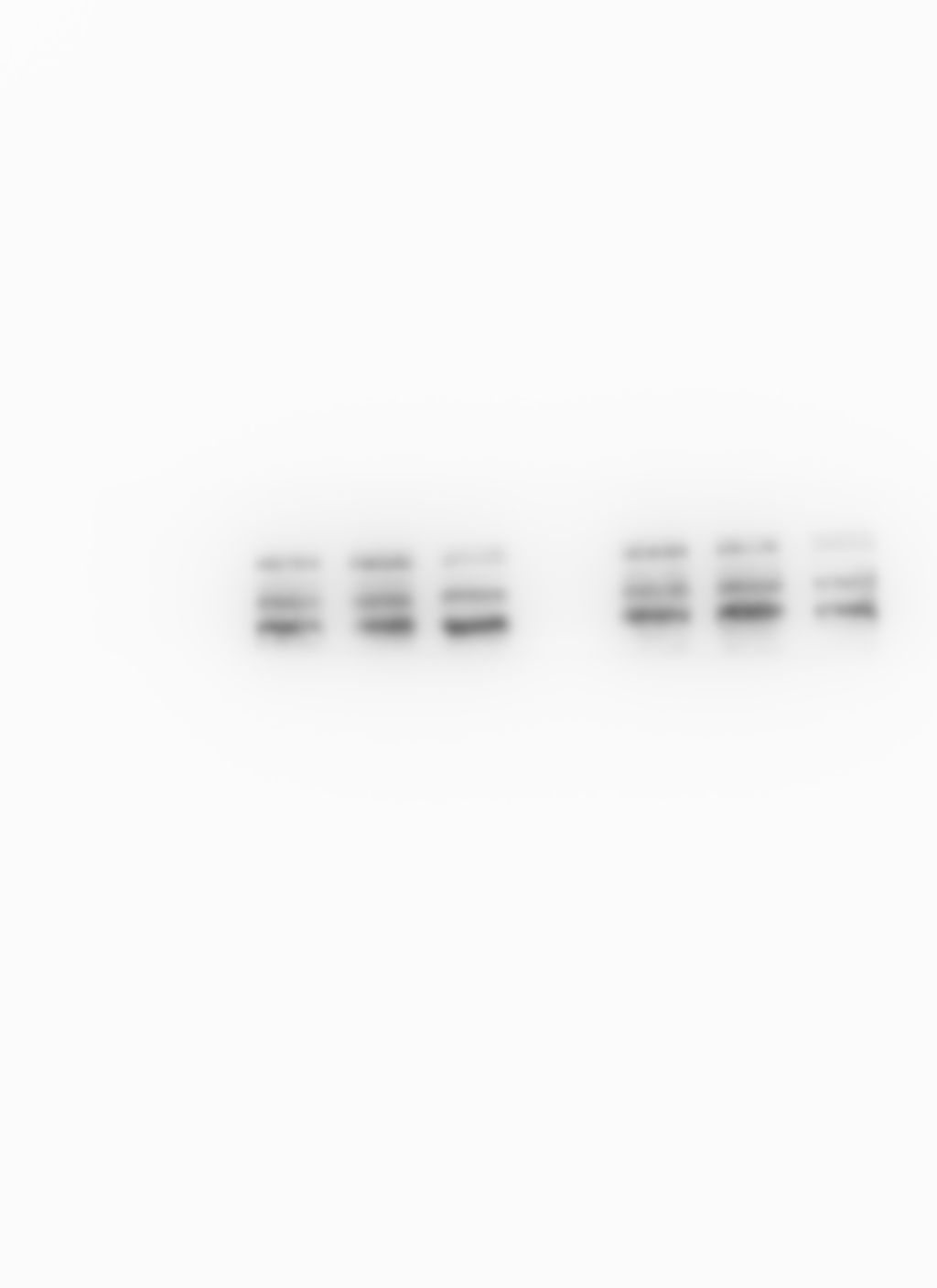

Supplement: Figure 4—source data 4. [file elife-97327-fig4-data4.zip › Figure4-Source data 4/E-cadherin.tif]

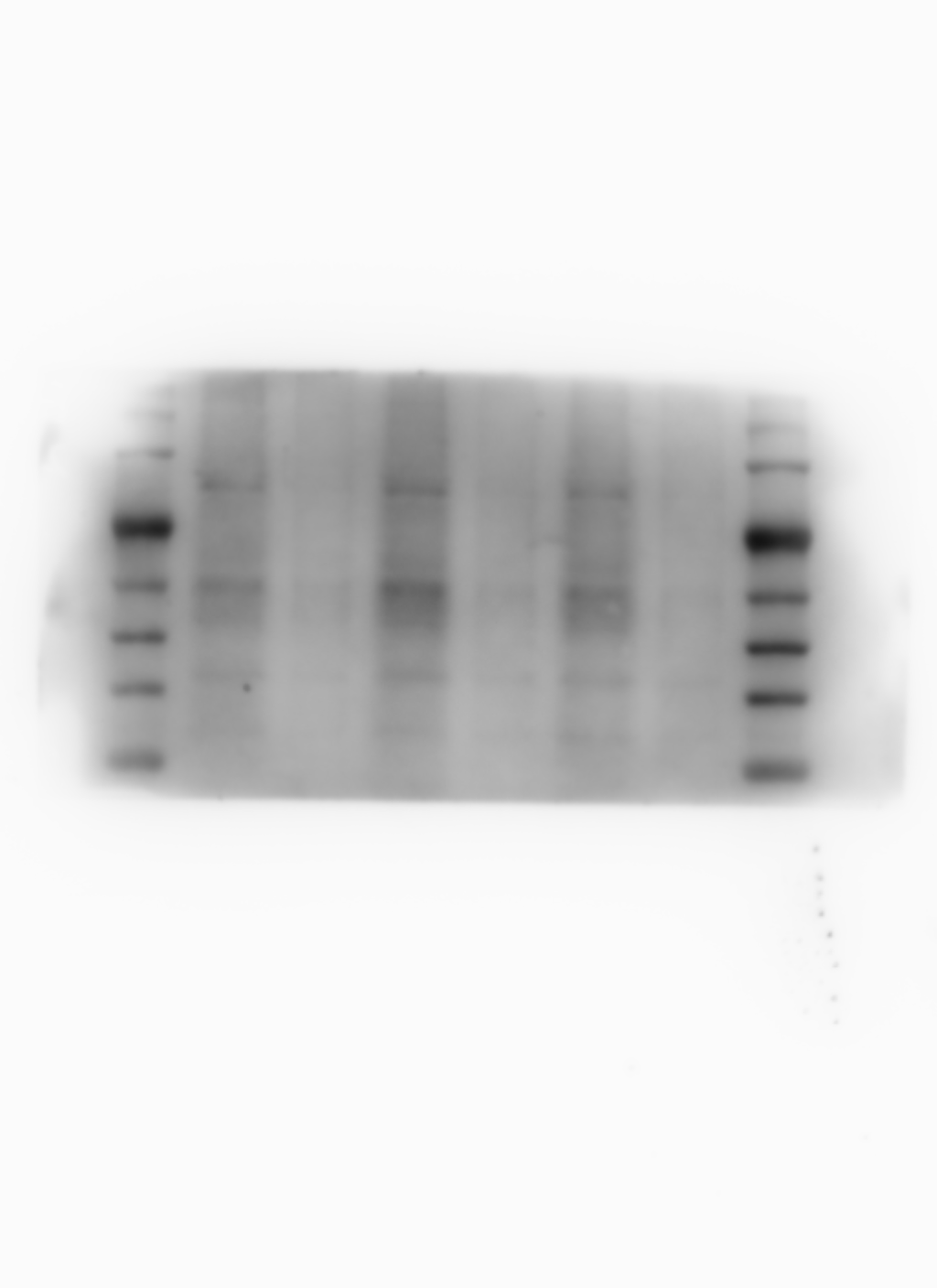

Supplement: Figure 4—source data 4. [file elife-97327-fig4-data4.zip › Figure4-Source data 4/F4H-Eca .tif]

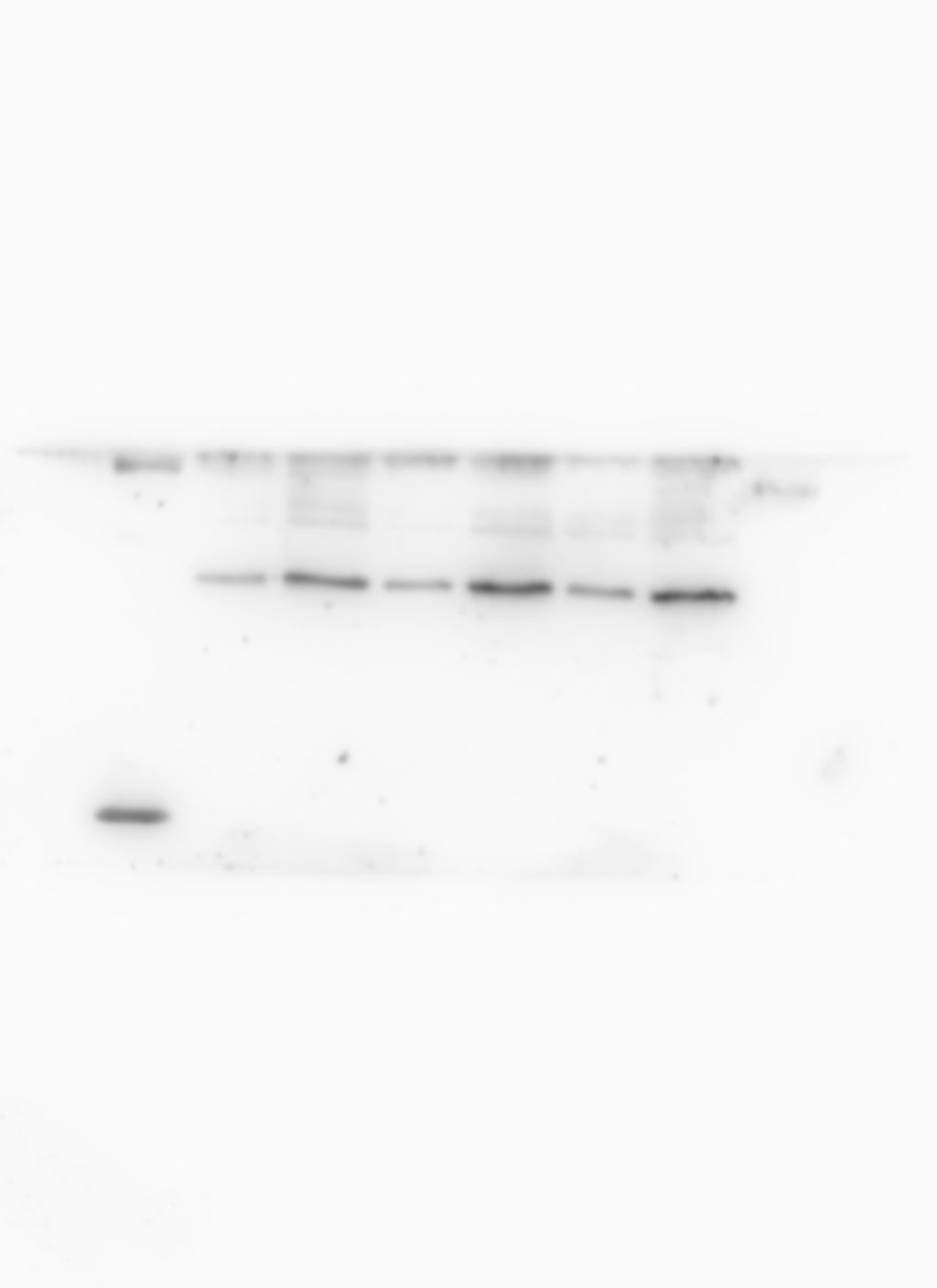

Supplement: Figure 4—source data 4. [file elife-97327-fig4-data4.zip › Figure4-Source data 4/F4H-LCN2 .tif]

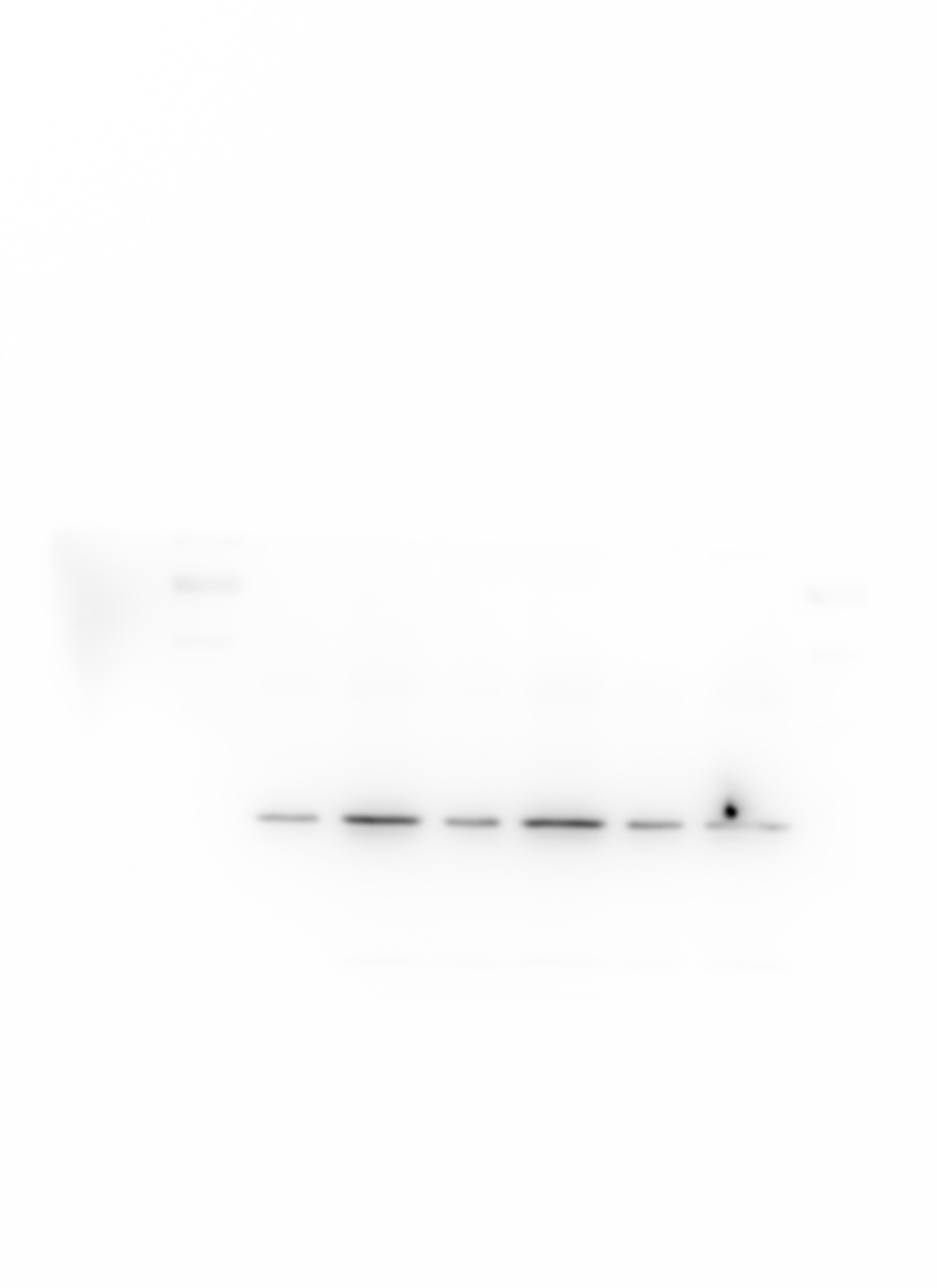

Supplement: Figure 4—source data 4. [file elife-97327-fig4-data4.zip › Figure4-Source data 4/F4H-Snail .tif]

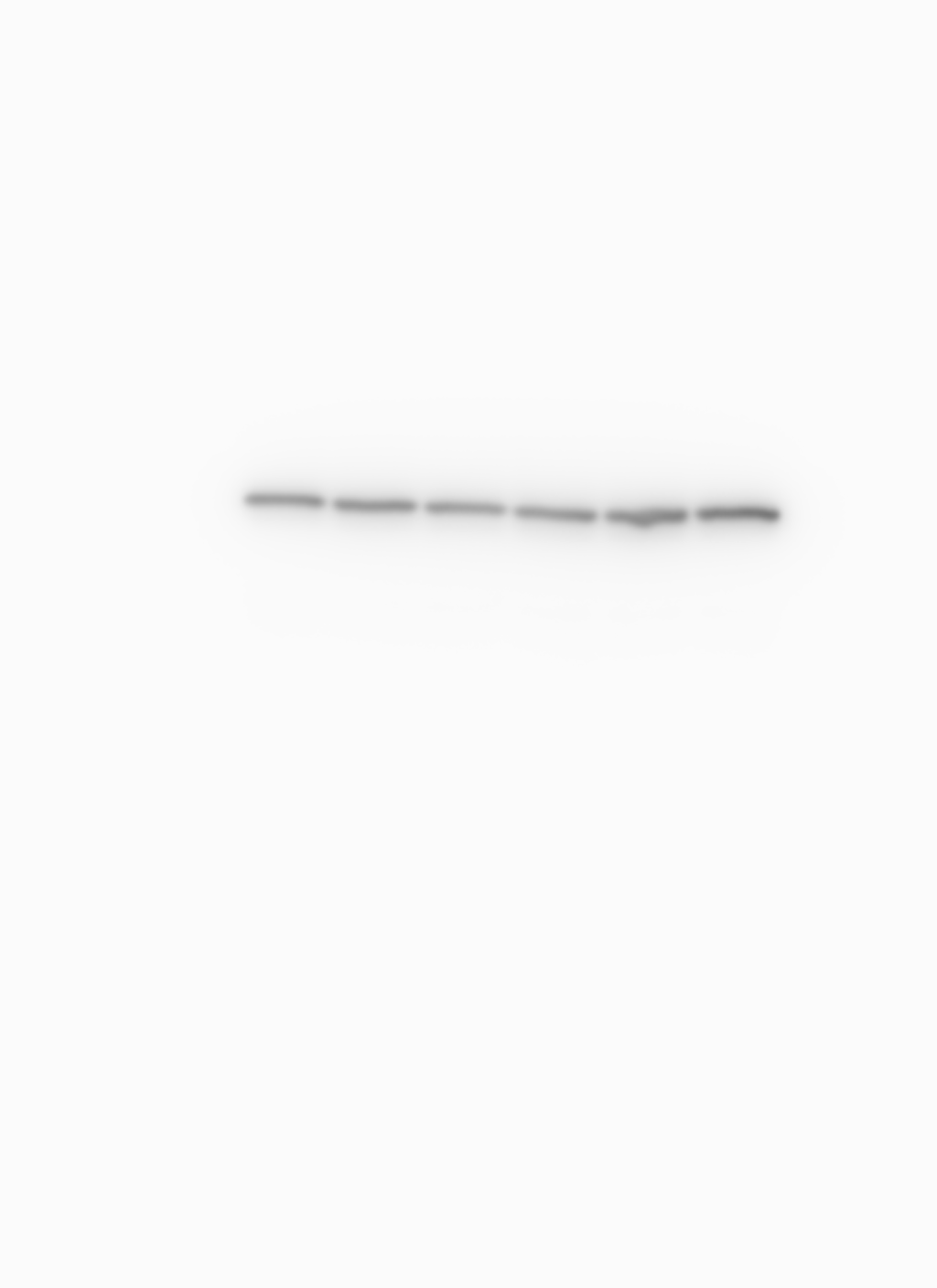

Supplement: Figure 4—source data 4. [file elife-97327-fig4-data4.zip › Figure4-Source data 4/F4H-tubulin .tif]

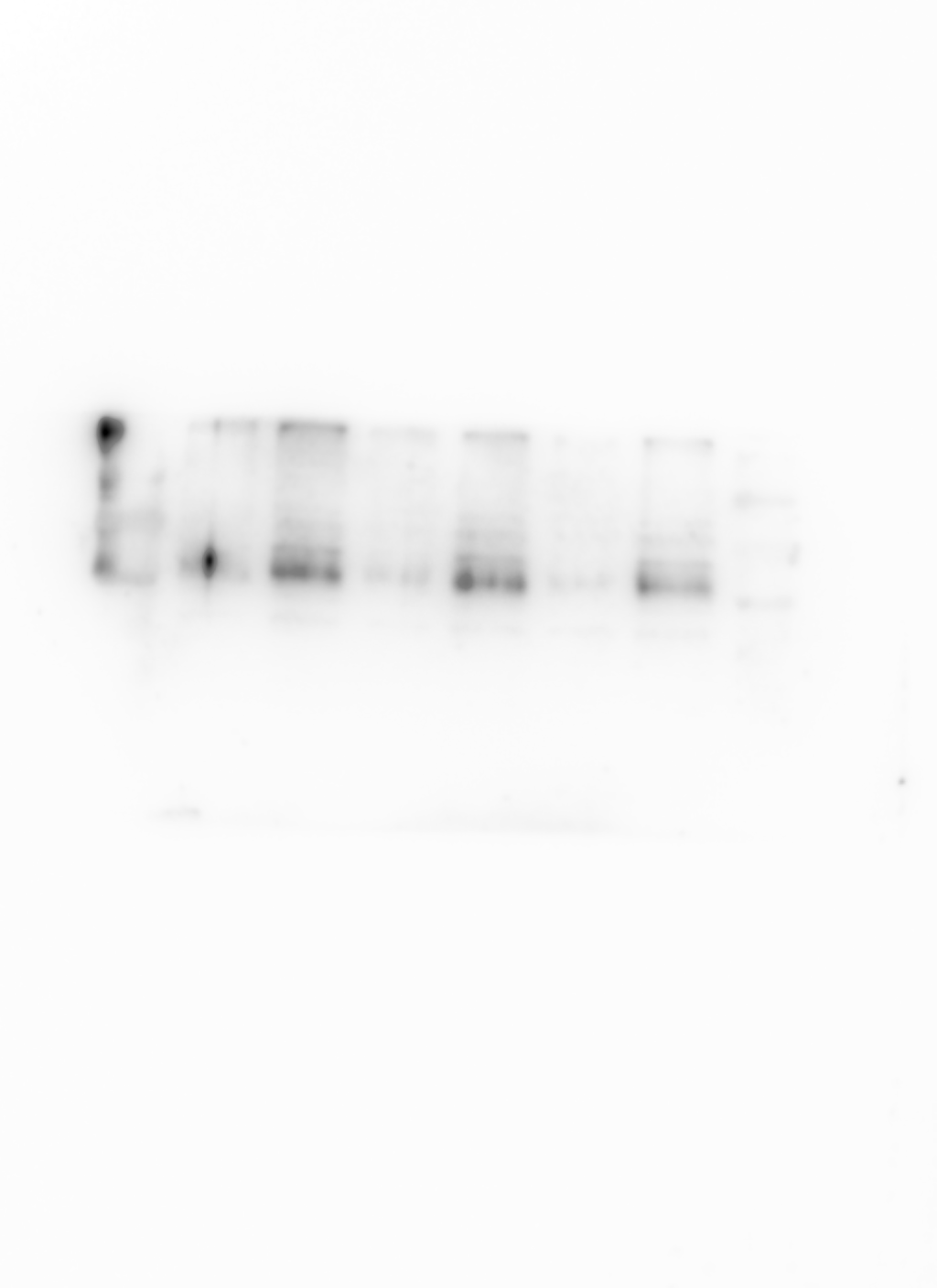

Supplement: Figure 4—source data 4. [file elife-97327-fig4-data4.zip › Figure4-Source data 4/F4H-Vimentin .tif]

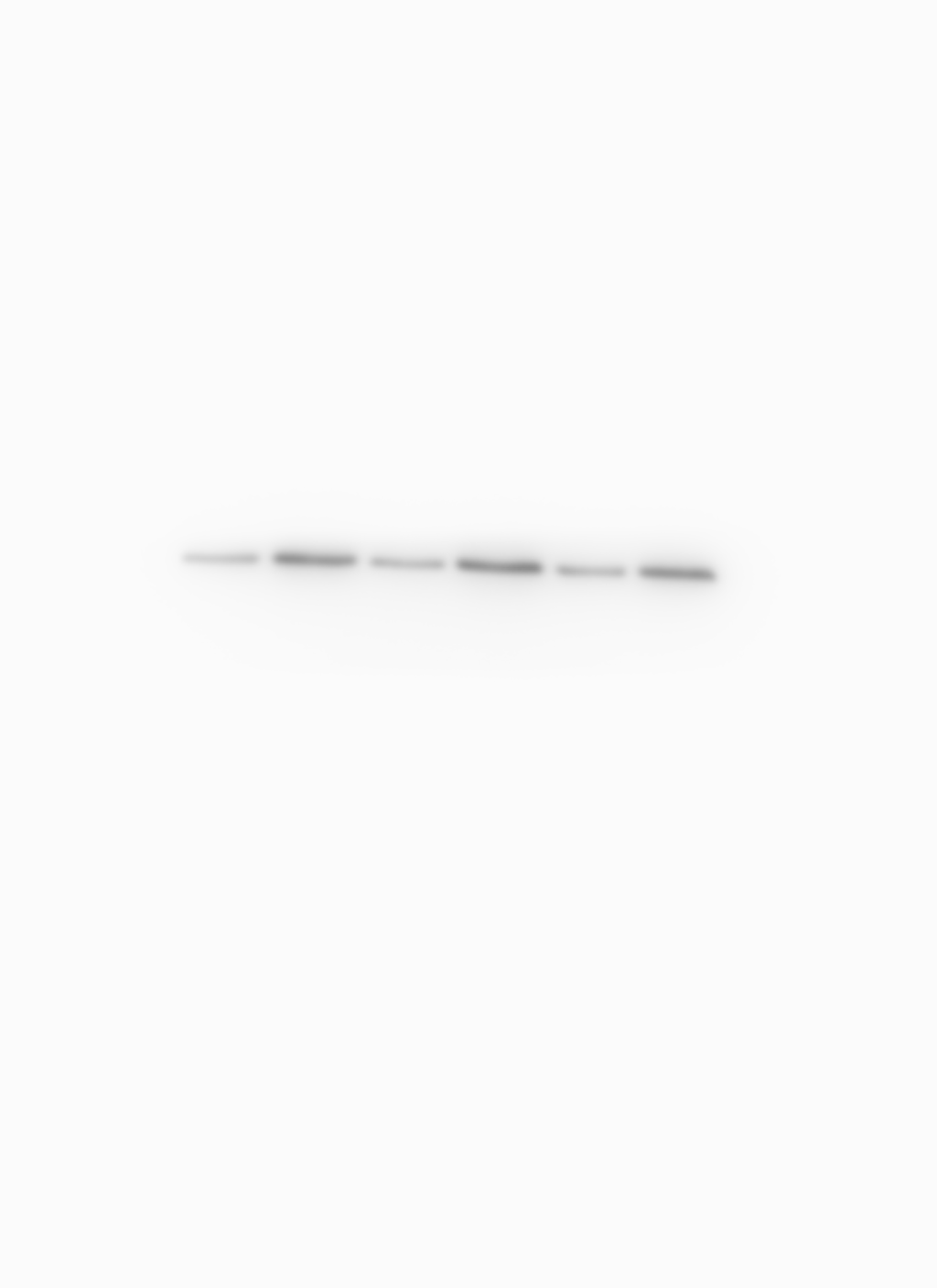

Supplement: Figure 4—source data 4. [file elife-97327-fig4-data4.zip › Figure4-Source data 4/snail.tif]

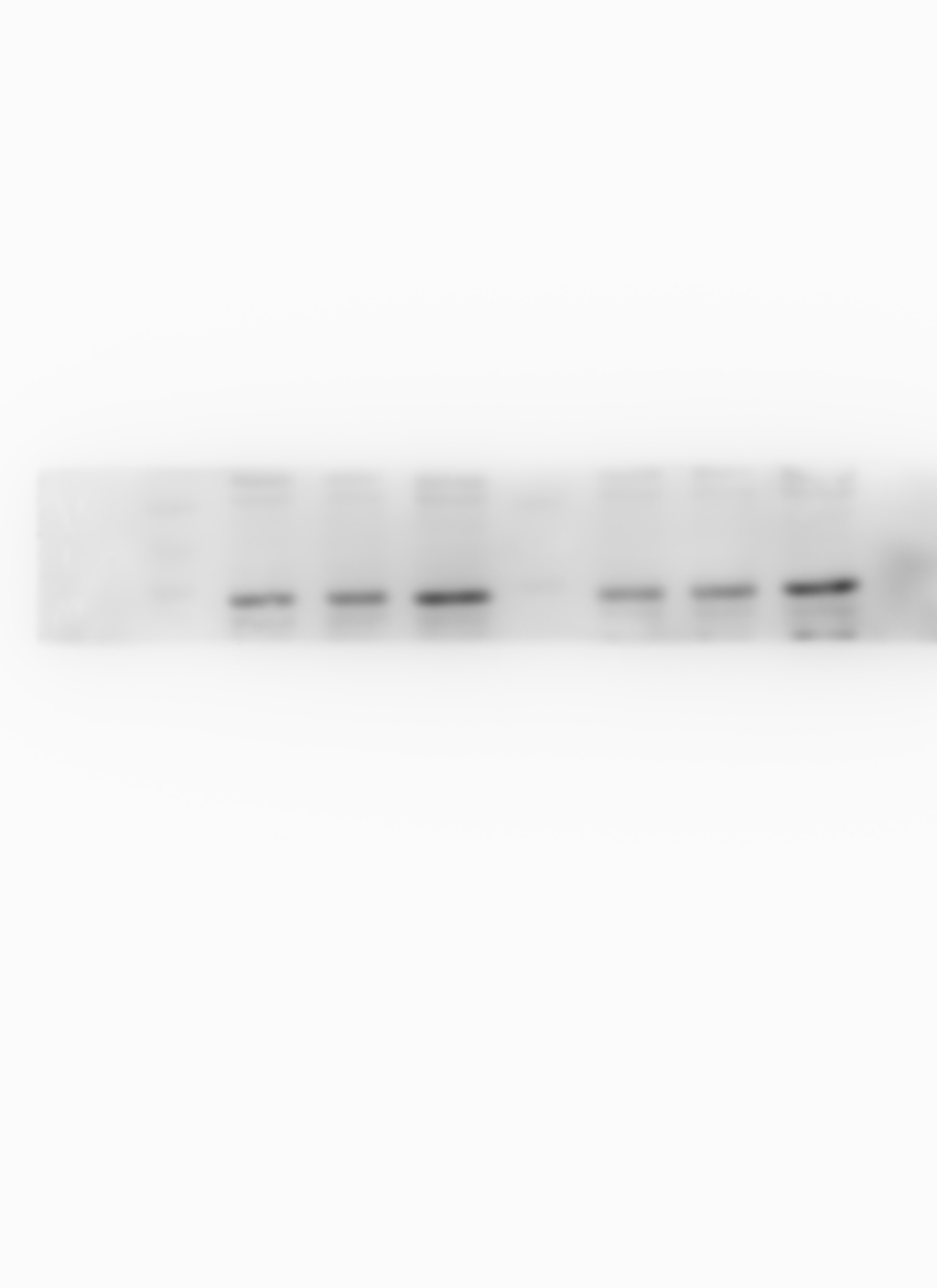

Supplement: Figure 4—source data 4. [file elife-97327-fig4-data4.zip › Figure4-Source data 4/vimentin.tif]

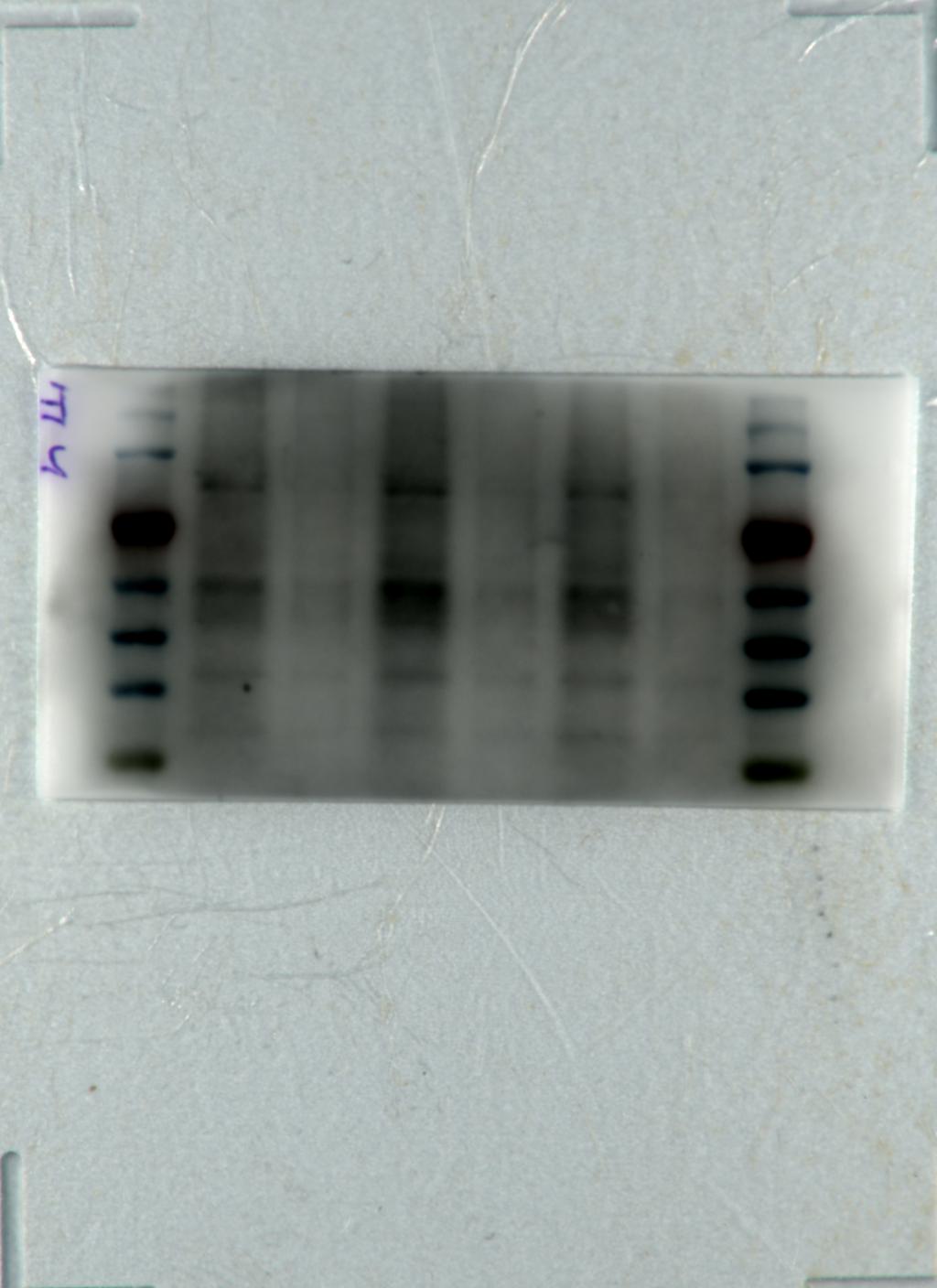

Supplement: Figure 4—source data 5. [file elife-97327-fig4-data5.zip › Figure4-Source data 5/F4H-Eca +Marker.jpg]

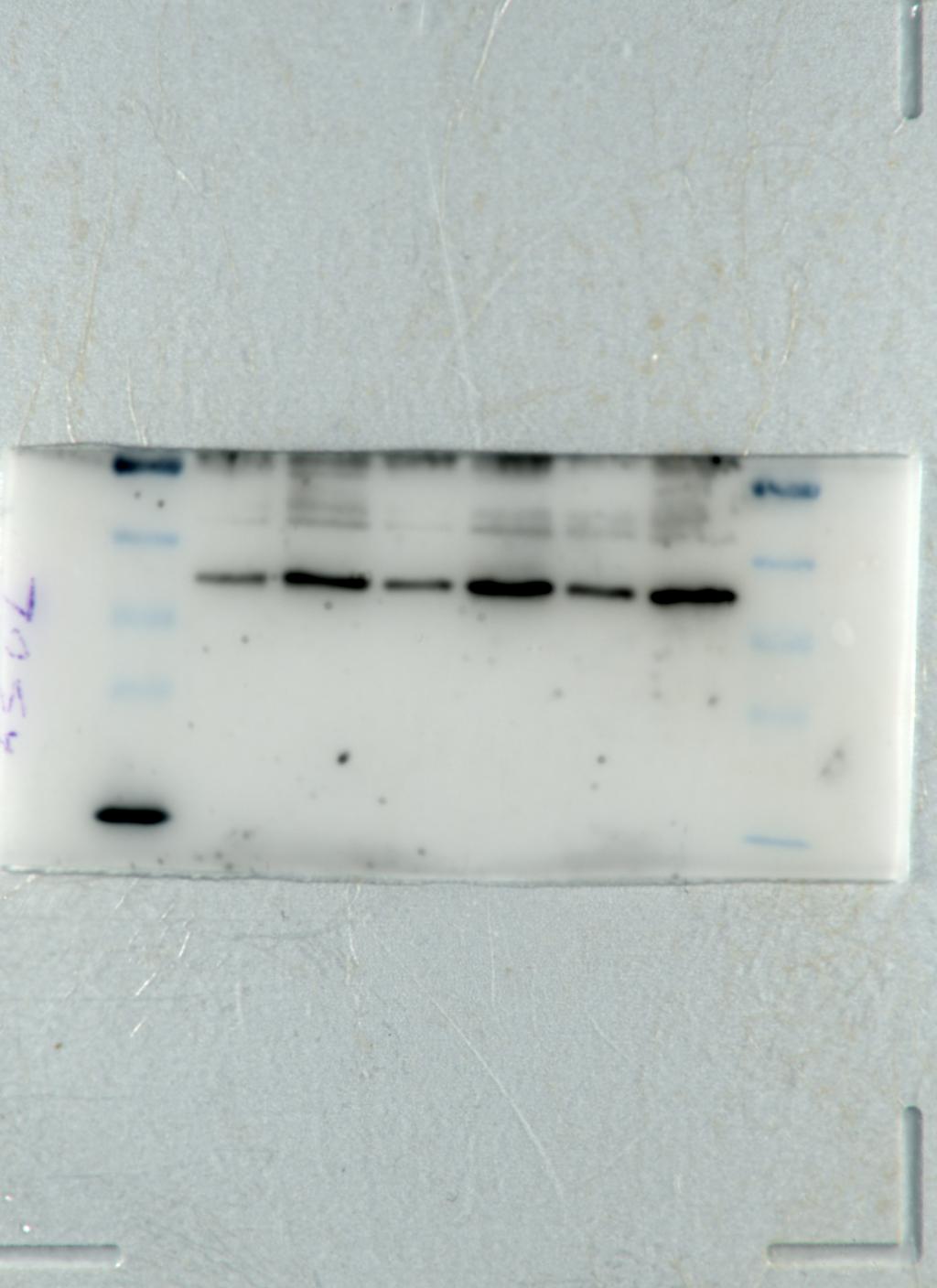

Supplement: Figure 4—source data 5. [file elife-97327-fig4-data5.zip › Figure4-Source data 5/F4H-LCN2 +Marker.jpg]

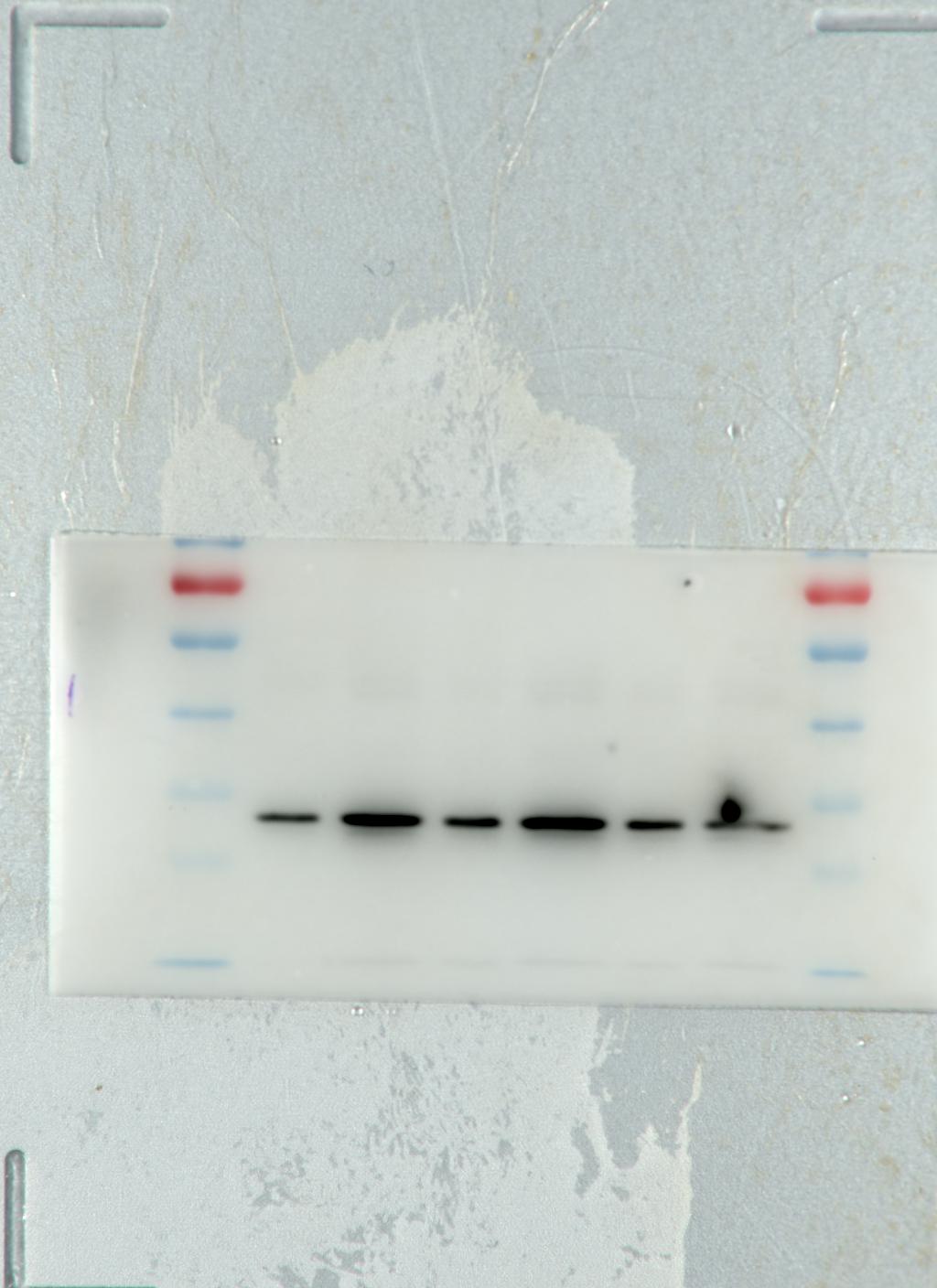

Supplement: Figure 4—source data 5. [file elife-97327-fig4-data5.zip › Figure4-Source data 5/F4H-Snail +Marker.jpg]

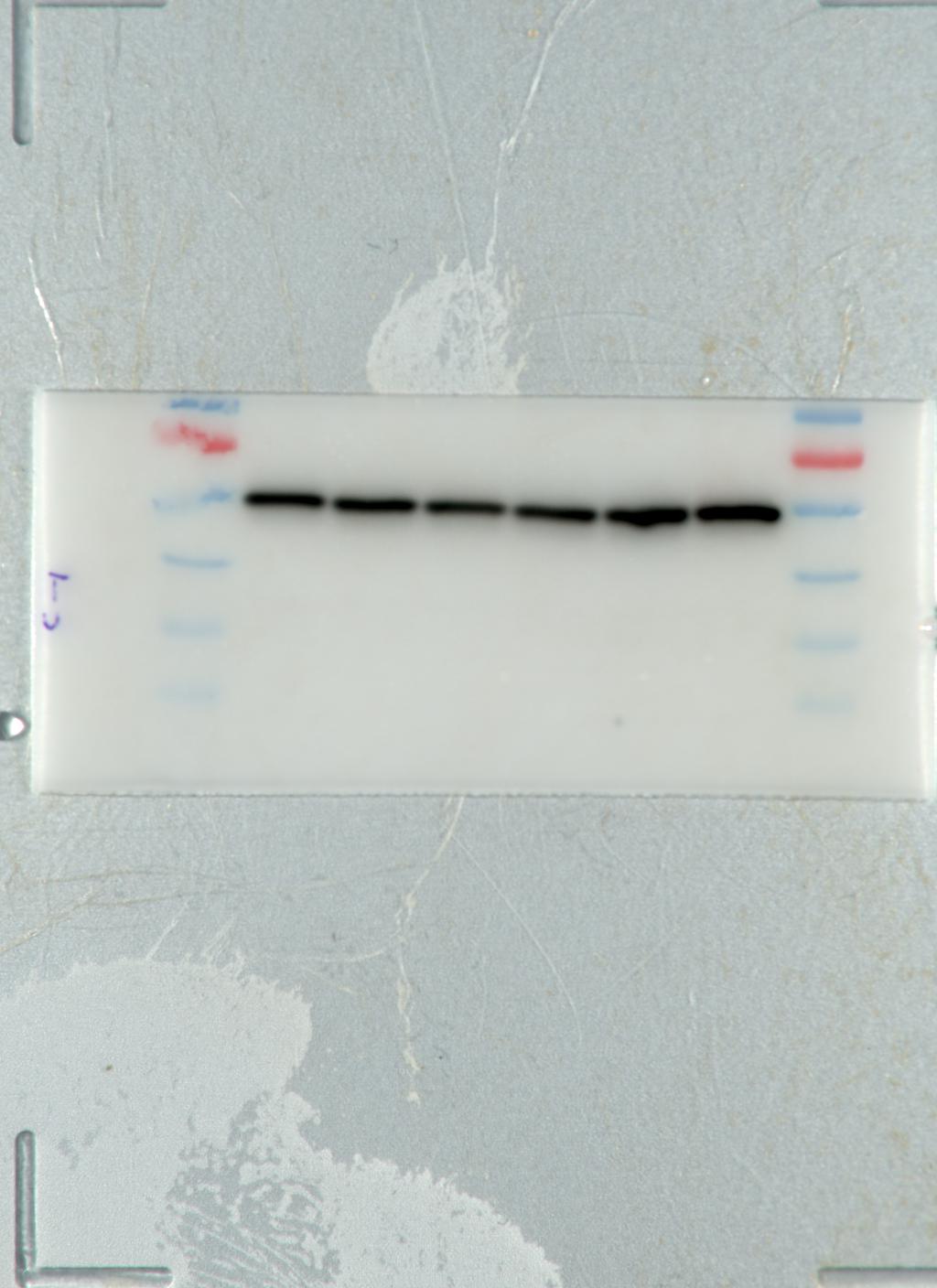

Supplement: Figure 4—source data 5. [file elife-97327-fig4-data5.zip › Figure4-Source data 5/F4H-tubulin +Marker.jpg]

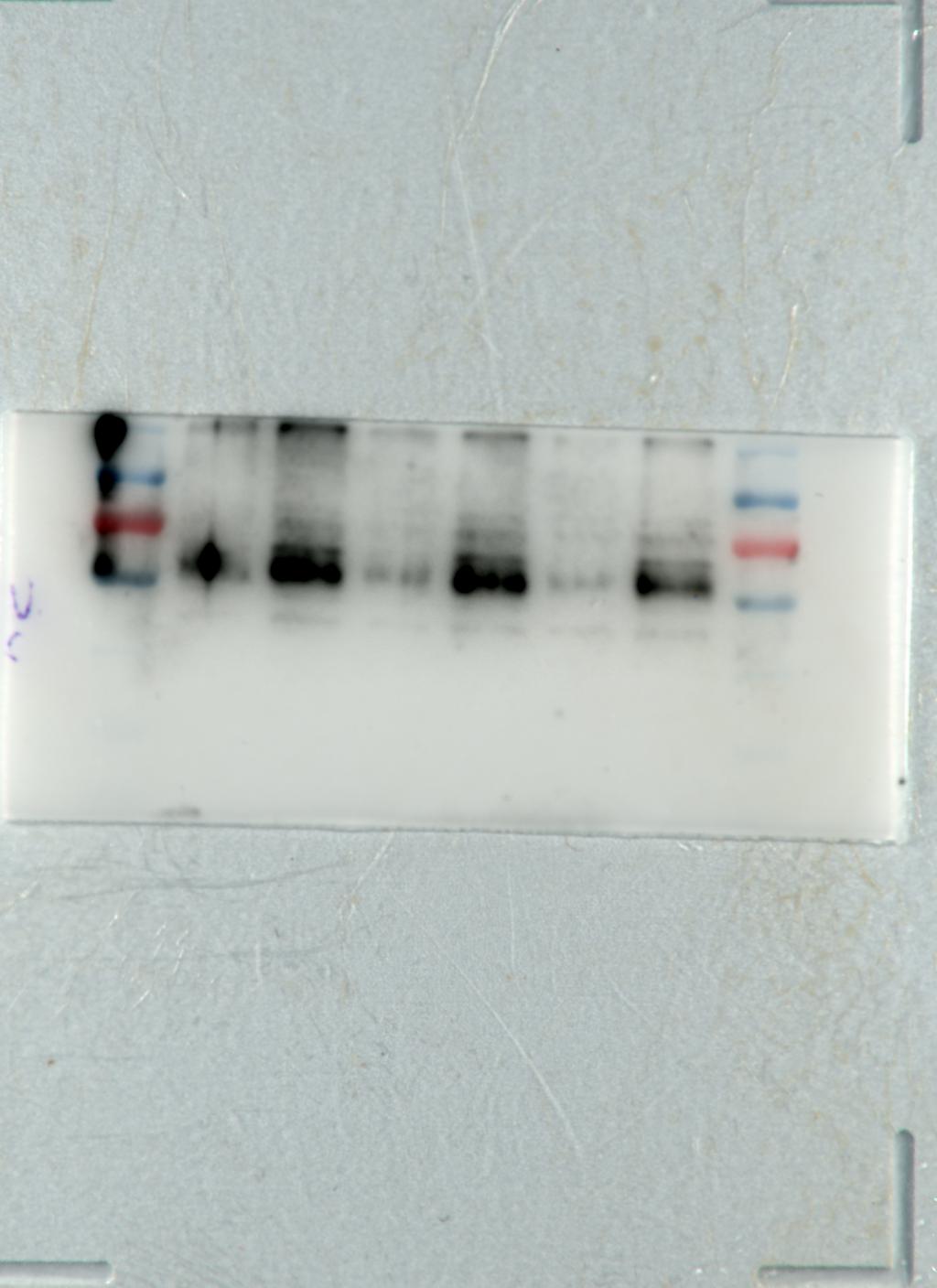

Supplement: Figure 4—source data 5. [file elife-97327-fig4-data5.zip › Figure4-Source data 5/F4H-Vimentin +Marker.jpg]

LCN2/ $\beta$ -Tubulin

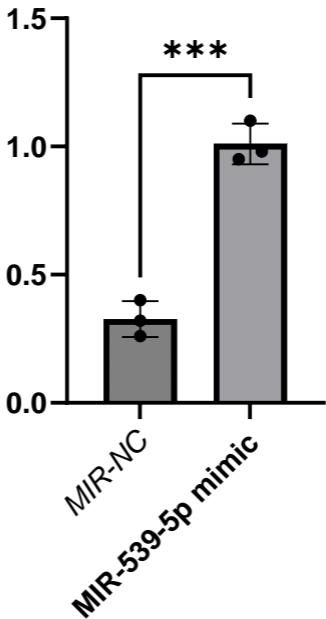

Supplement: Figure 4—source data 6. [file elife-97327-fig4-data6.pdf]

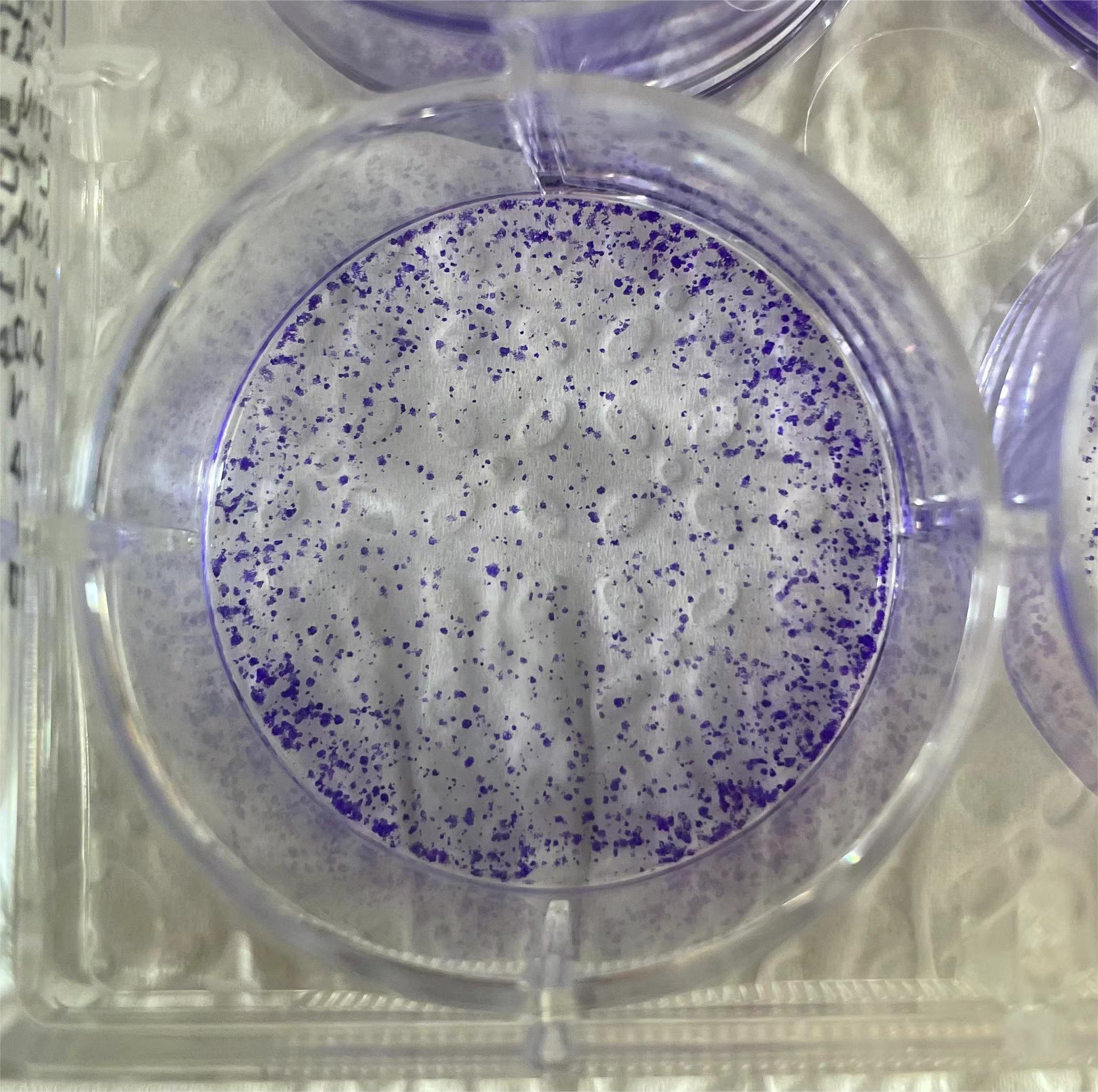

Supplement: Figure 4—source data 8. [file elife-97327-fig4-data8.zip › Figure4-Source data 8/F4E-miR-539-5p.jpg]

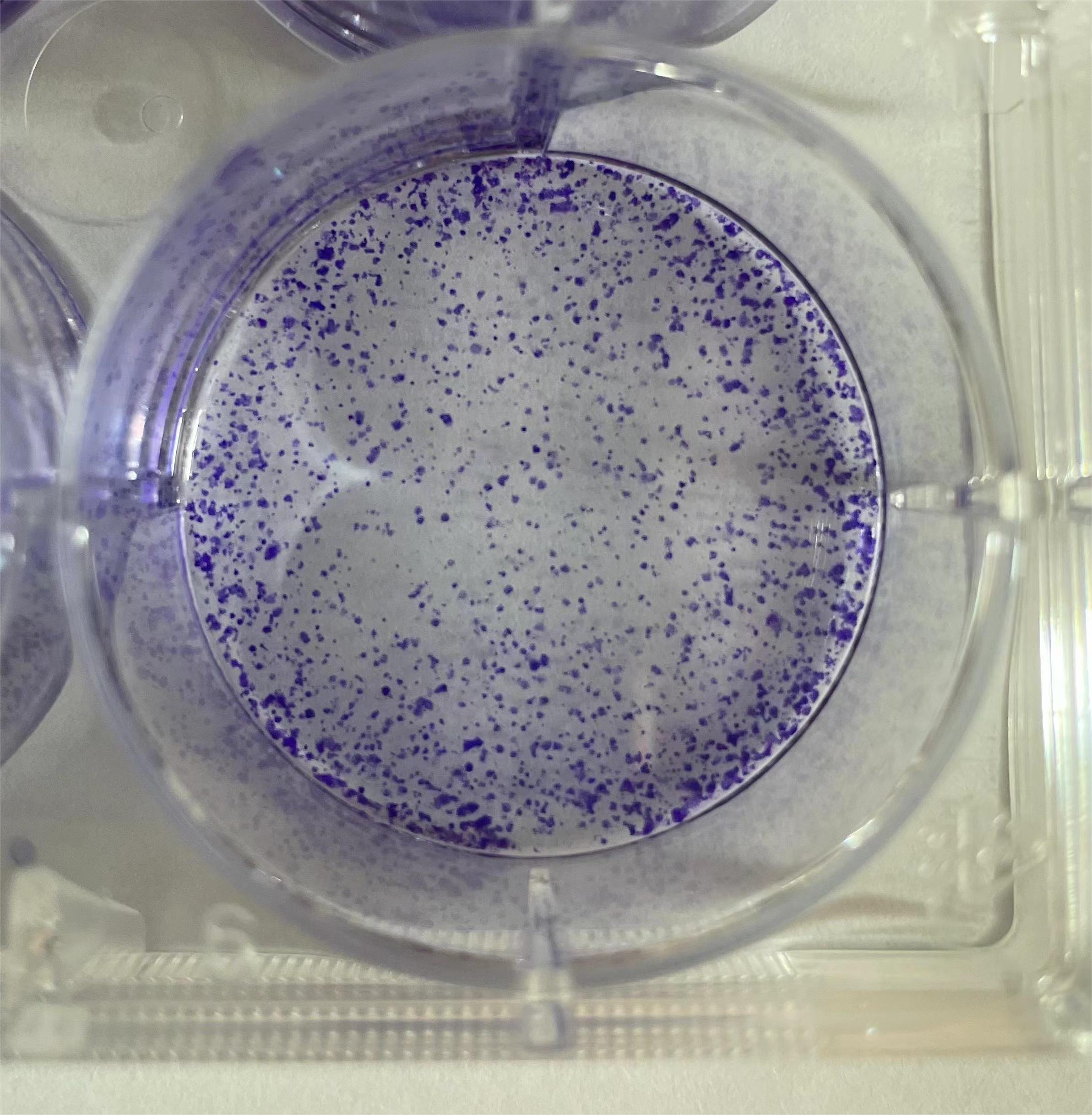

Supplement: Figure 4—source data 8. [file elife-97327-fig4-data8.zip › Figure4-Source data 8/F4E-NC.jpg]

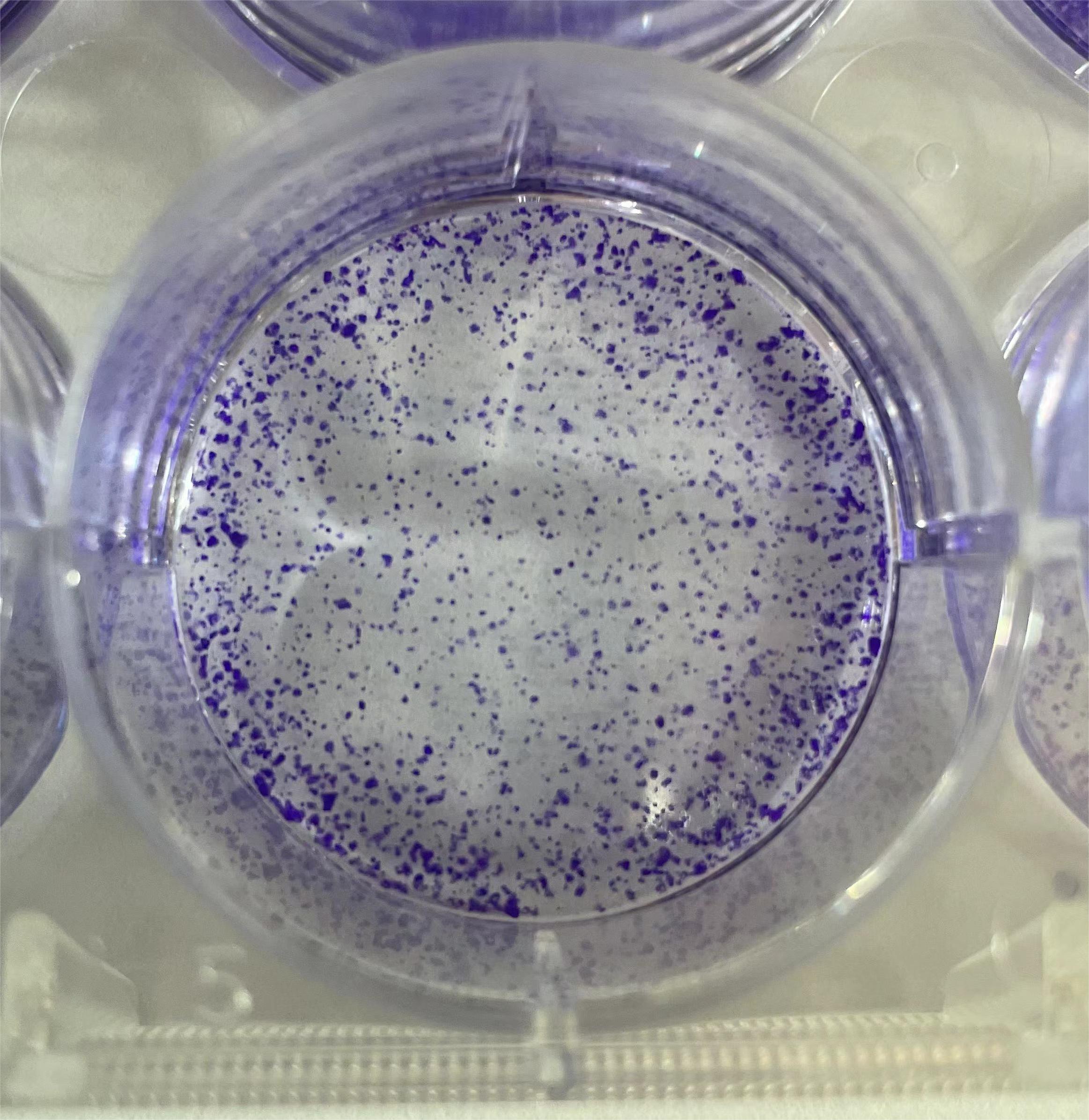

Supplement: Figure 4—source data 8. [file elife-97327-fig4-data8.zip › Figure4-Source data 8/F4E-WT.jpg]

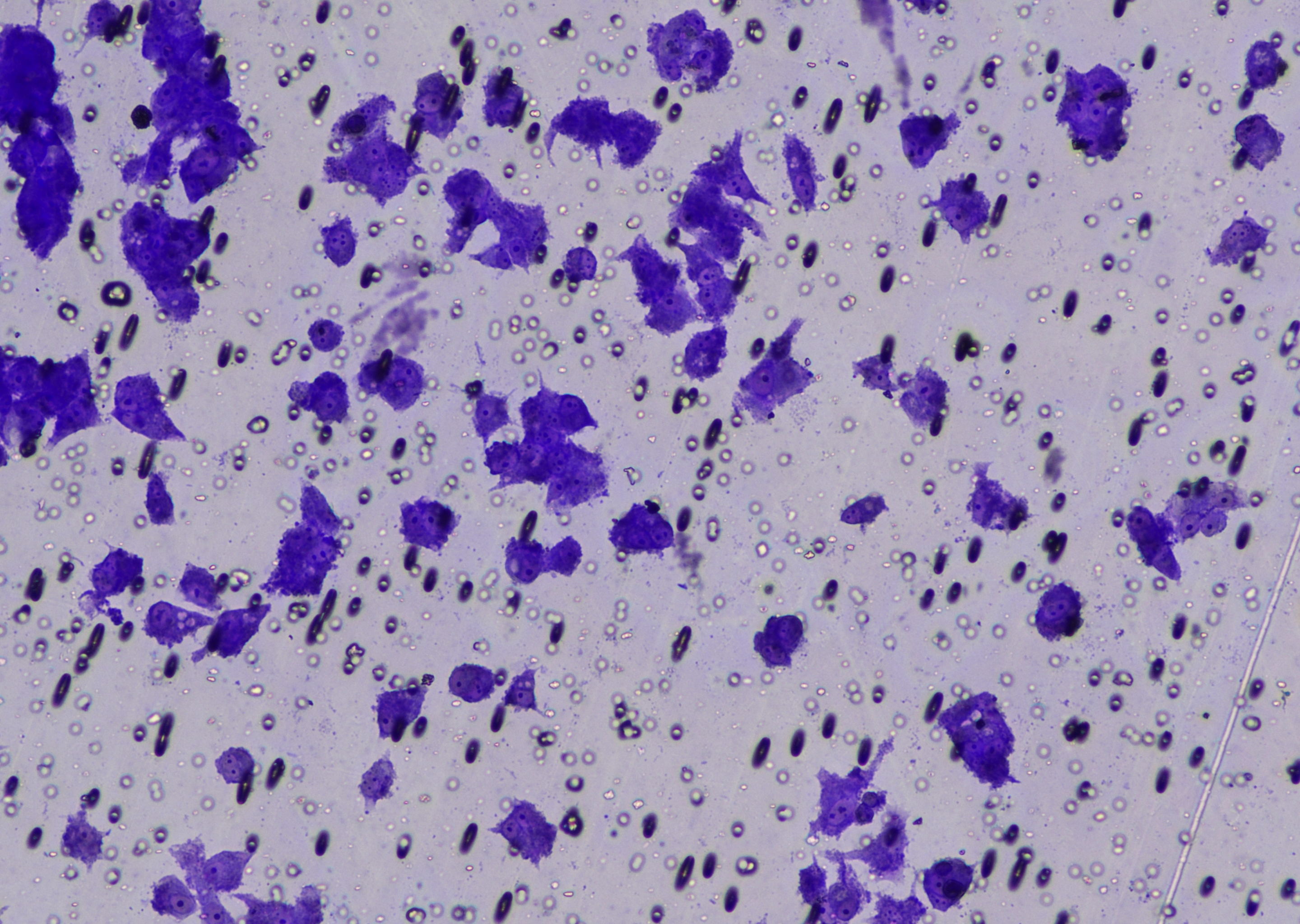

Supplement: Figure 4—source data 9. [file elife-97327-fig4-data9.zip › Figure4-Source data 9/F4F-miR-539-5p mimic.tif]

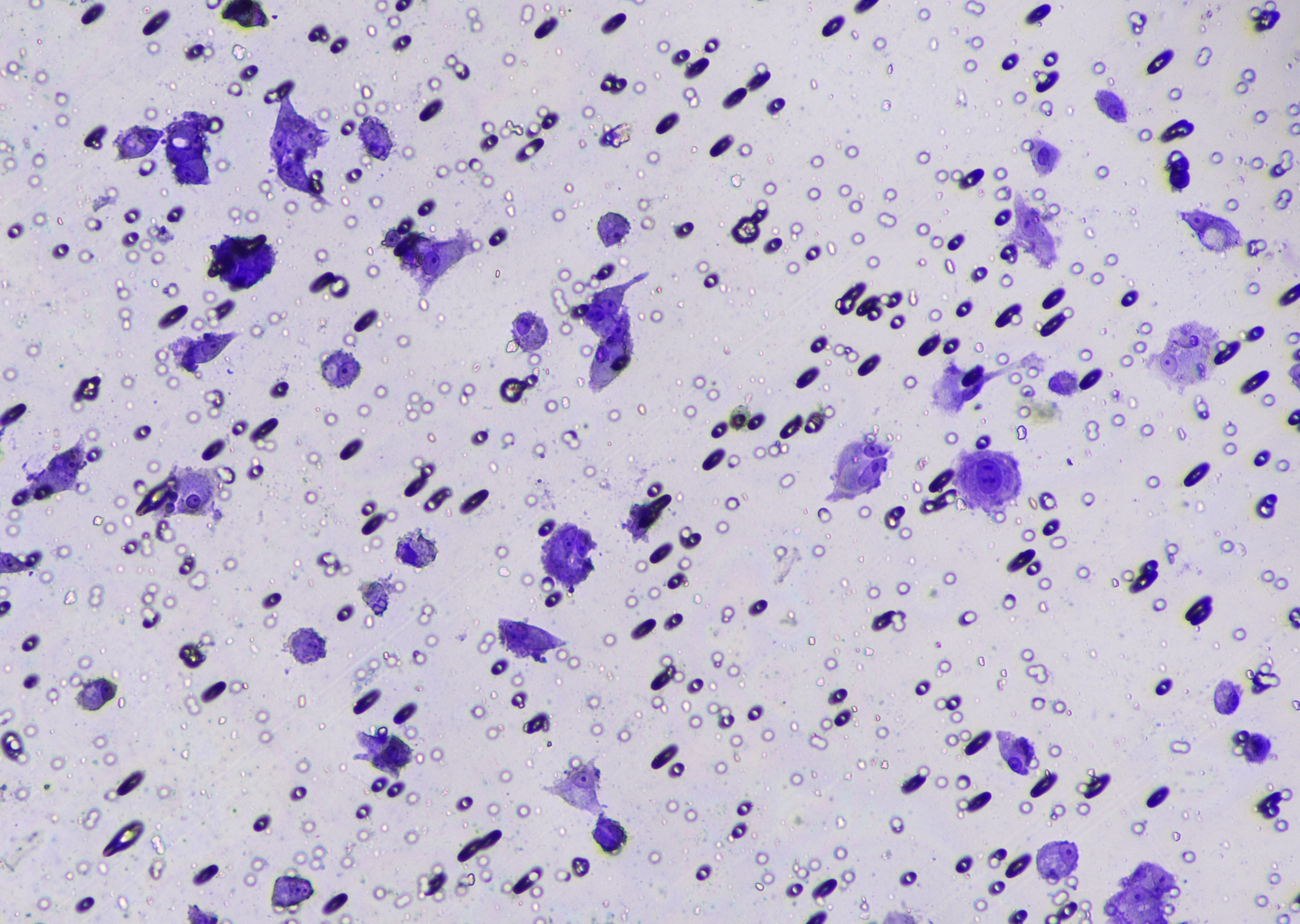

Supplement: Figure 4—source data 9. [file elife-97327-fig4-data9.zip › Figure4-Source data 9/F4F-NC.tif]

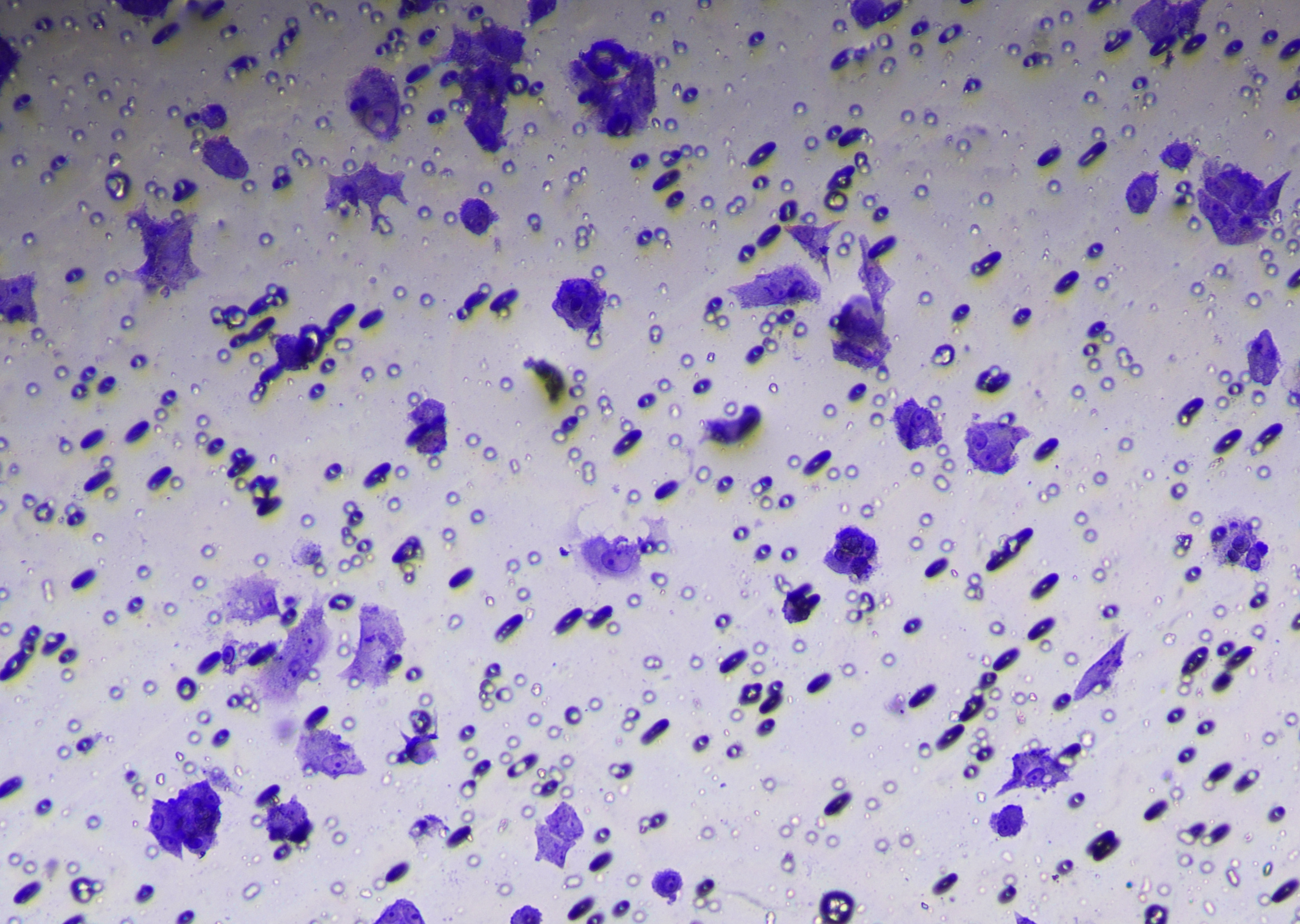

Supplement: Figure 4—source data 9. [file elife-97327-fig4-data9.zip › Figure4-Source data 9/F4F-WT.tif]

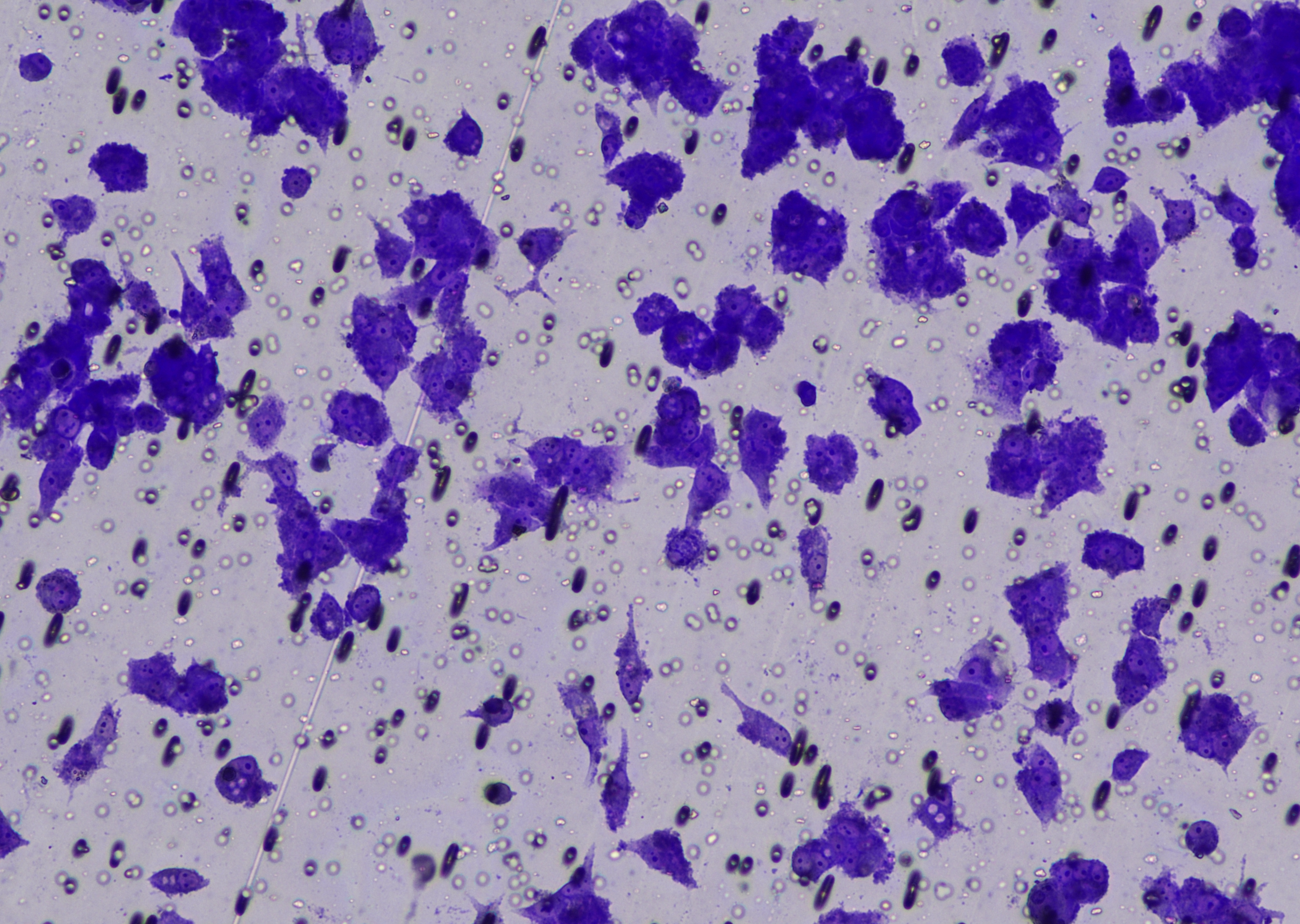

Supplement: Figure 4—source data 10. [file elife-97327-fig4-data10.zip › Figure4-Source data 10/F4G-miR-539-5p mimic.tif]

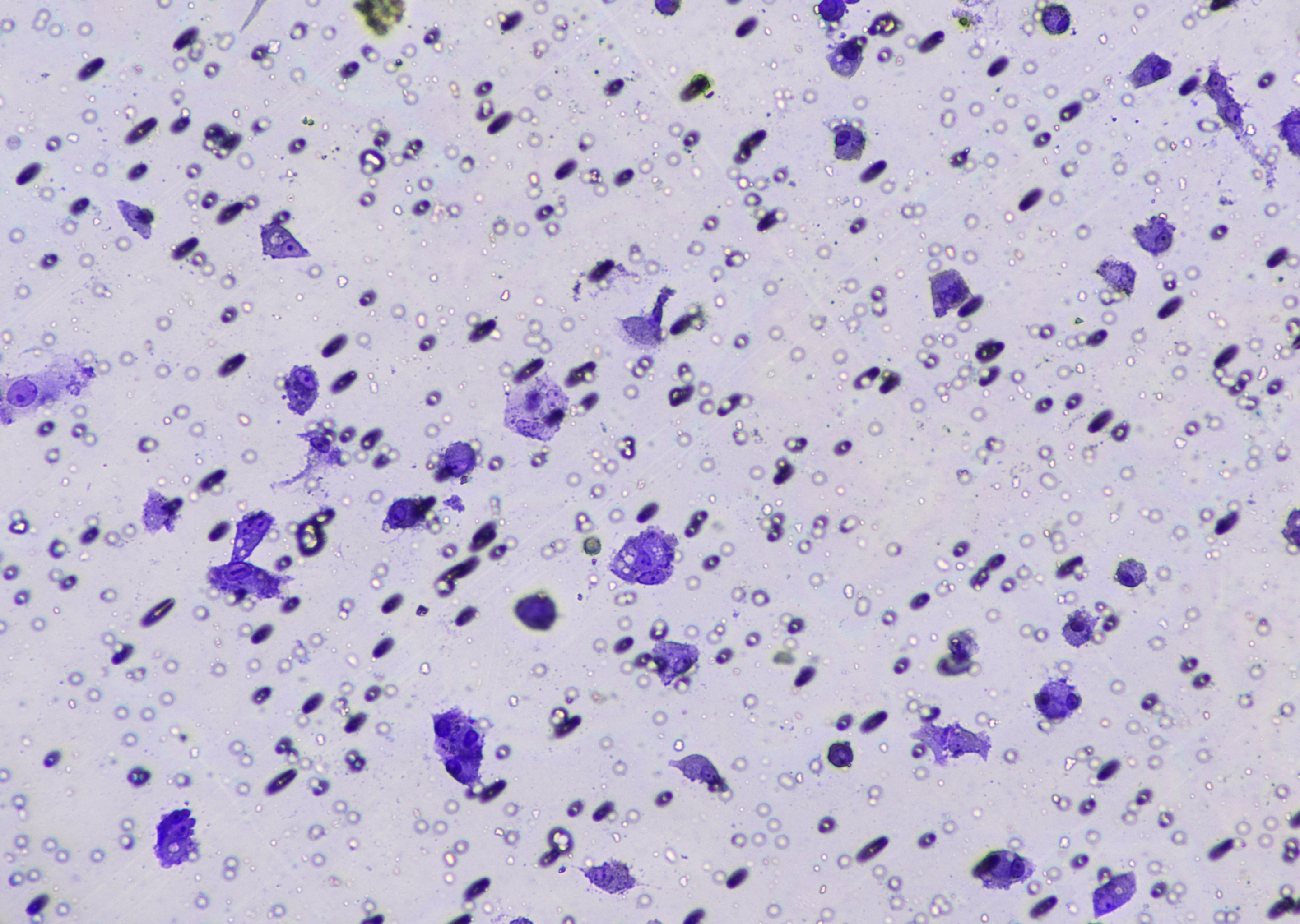

Supplement: Figure 4—source data 10. [file elife-97327-fig4-data10.zip › Figure4-Source data 10/F4G-NC.tif]

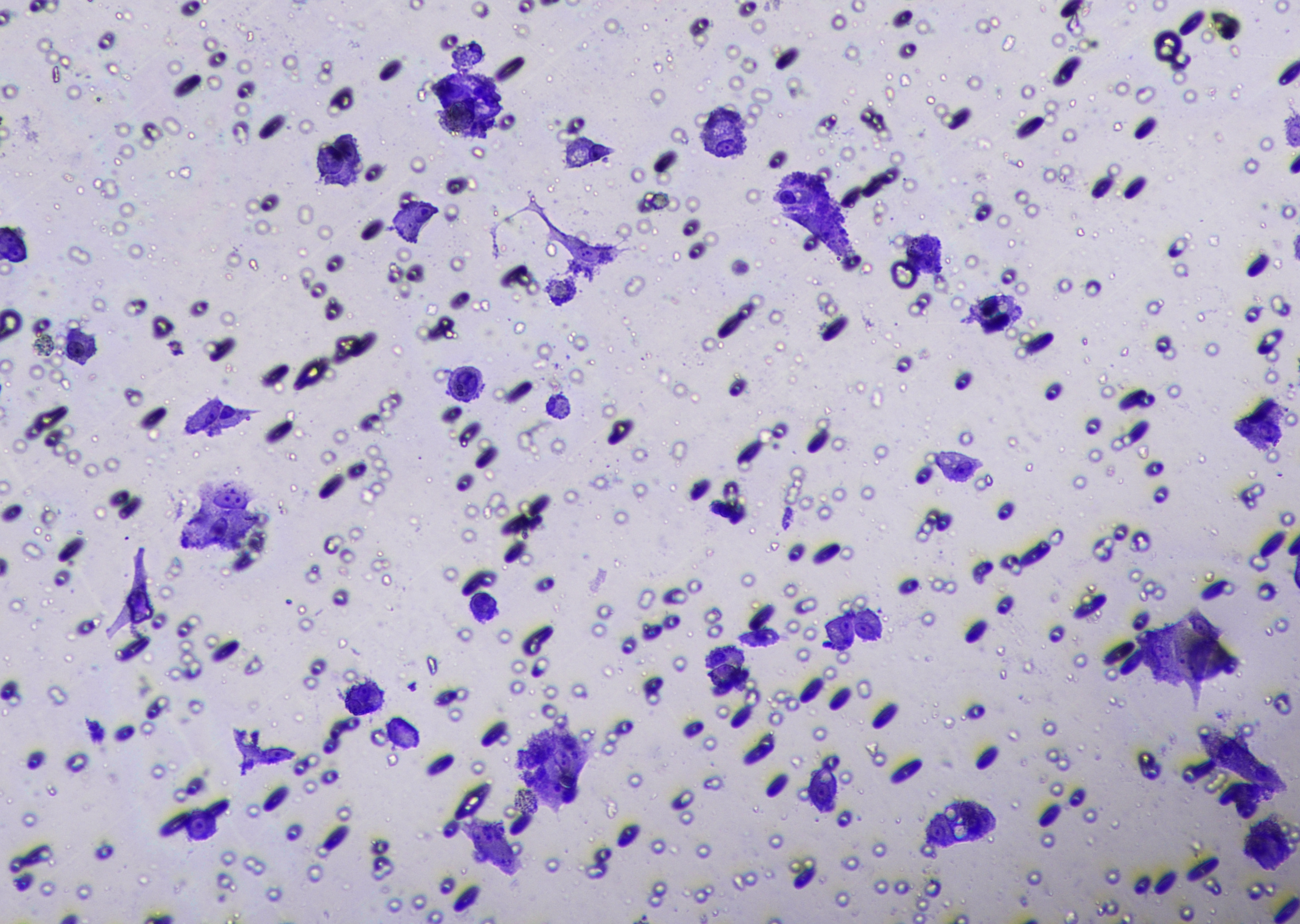

Supplement: Figure 4—source data 10. [file elife-97327-fig4-data10.zip › Figure4-Source data 10/F4G-WT.tif]

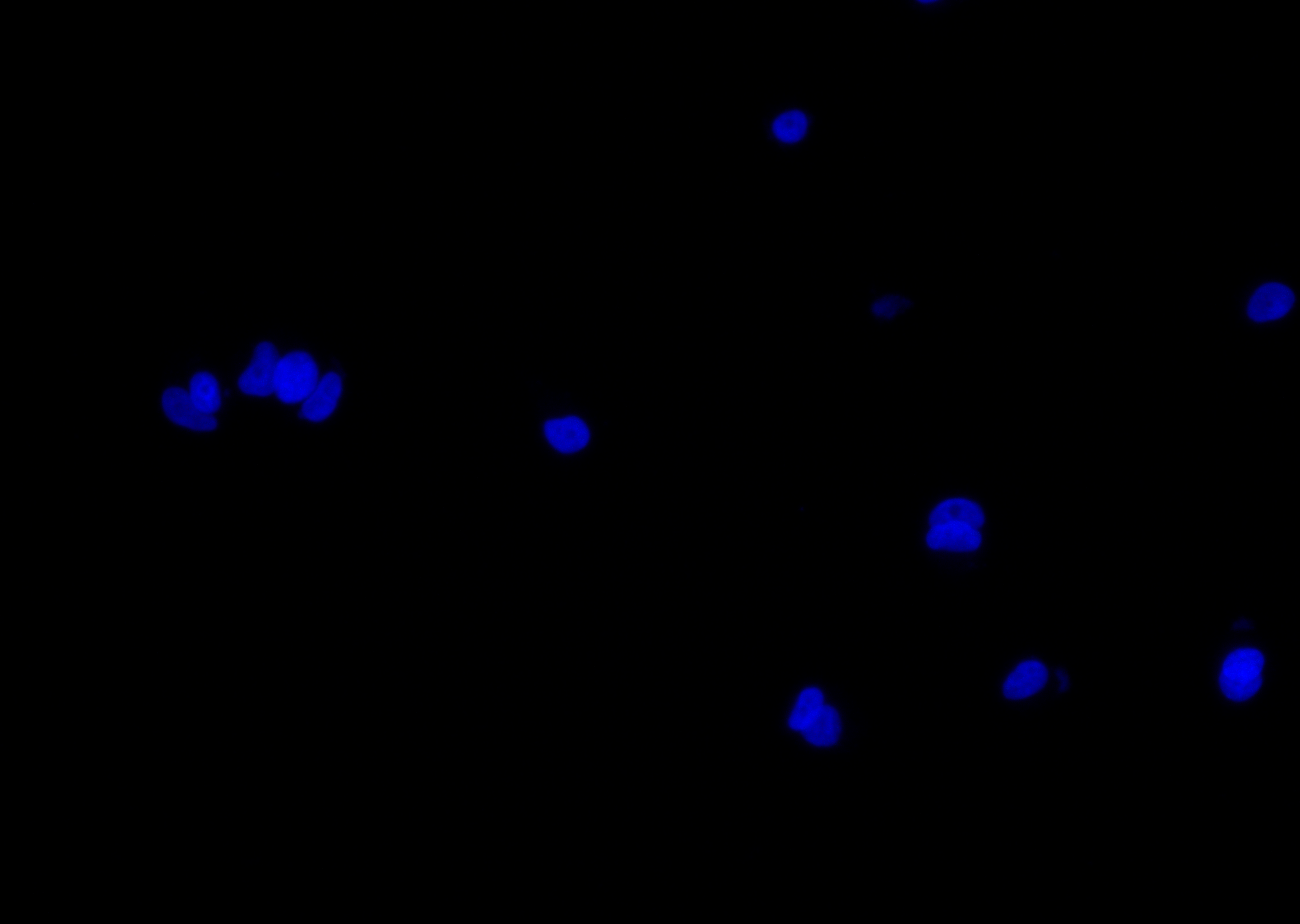

Supplement: Figure 4—source data 11. [file elife-97327-fig4-data11.zip › Figure4-Source data 11/E-cadherin-mimic-DAPI.tif]

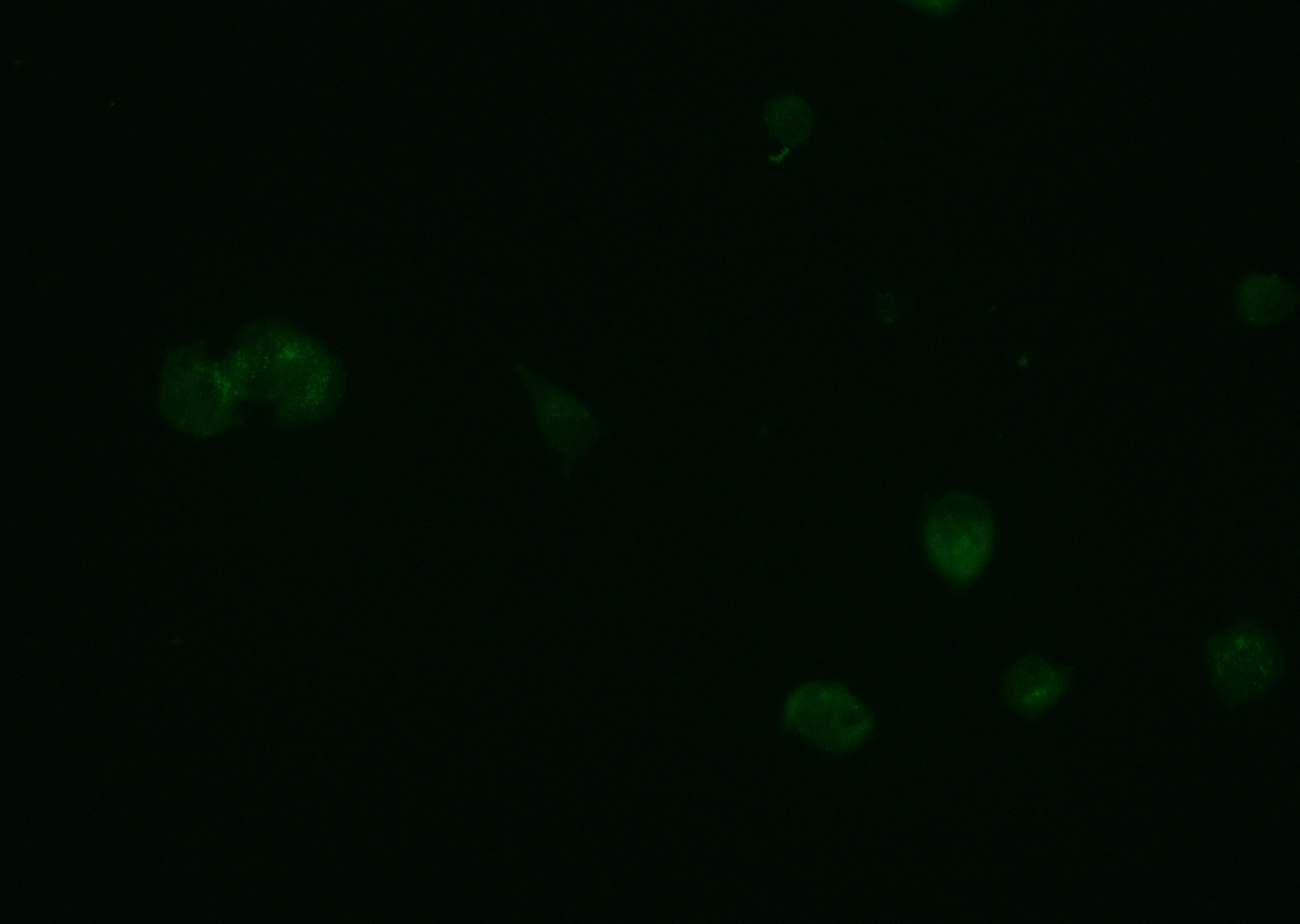

Supplement: Figure 4—source data 11. [file elife-97327-fig4-data11.zip › Figure4-Source data 11/E-cadherin-mimic-FITC.tif]

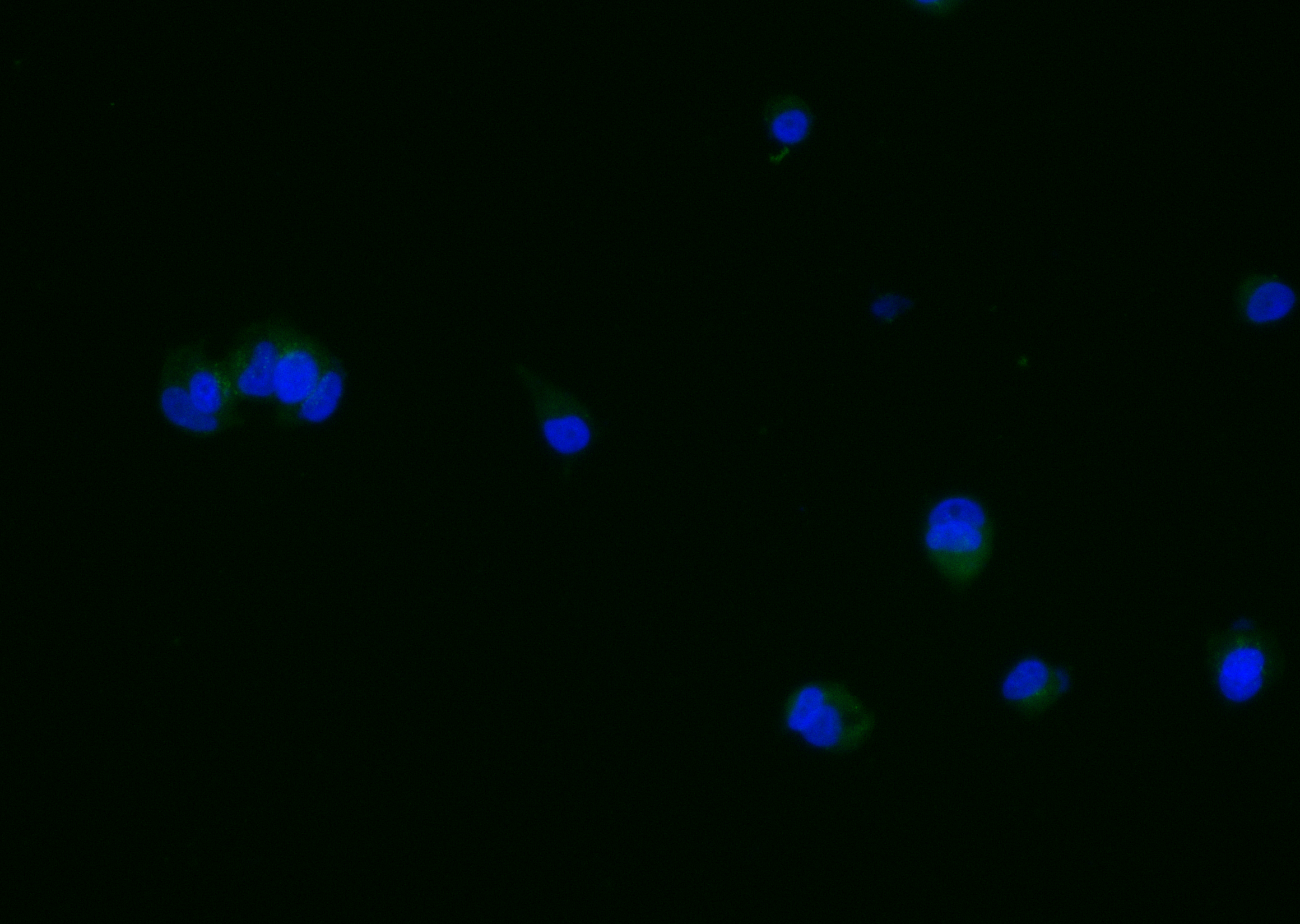

Supplement: Figure 4—source data 11. [file elife-97327-fig4-data11.zip › Figure4-Source data 11/E-cadherin-mimic-mergde.tif]
