# Supplementary figures and images for: RGS10 deficiency facilitates distant metastasis by inducing epithelial–mesenchymal transition in breast cancer (part 2 of 2)
Source: eLife. 2024 Aug 15;13:RP97327. doi: 10.7554/eLife.97327 (PMC11326775; doi:10.7554/eLife.97327)

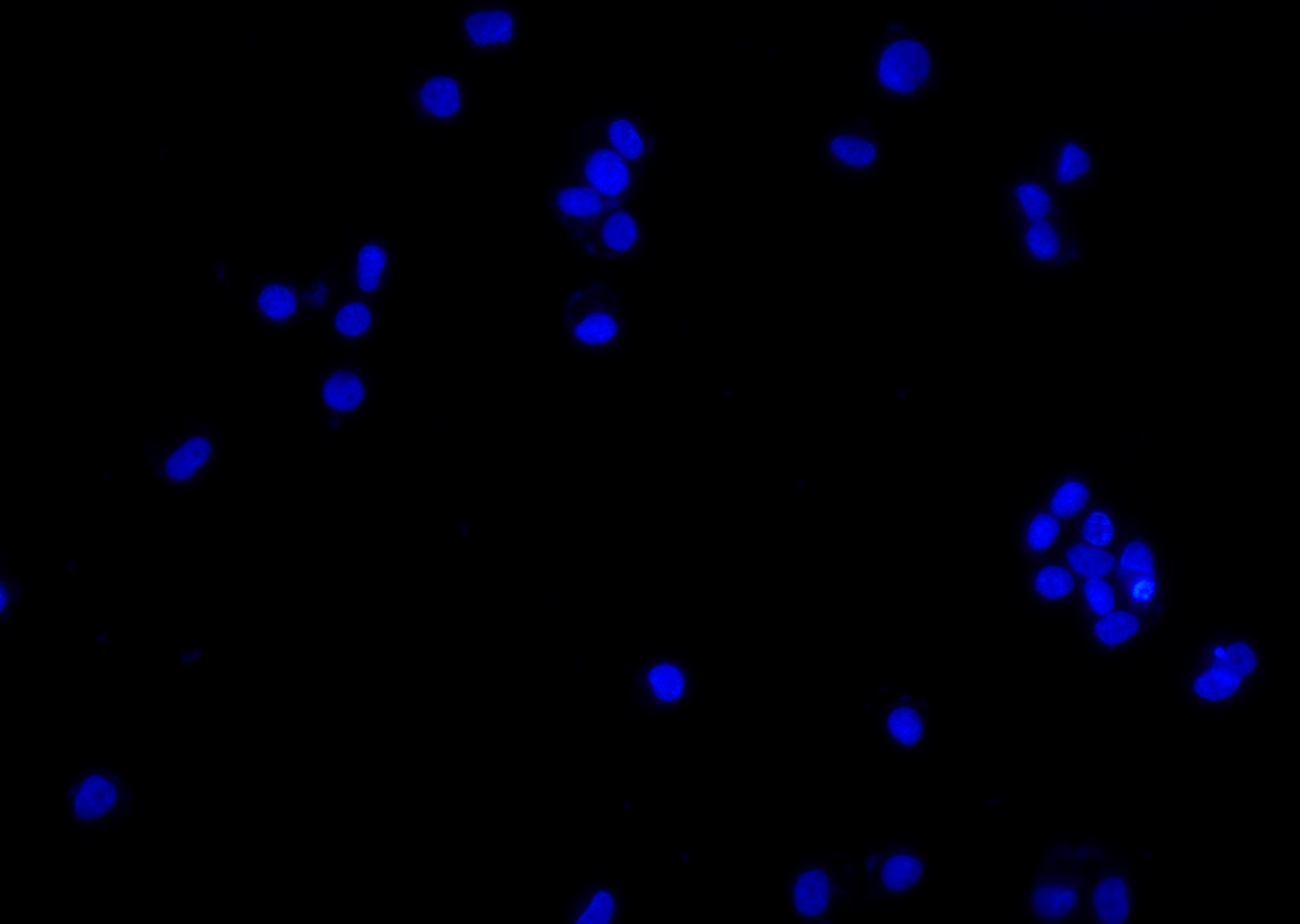

Supplement: Figure 4—source data 11. [file elife-97327-fig4-data11.zip › Figure4-Source data 11/E-cadherin-NC-DAPI.tif]

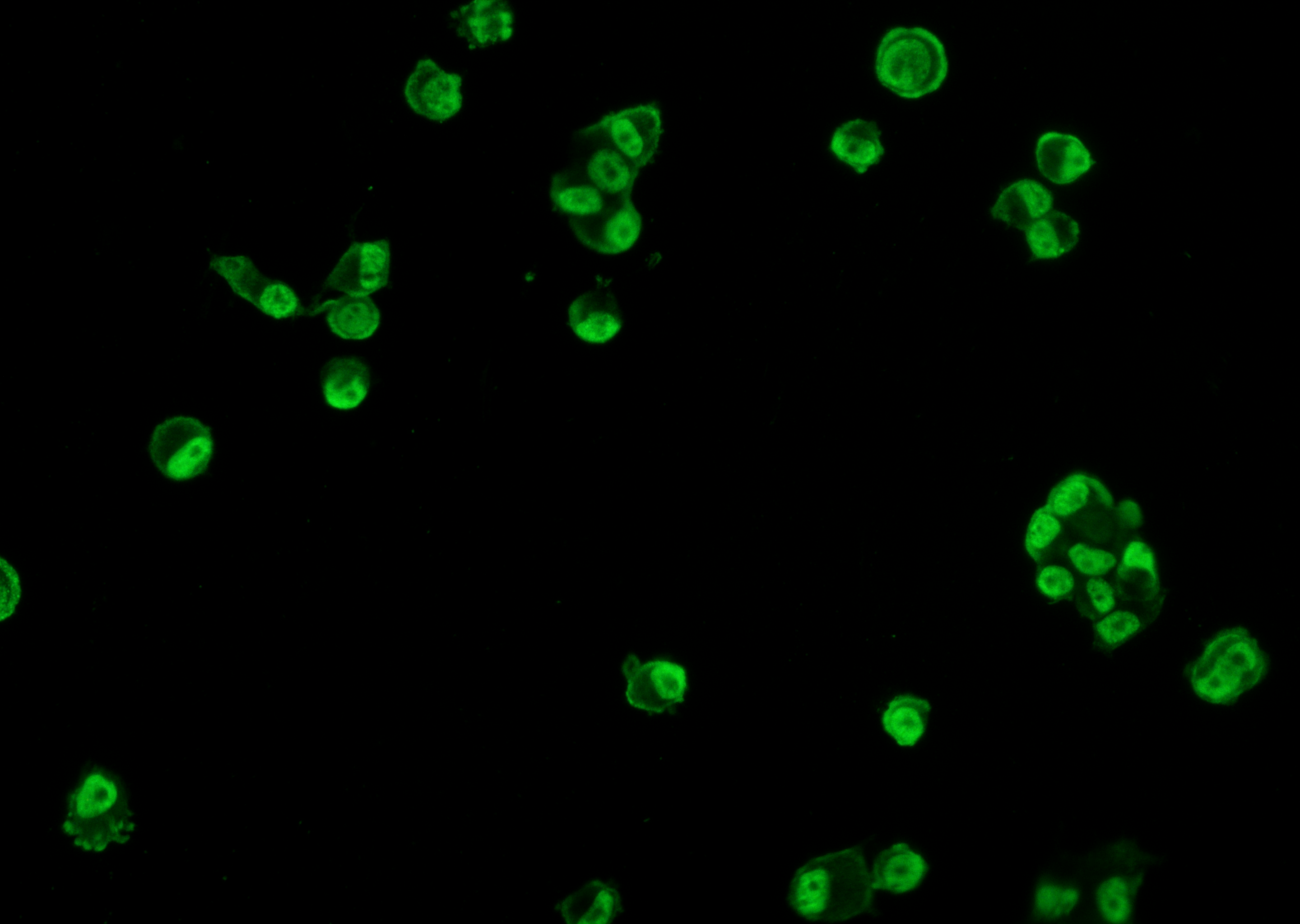

Supplement: Figure 4—source data 11. [file elife-97327-fig4-data11.zip › Figure4-Source data 11/E-cadherin-NC-FITC.tif]

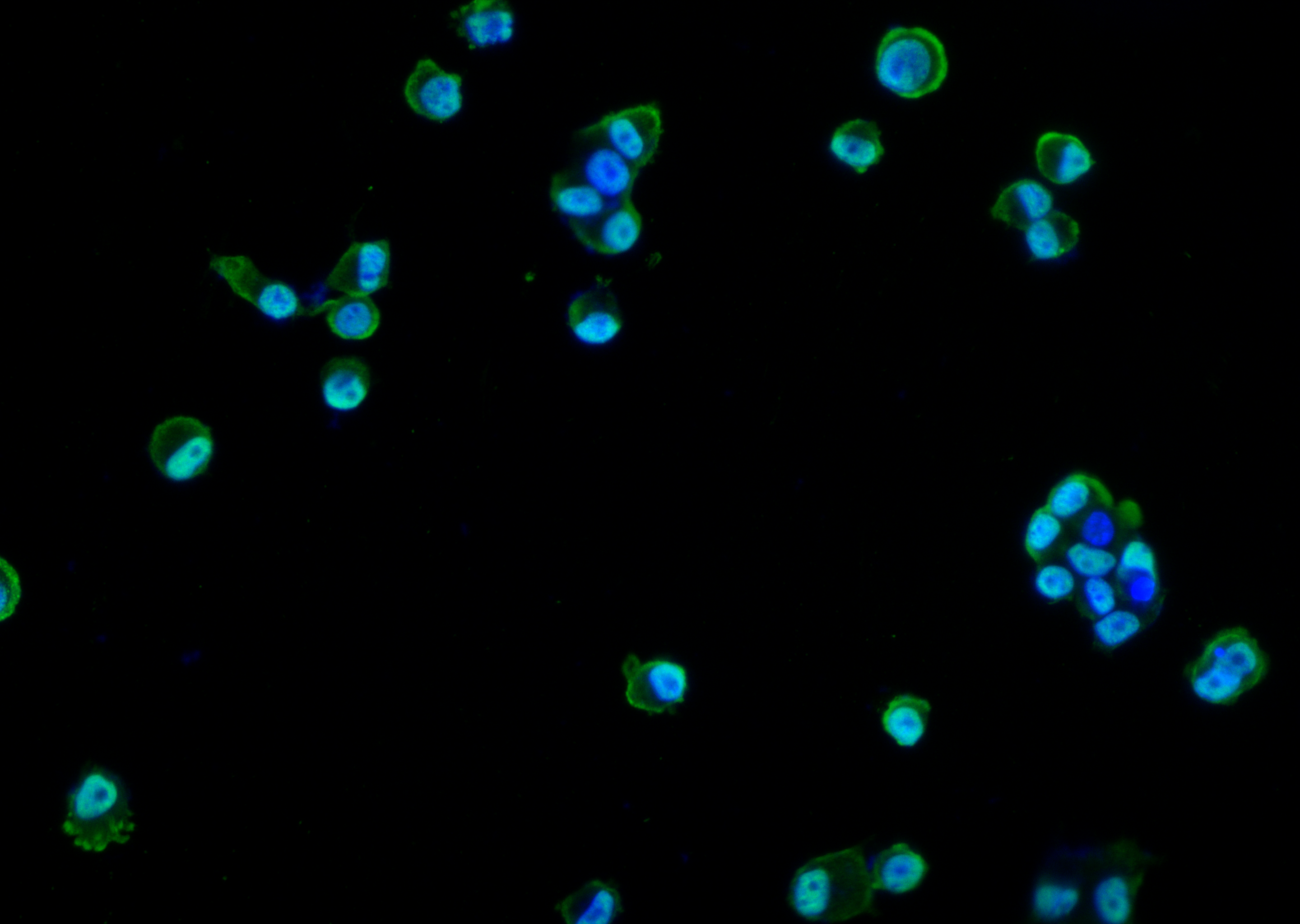

Supplement: Figure 4—source data 11. [file elife-97327-fig4-data11.zip › Figure4-Source data 11/E-cadherin-NC-merged.tif]

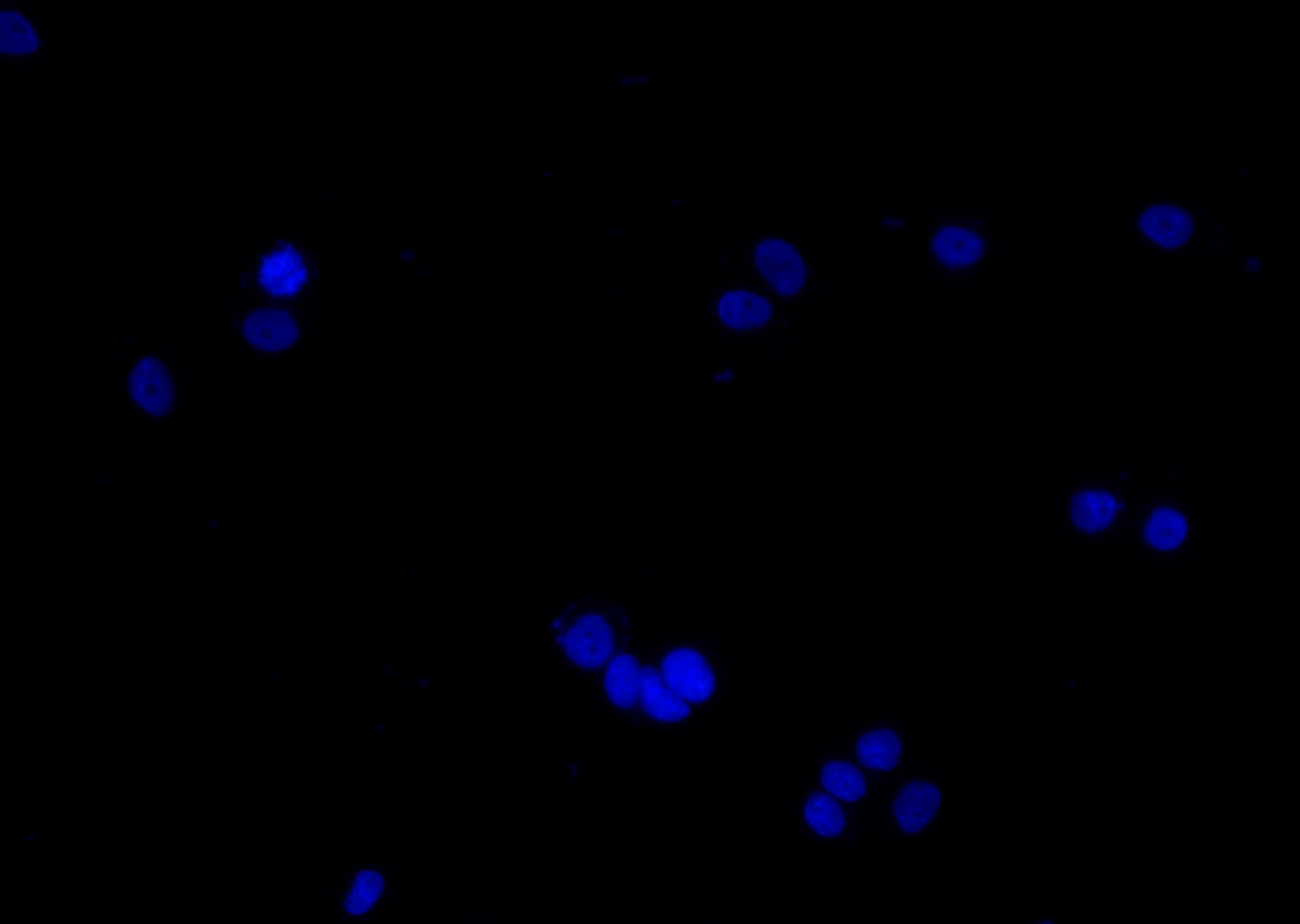

Supplement: Figure 4—source data 11. [file elife-97327-fig4-data11.zip › Figure4-Source data 11/Snail-mimic-DAPI.tif]

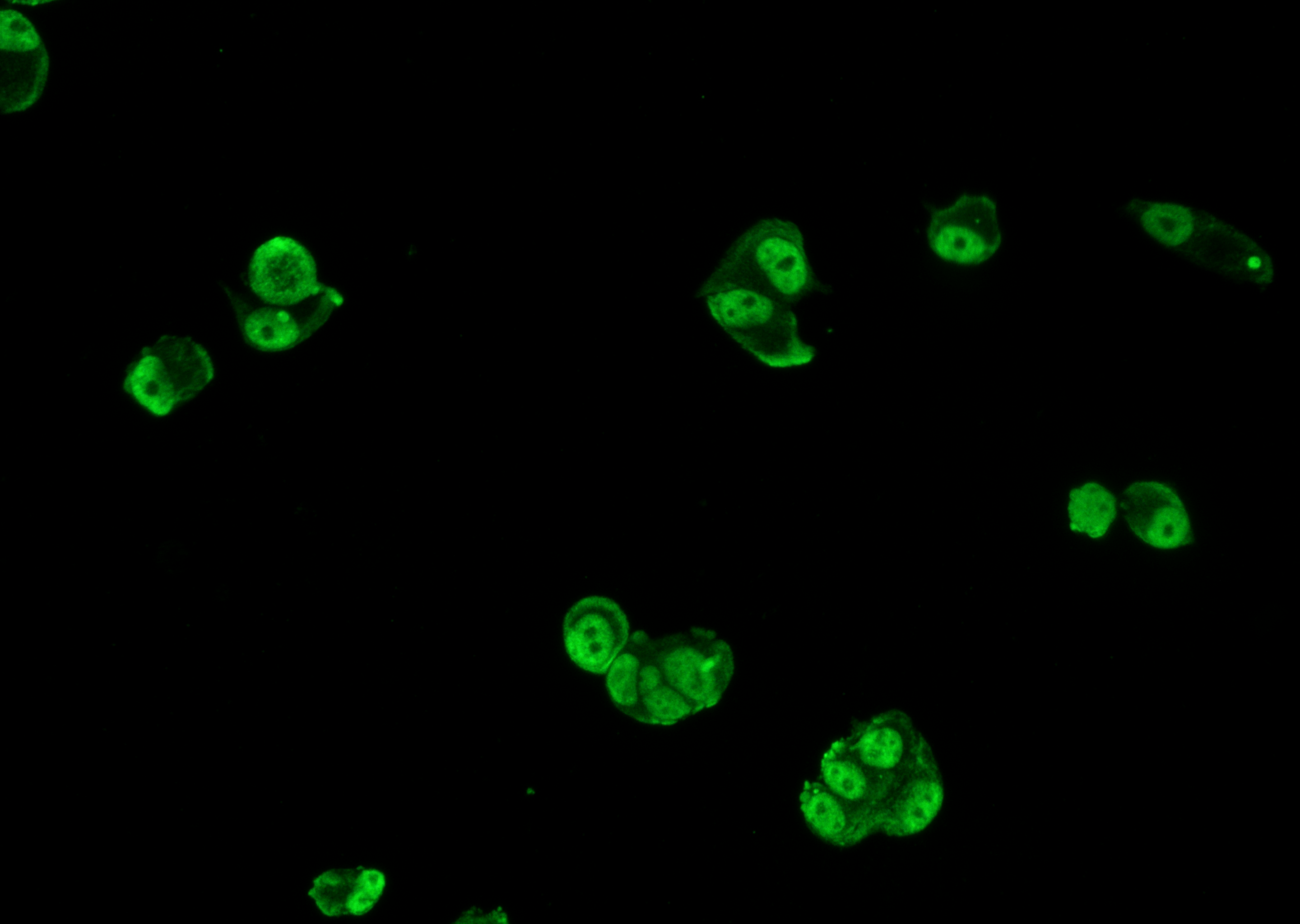

Supplement: Figure 4—source data 11. [file elife-97327-fig4-data11.zip › Figure4-Source data 11/Snail-mimic-FITC.tif]

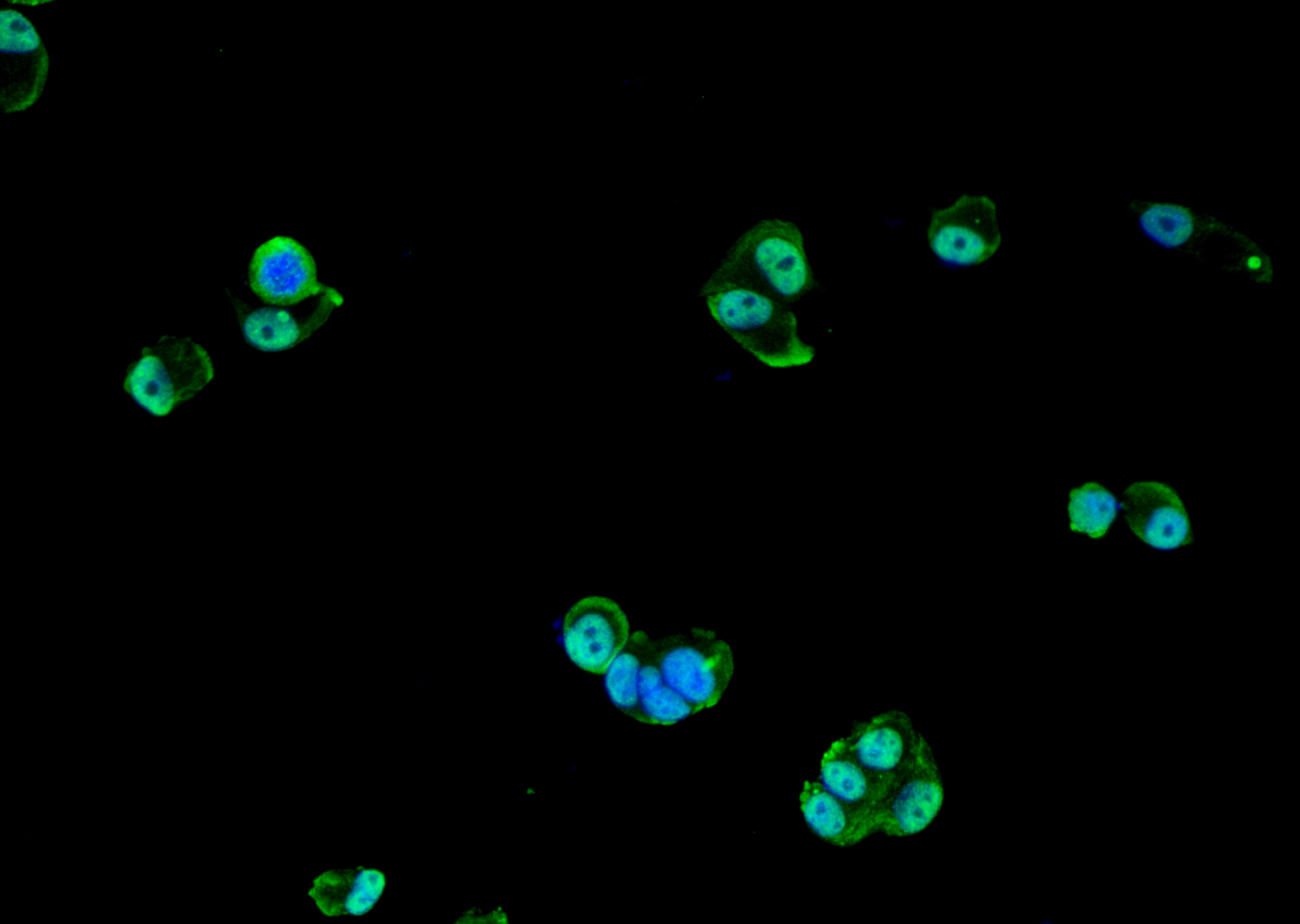

Supplement: Figure 4—source data 11. [file elife-97327-fig4-data11.zip › Figure4-Source data 11/Snail-mimic-merged.tif]

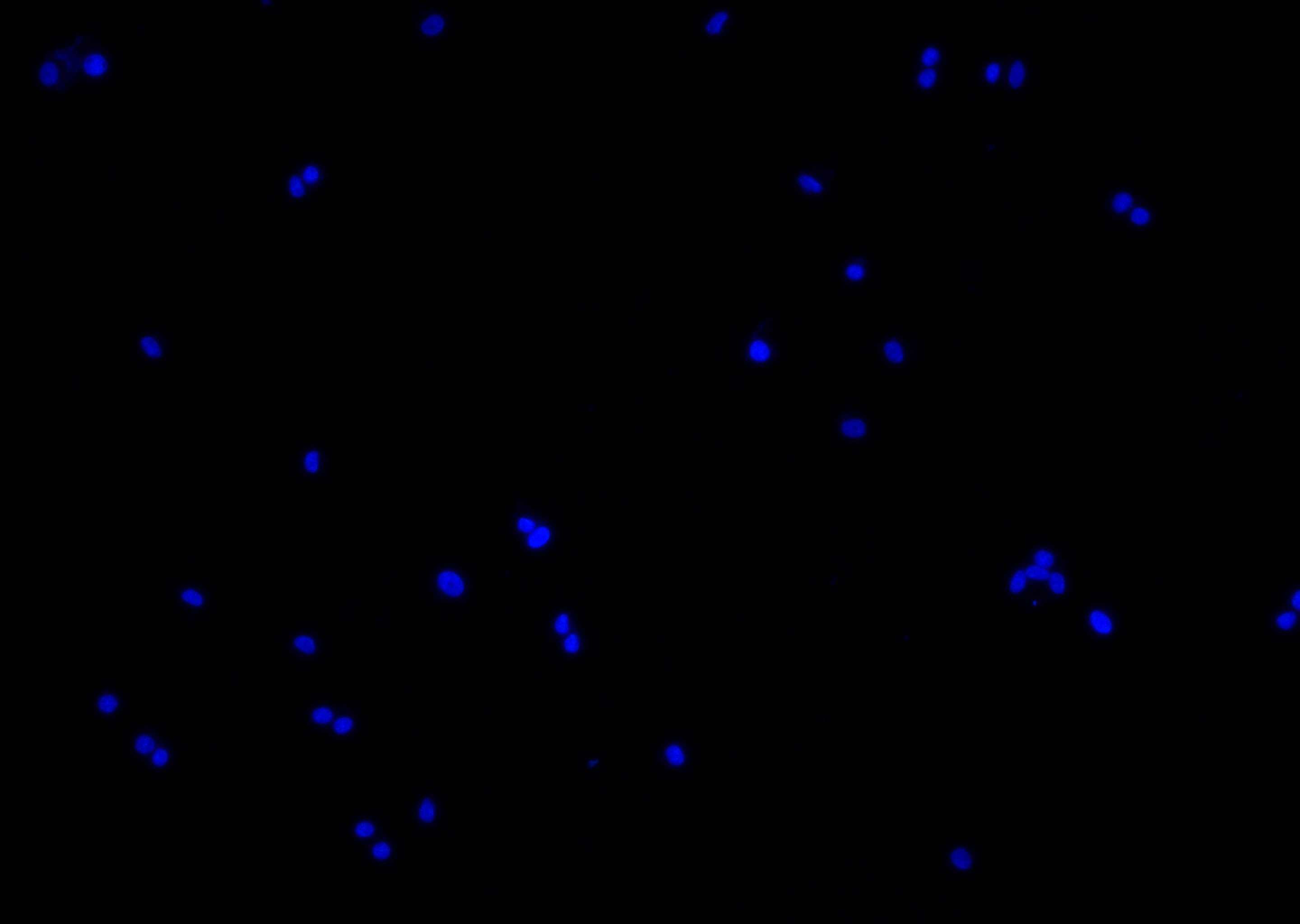

Supplement: Figure 4—source data 11. [file elife-97327-fig4-data11.zip › Figure4-Source data 11/Snail-NC-DAPI.tif]

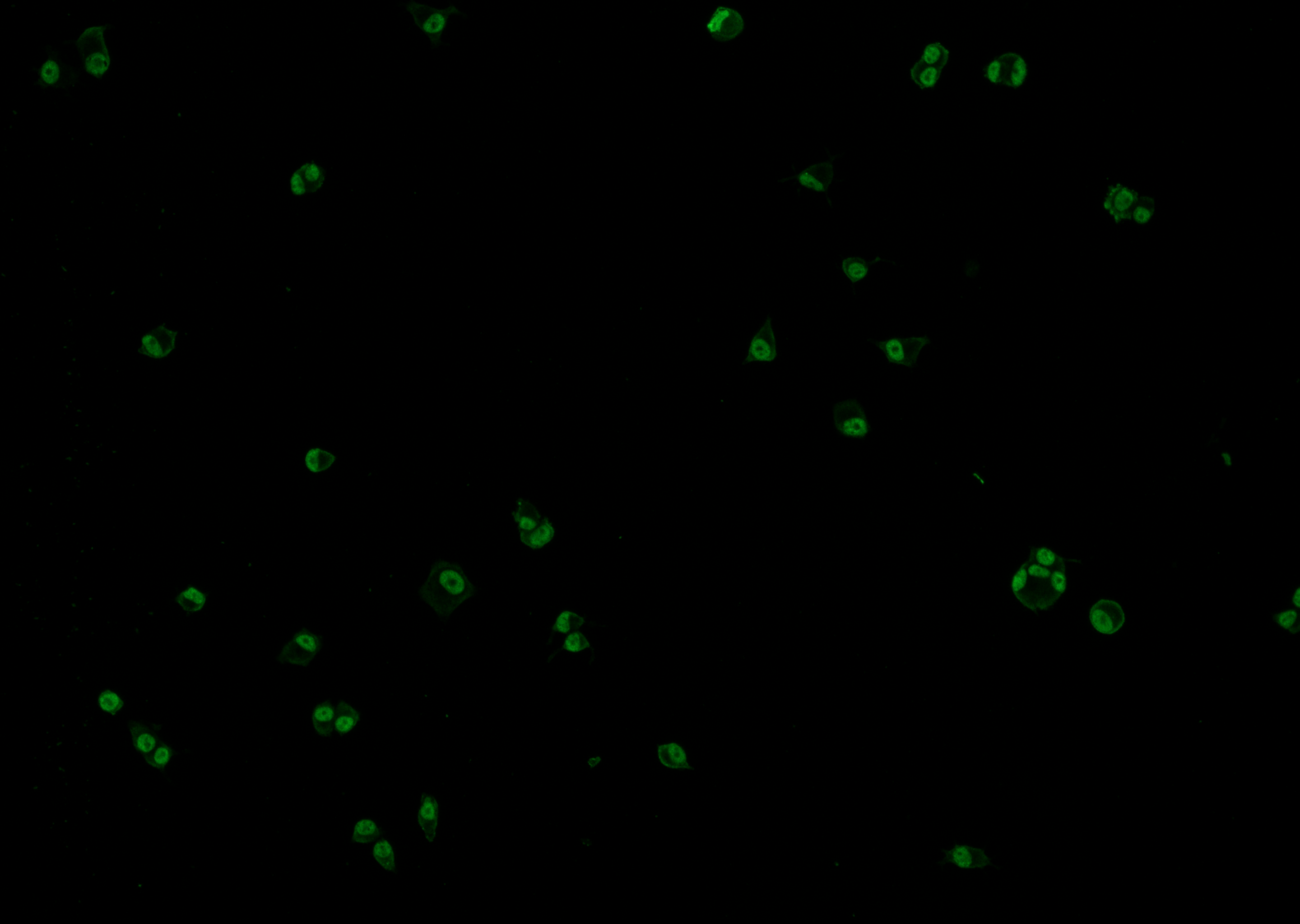

Supplement: Figure 4—source data 11. [file elife-97327-fig4-data11.zip › Figure4-Source data 11/Snail-NC-FITC.tif]

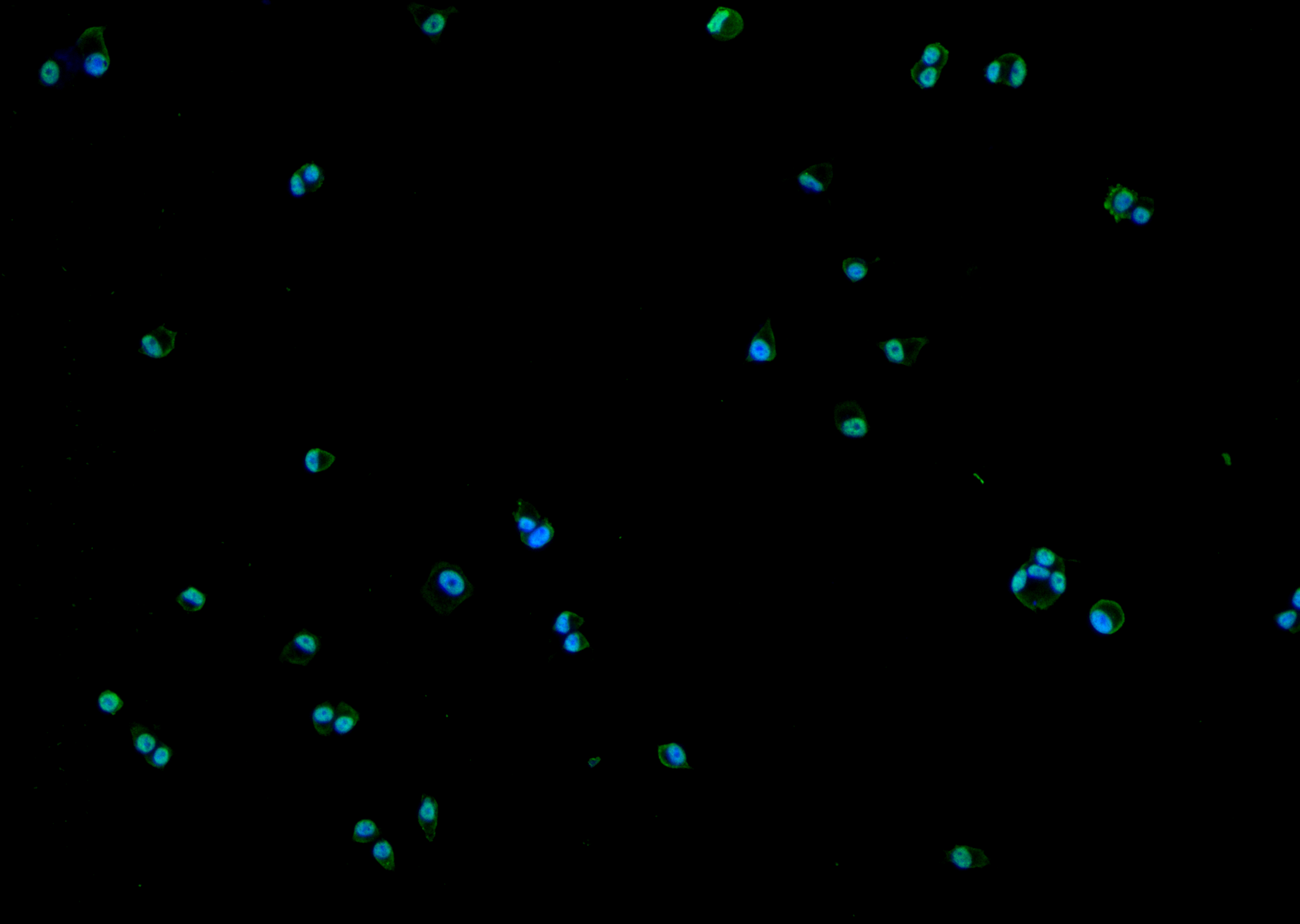

Supplement: Figure 4—source data 11. [file elife-97327-fig4-data11.zip › Figure4-Source data 11/Snail-NC-merged.tif]

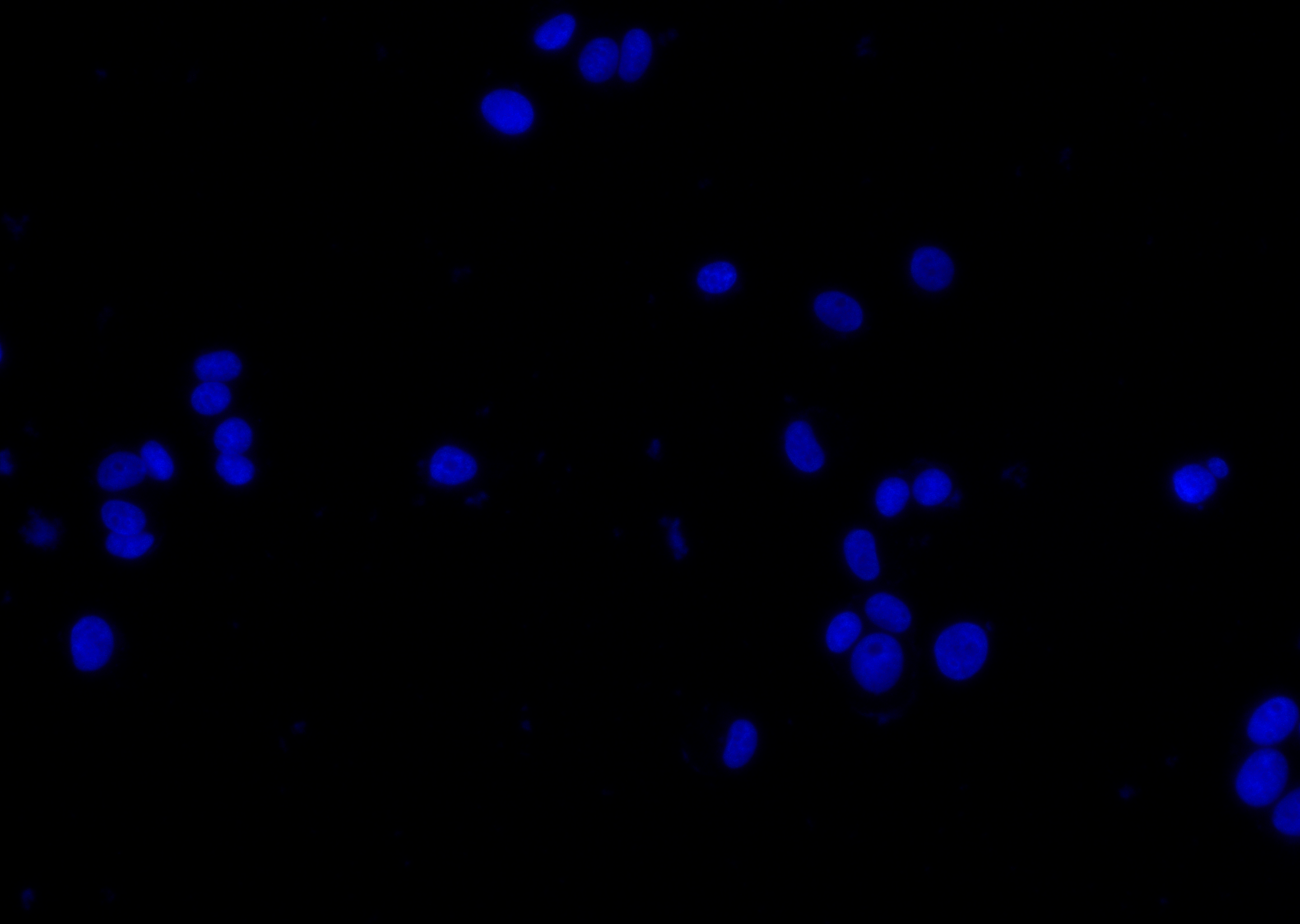

Supplement: Figure 4—source data 11. [file elife-97327-fig4-data11.zip › Figure4-Source data 11/Vimentin-mimic-DAPI.tif]

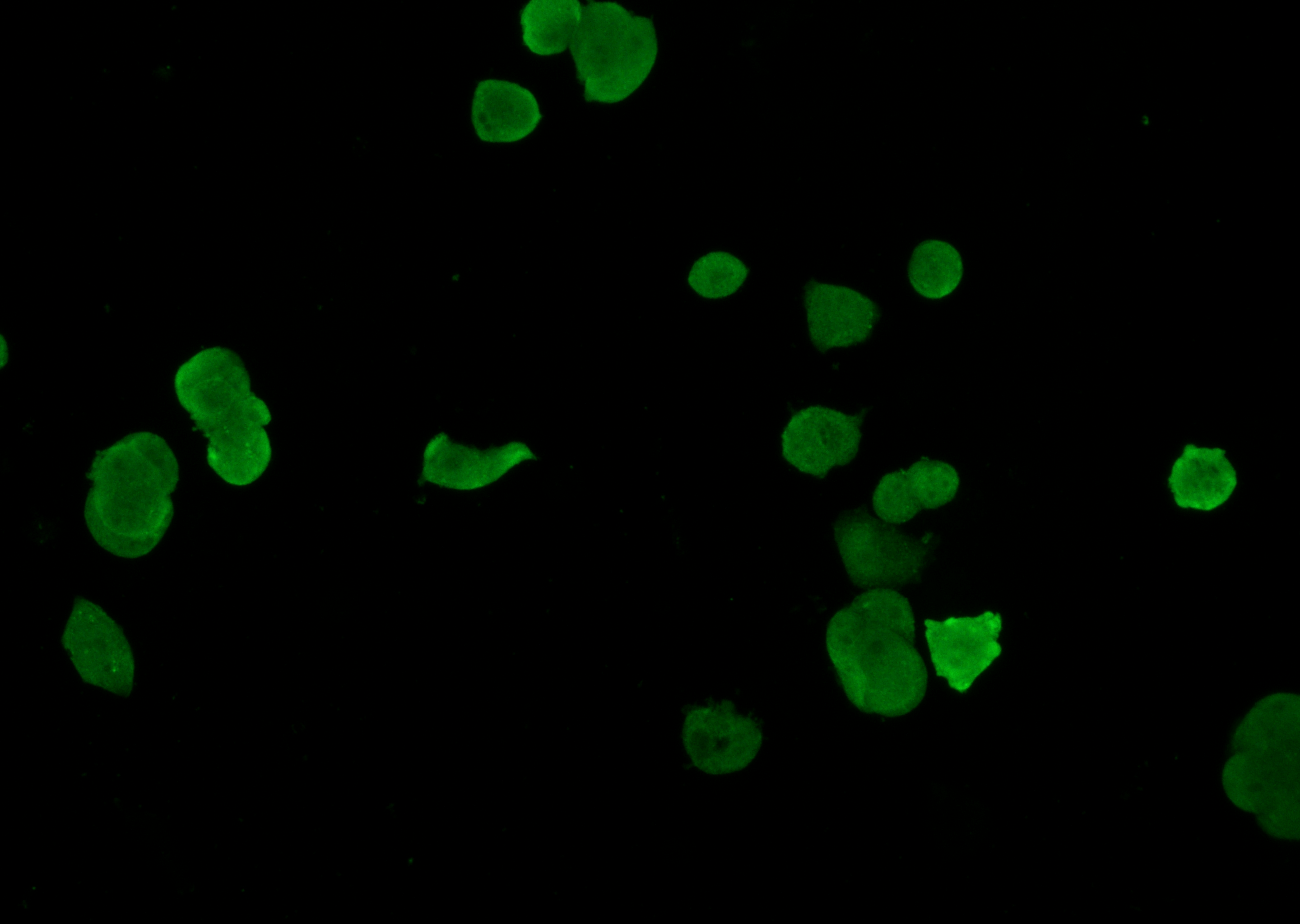

Supplement: Figure 4—source data 11. [file elife-97327-fig4-data11.zip › Figure4-Source data 11/Vimentin-mimic-FITC.tif]

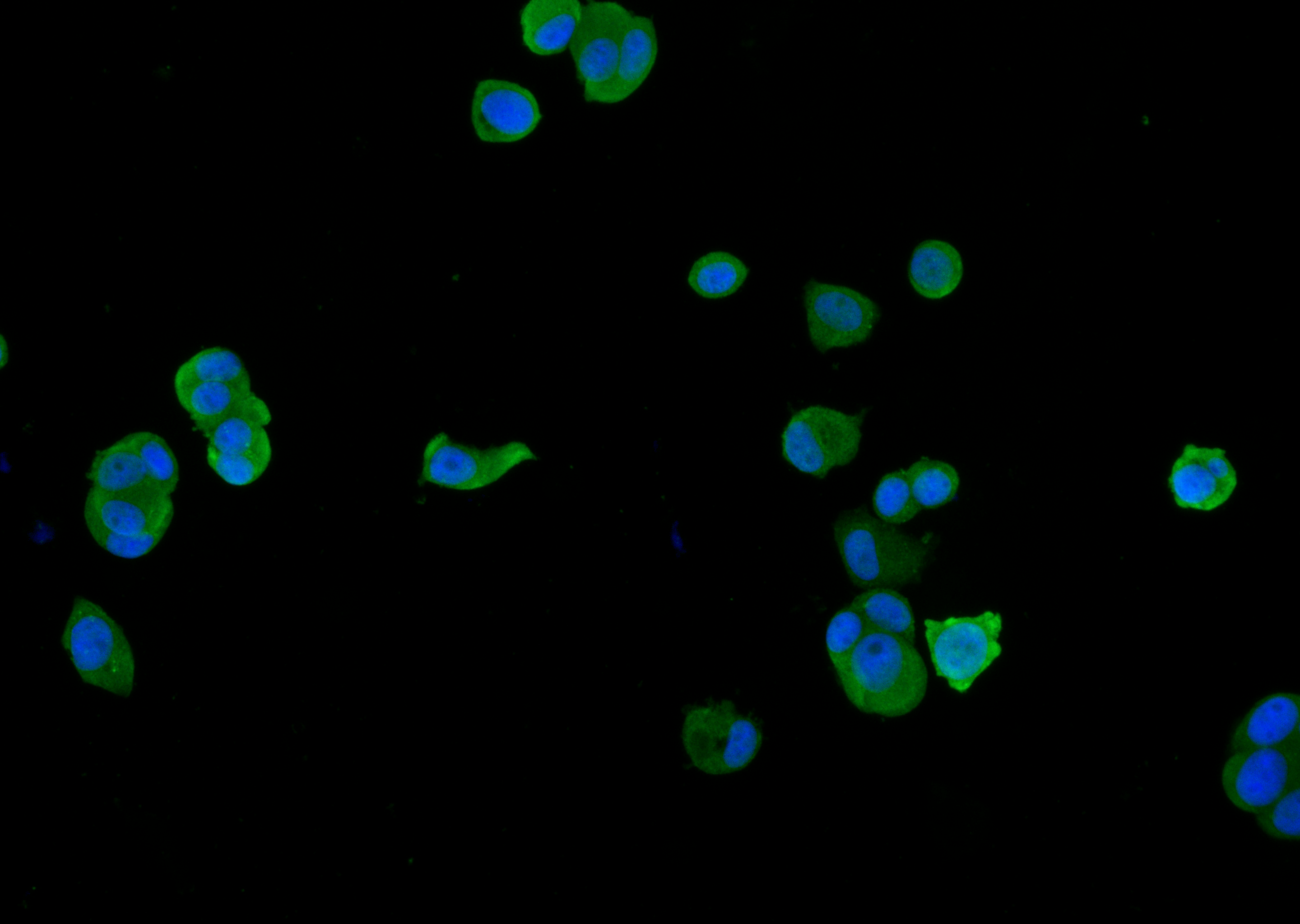

Supplement: Figure 4—source data 11. [file elife-97327-fig4-data11.zip › Figure4-Source data 11/Vimentin-mimic-merged.tif]

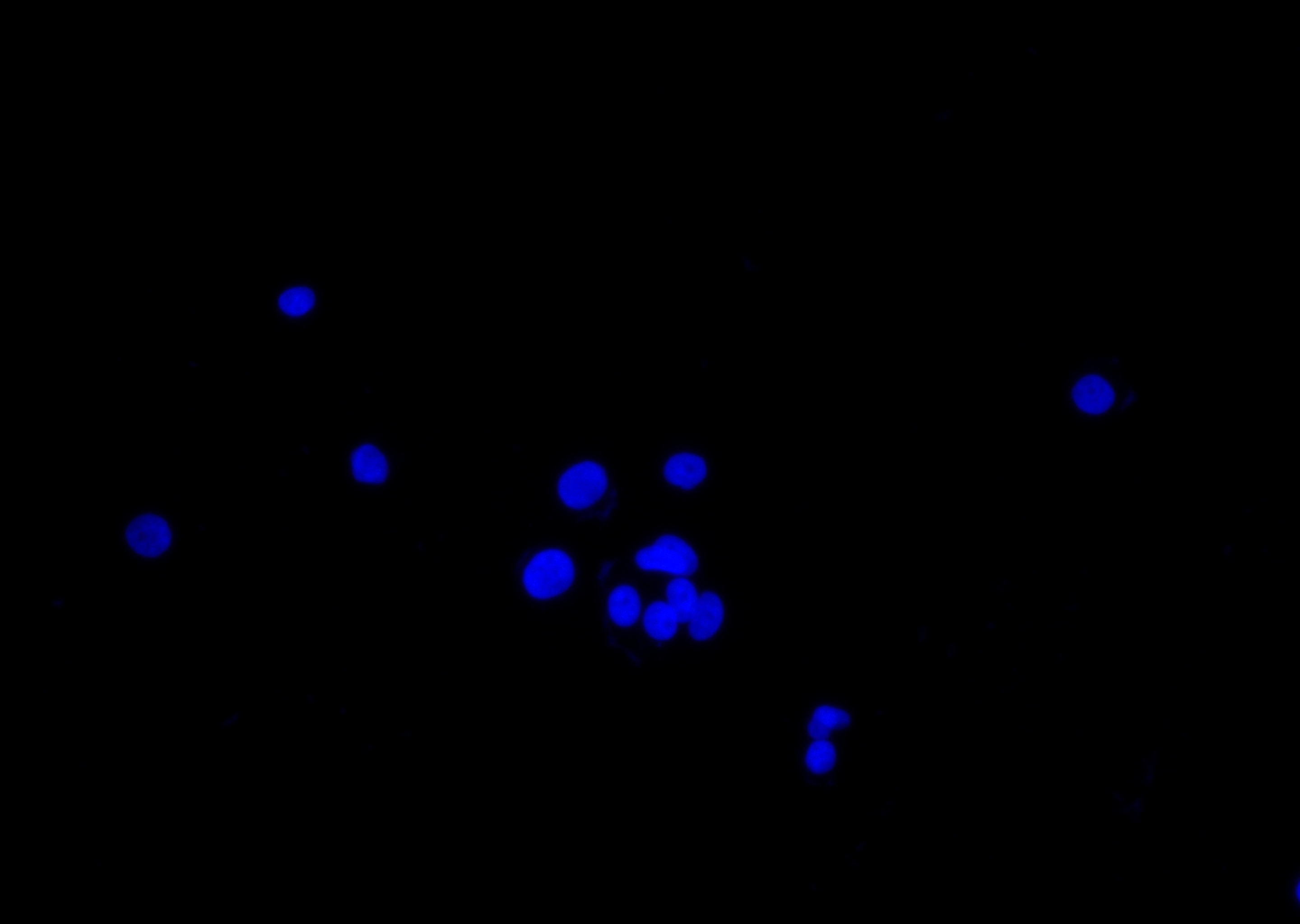

Supplement: Figure 4—source data 11. [file elife-97327-fig4-data11.zip › Figure4-Source data 11/Vimentin-NC-DAPI.tif]

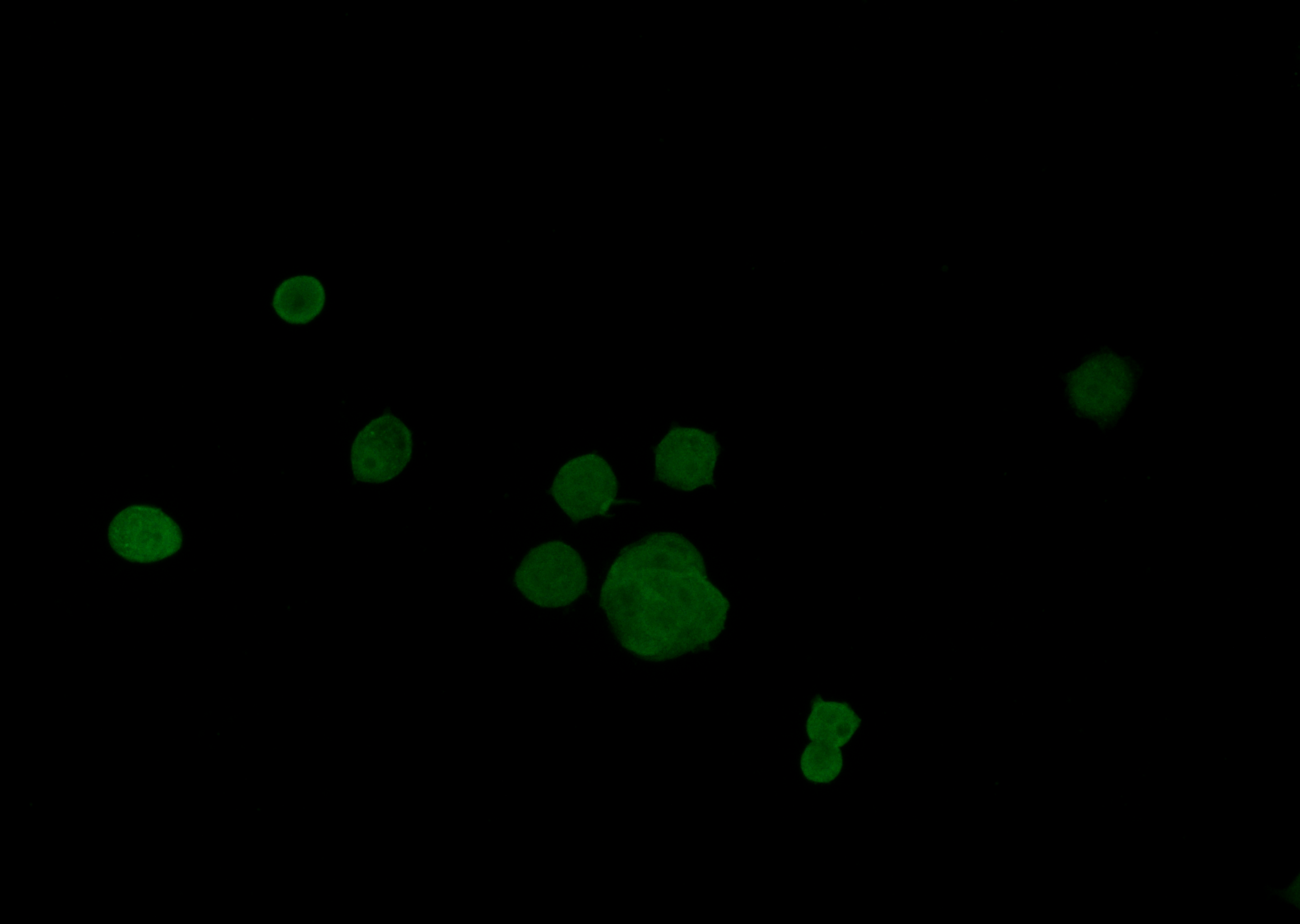

Supplement: Figure 4—source data 11. [file elife-97327-fig4-data11.zip › Figure4-Source data 11/Vimentin-NC-FITC.tif]

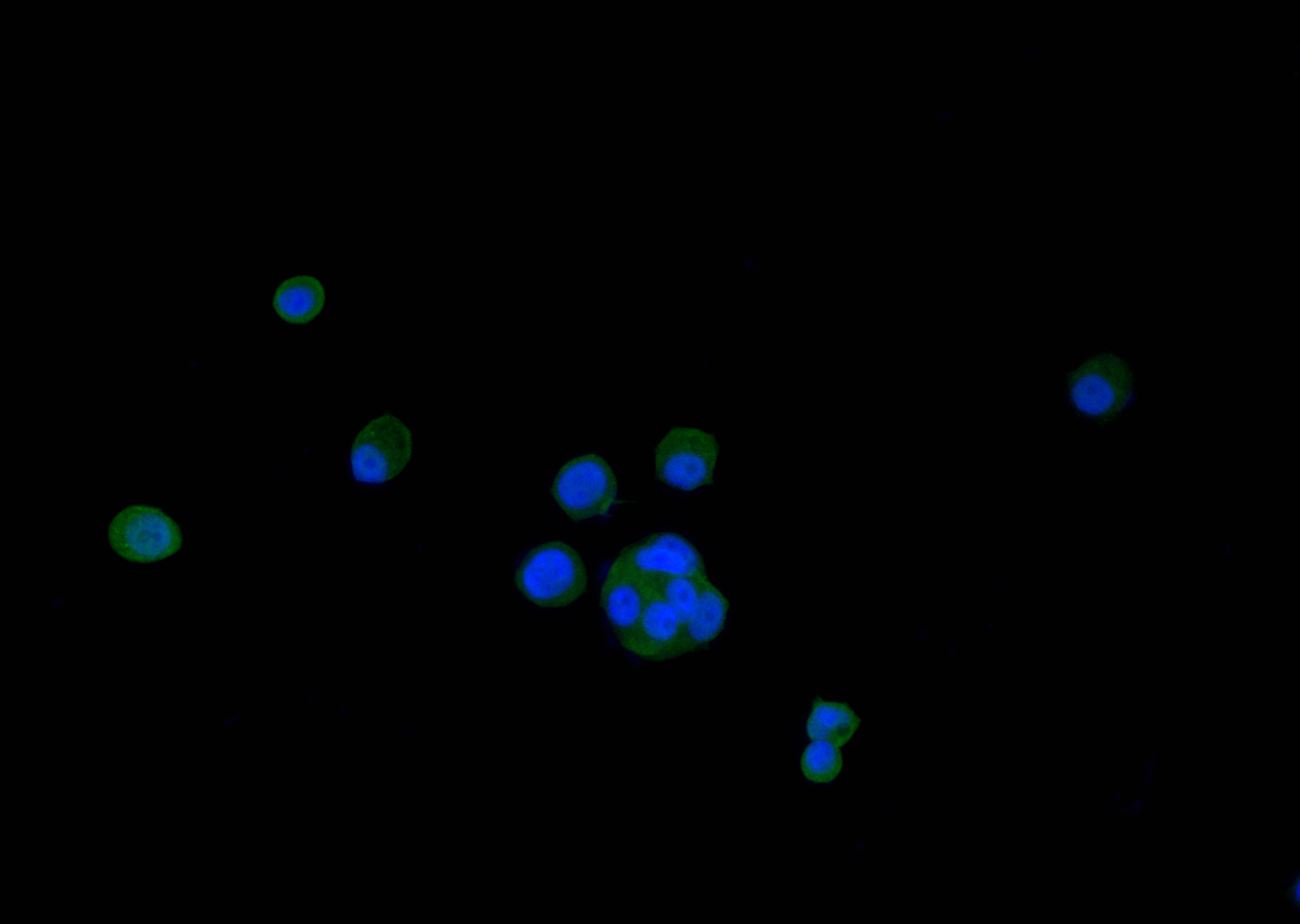

Supplement: Figure 4—source data 11. [file elife-97327-fig4-data11.zip › Figure4-Source data 11/Vimentin-NC-merged.tif]

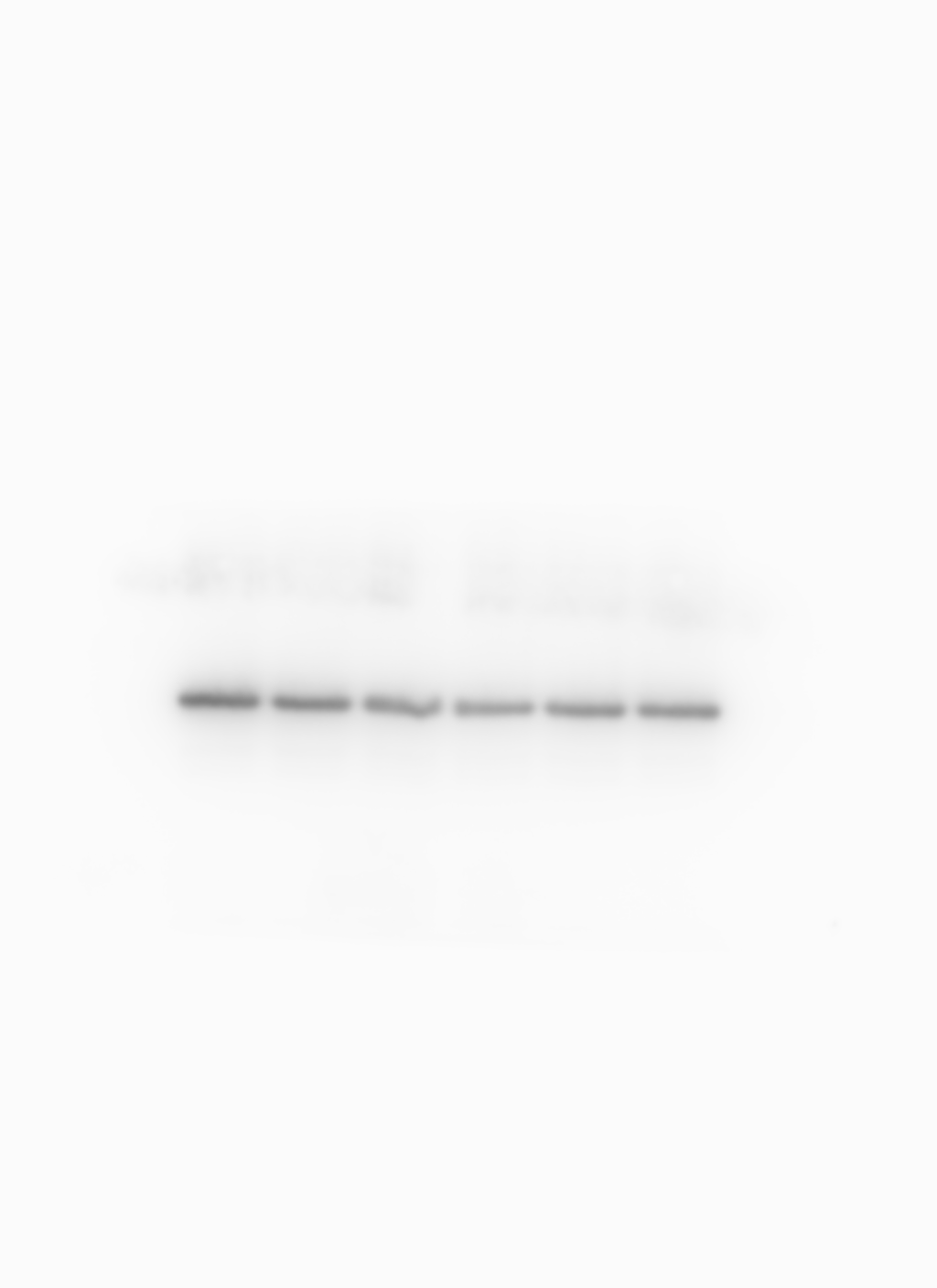

Supplement: Figure 5—source data 1. [file elife-97327-fig5-data1.zip › Figure 5-Source data 1/F5C-GAPDH .tif]

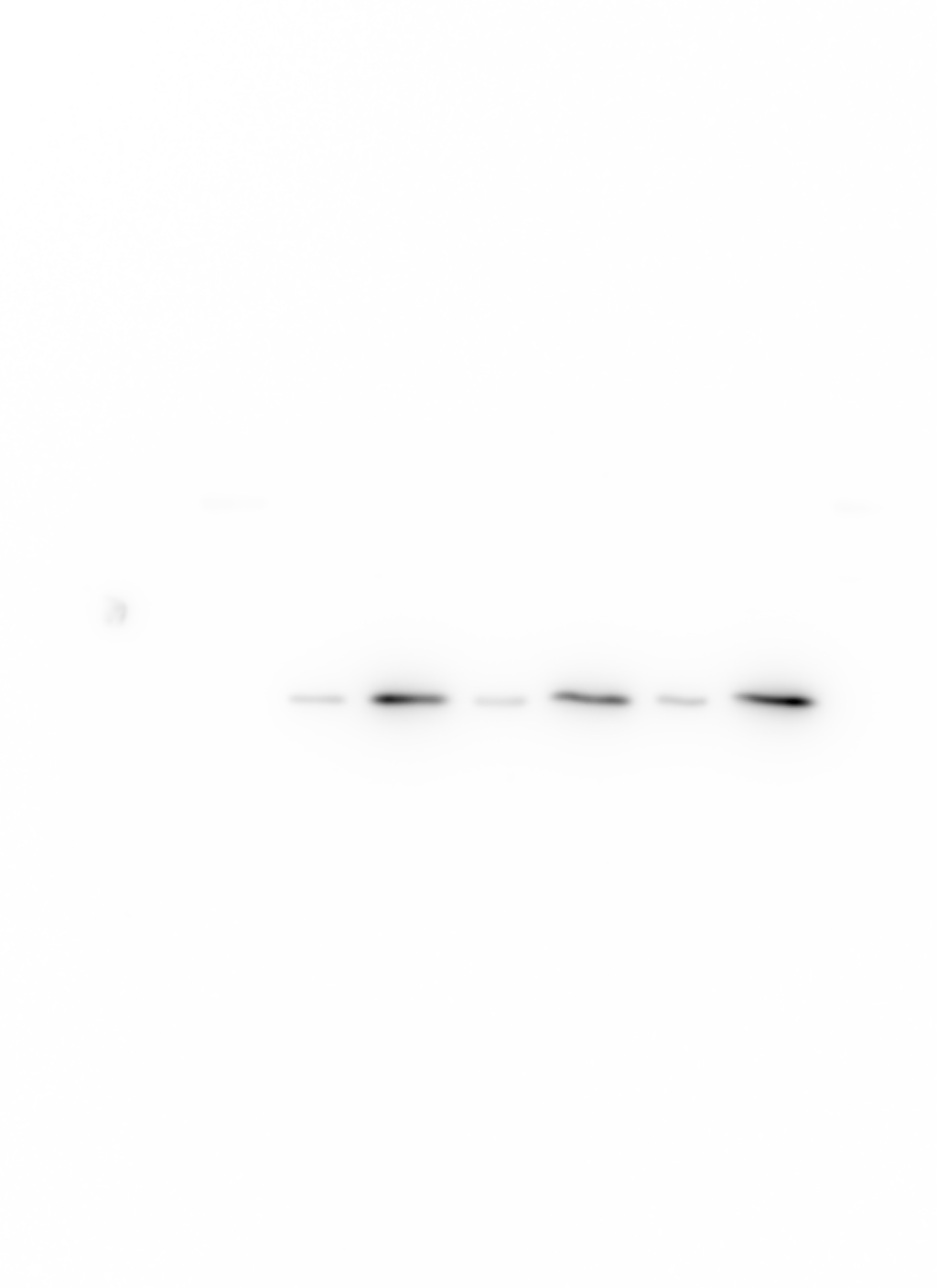

Supplement: Figure 5—source data 1. [file elife-97327-fig5-data1.zip › Figure 5-Source data 1/F5C-RGS10 .tif]

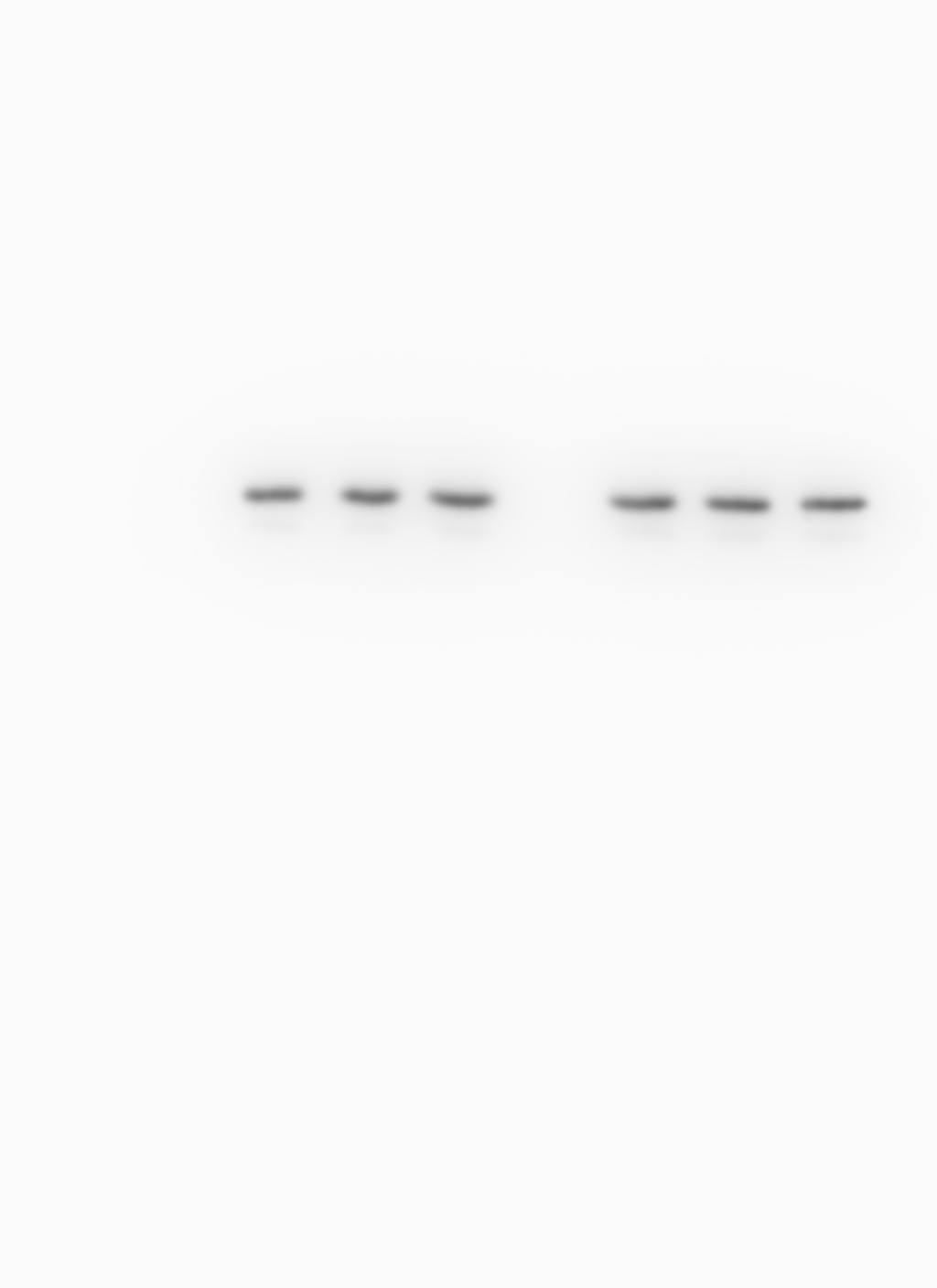

Supplement: Figure 5—source data 1. [file elife-97327-fig5-data1.zip › Figure 5-Source data 1/GAPDH.tif]

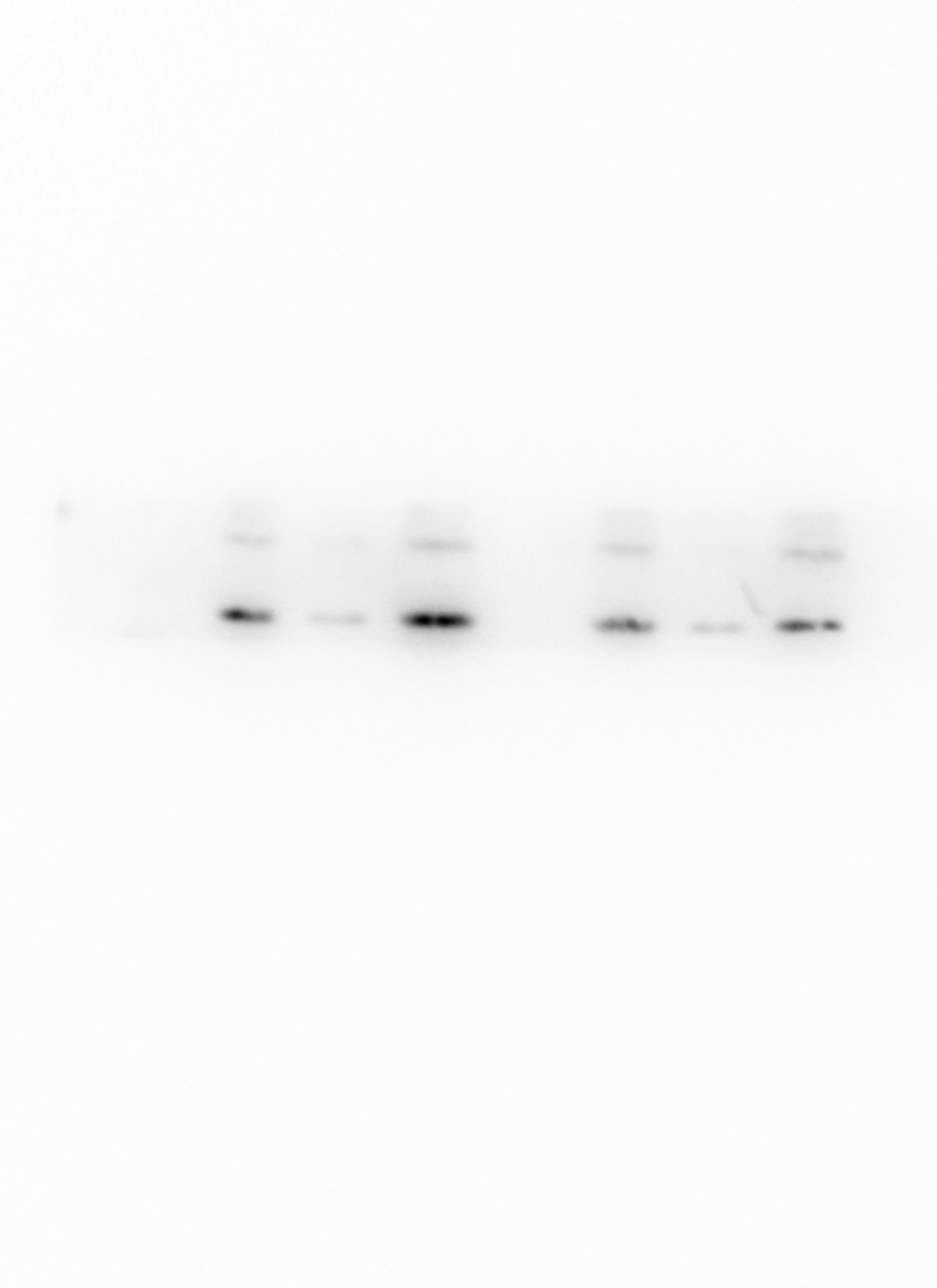

Supplement: Figure 5—source data 1. [file elife-97327-fig5-data1.zip › Figure 5-Source data 1/RGS10.tif]

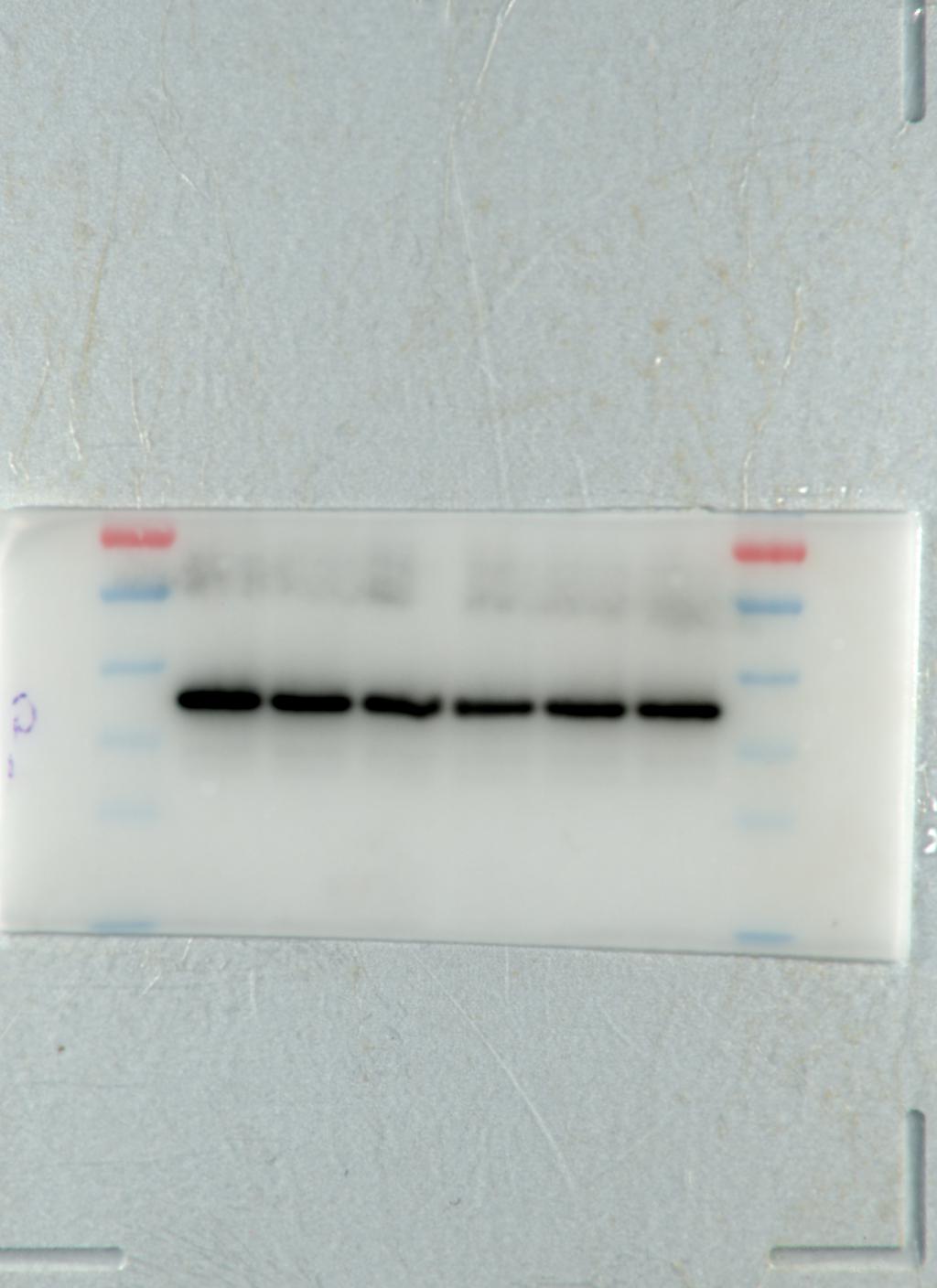

Supplement: Figure 5—source data 2. [file elife-97327-fig5-data2.zip › Figure 5-Source data 2/F5C-GAPDH+Marker.jpg]

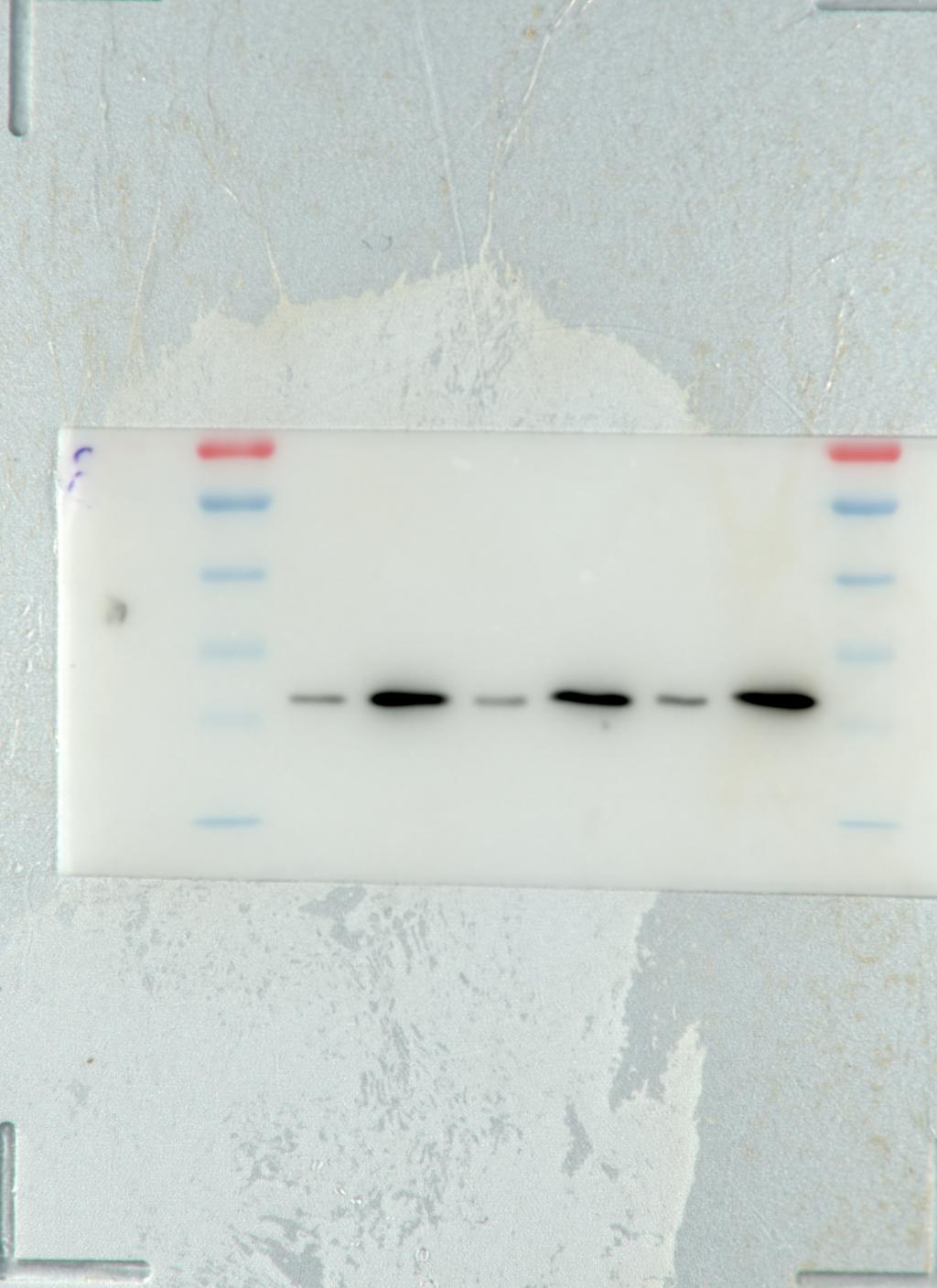

Supplement: Figure 5—source data 2. [file elife-97327-fig5-data2.zip › Figure 5-Source data 2/F5C-RGS10 +Marker.jpg]

The expression of RGS10/GAPDH

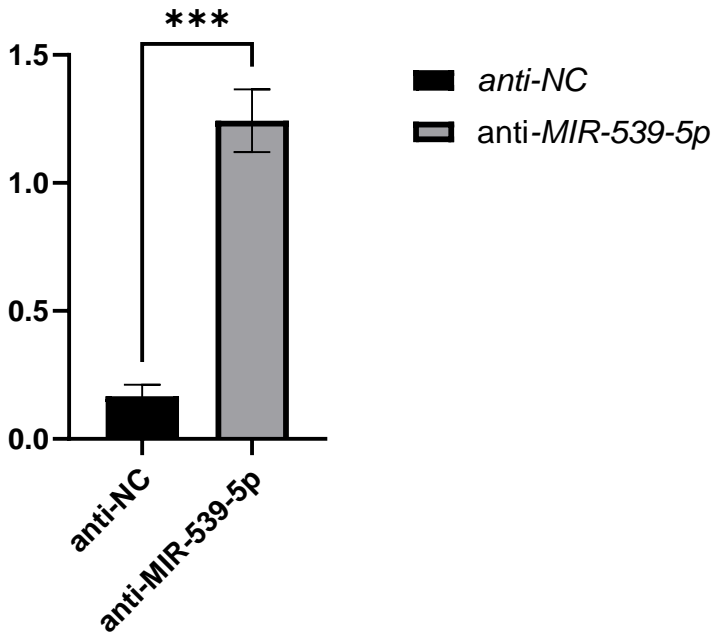

Supplement: Figure 5—source data 3. [file elife-97327-fig5-data3.pdf]

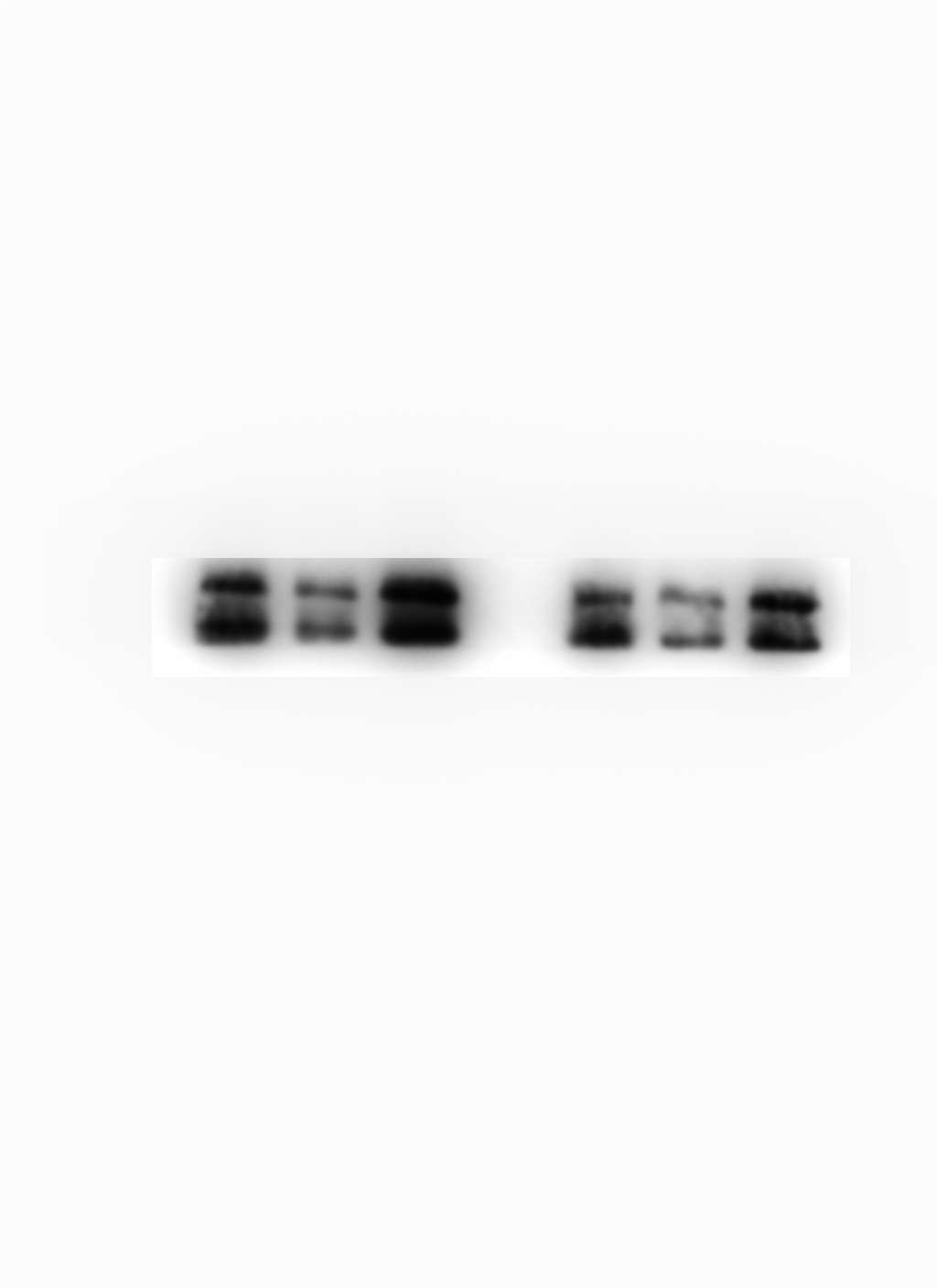

Supplement: Figure 5—source data 4. [file elife-97327-fig5-data4.zip › Figure 5-Source data 4/E-cadherin.tif]

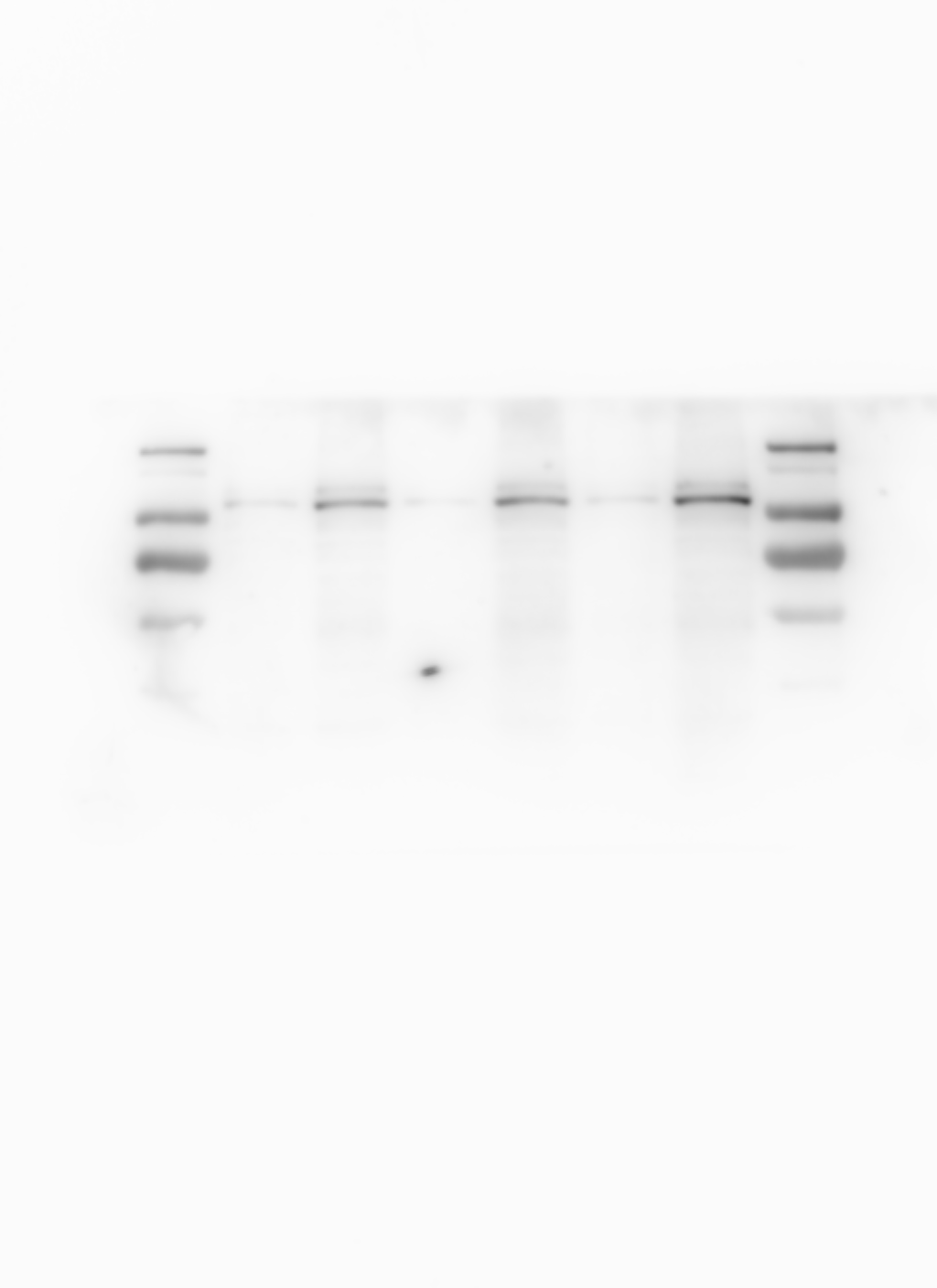

Supplement: Figure 5—source data 4. [file elife-97327-fig5-data4.zip › Figure 5-Source data 4/F5H-Eca .tif]

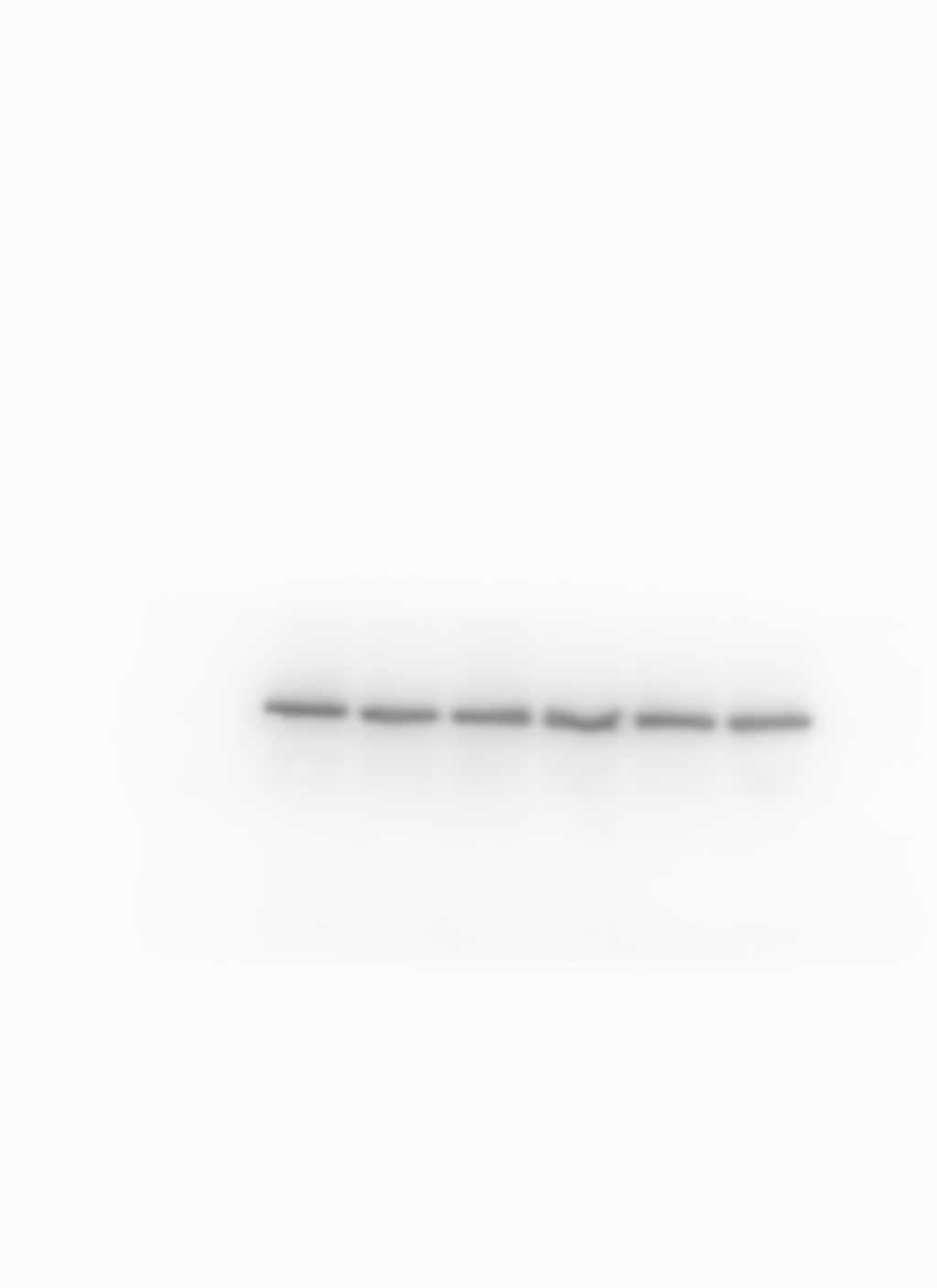

Supplement: Figure 5—source data 4. [file elife-97327-fig5-data4.zip › Figure 5-Source data 4/F5H-GAPDH .tif]

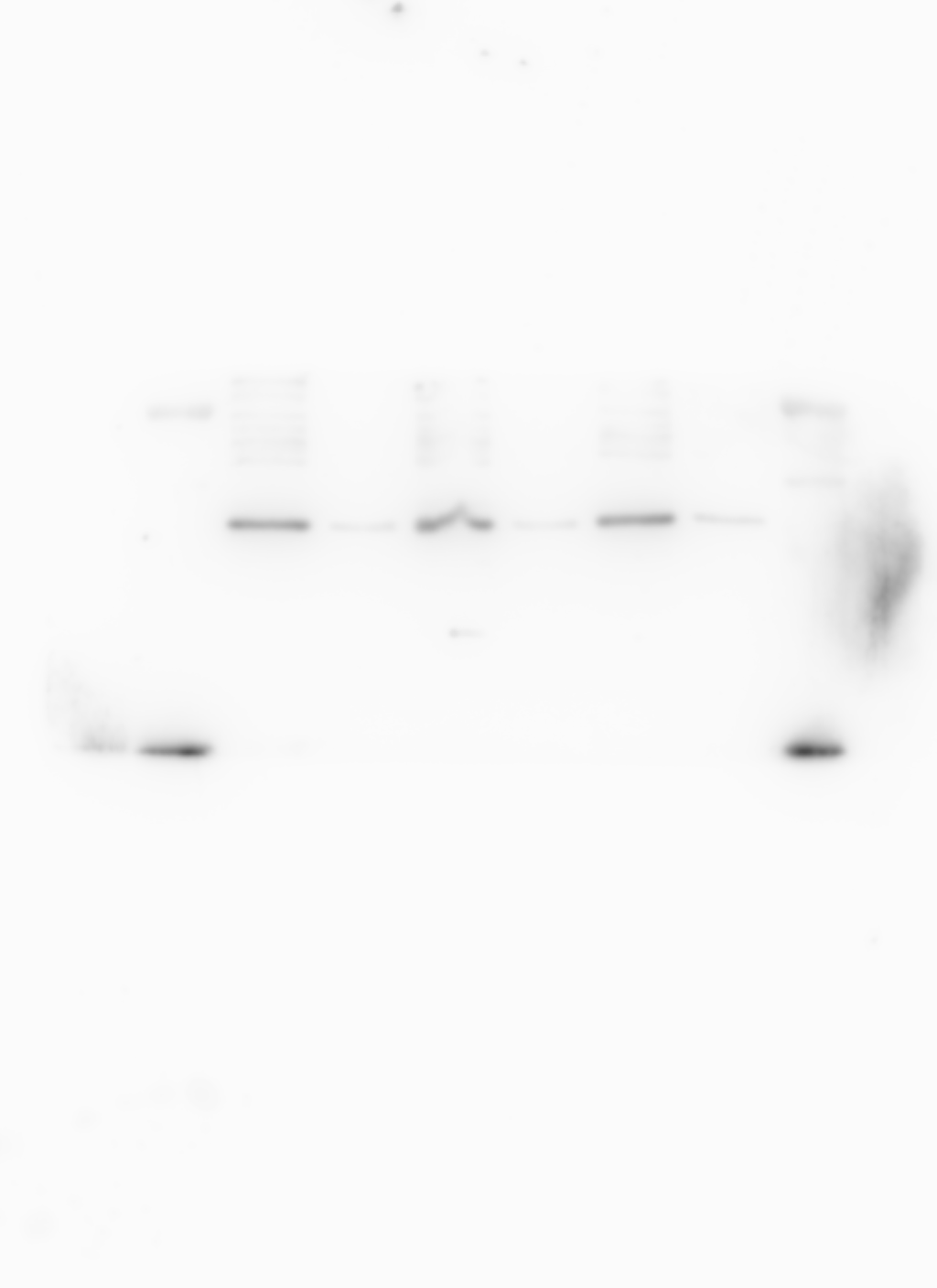

Supplement: Figure 5—source data 4. [file elife-97327-fig5-data4.zip › Figure 5-Source data 4/F5H-LCN2 .tif]

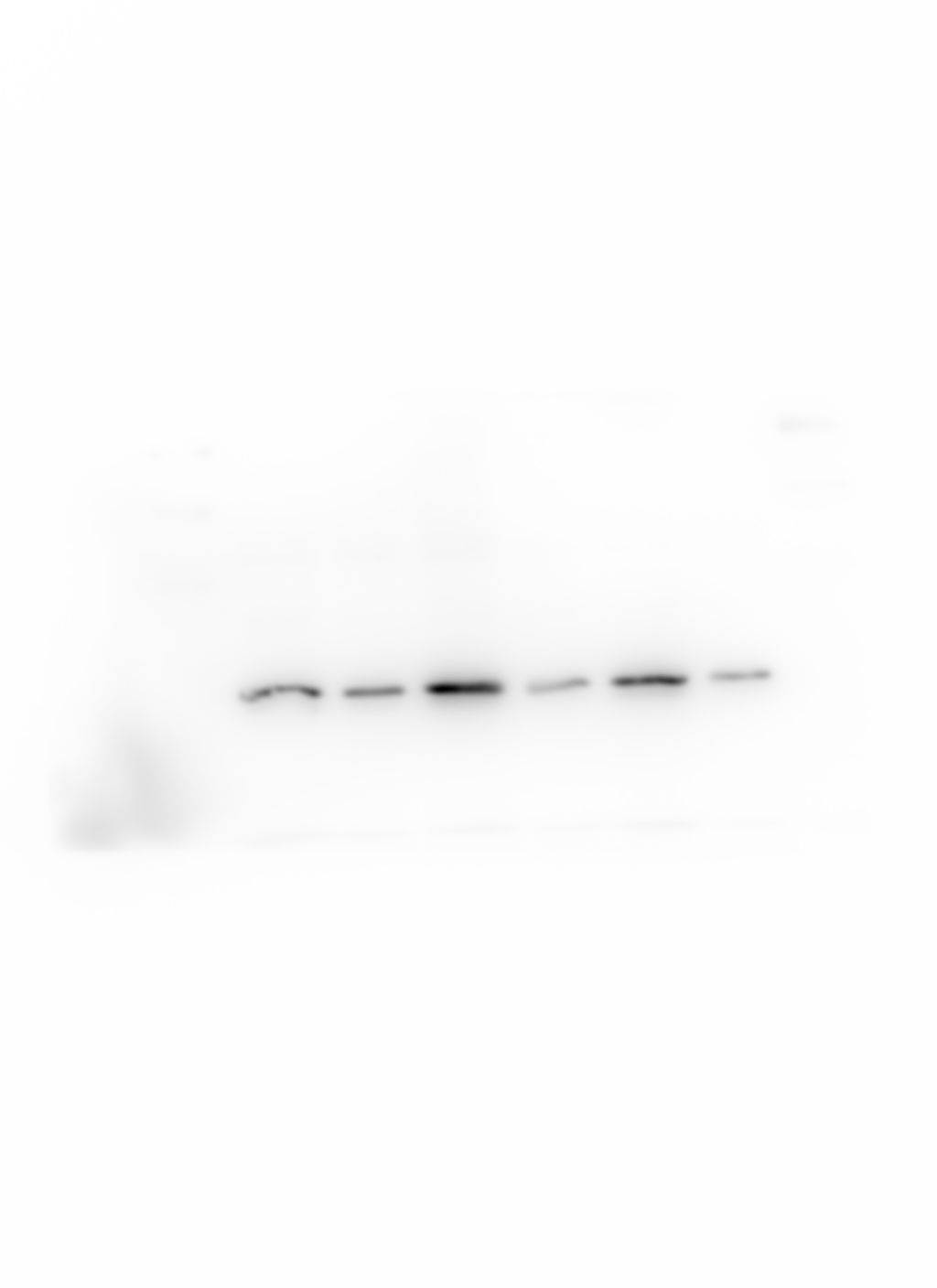

Supplement: Figure 5—source data 4. [file elife-97327-fig5-data4.zip › Figure 5-Source data 4/F5H-Snail .tif]

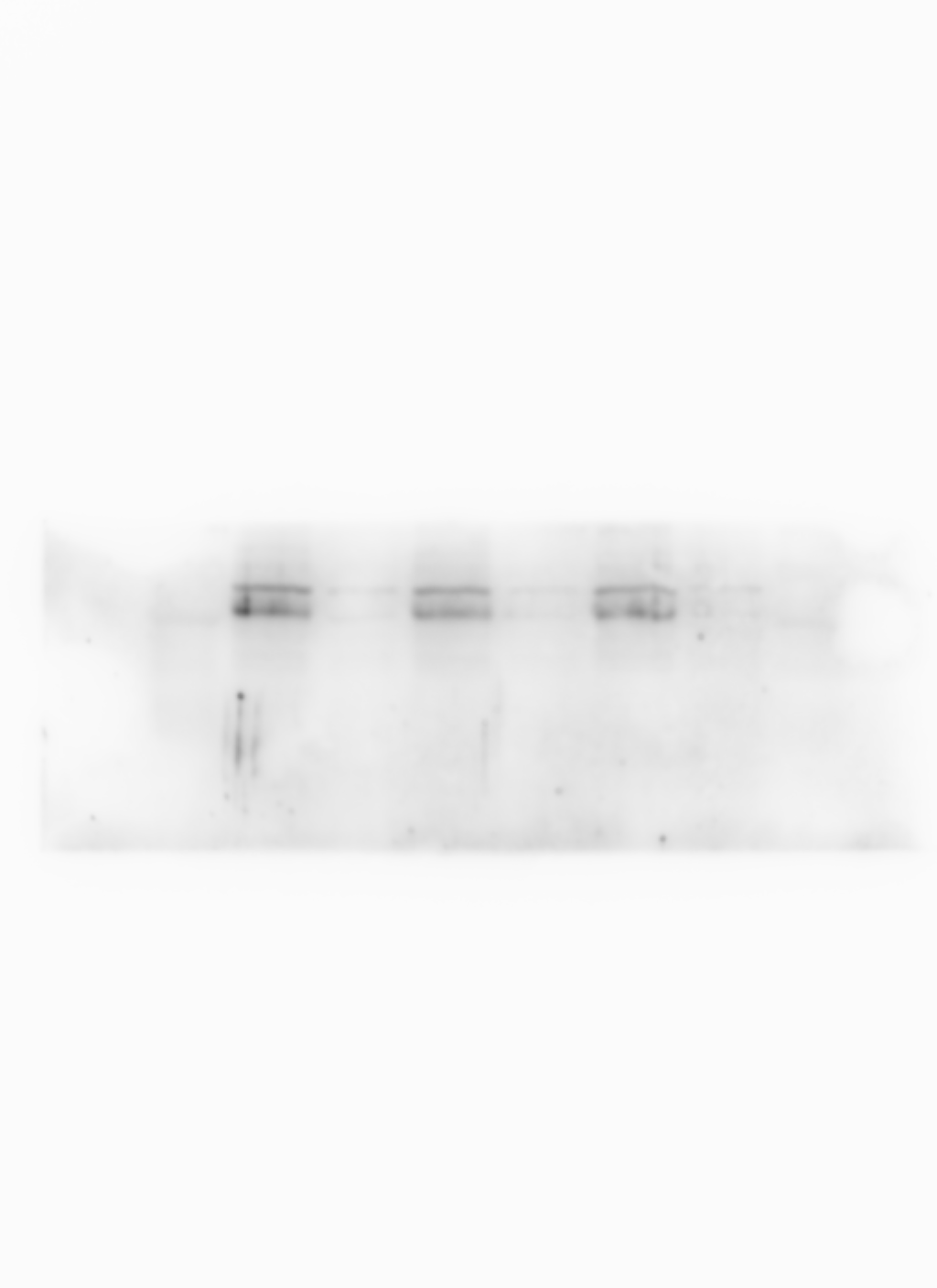

Supplement: Figure 5—source data 4. [file elife-97327-fig5-data4.zip › Figure 5-Source data 4/F5H-Vimentin .tif]

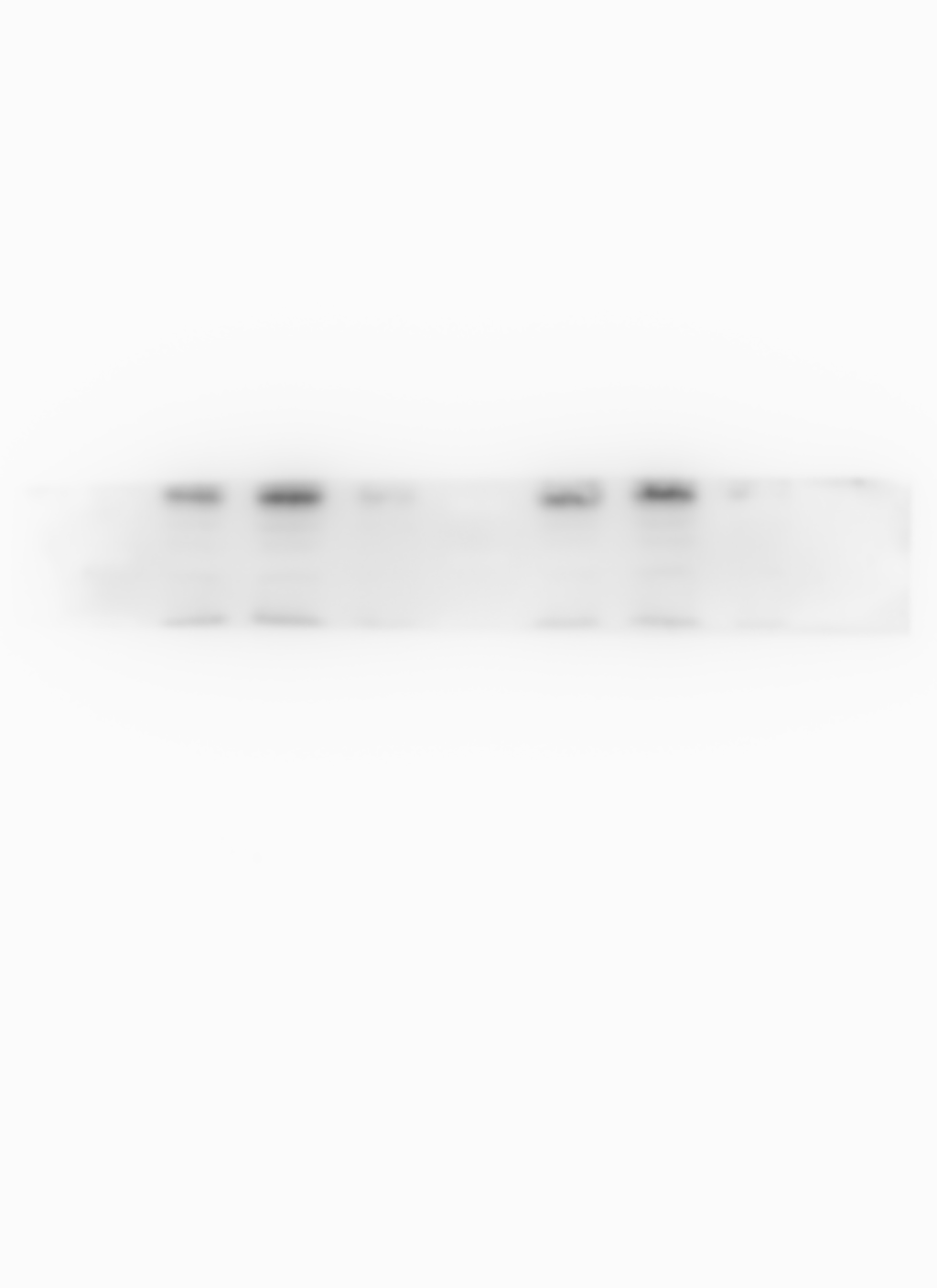

Supplement: Figure 5—source data 4. [file elife-97327-fig5-data4.zip › Figure 5-Source data 4/LCN2.tif]

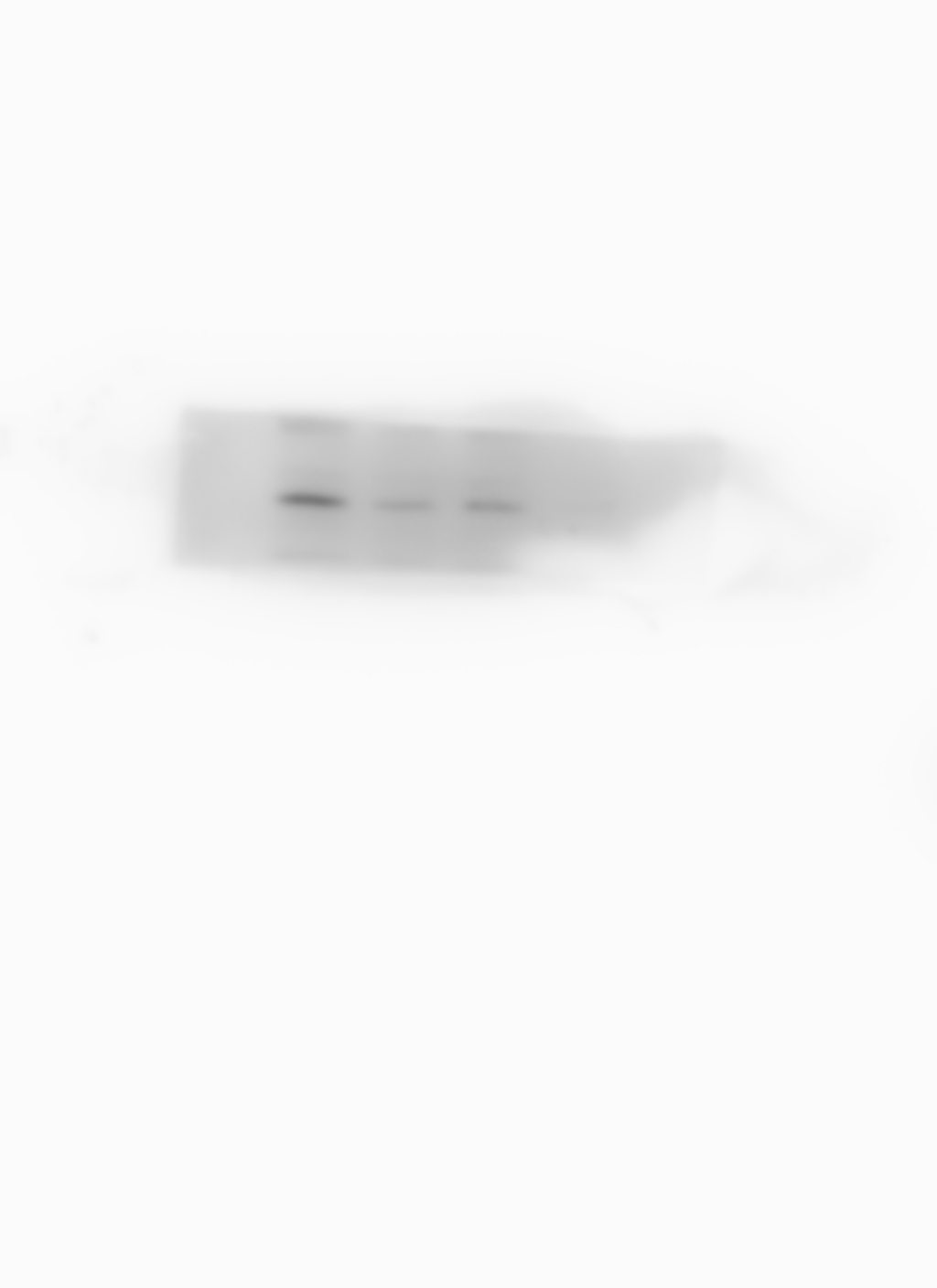

Supplement: Figure 5—source data 4. [file elife-97327-fig5-data4.zip › Figure 5-Source data 4/snail.tif]

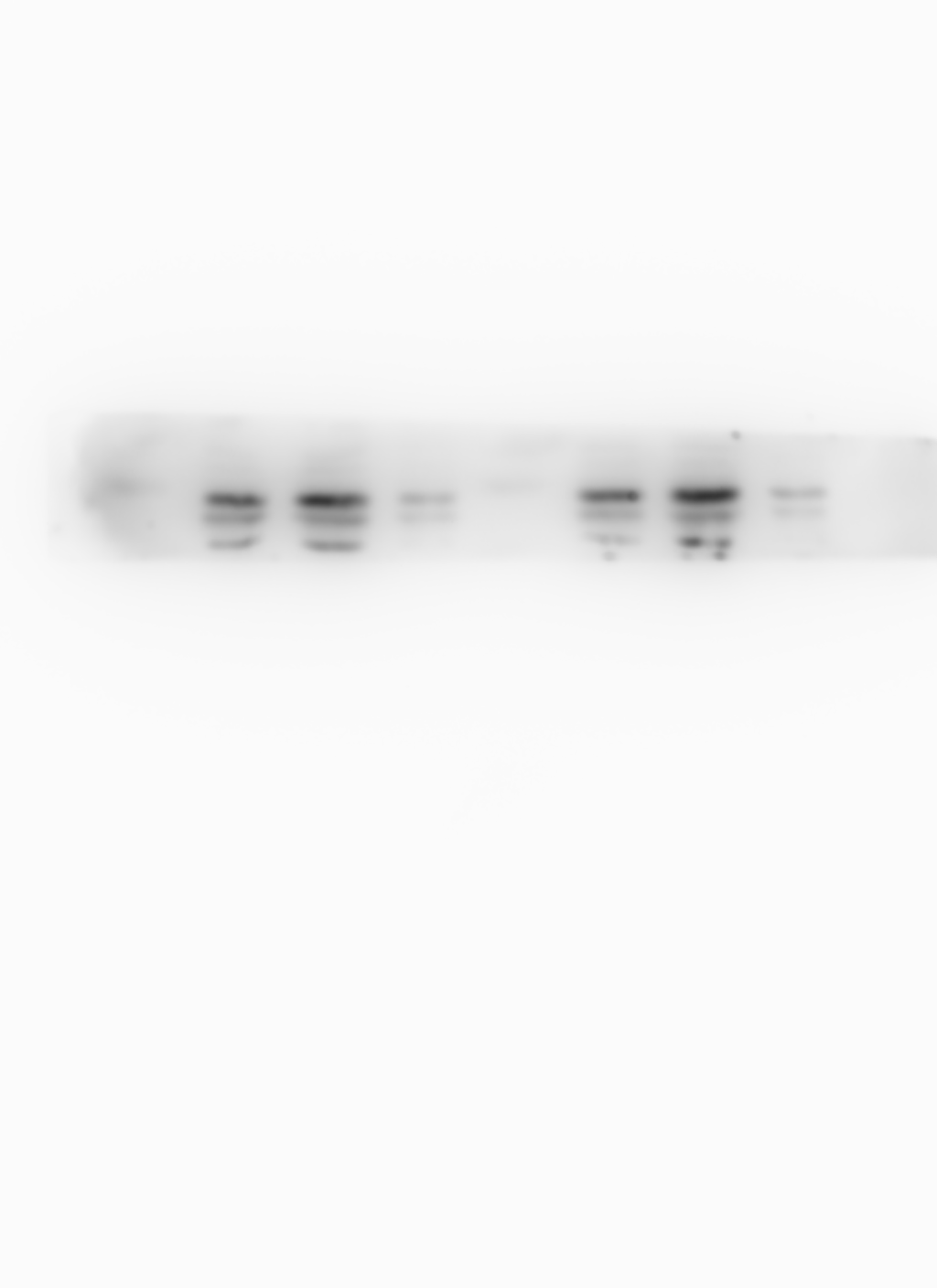

Supplement: Figure 5—source data 4. [file elife-97327-fig5-data4.zip › Figure 5-Source data 4/Vimentin.tif]

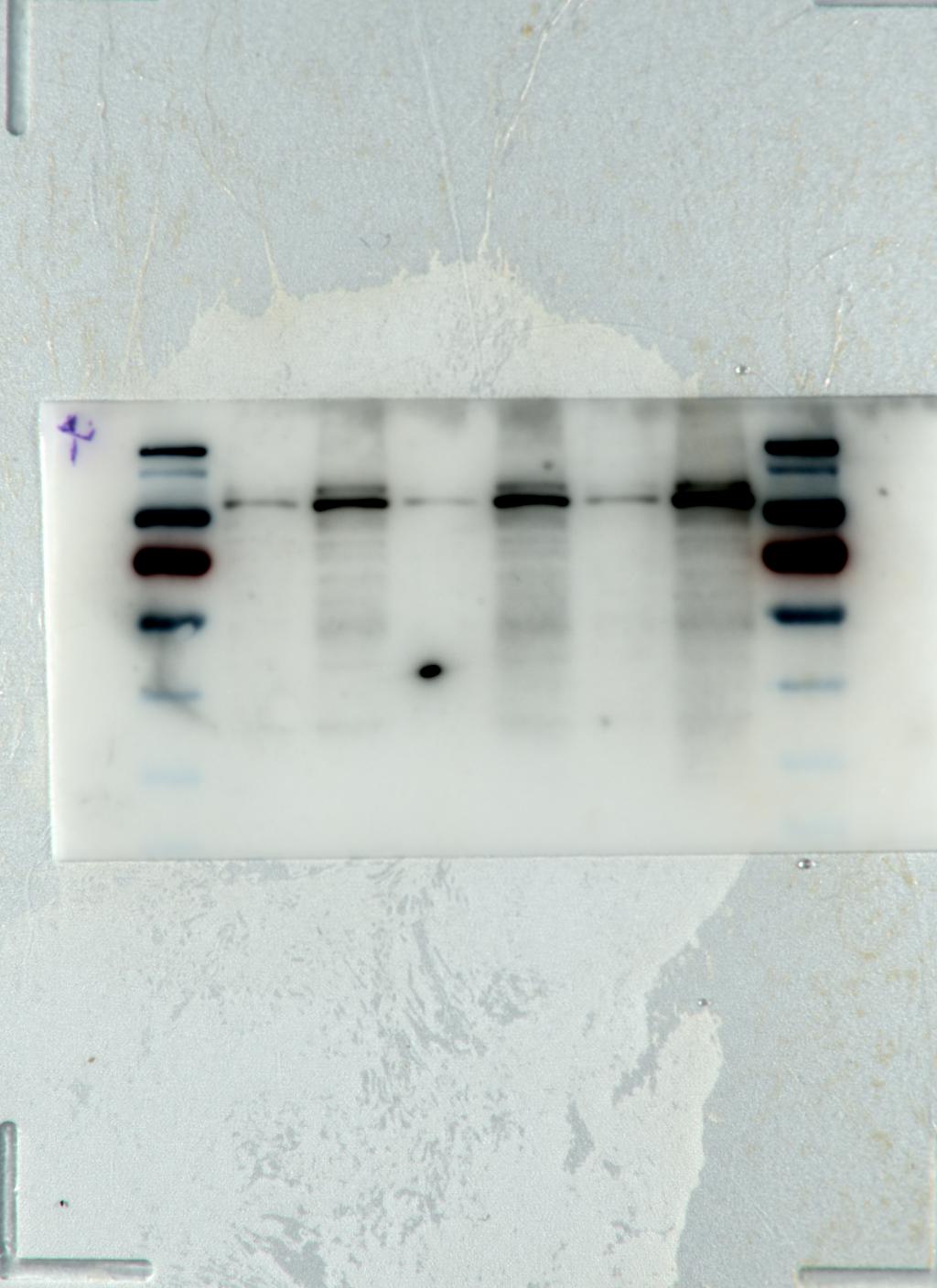

Supplement: Figure 5—source data 5. [file elife-97327-fig5-data5.zip › Figure 5-Source data 5/F5H-Eca +Marker.jpg]

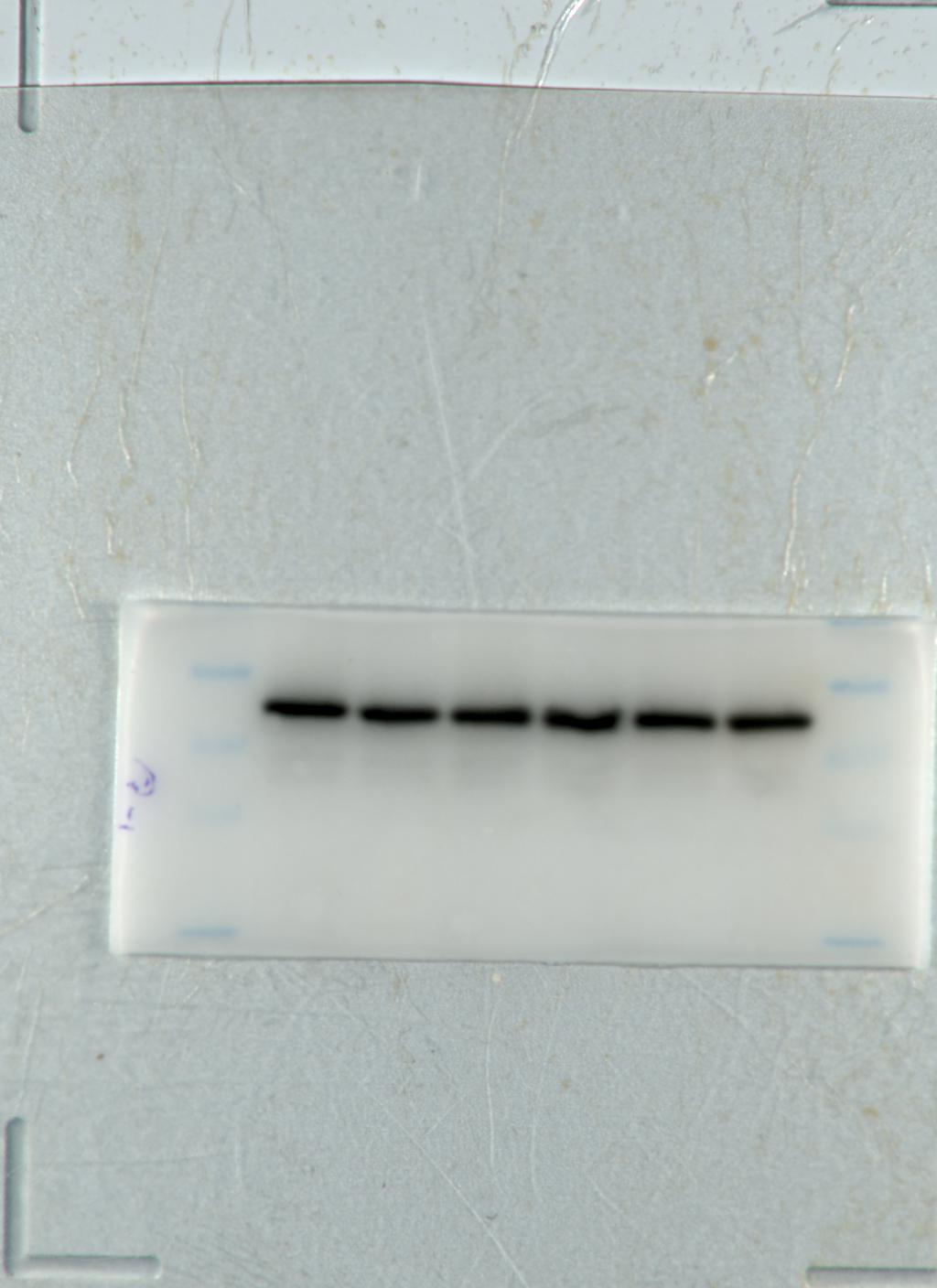

Supplement: Figure 5—source data 5. [file elife-97327-fig5-data5.zip › Figure 5-Source data 5/F5H-GAPDH +Marker.jpg]

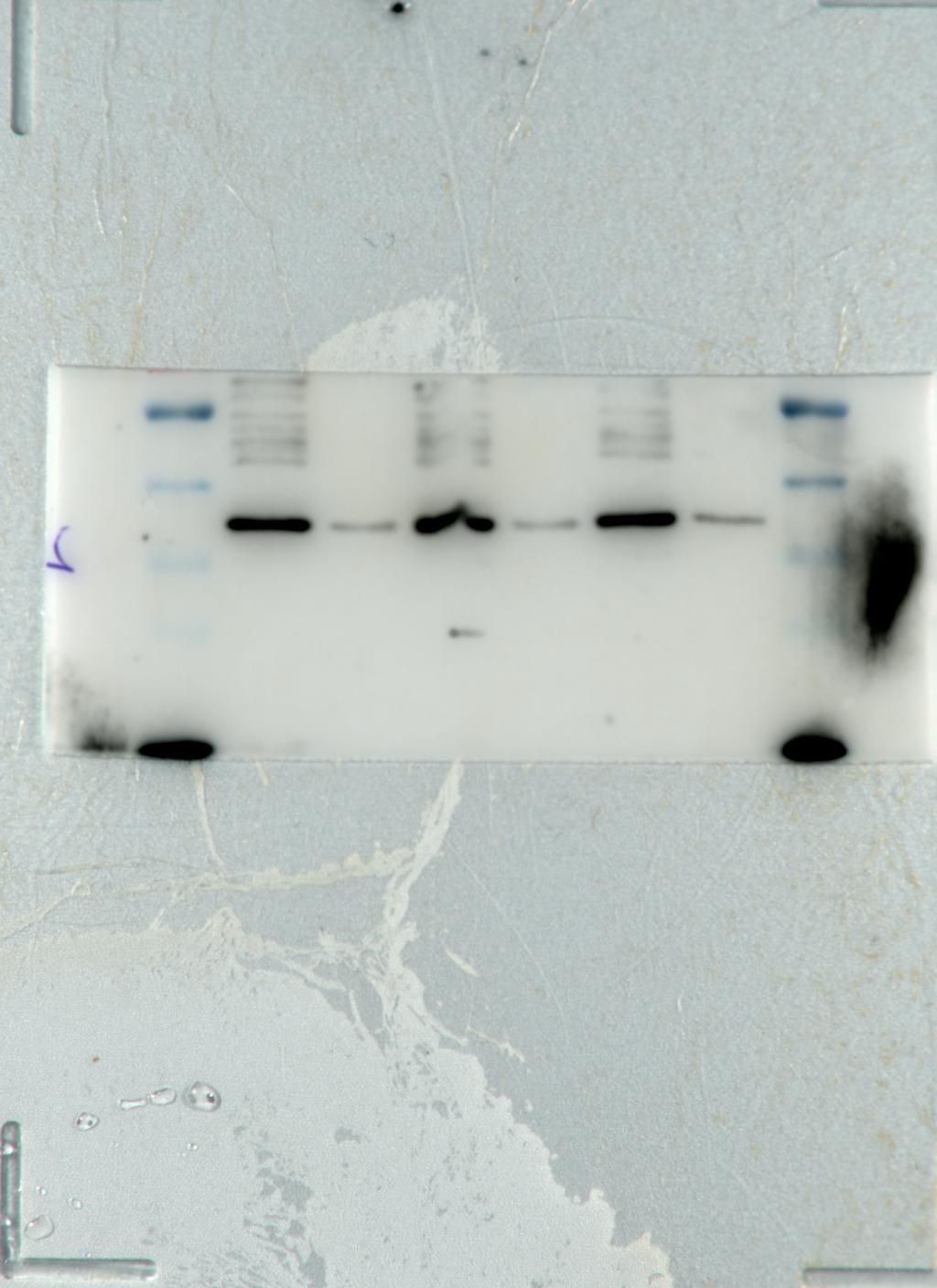

Supplement: Figure 5—source data 5. [file elife-97327-fig5-data5.zip › Figure 5-Source data 5/F5H-LCN2 +Marker.jpg]

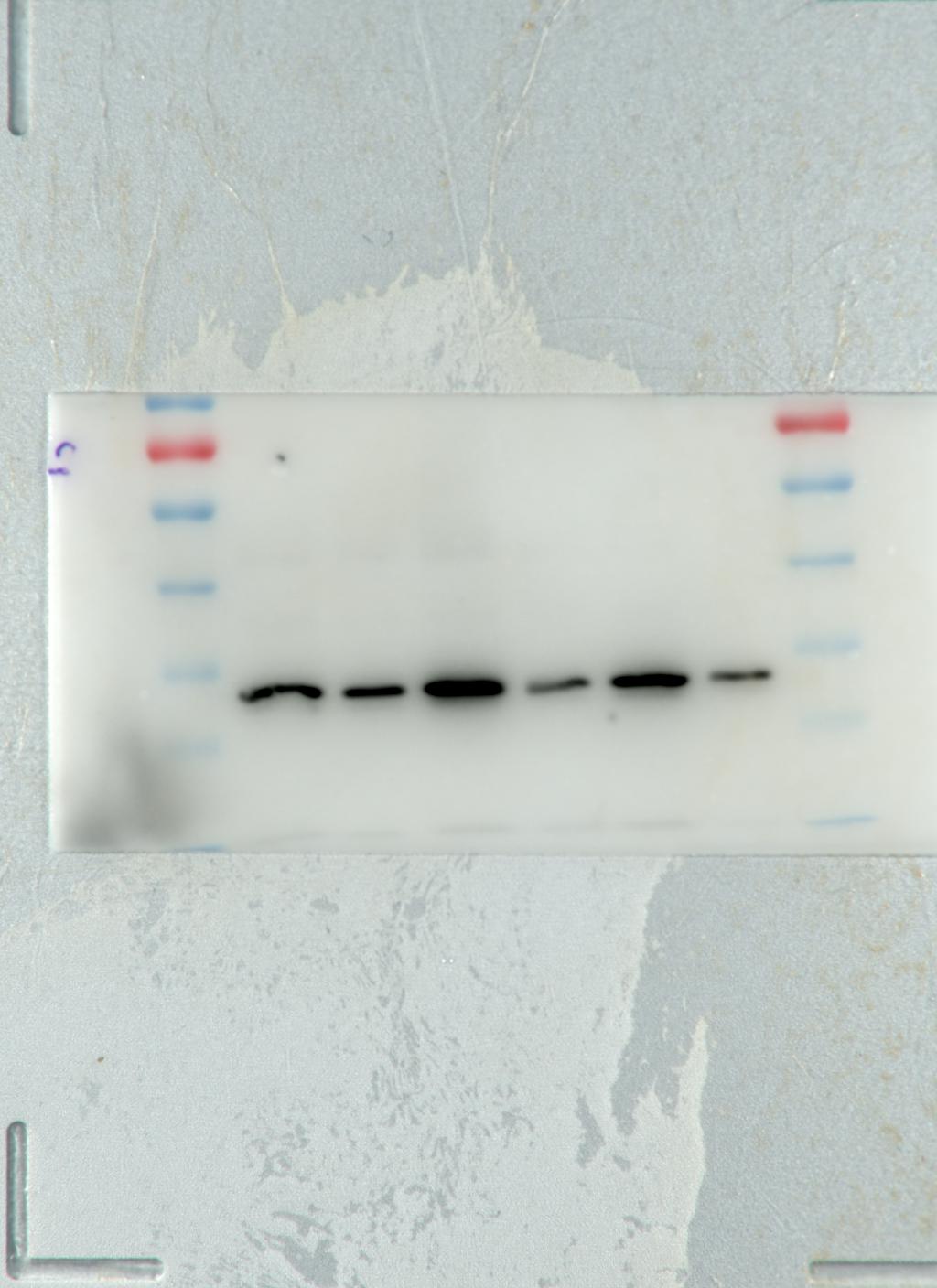

Supplement: Figure 5—source data 5. [file elife-97327-fig5-data5.zip › Figure 5-Source data 5/F5H-Snail +Marker.jpg]

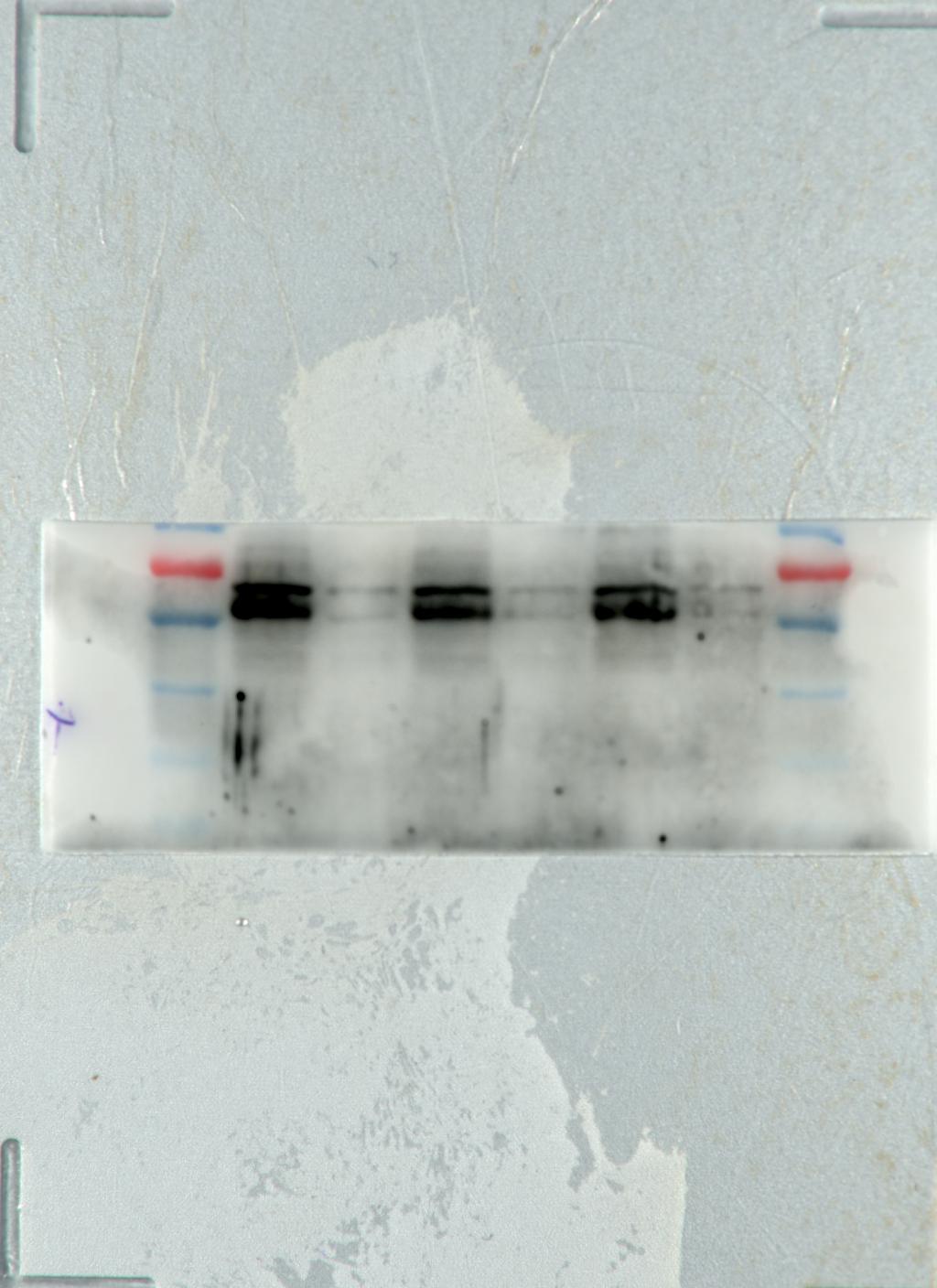

Supplement: Figure 5—source data 5. [file elife-97327-fig5-data5.zip › Figure 5-Source data 5/F5H-Vimentin +Marker.jpg]

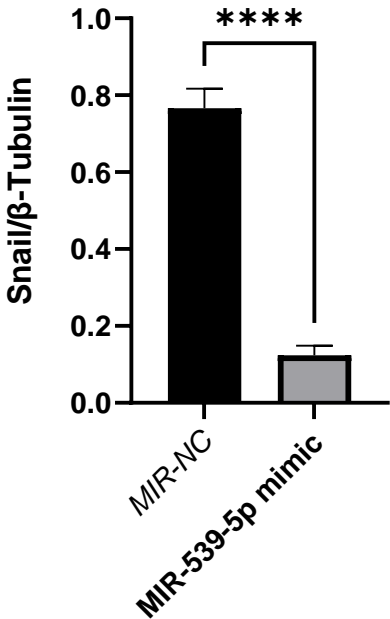

Supplement: Figure 5—source data 6. [file elife-97327-fig5-data6.pdf]

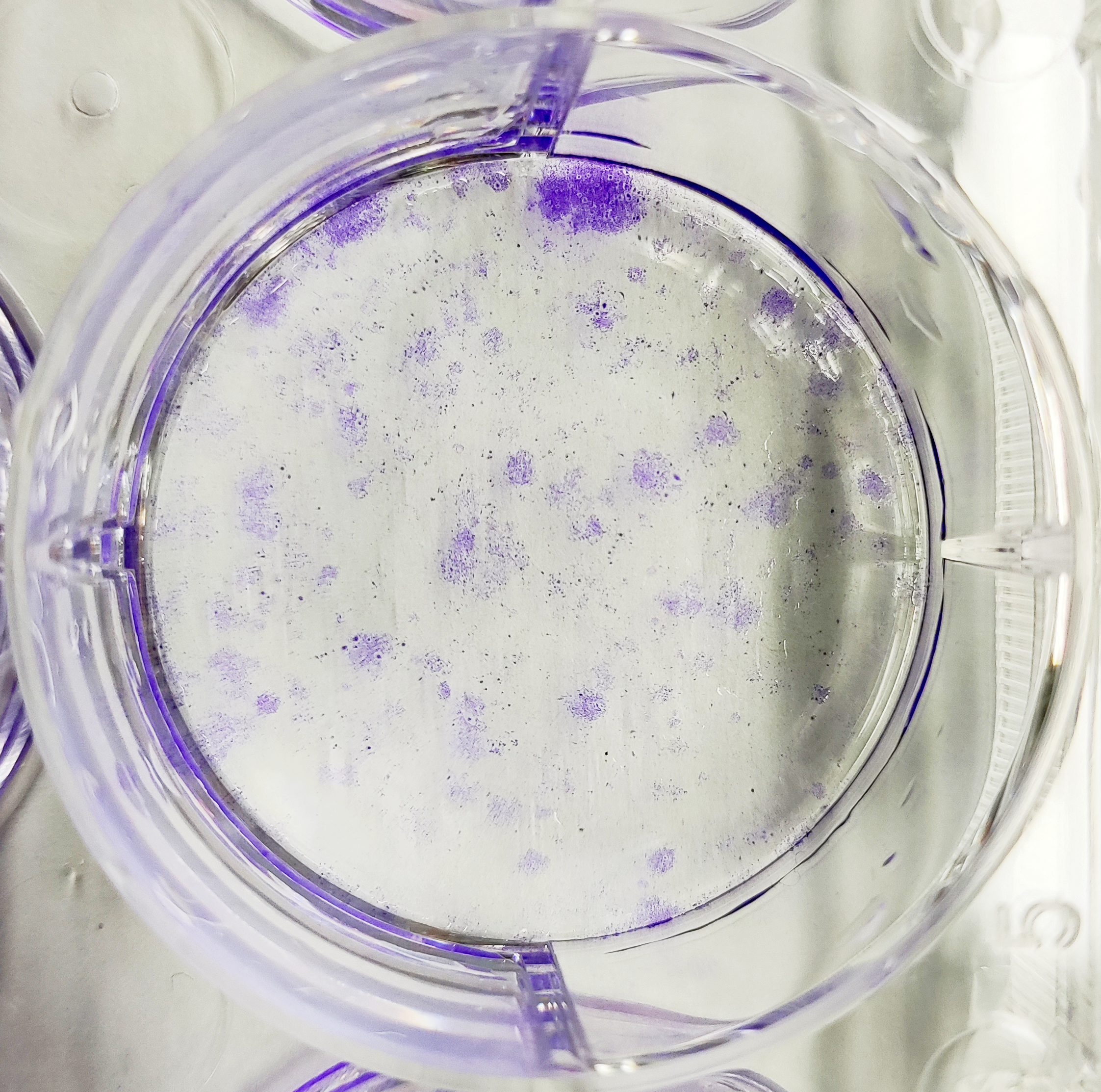

Supplement: Figure 5—source data 8. [file elife-97327-fig5-data8.zip › Figure 5-Source data 8/F5E-anti-miR-539-5p.tif]

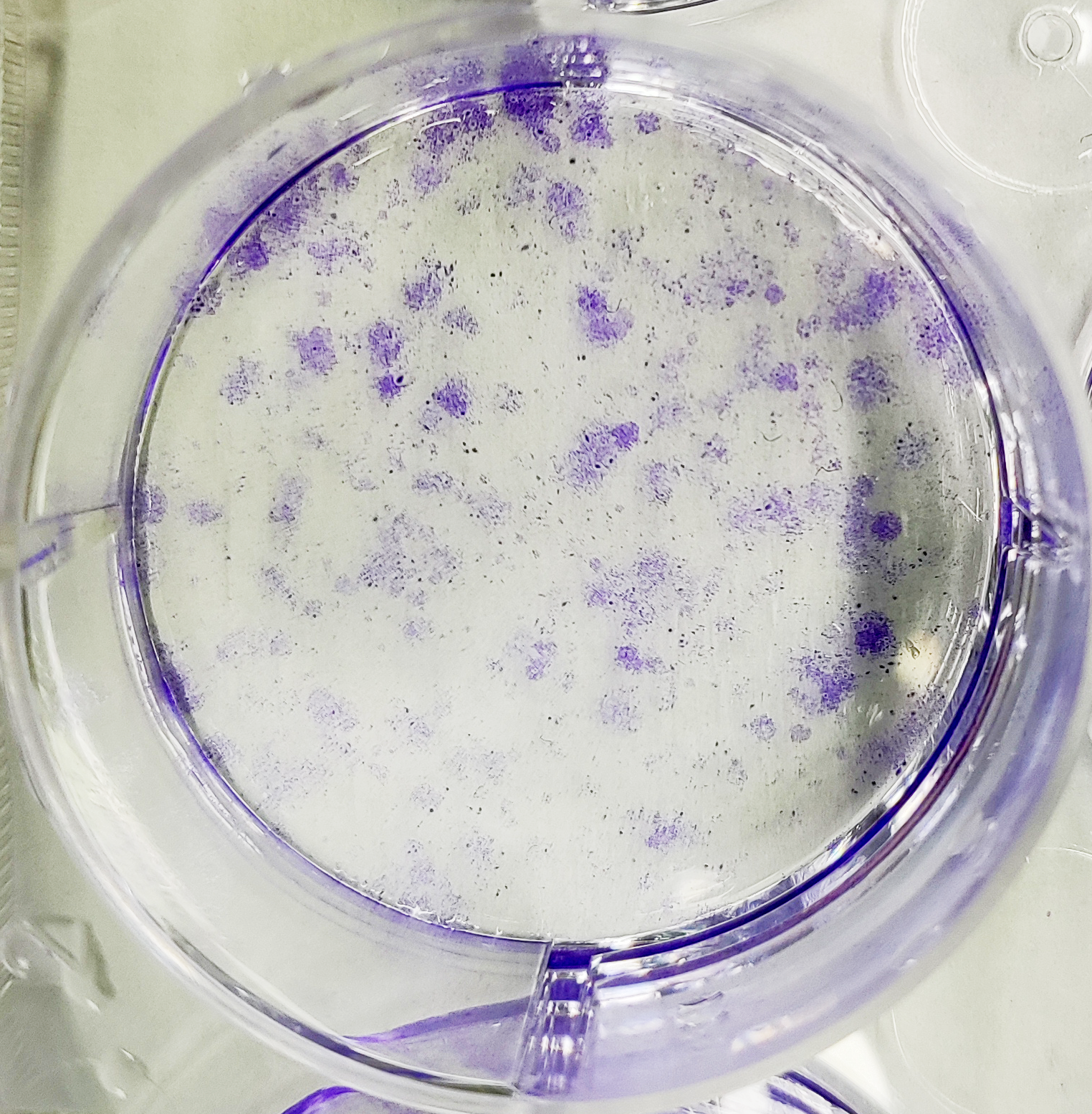

Supplement: Figure 5—source data 8. [file elife-97327-fig5-data8.zip › Figure 5-Source data 8/F5E-anti-NC.tif]

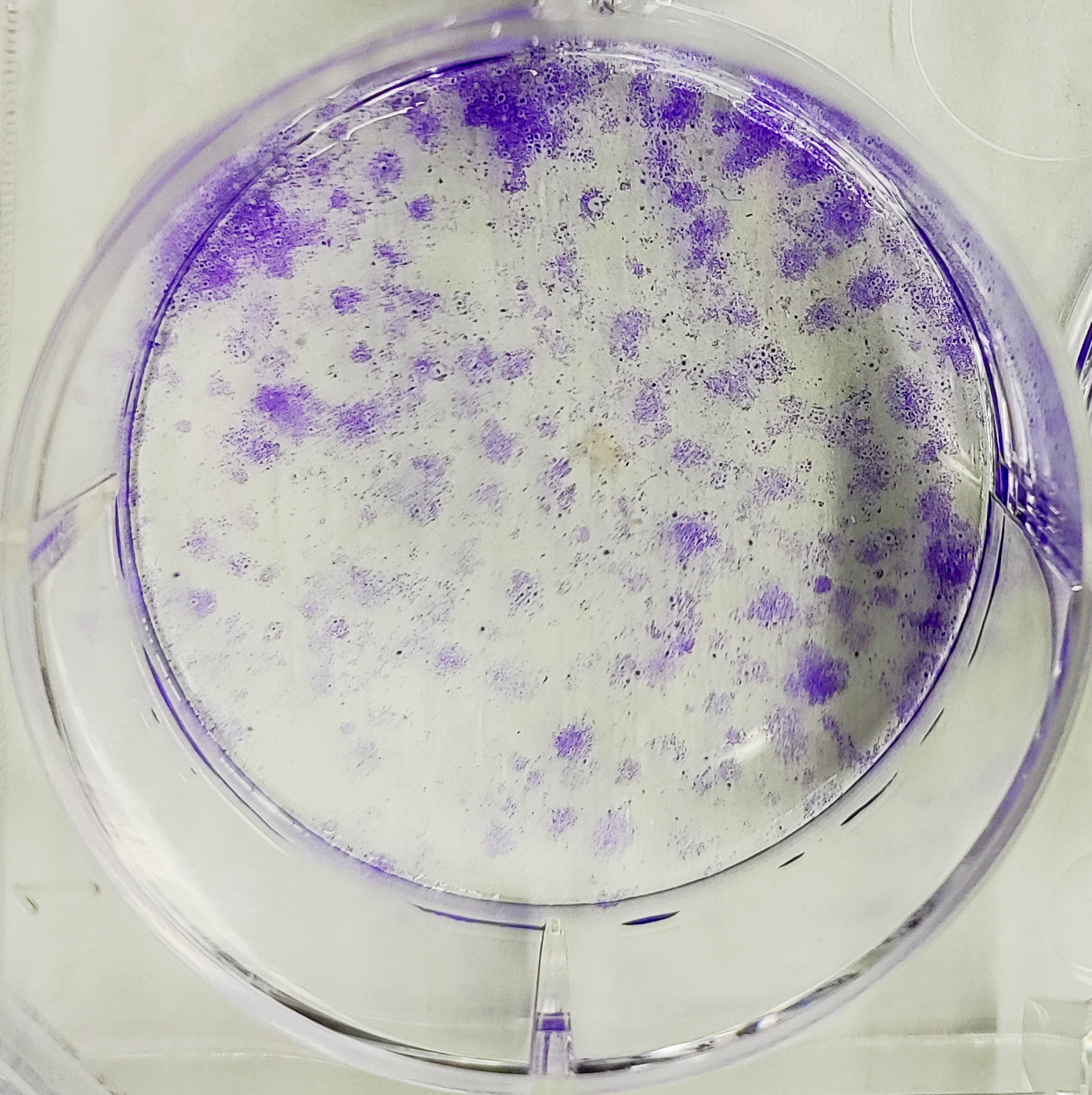

Supplement: Figure 5—source data 8. [file elife-97327-fig5-data8.zip › Figure 5-Source data 8/F5E-WT.tif]

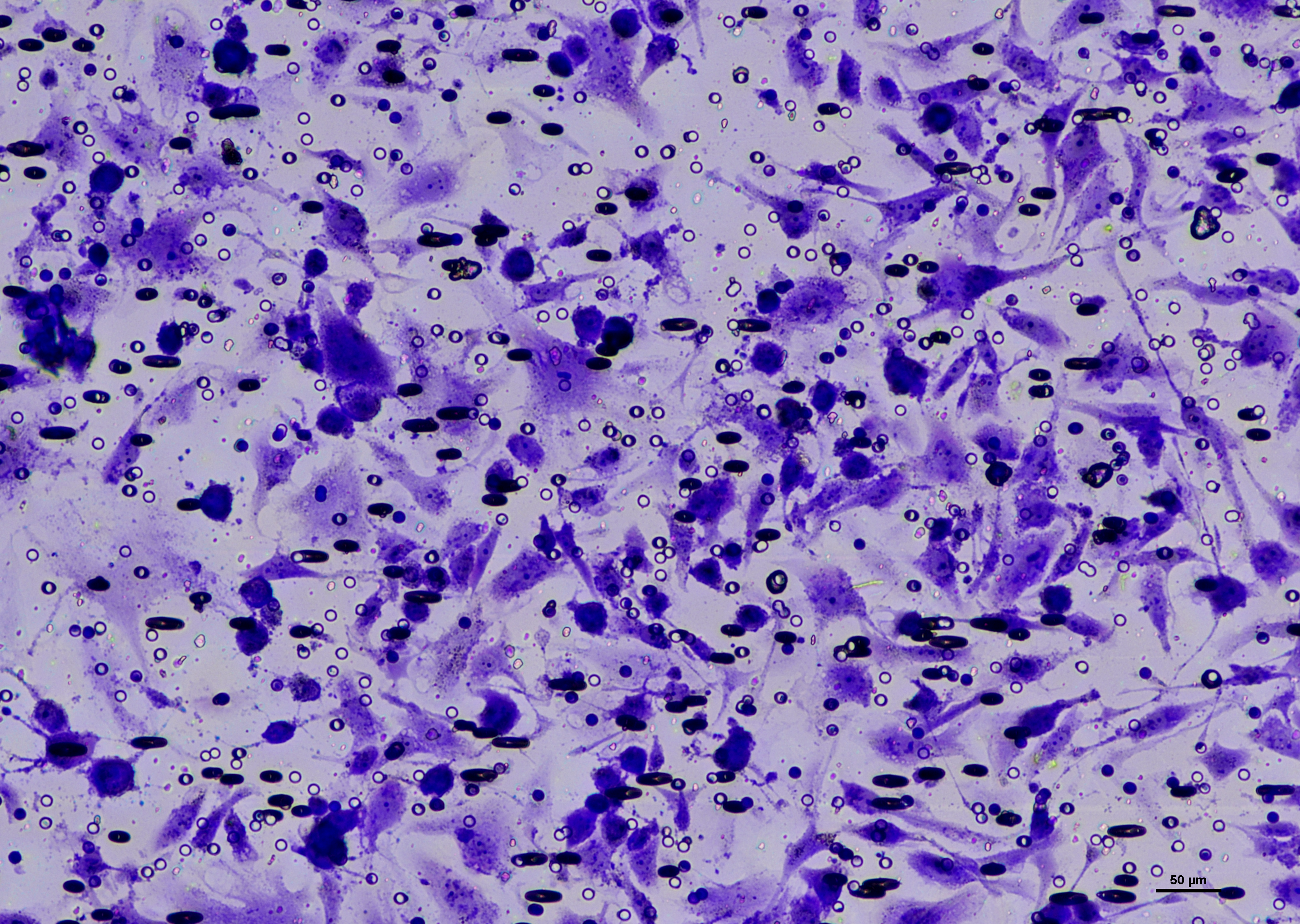

Supplement: Figure 5—source data 9. [file elife-97327-fig5-data9.zip › Figure 5-Source data 9/F5F-anti-NC.tif]

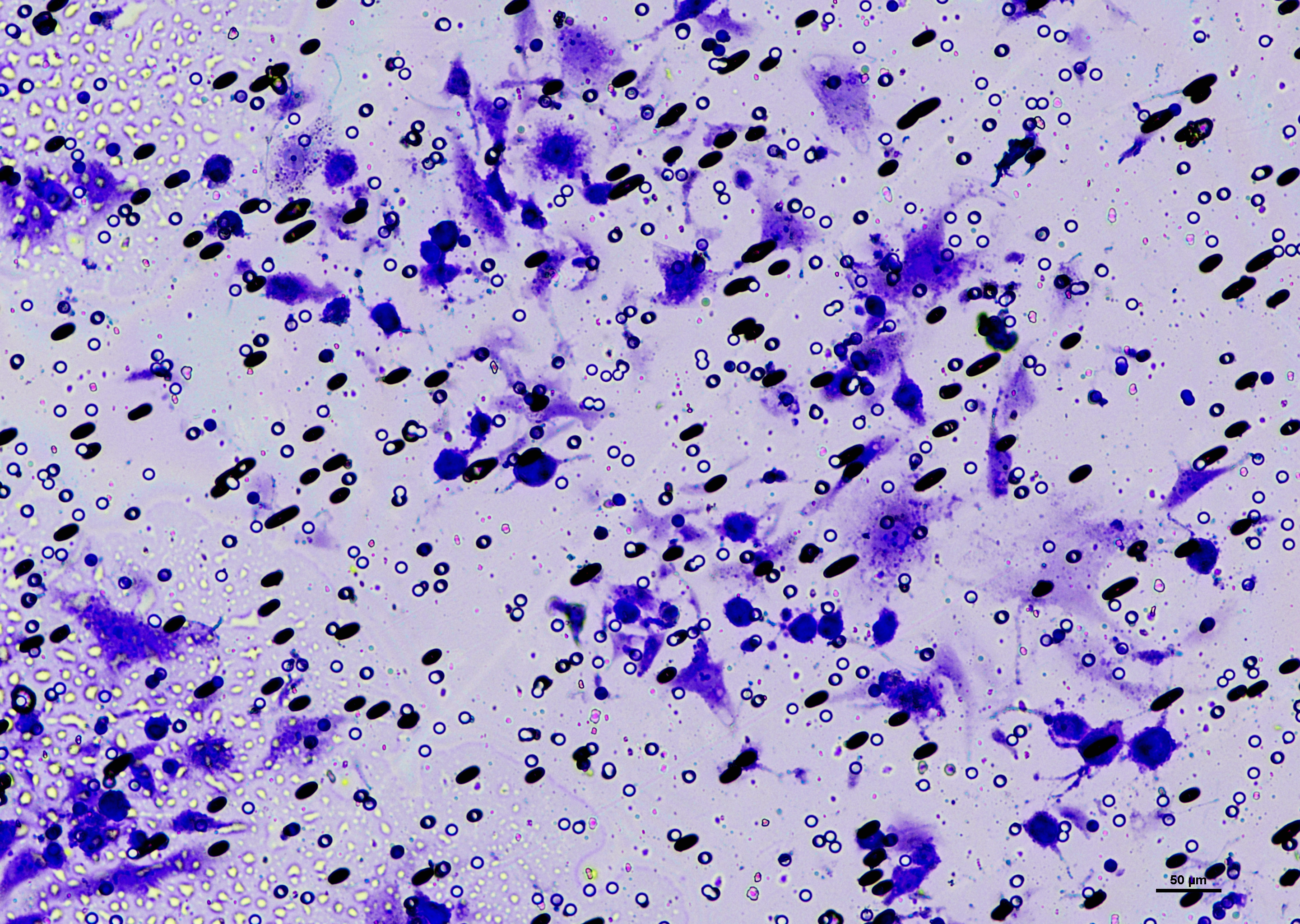

Supplement: Figure 5—source data 9. [file elife-97327-fig5-data9.zip › Figure 5-Source data 9/F5F-miR-539-5p inhibitor.tif]

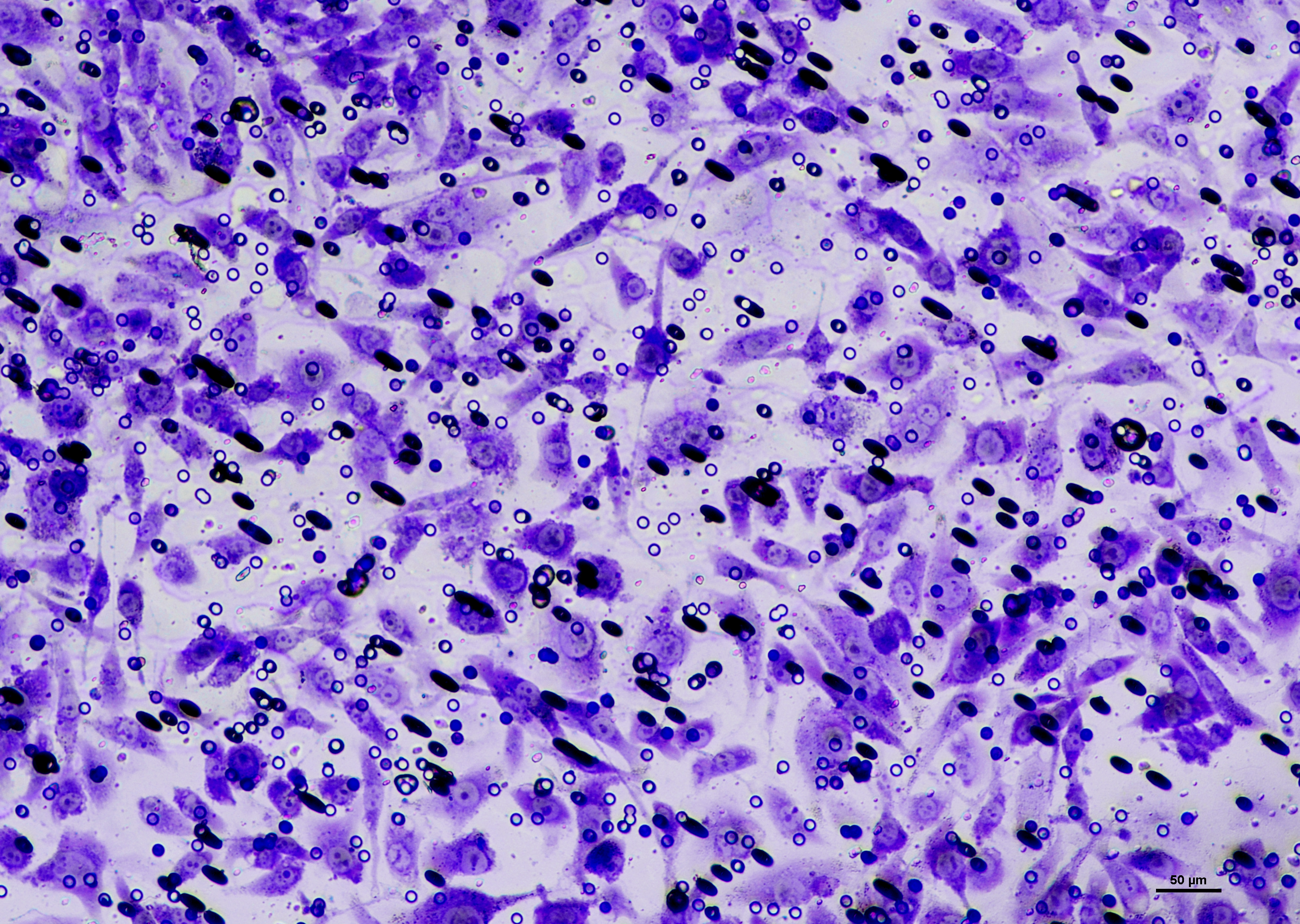

Supplement: Figure 5—source data 9. [file elife-97327-fig5-data9.zip › Figure 5-Source data 9/F5F-WT.tif]

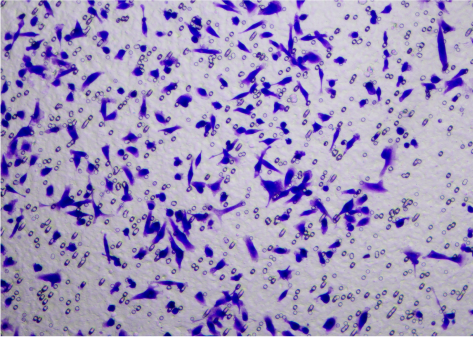

Supplement: Figure 5—source data 10. [file elife-97327-fig5-data10.zip › Figure 5-Source data 10/F5G-miR-539-5P inhibitor.tif]

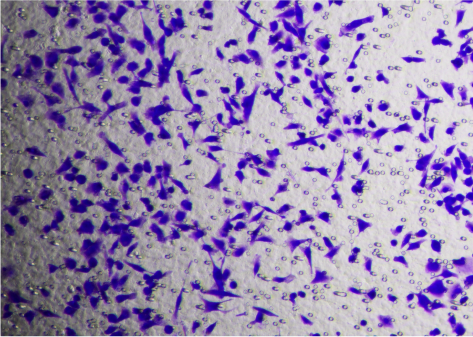

Supplement: Figure 5—source data 10. [file elife-97327-fig5-data10.zip › Figure 5-Source data 10/F5G-NC.tif]

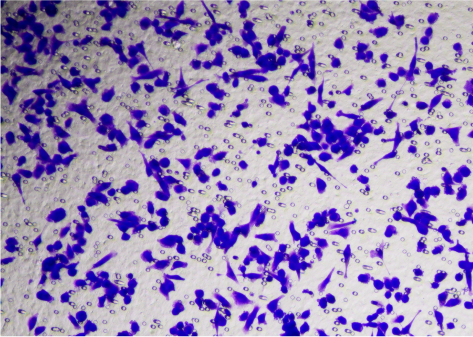

Supplement: Figure 5—source data 10. [file elife-97327-fig5-data10.zip › Figure 5-Source data 10/F5G-WT.tif]

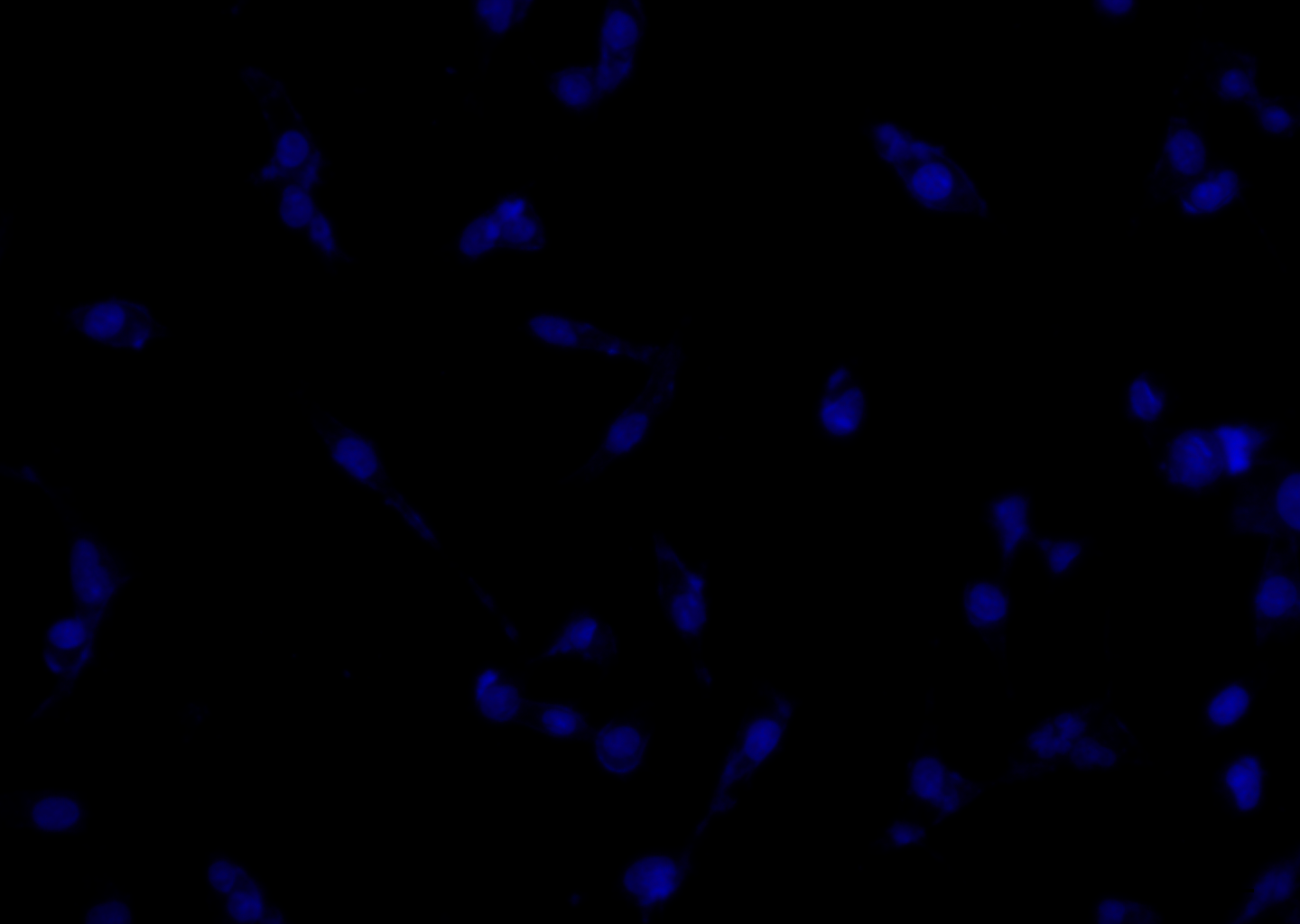

Supplement: Figure 5—source data 11. [file elife-97327-fig5-data11.zip › Figure 5-Source data 11/E-cadherin-inhibitor-DAPI.tif]

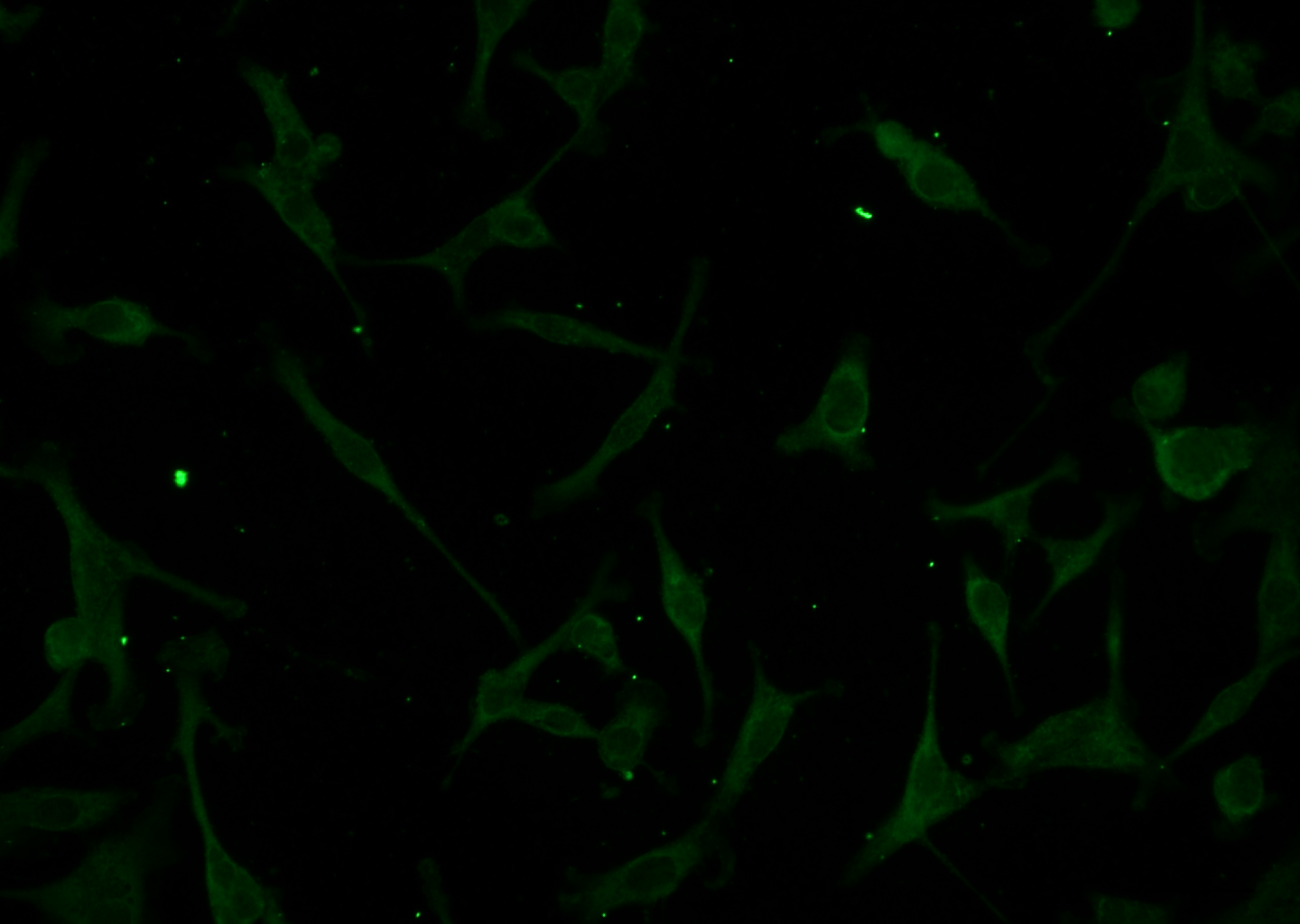

Supplement: Figure 5—source data 11. [file elife-97327-fig5-data11.zip › Figure 5-Source data 11/E-cadherin-inhibitor-FITC.tif]

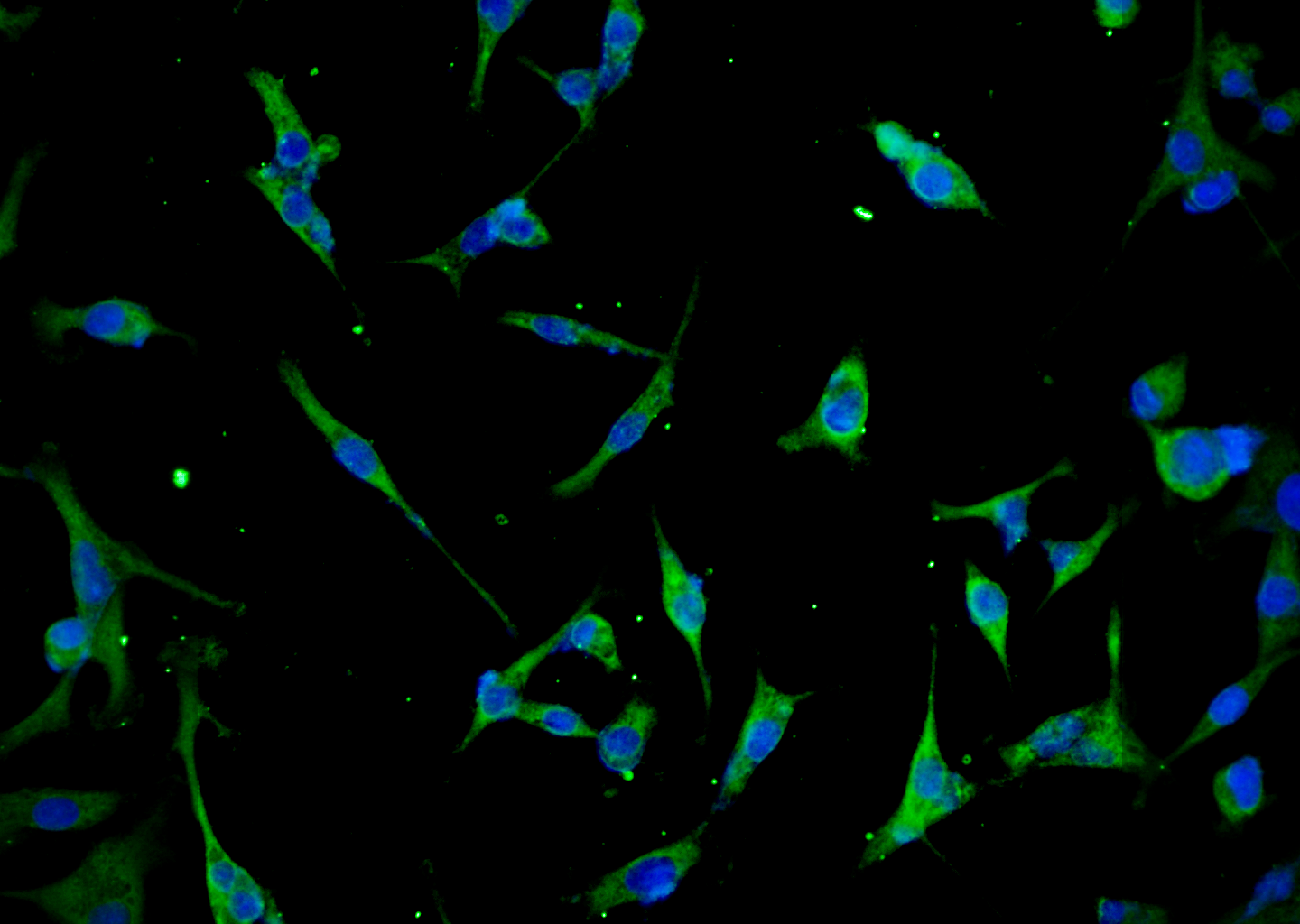

Supplement: Figure 5—source data 11. [file elife-97327-fig5-data11.zip › Figure 5-Source data 11/E-cadherin-inhibitor-merged.tif]

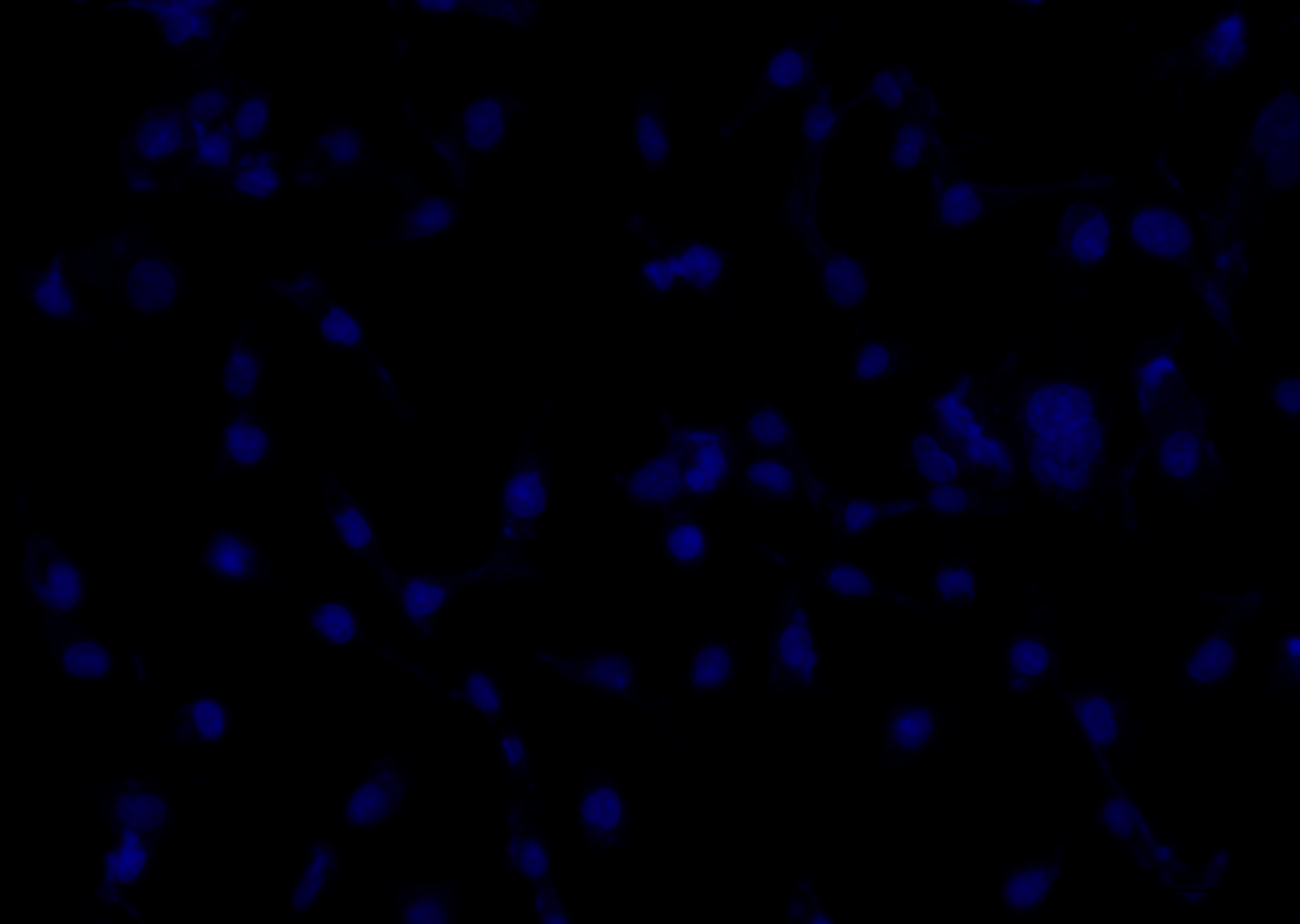

Supplement: Figure 5—source data 11. [file elife-97327-fig5-data11.zip › Figure 5-Source data 11/E-cadherin-NC-DAPI.tif]

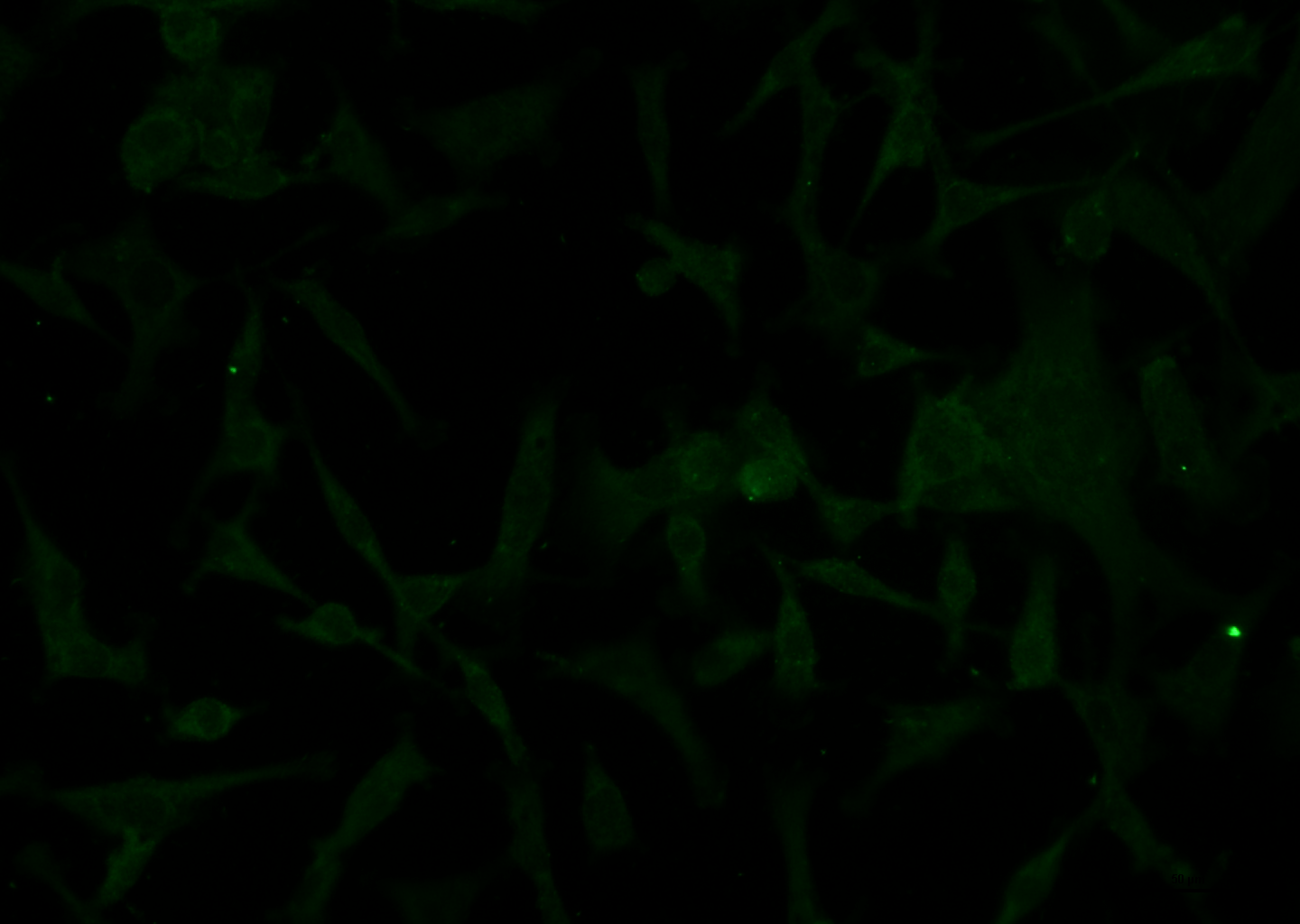

Supplement: Figure 5—source data 11. [file elife-97327-fig5-data11.zip › Figure 5-Source data 11/E-cadherin-NC-FITC.tif]

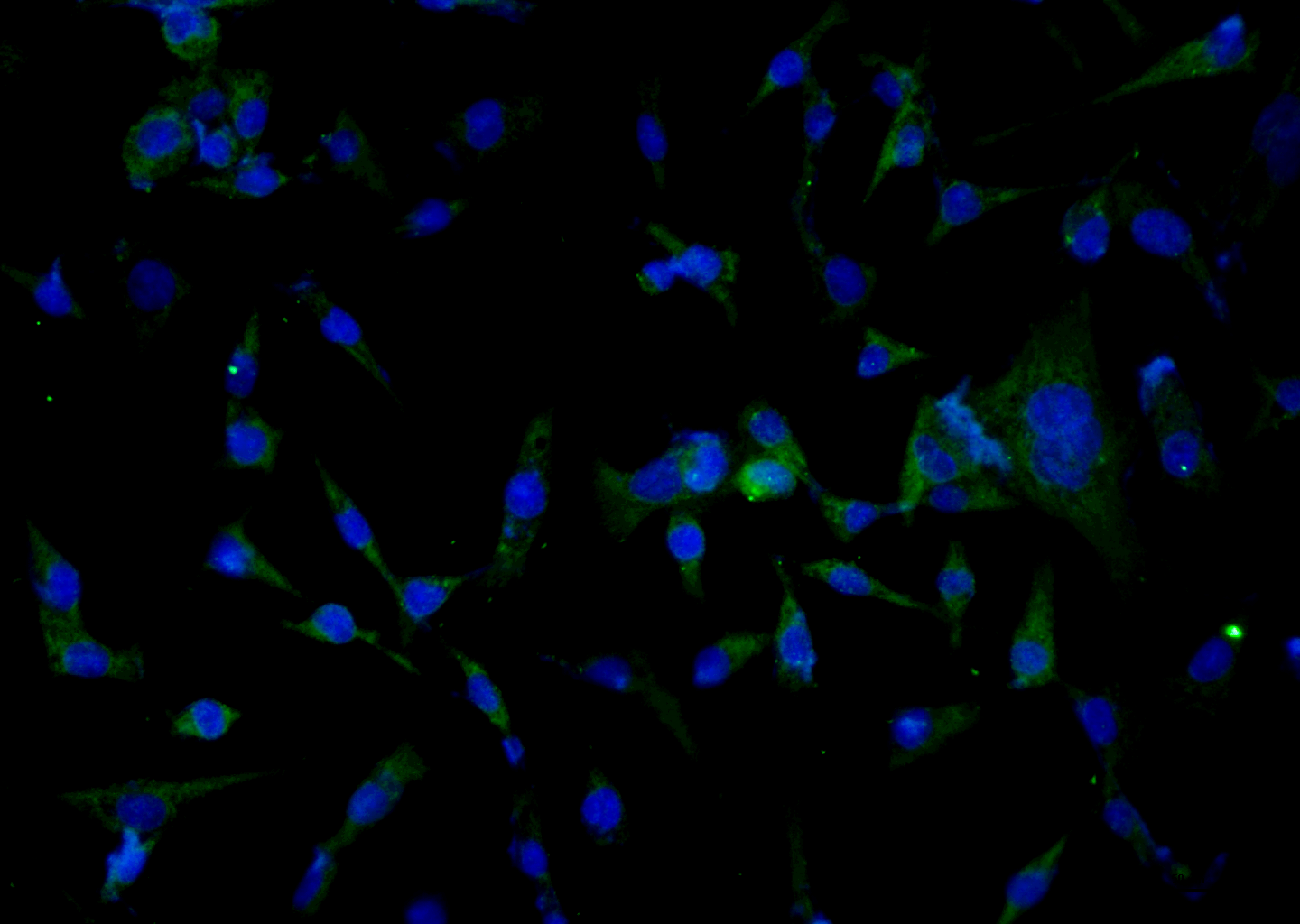

Supplement: Figure 5—source data 11. [file elife-97327-fig5-data11.zip › Figure 5-Source data 11/E-cadherin-NC-merged.tif]

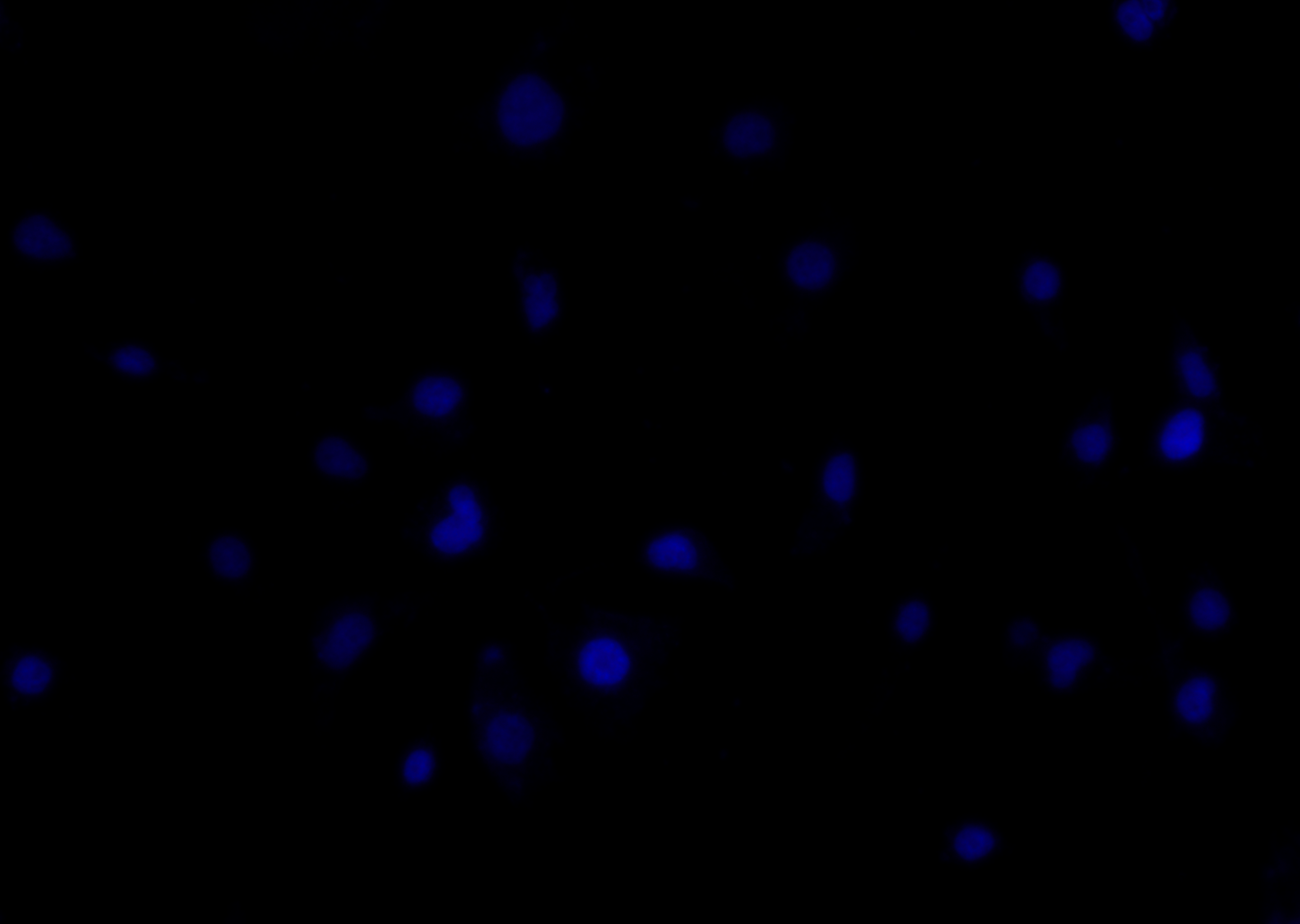

Supplement: Figure 5—source data 11. [file elife-97327-fig5-data11.zip › Figure 5-Source data 11/Snail-inhibitor-DAPI.tif]

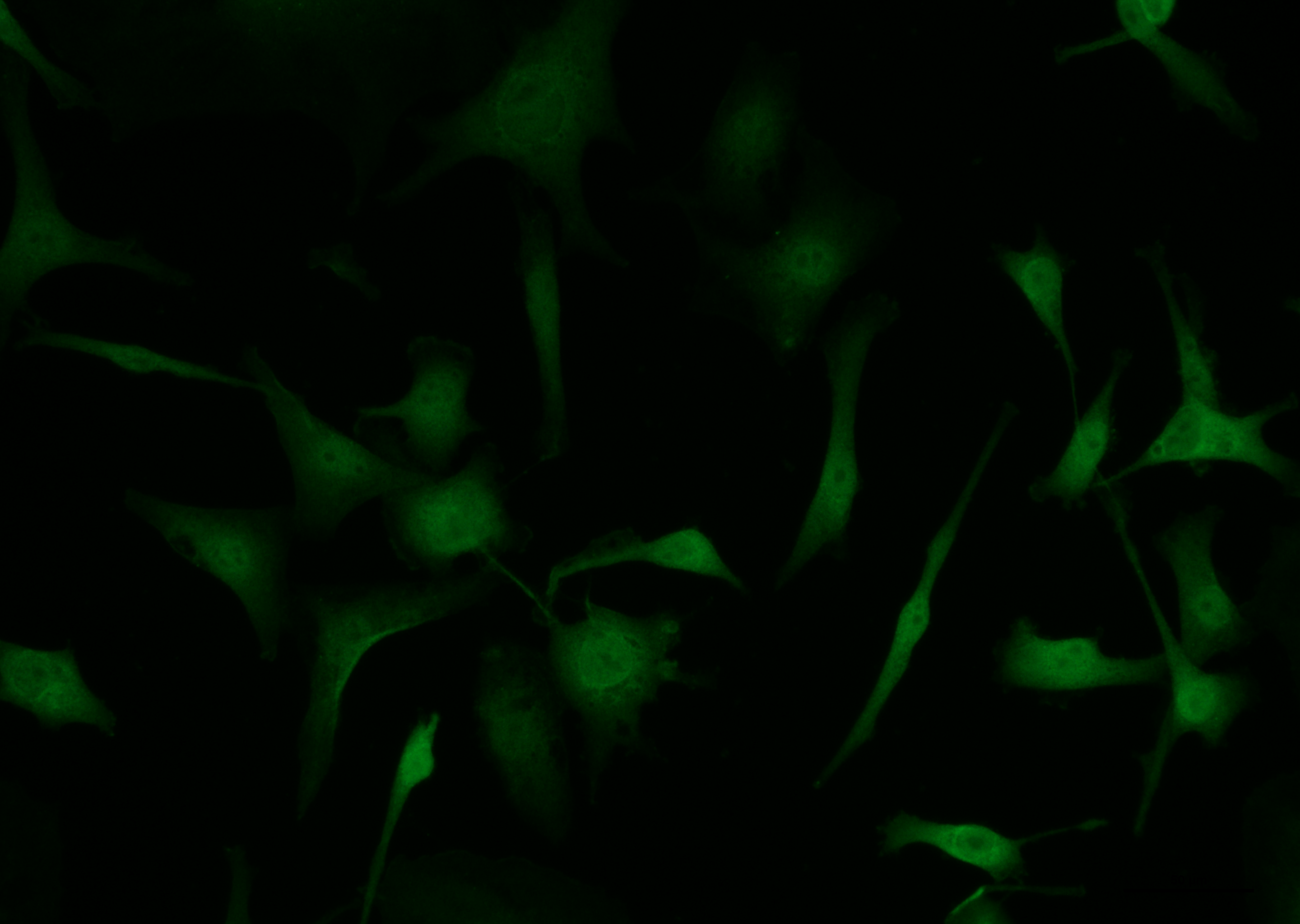

Supplement: Figure 5—source data 11. [file elife-97327-fig5-data11.zip › Figure 5-Source data 11/Snail-inhibitor-FITC.tif]

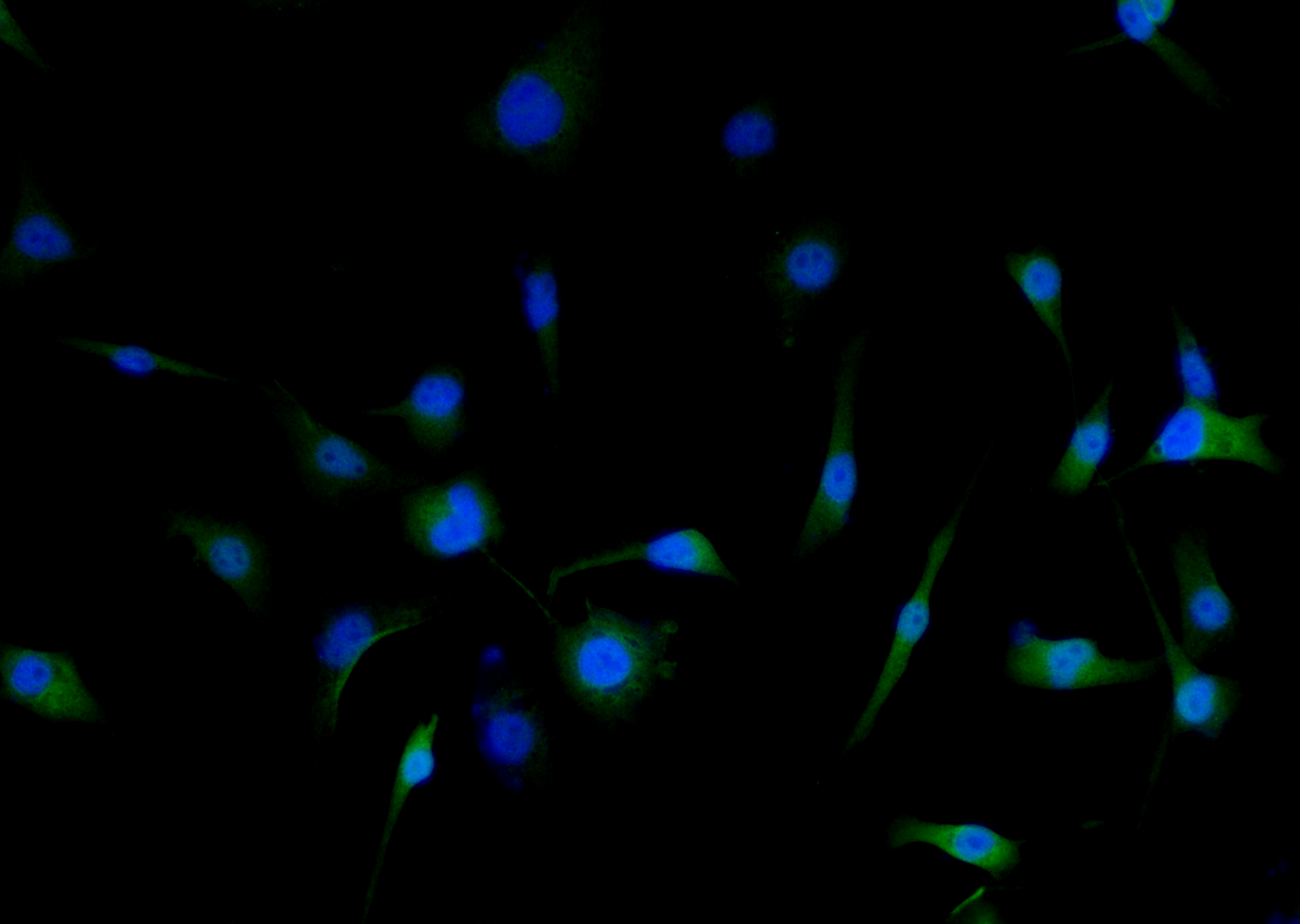

Supplement: Figure 5—source data 11. [file elife-97327-fig5-data11.zip › Figure 5-Source data 11/Snail-inhibitor-merged.tif]

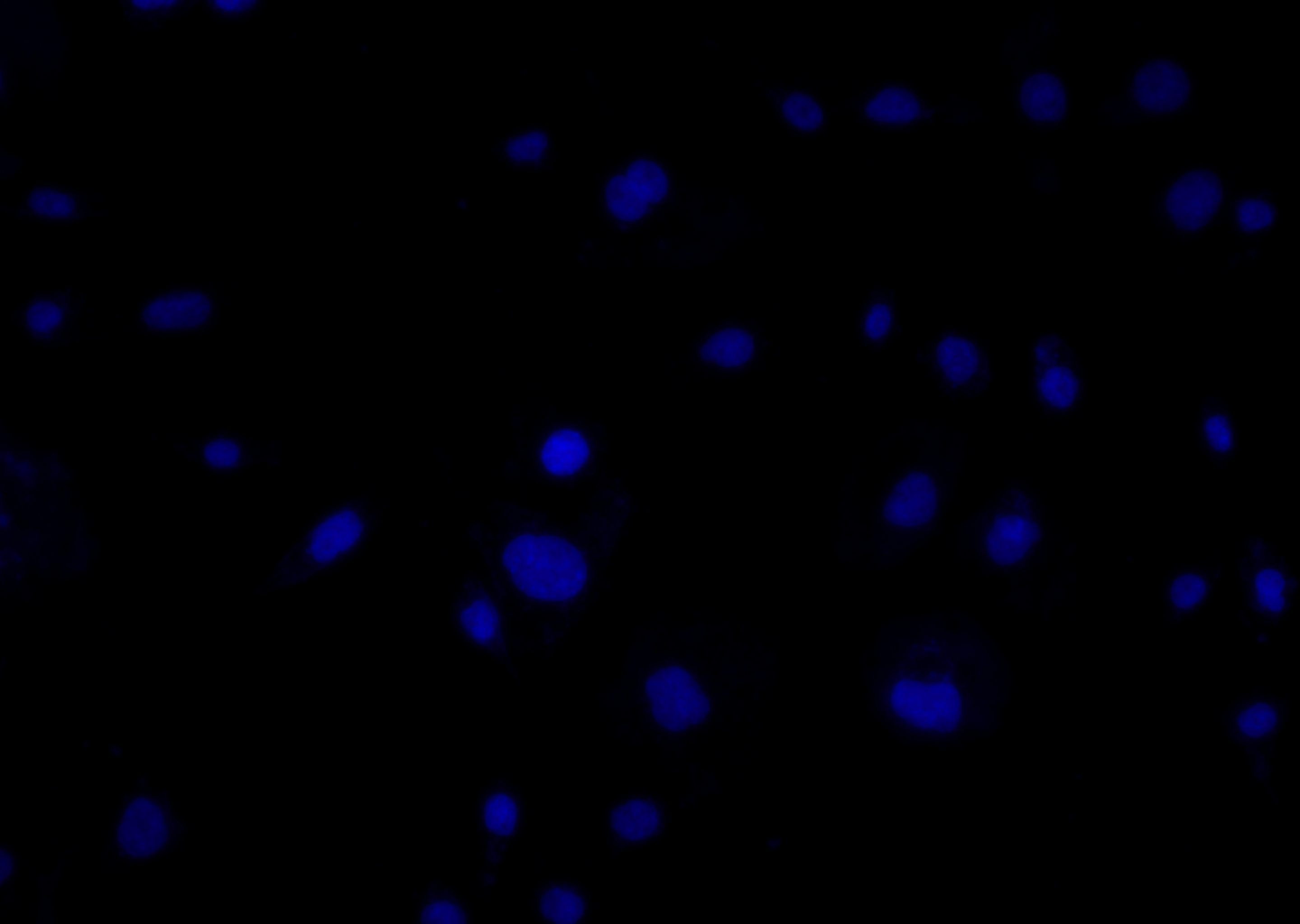

Supplement: Figure 5—source data 11. [file elife-97327-fig5-data11.zip › Figure 5-Source data 11/Snail-NC-DAPI.tif]

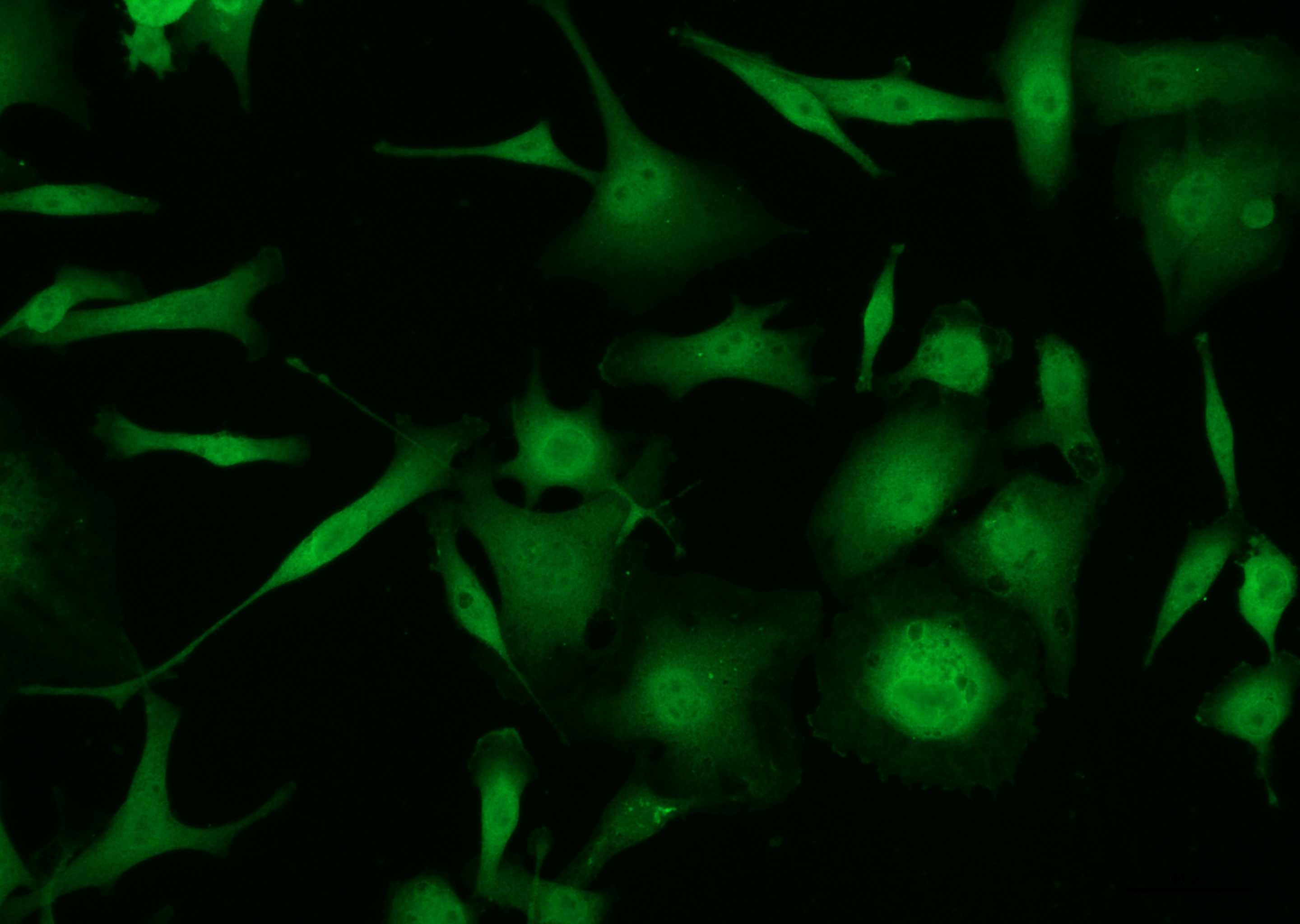

Supplement: Figure 5—source data 11. [file elife-97327-fig5-data11.zip › Figure 5-Source data 11/Snail-NC-FITC.tif]

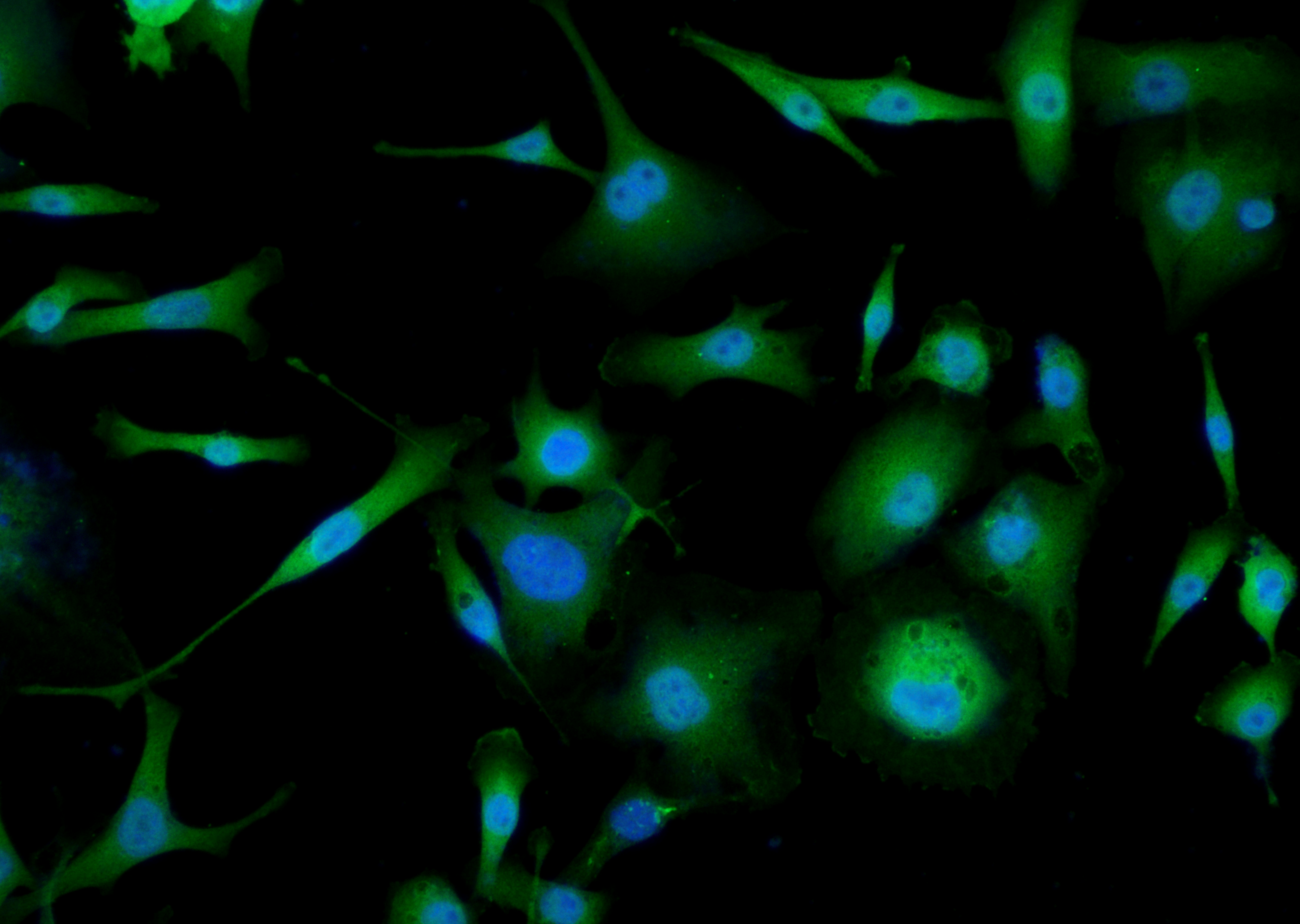

Supplement: Figure 5—source data 11. [file elife-97327-fig5-data11.zip › Figure 5-Source data 11/Snail-NC-merged.tif]

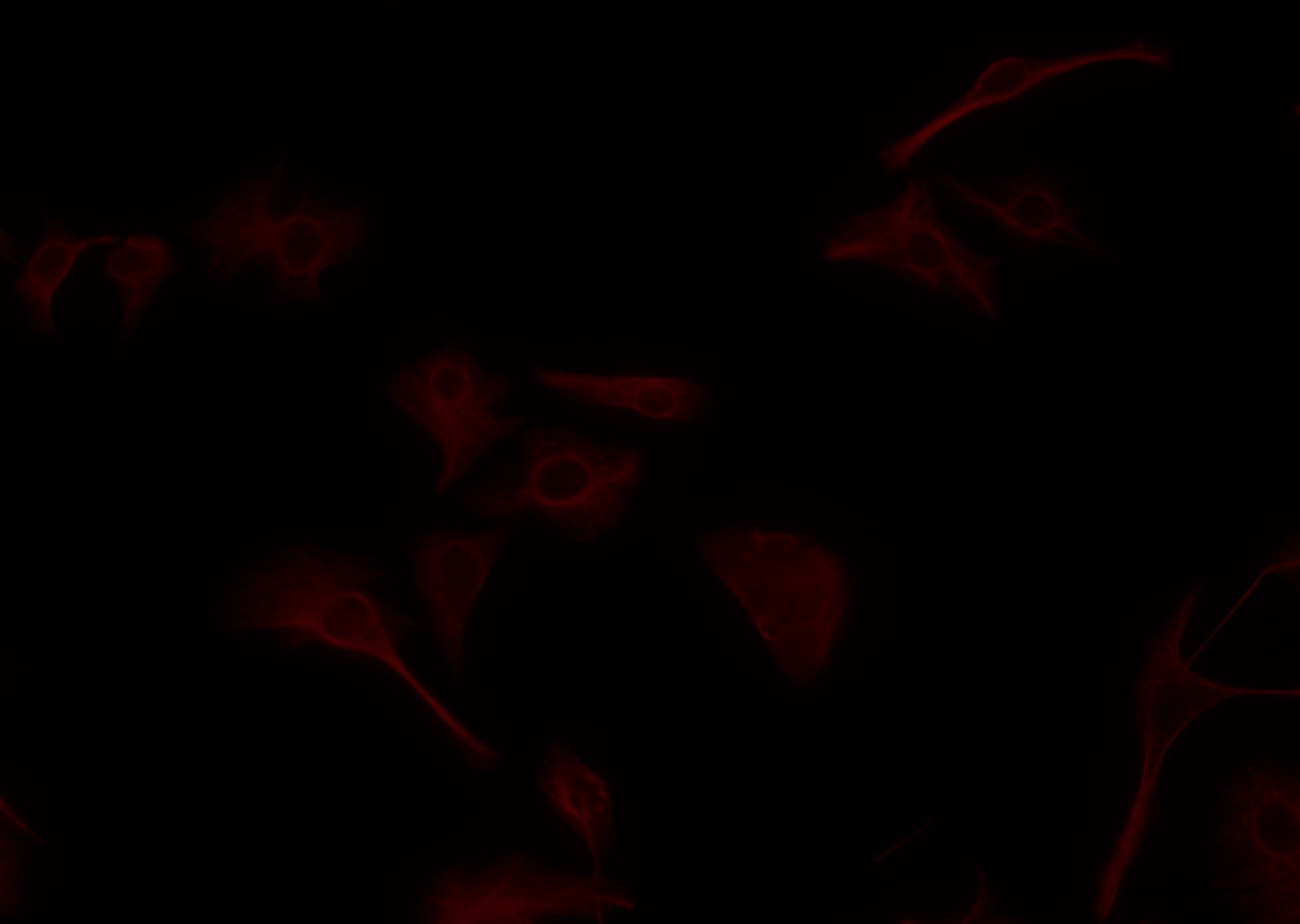

Supplement: Figure 5—source data 11. [file elife-97327-fig5-data11.zip › Figure 5-Source data 11/Vimentin-inhibitor-488.tif]

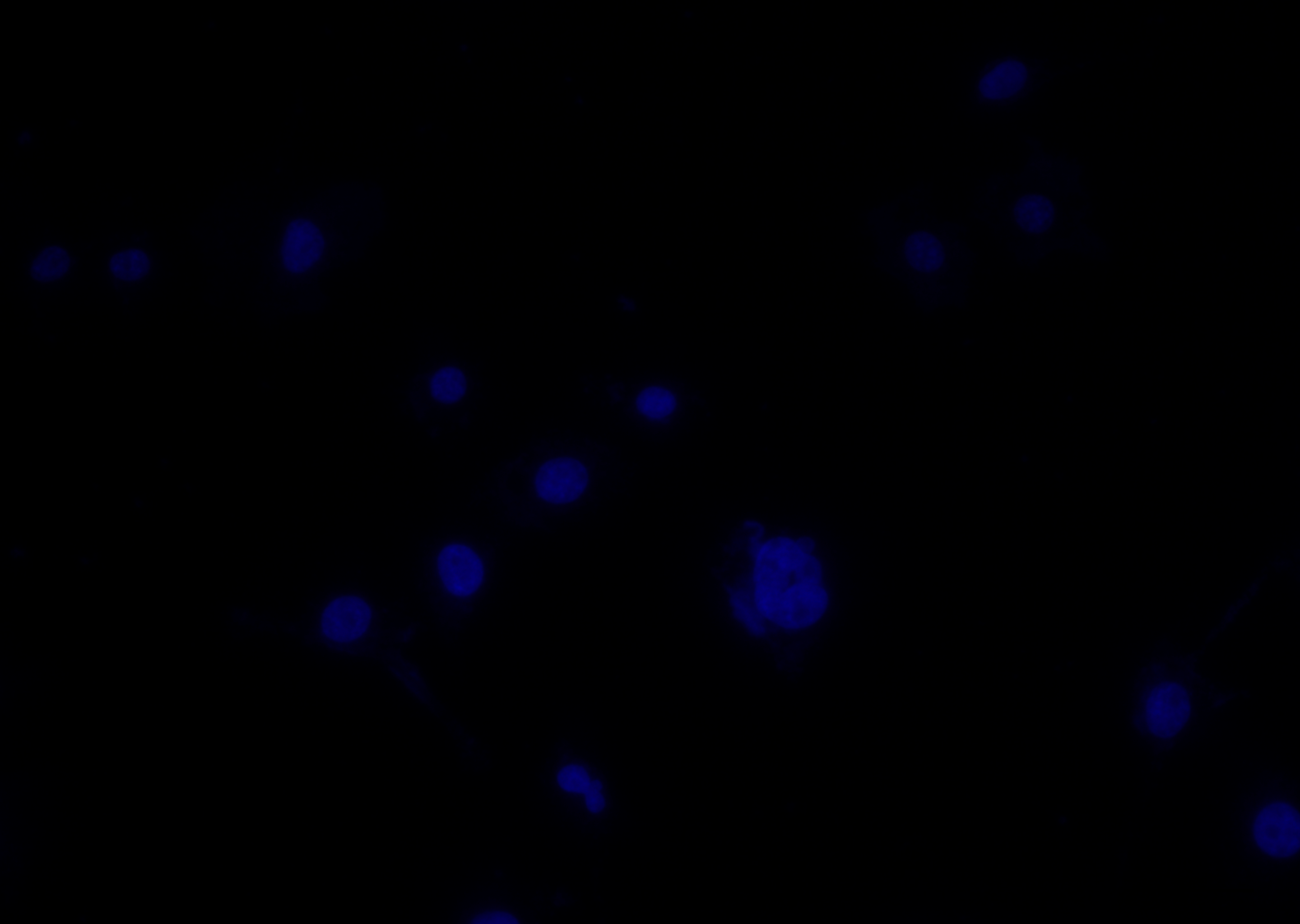

Supplement: Figure 5—source data 11. [file elife-97327-fig5-data11.zip › Figure 5-Source data 11/Vimentin-inhibitor-DAPI.tif]

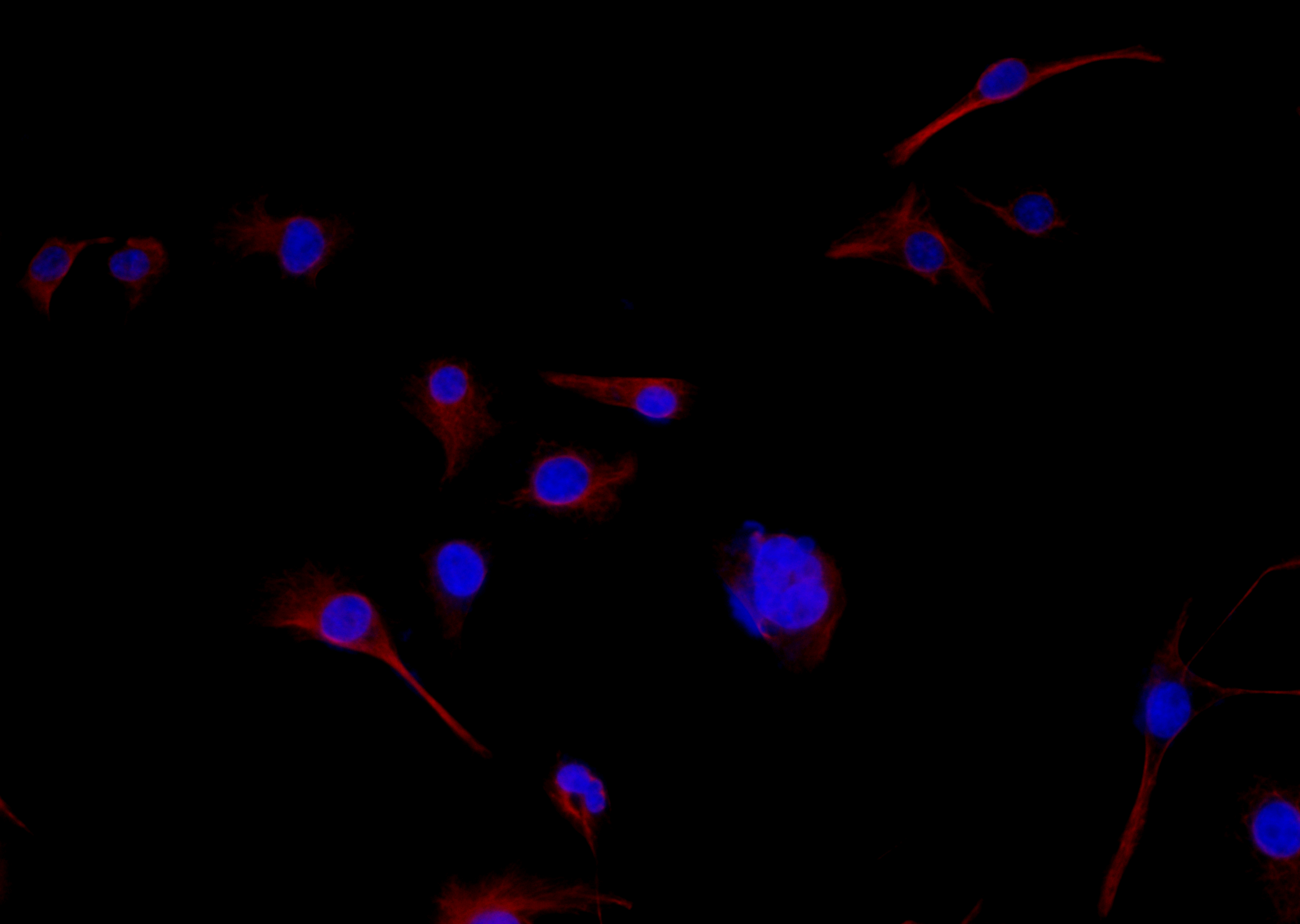

Supplement: Figure 5—source data 11. [file elife-97327-fig5-data11.zip › Figure 5-Source data 11/Vimentin-inhibitor-merged.tif]

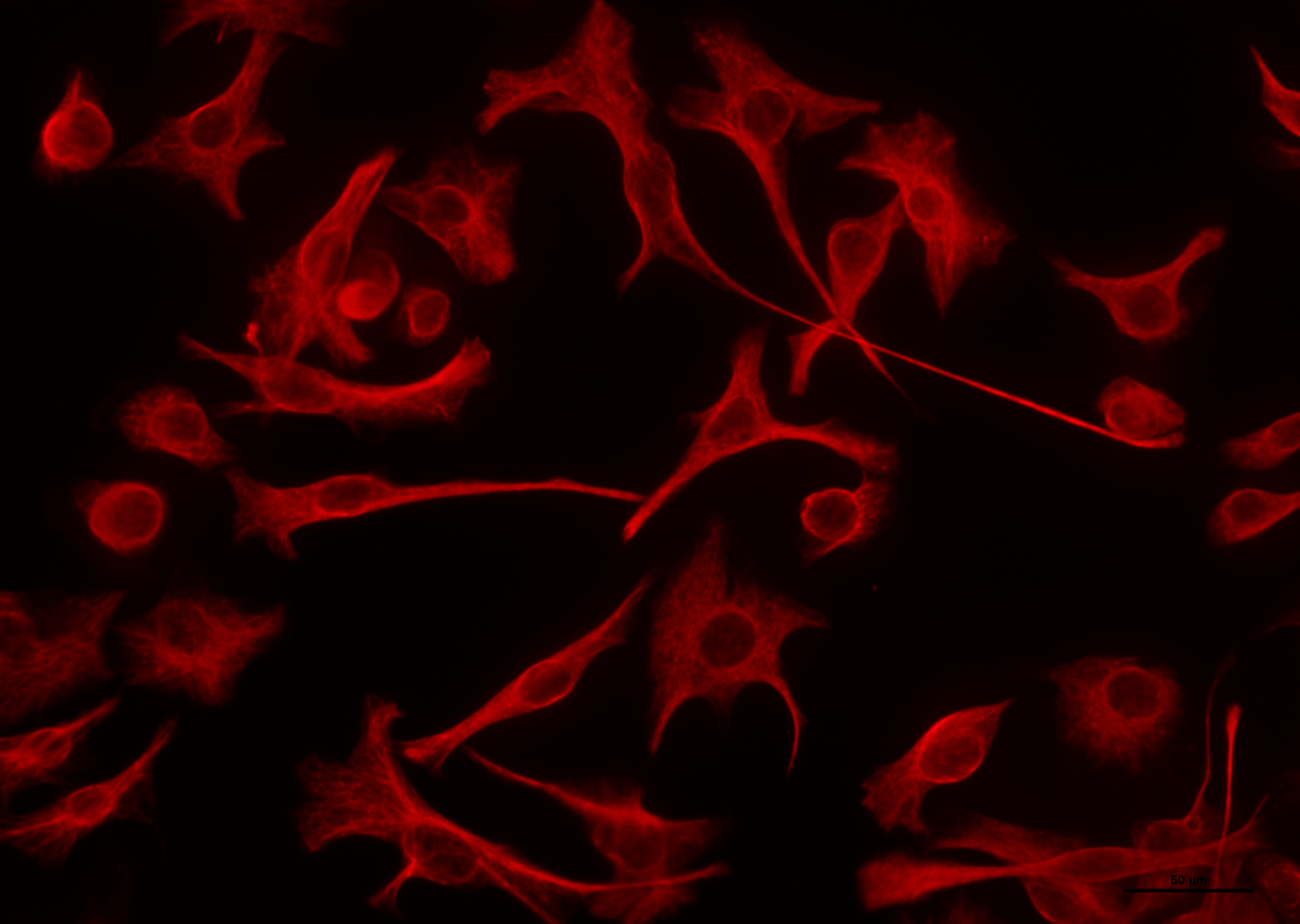

Supplement: Figure 5—source data 11. [file elife-97327-fig5-data11.zip › Figure 5-Source data 11/Vimentin-NC-488.tif]

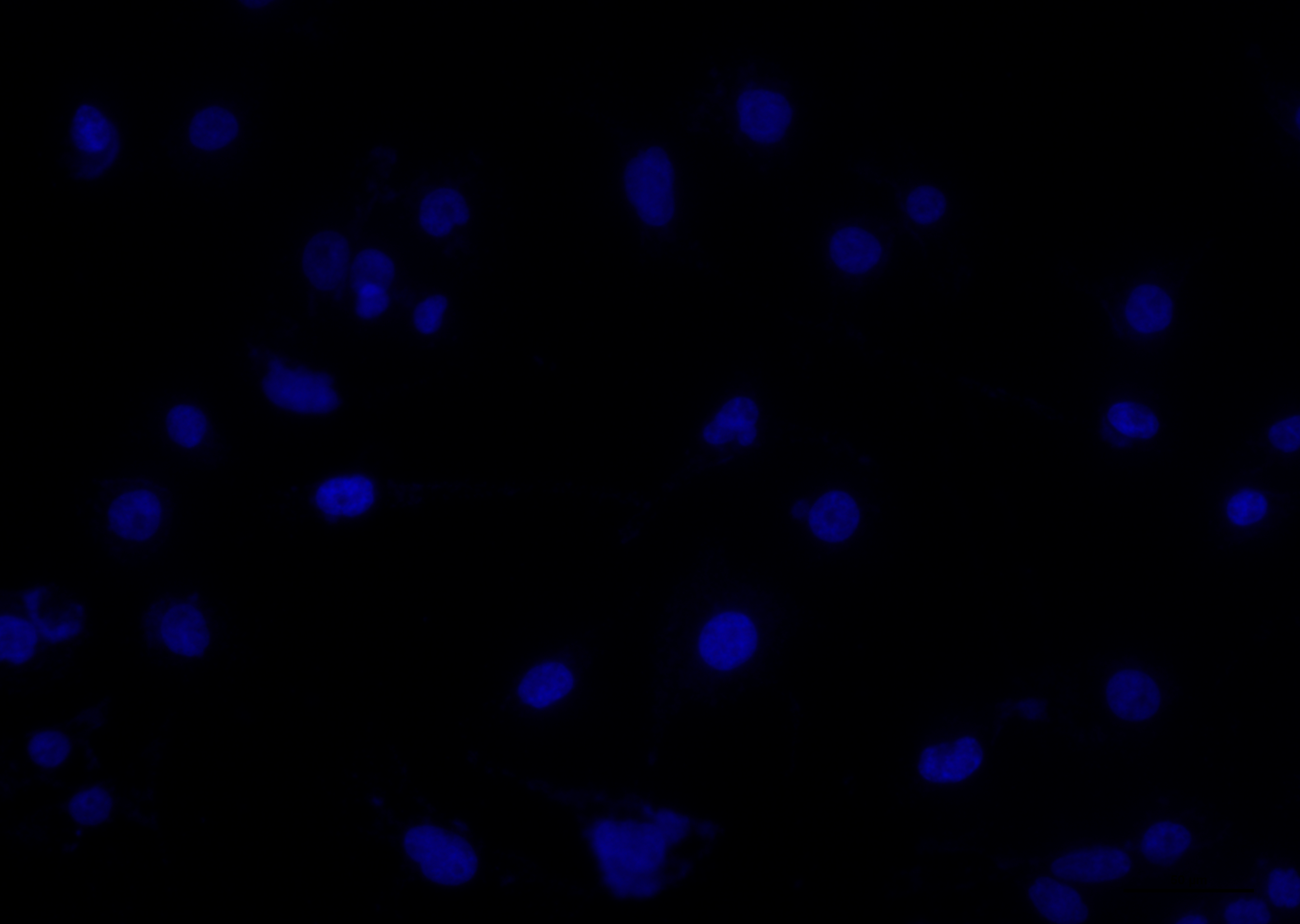

Supplement: Figure 5—source data 11. [file elife-97327-fig5-data11.zip › Figure 5-Source data 11/Vimentin-NC-DAPI.tif]

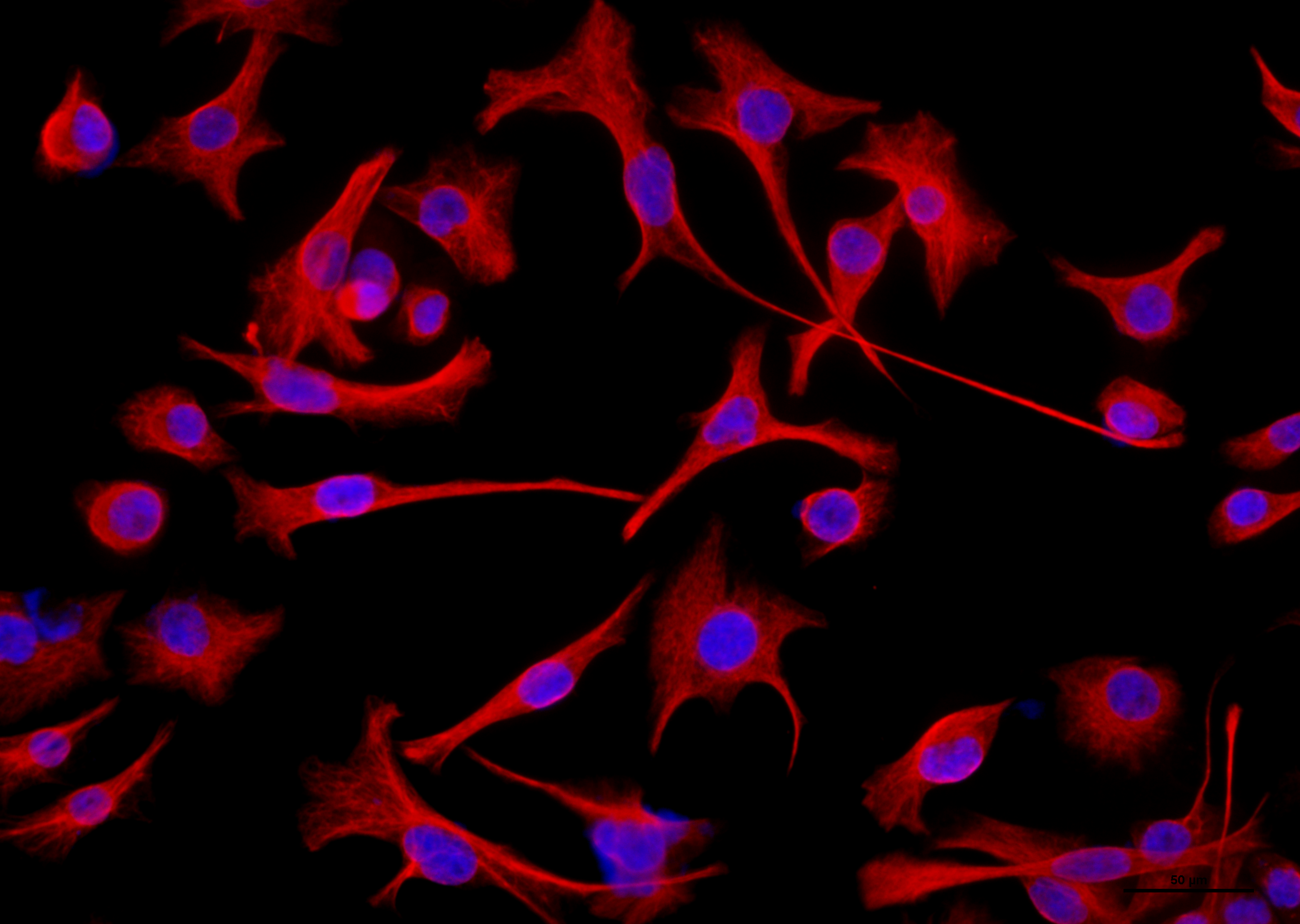

Supplement: Figure 5—source data 11. [file elife-97327-fig5-data11.zip › Figure 5-Source data 11/Vimentin-NC-merged.tif]

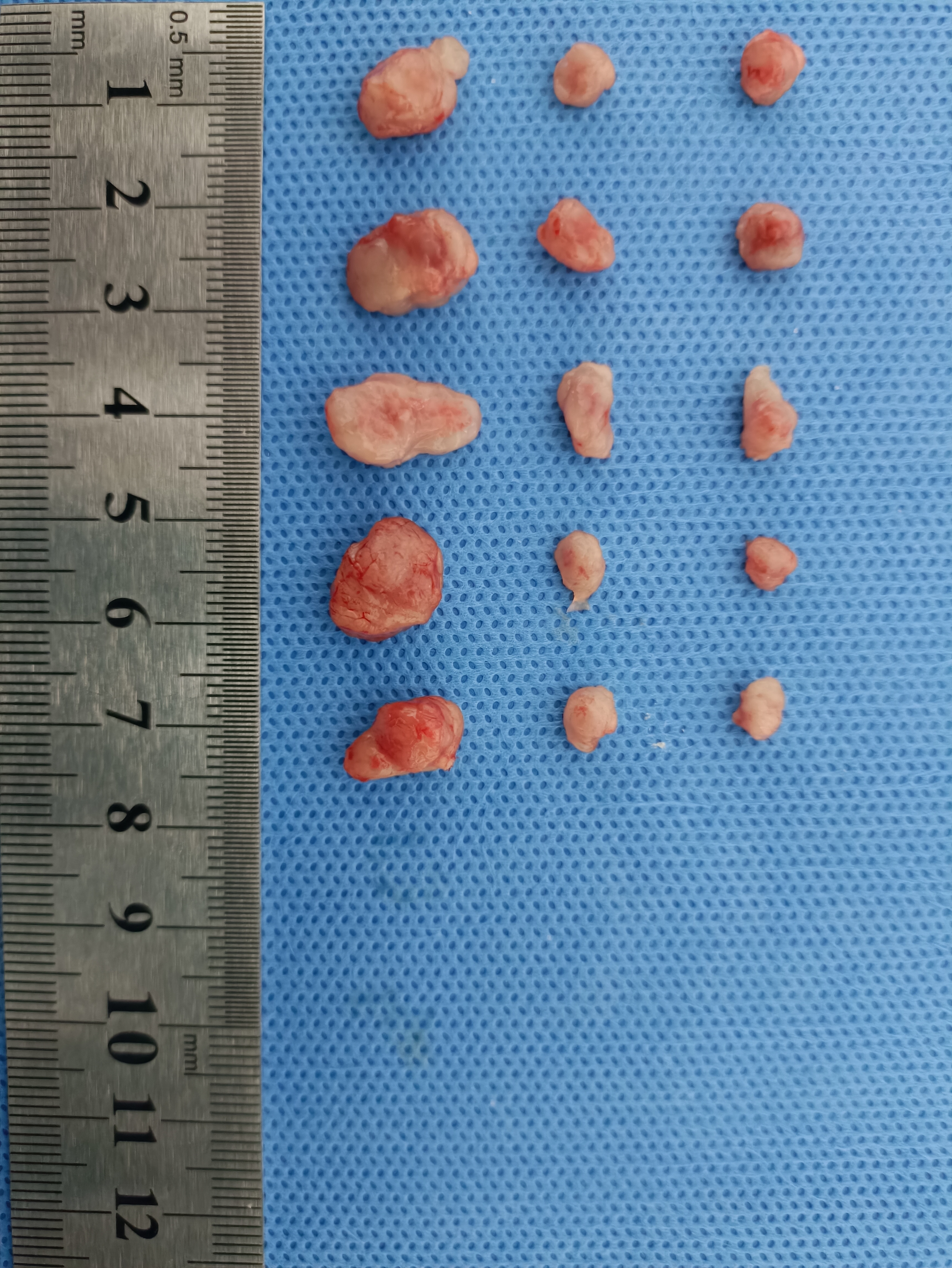

Supplement: Figure 6—source data 1. [file elife-97327-fig6-data1.zip › Figure 6-Source data 1/F6A.jpg]

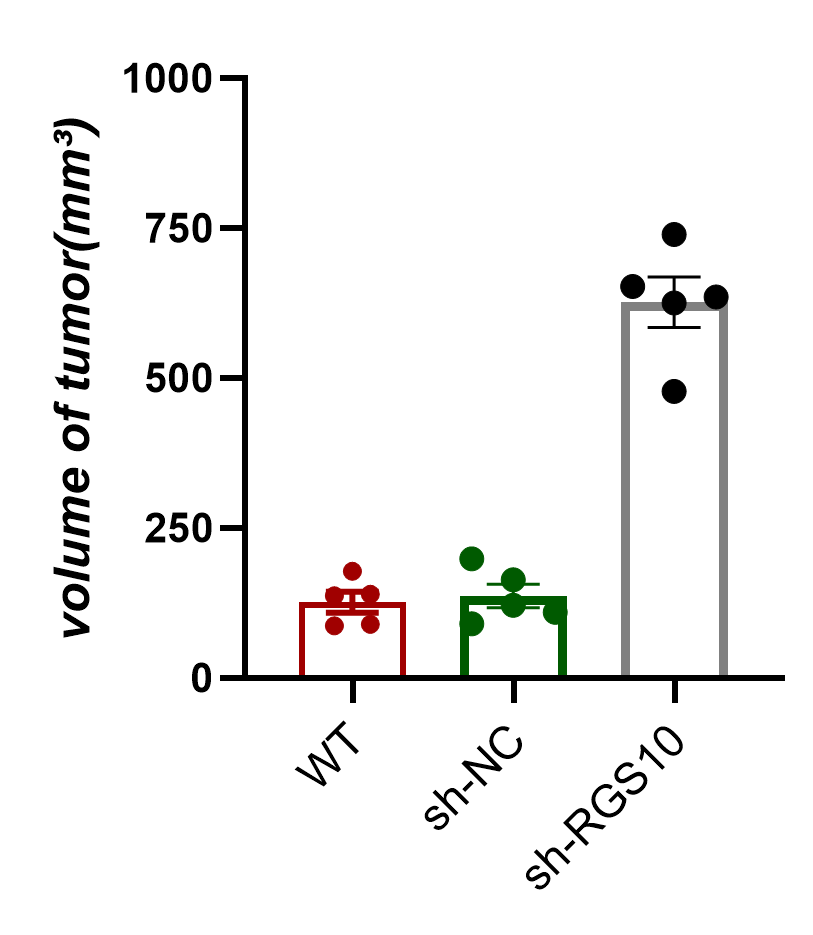

Supplement: Figure 6—source data 2. [file elife-97327-fig6-data2.zip › Figure 6-Source data 2/F6B-volume of tumor.tif]

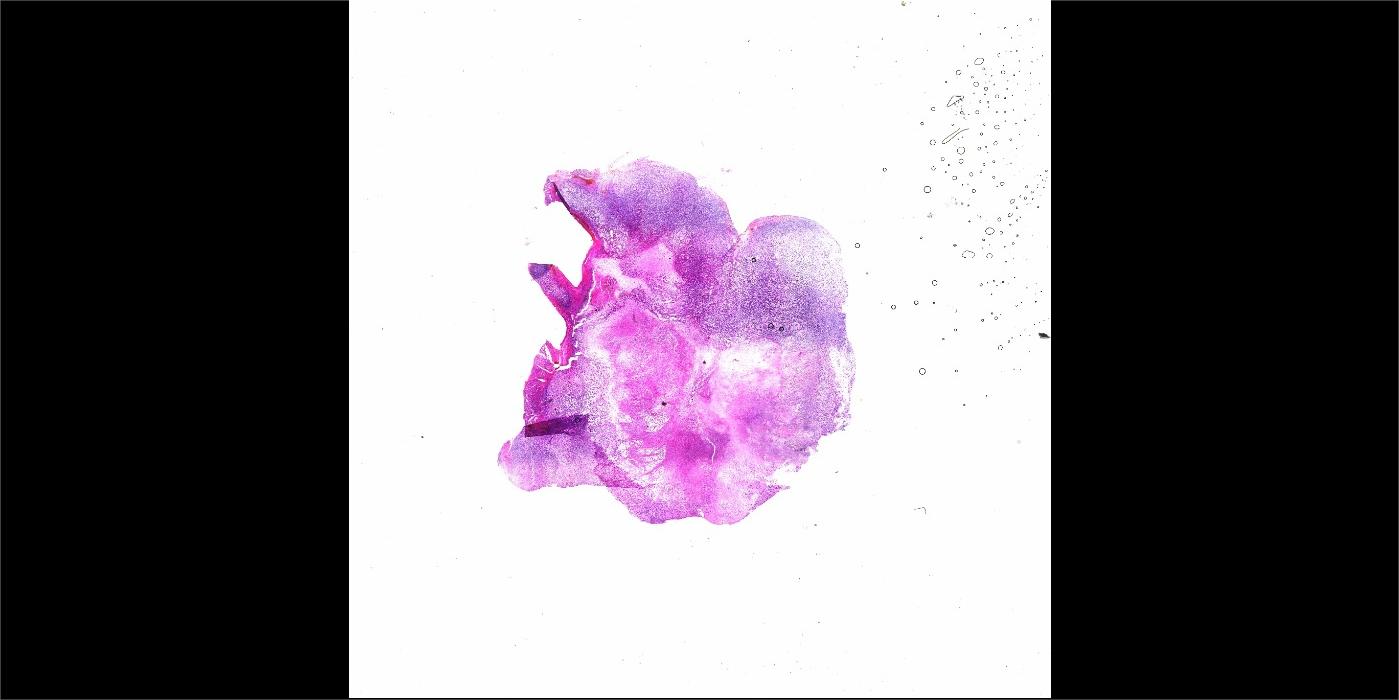

Supplement: Figure 6—source data 3. [file elife-97327-fig6-data3.zip › Figure 6-Source data 3/F6C-2023-HE -JY-123__A2.jpeg]

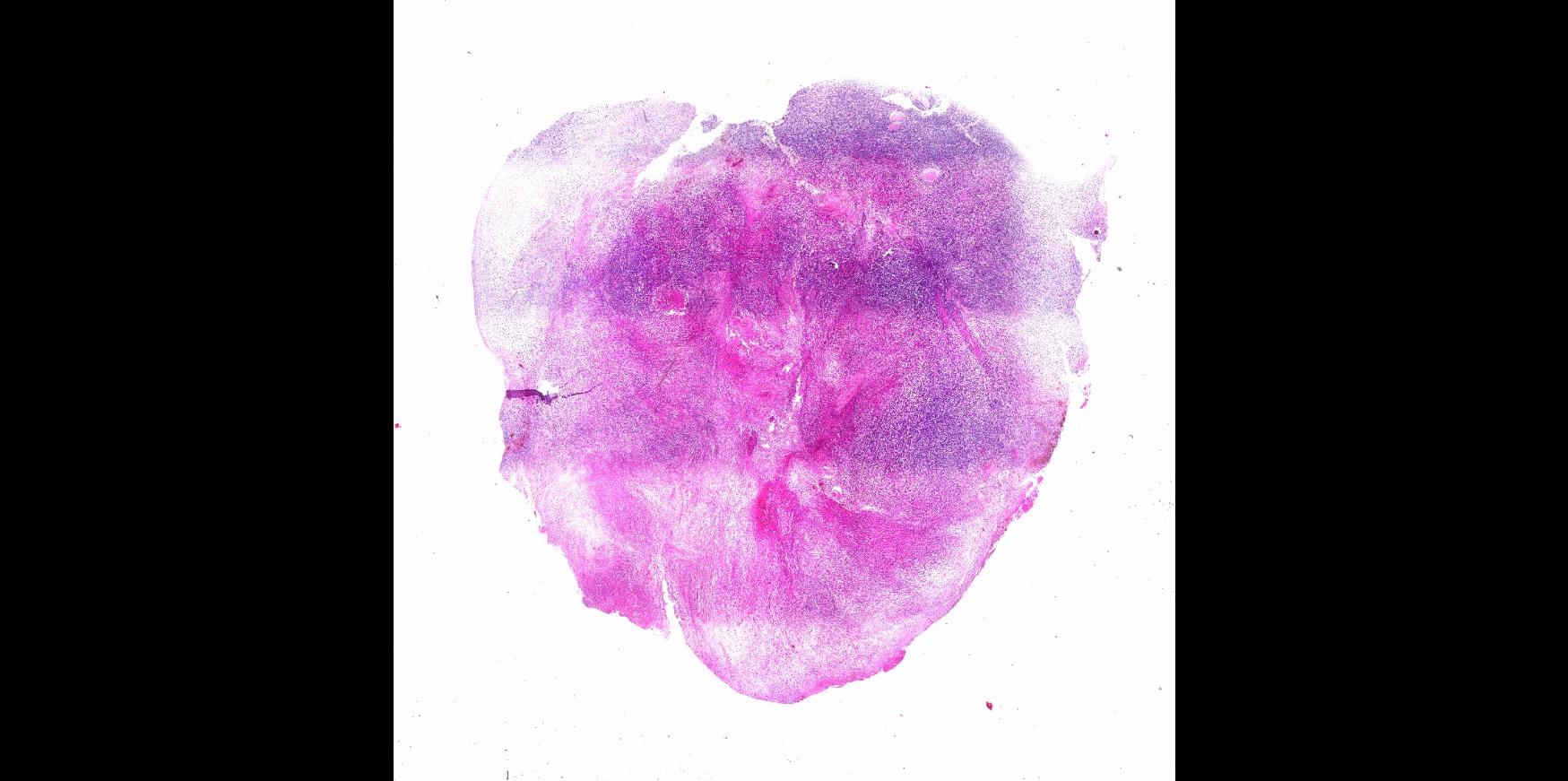

Supplement: Figure 6—source data 3. [file elife-97327-fig6-data3.zip › Figure 6-Source data 3/F6C-sh-RGS10.jpeg]

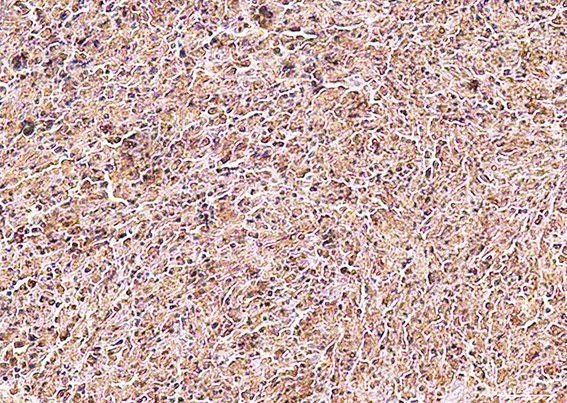

Supplement: Figure 6—source data 4. [file elife-97327-fig6-data4.zip › Figure 6-Source data 4/sh-NC-CDH10007.tif]

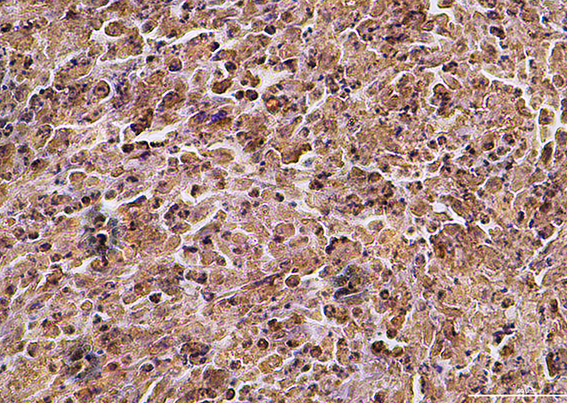

Supplement: Figure 6—source data 4. [file elife-97327-fig6-data4.zip › Figure 6-Source data 4/sh-NC-CDH10008.tif]

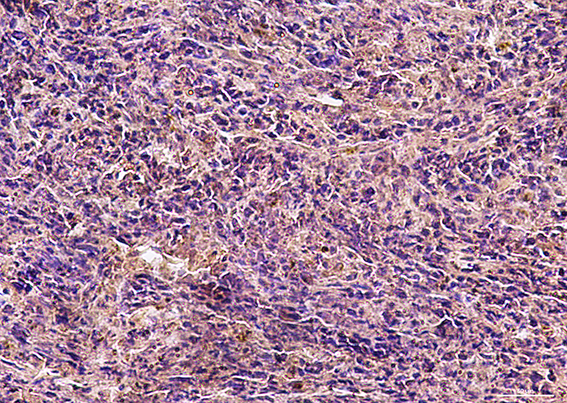

Supplement: Figure 6—source data 4. [file elife-97327-fig6-data4.zip › Figure 6-Source data 4/sh-NC-LCN20001.tif]

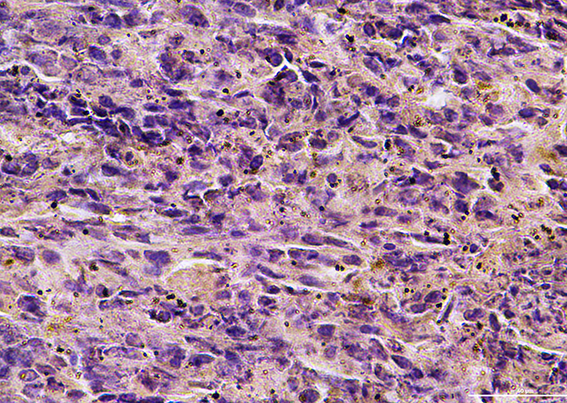

Supplement: Figure 6—source data 4. [file elife-97327-fig6-data4.zip › Figure 6-Source data 4/sh-NC-LCN20002.tif]

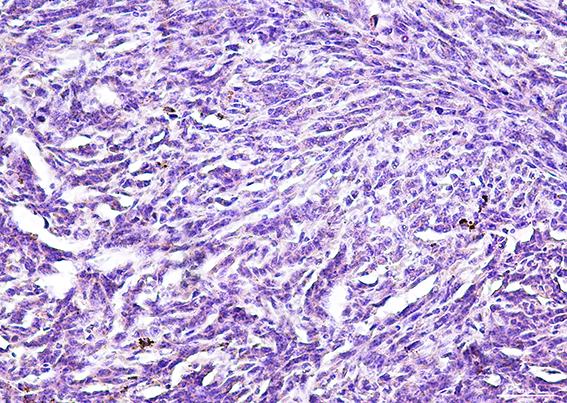

Supplement: Figure 6—source data 4. [file elife-97327-fig6-data4.zip › Figure 6-Source data 4/sh-NC-Snail0011.tif]

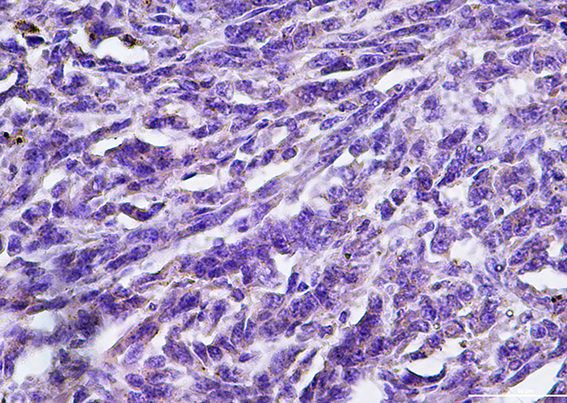

Supplement: Figure 6—source data 4. [file elife-97327-fig6-data4.zip › Figure 6-Source data 4/sh-NC-Snail0012.tif]

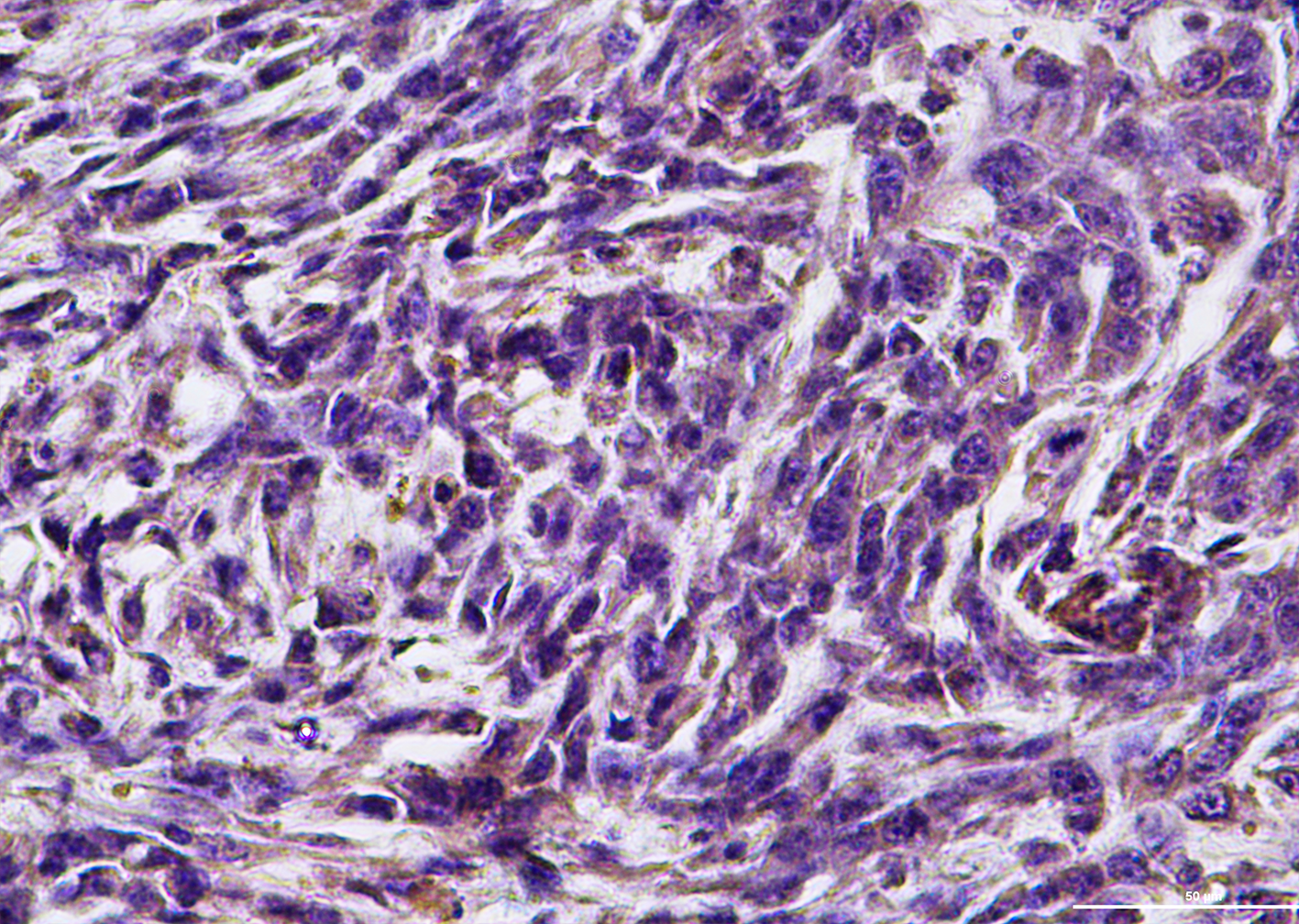

Supplement: Figure 6—source data 4. [file elife-97327-fig6-data4.zip › Figure 6-Source data 4/sh-NC-Vimentin0003.tif]

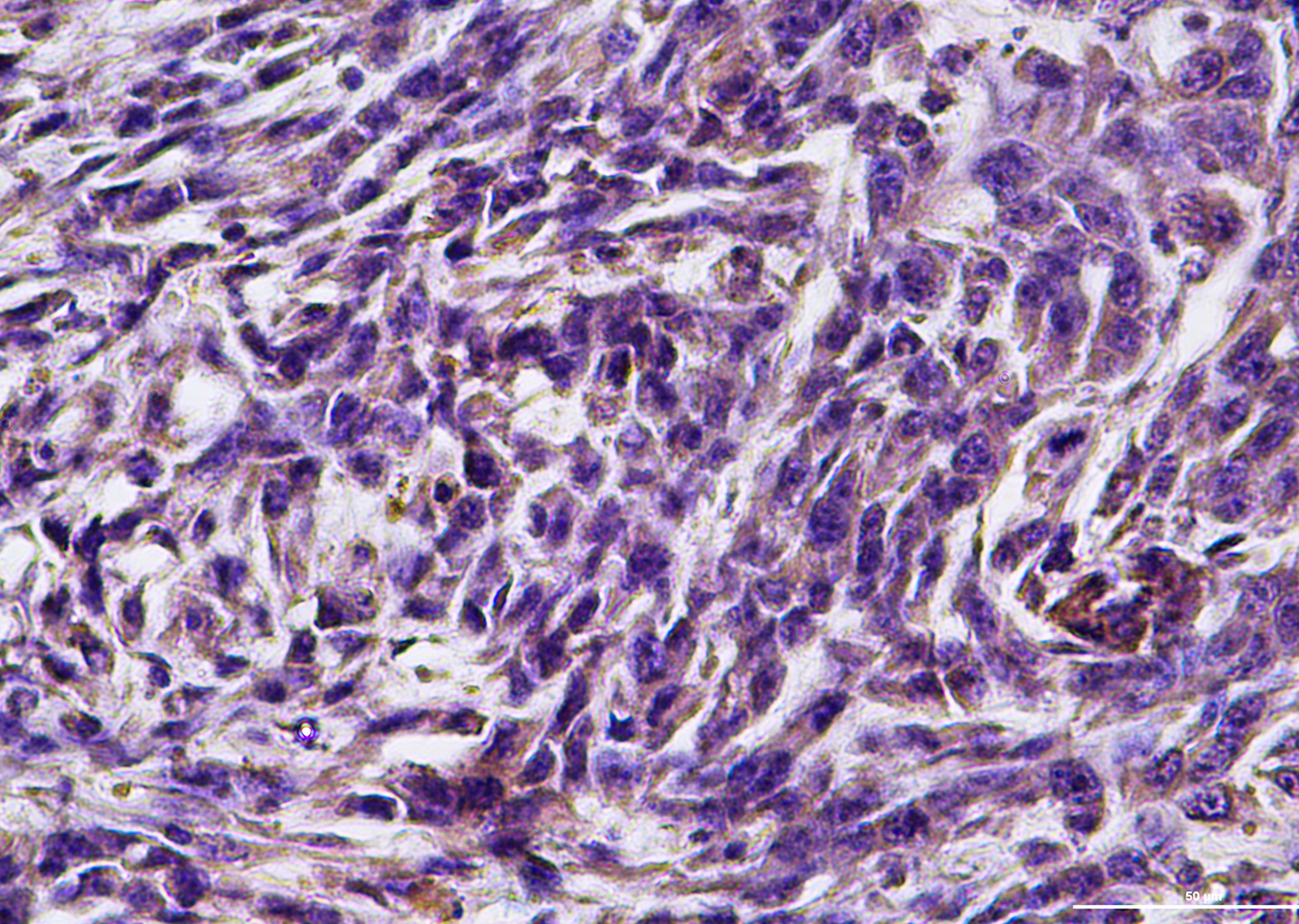

Supplement: Figure 6—source data 4. [file elife-97327-fig6-data4.zip › Figure 6-Source data 4/sh-NC-Vimentin0004.tif]

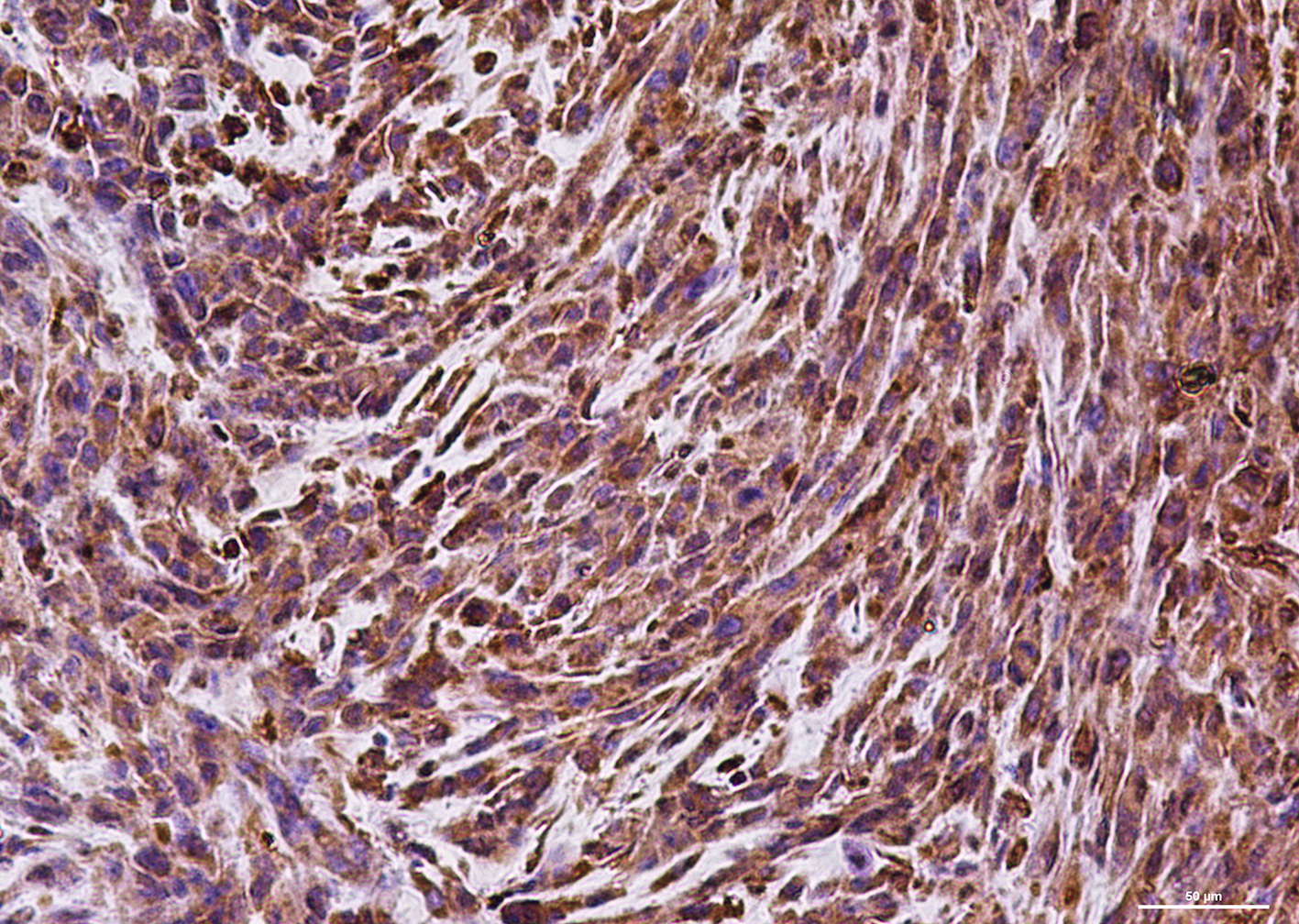

Supplement: Figure 6—source data 4. [file elife-97327-fig6-data4.zip › Figure 6-Source data 4/sh-RG1010-LCN20001.tif]

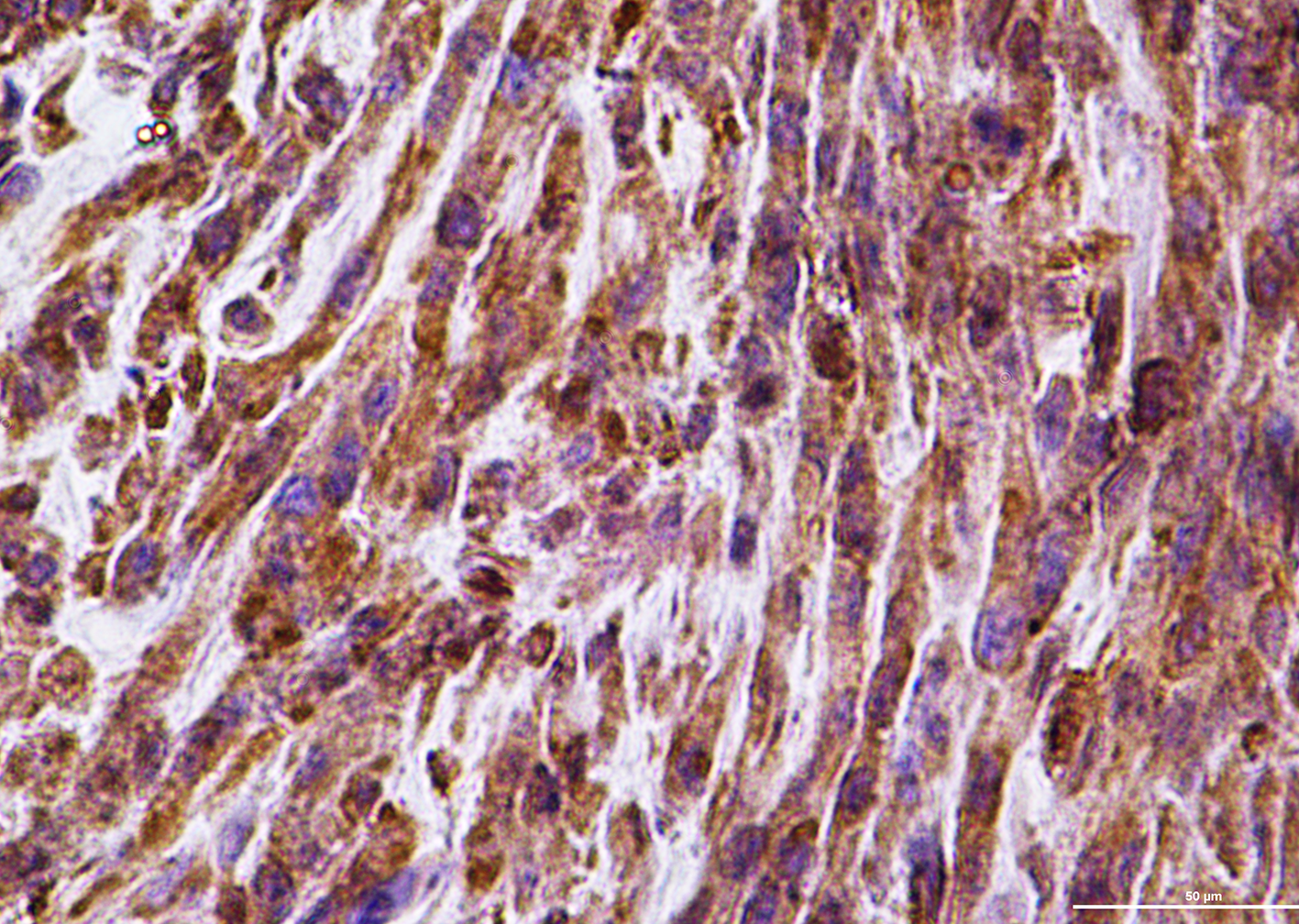

Supplement: Figure 6—source data 4. [file elife-97327-fig6-data4.zip › Figure 6-Source data 4/sh-RG1010-LCN20002.tif]

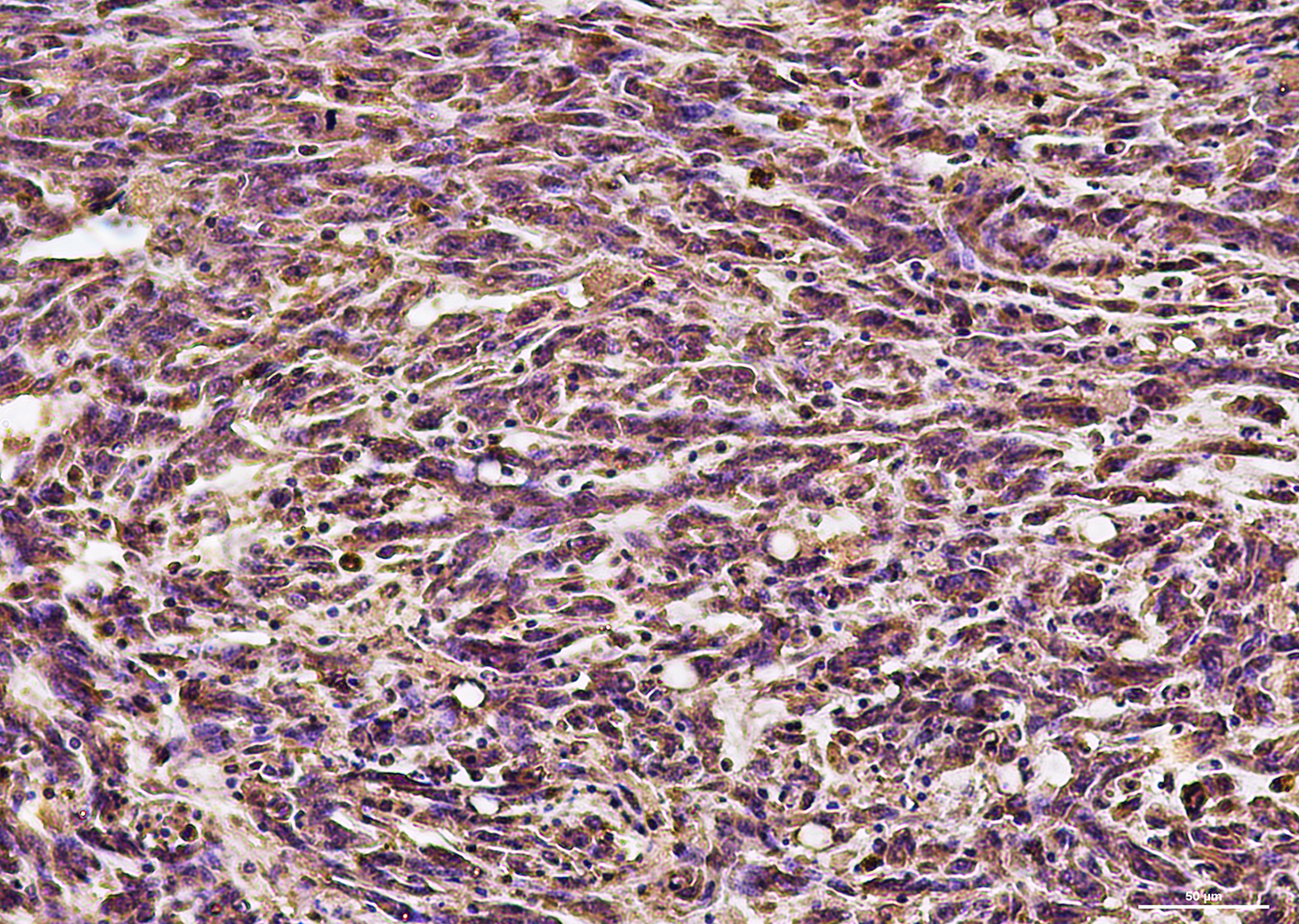

Supplement: Figure 6—source data 4. [file elife-97327-fig6-data4.zip › Figure 6-Source data 4/sh-RGS10-CDH10009.tif]

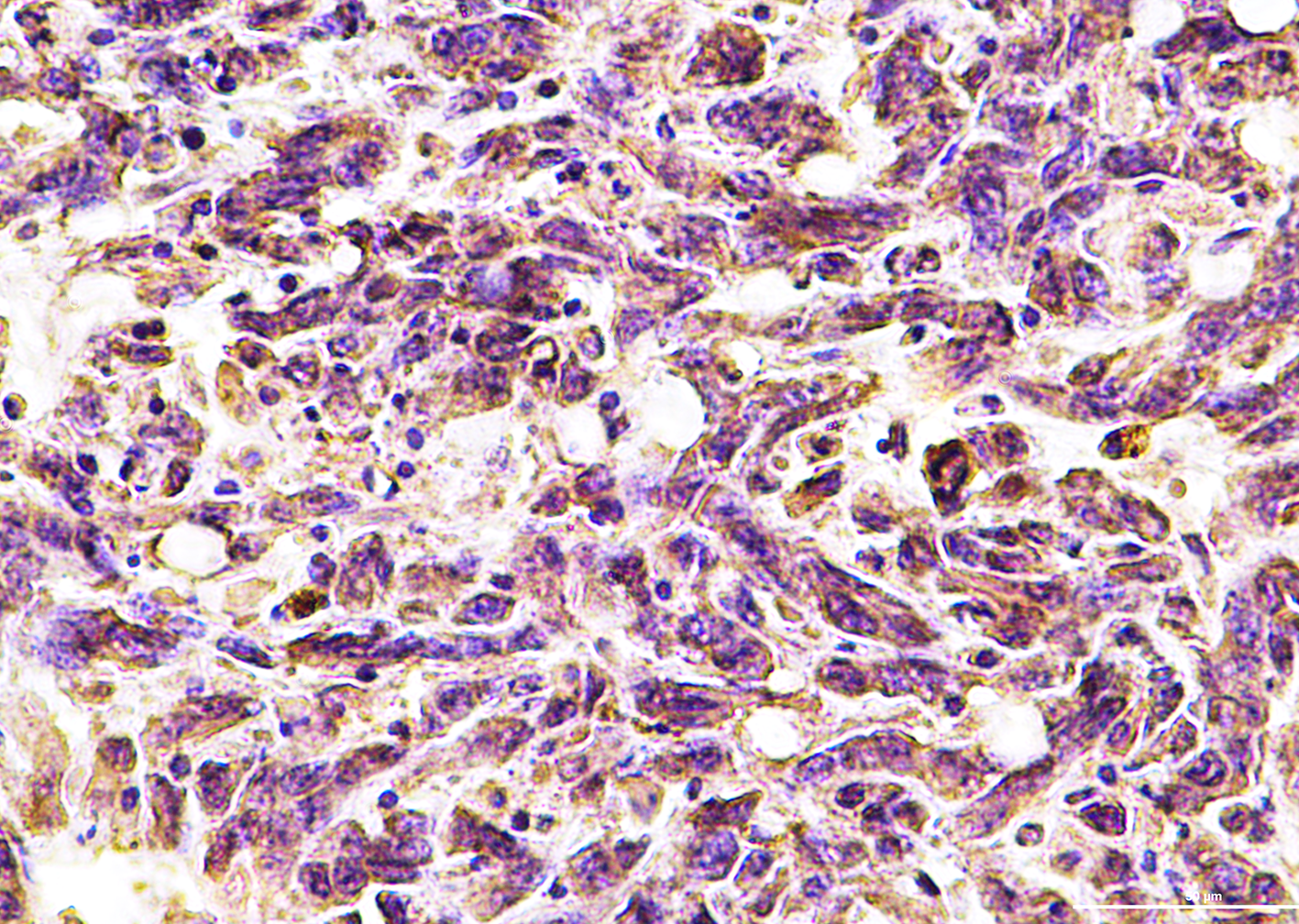

Supplement: Figure 6—source data 4. [file elife-97327-fig6-data4.zip › Figure 6-Source data 4/sh-RGS10-CDH10010.tif]

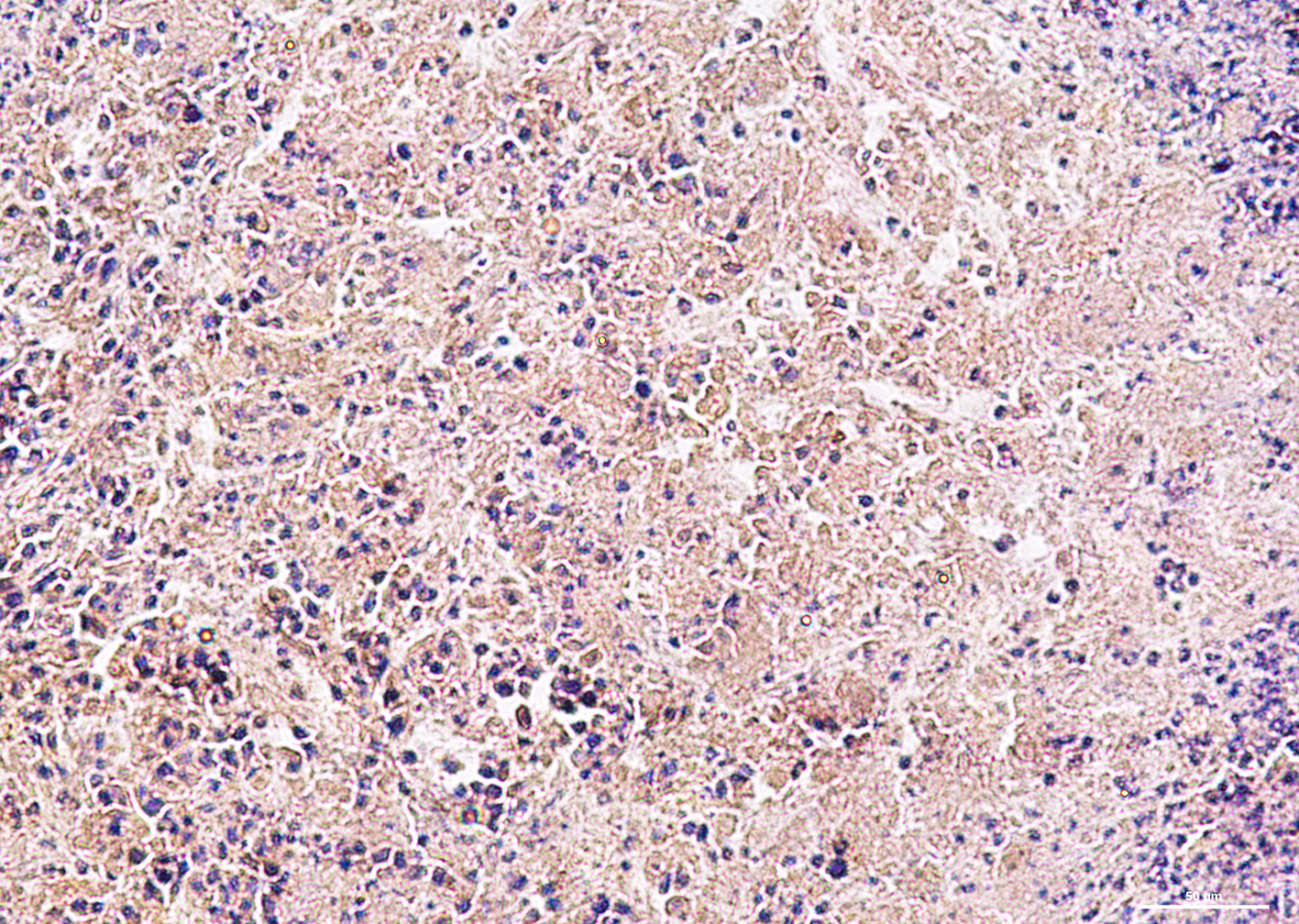

Supplement: Figure 6—source data 4. [file elife-97327-fig6-data4.zip › Figure 6-Source data 4/sh-RGS10-Snail0013+.tif]

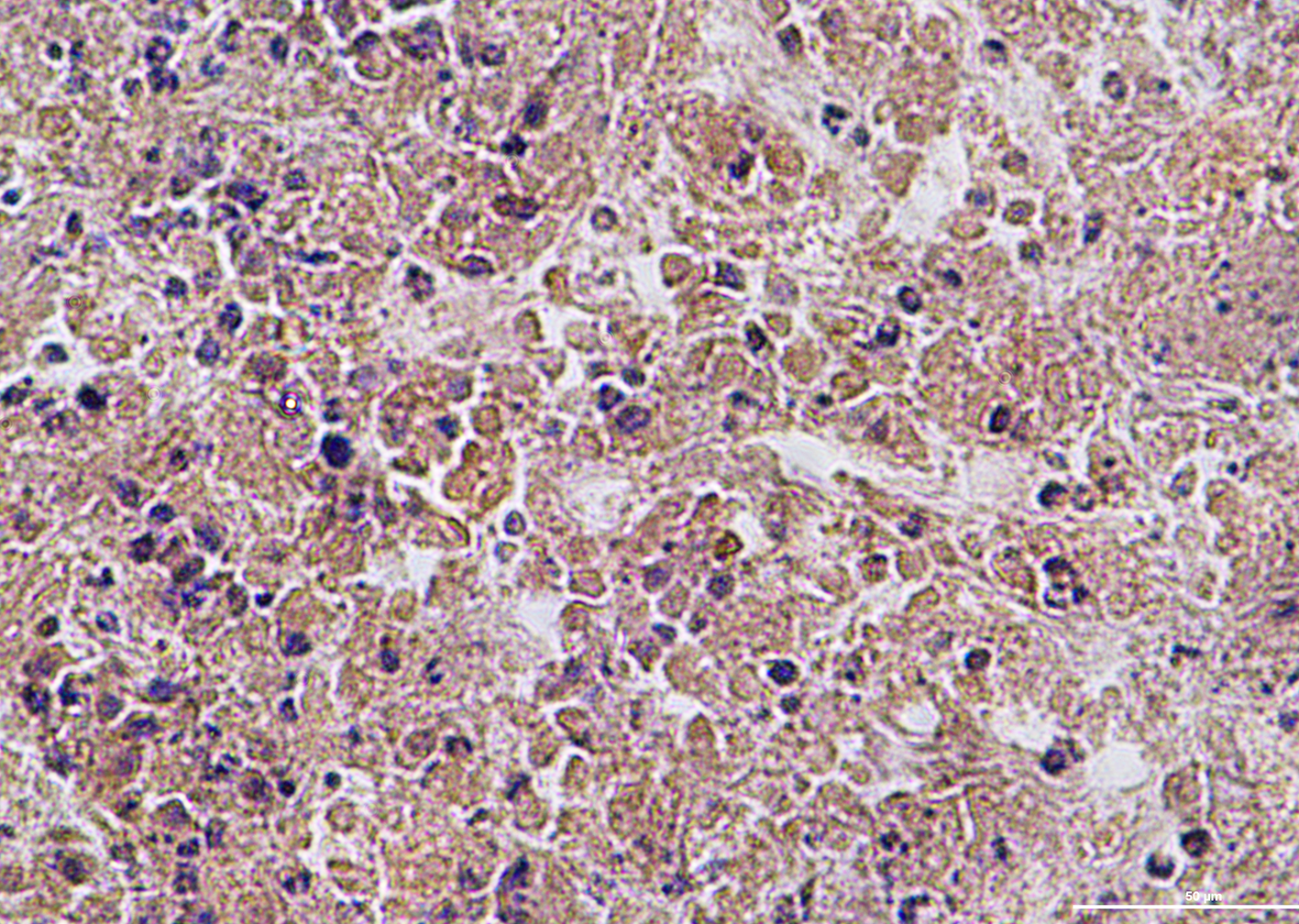

Supplement: Figure 6—source data 4. [file elife-97327-fig6-data4.zip › Figure 6-Source data 4/sh-RGS10-Snail0014+.tif]

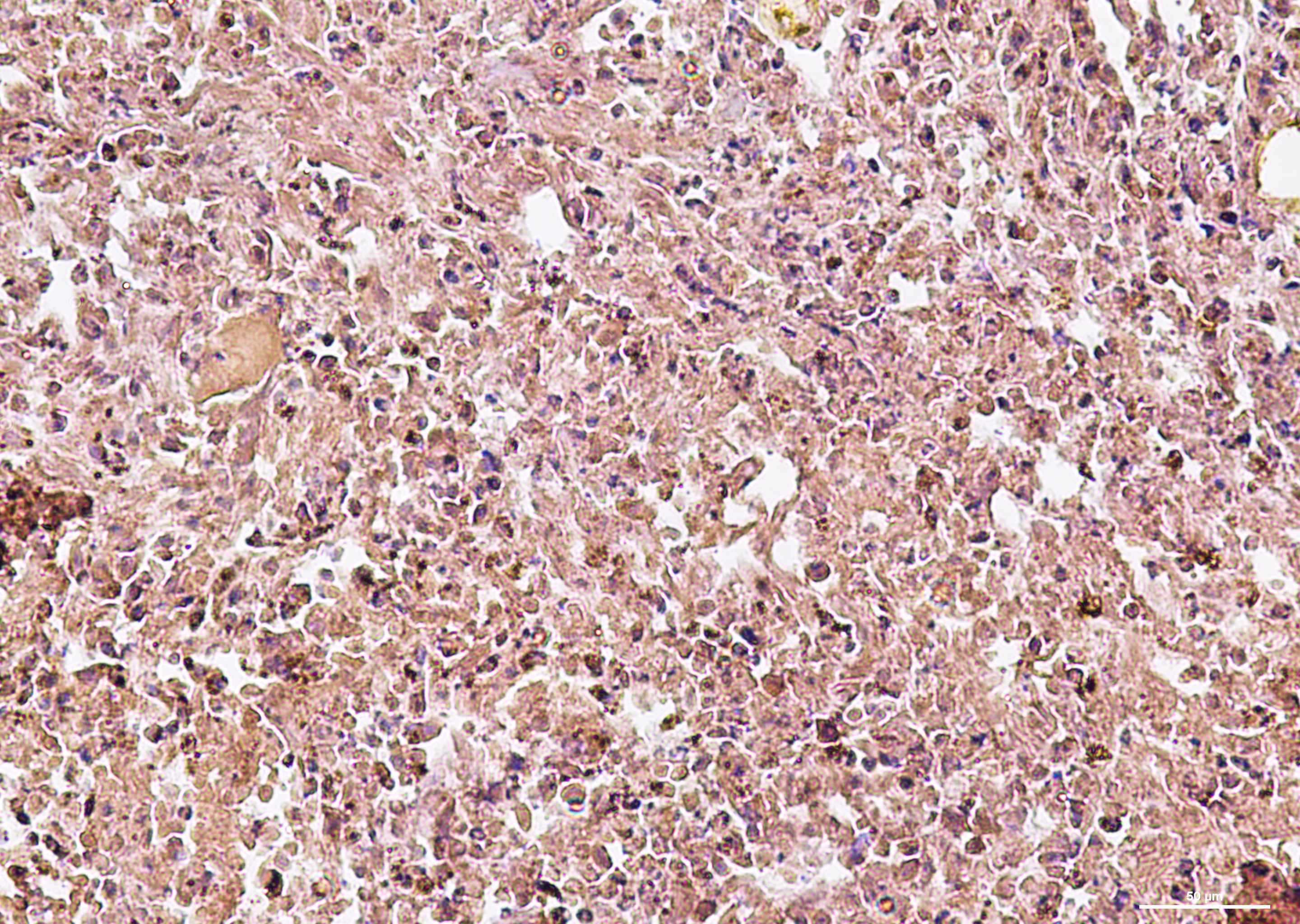

Supplement: Figure 6—source data 4. [file elife-97327-fig6-data4.zip › Figure 6-Source data 4/sh-RGS10-Vimentin0005.tif]

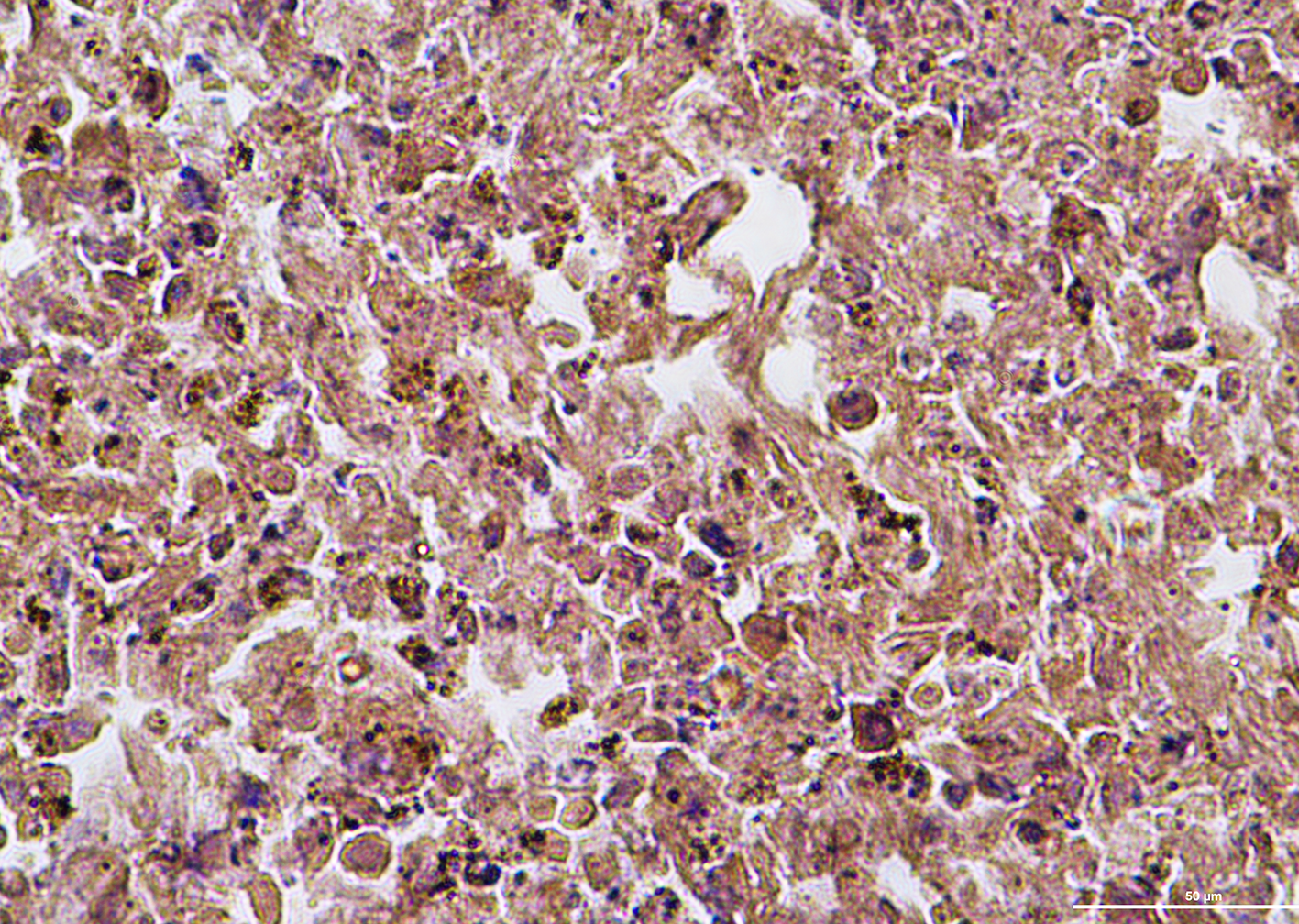

Supplement: Figure 6—source data 4. [file elife-97327-fig6-data4.zip › Figure 6-Source data 4/sh-RGS10-Vimentin0006.tif]
