# Supplementary material for: RGS10 deficiency facilitates distant metastasis by inducing epithelial–mesenchymal transition in breast cancer
Source: eLife. 2024 Aug 15;13:RP97327. doi: 10.7554/eLife.97327 (PMC11326775; doi:10.7554/eLife.97327)
Supplement: Peer review history

# Editor report: eLife assessment

**DOI:** 10.7554/eLife.97327.3.sa0

**Authors:** Yaoting Ji

**Affiliations:** Wuhan University China

**Keywords:** Solid, Valuable

This **valuable** article first demonstrated that RGS10 was identified as a biomarker to evaluate the prognosis of breast cancer. To prevent the loss of RGS10, it theoretically provides a new strategy for the treatment of breast cancer. The evidence supporting the claims of the authors is **solid**, although inclusion of a larger number of patient samples and an animal model would have strengthened the study. The work will be of interest to clinicians working on breast cancer.

# Referee report: Reviewer #1 (Public Review)

**DOI:** 10.7554/eLife.97327.3.sa1

The paper has shown the expression of RGS10 is related to the molecular subtype, distant metastasis, and survival status of breast cancer. The study utilizes bioinformatic analyses, human tissue samples, and in vitro and in vivo experiments which strengthen the data. RGS10 was validated to inhibit EMT through a novel mechanism dependent on LCN2 and miR-539-5p, thereby reducing cancer cell proliferation, colony formation, invasion, and migration. The study elaborated on the function of RGS10 in influencing the prognosis and biological behavior which could be considered as a potential drug target in breast cancer.

# Referee report: Reviewer #2 (Public Review)

**DOI:** 10.7554/eLife.97327.3.sa2

Liu et al., by focusing on the regulation of G protein-signaling 10 (RGS10), reported that RGS10 expression was significantly lower in patients with breast cancer, compared with normal adjacent tissue. Genetic inhibition of RGS10 caused epithelial-mesenchymal transition, and enhanced cell proliferation, migration, and invasion, respectively. These results suggest an inhibitory role of RGS10 in tumor metastasis. Furthermore, bioinformatic analyses determined signaling cascades for RGS10-mediated breast cancer distant metastasis. More importantly, both in vitro and in vivo studies evidenced that alteration of RGS10 expression by modulating its upstream regulator miR-539-5p affects breast cancer metastasis. Altogether, these findings provide insight into the pathogenesis of breast tumors and hence identify potential therapeutic targets in breast cancer.

The conclusions of this study are mostly well supported by data.

# Referee report: Reviewer #3 (Public Review)

**DOI:** 10.7554/eLife.97327.3.sa3

Distant metastasis is the major cause of death in patients with breast cancer. In this manuscript, Liu et al. show that RGS10 deficiency elicits distant metastasis via epithelial-mesenchymal transition in breast cancer. As a prognostic indicator of breast cancer, RGS10 regulates the progress of breast cancer and affects tumor phenotypes such as epithelial-mesenchymal transformation, invasion, and migration. The conclusions of this paper are mostly well supported by data.

# Author comment: Author response

**DOI:** 10.7554/eLife.97327.3.sa4

**Authors:** Yang Liu, Yi Jiang, Peng Qiu, Tie Ma, Yang Bai, Jiawen Bu, Yueting Hu, Ming Jin, Tong Zhu, Xi Gu

**Affiliations:** Shengjing Hospital of China Medical University Shenyang China; Shengjing Hospital of China Medical University Shenyang China; Shengjing Hospital of China Medical University Shenyang China; Shengjing Hospital of China Medical University Shenyang China; Shengjing Hospital of China Medical University shenyang China; Shengjing Hospital of China Medical Universit Shenyang China; Shengjing Hospital of China Medical University Shenyang China; Shengjing Hospital of China Medical University Shenyang China; Panjin Central Hospital Panjin China; China Medical University Shenyang China

The following is the authors’ response to the original reviews.

**eLife assessment**

This study presents a valuable finding on the mechanism to promote distant metastasis in breast cancer. The evidence supporting the claims of the authors is convincing. The work will be of interest to medical biologists working on breast cancer.

**Public Reviews:**

**Reviewer #1 (Public Review):**

Strengths

The paper has shown the expression of RGS10 is related to the molecular subtype, distant metastasis, and survival status of breast cancer. The study utilizes bioinformatic analyses, human tissue samples, and in vitro and in vivo experiments which strengthen the data. RGS10 was validated to inhibit EMT through a novel mechanism dependent on LCN2 and miR-539-5p, thereby reducing cancer cell proliferation, colony formation, invasion, and migration. The study elaborated the function of RGS10 in influencing the prognosis and biological behavior which could be considered as a potential drug target in breast cancer.

Weakness

The mechanism by which the miR-539-5p/RGS10/LCN2 axis may be related to the prognosis of cancer patients still needs to be elucidated. In addition, the sample size used is relatively limited. Especially, if further exploration of the related pathways and mechanisms of LCN2 can be carried out by using organoid models, as well as the potential of RGS10 as a biomarker for further clinical translation to verify its therapeutic target effect, which will make the data more convincing.

Answer: Thank you for your comments and suggestions. In future research, we will utilize large clinical cohorts and organoid models to further explore relevant research mechanisms.

**Reviewer #2 (Public Review):**

Liu et al., by focusing on the regulation of G protein-signaling 10 (RGS10), reported that RGS10 expression was significantly lower in patients with breast cancer, compared with normal adjacent tissue. Genetic inhibition of RGS10 caused epithelial-mesenchymal transition, and enhanced cell proliferation, migration, and invasion, respectively. These results suggest an inhibitory role of RGS10 in tumor metastasis. Furthermore, bioinformatic analyses determined signaling cascades for RGS10-mediated breast cancer distant metastasis. More importantly, both in vitro and in vivo studies evidenced that alteration of RGS10 expression by modulating its upstream regulator miR-539-5p affects breast cancer metastasis. Altogether, these findings provide insight into the pathogenesis of breast tumors and hence identify potential therapeutic targets in breast cancer.

The conclusions of this study are mostly well supported by data. However, there is a weakness in the study that needs to be clarified.

In Figure 2A, although some references supported that SKBR3 and MCF-7 possess poorly aggressive and less invasive abilities, examining only RGS10 expression in those cells, it could not be concluded that 'RGS10 acts as a tumor suppressor in breast cancer'. It would be better to introduce a horizontal comparison of the invasive ability of these 3 types of cells using an invasion assay.

Answer: Thank you for your comments and suggestions. MDA-MB-231, SKBR3, and MCF-7 originate from triple-negative breast cancer (high invasiveness), Her-2 receptor overexpression (relatively weak invasiveness), and luminal type breast cancer (relatively weak invasiveness) separately. Previous studies have demonstrated the invasive ability of these 3 types of cells. (PMID: 34390568)

**Reviewer #3 (Public Review):**

Distant metastasis is the major cause of death in patients with breast cancer. In this manuscript, Liu et al. show that RGS10 deficiency elicits distant metastasis via epithelial-mesenchymal transition in breast cancer. As a prognostic indicator of breast cancer, RGS10 regulates the progress of breast cancer and affects tumor phenotypes such as epithelial-mesenchymal transformation, invasion, and migration. The conclusions of this paper are mostly well supported by data, but some analyses need to be clarified.

(1) Because diverse biomarkers have been identified for EMT, it is recommended to declare the advantages of using RGS10 as an EMT marker.

Answer: Thank you for your comments. The dysregulation of RGS protein expression has been observed to be associated with various types of cancer. (PMID: 26293348). Previous studies have shown that RGS10 knocking down can lead to chemotherapy resistance of ovarian cancer cells to paclitaxel, cisplatin, and vincristine. In colorectal tumors, the transcription of RGS10 is regulated by DNA methylation and histone deacetylation. As a key regulatory factor in the G protein signaling pathway, RGS 10 is involved in tumor development including survival, polarization, adhesion, chemotaxis, and differentiation, these hints suggest RGS10 might be a marker for EMT in breast cancer.

(2) The authors utilized databases to study the upstream regulatory mechanisms of RSG10. It is recommended to clarify why the authors focused on miRNAs rather than other epigenetic modifications.

Answer: Thank you for your comments. miRNAs are short-chain non-coding RNA molecules that bind to the target mRNA's 3 'untranslated region (3'UTR) to cause mRNA degradation or translation inhibition, thus regulating gene expression in cells. These small molecules play a crucial role in regulating the expression of cancer-related genes and can act as tumor promoters or tumor suppressors. To further improve the molecular mechanism of malignant biological behavior of breast cancer cells with RGS10, we verified that miR-539-5p might be the upstream regulation target of RGS10 through bioinformatics prediction and in-vitro experiments.

(3) The role of miR-539-5p in breast cancer has been described in previous studies. Hence, it is recommended to provide detailed elaboration on how miR-539-5p regulates the expression of RSG10.

Answer: Thank you for your comments. To verify the effect of miRNA-539-5p regulating the expression of RSG10, we transfected miR-539-5p mimic, miR-539-5p mimic NC, miR-539-5p inhibitor, miR-539-5p inhibitor NC in SKBR3 cells and MDA-MB-231 cells respectively, and verified the expression of RGS10 through RT-qPCR and Western blot experiments. The results showed that compared with the transfected miR-539-5p mimic NC or wild-type SKBR3 cells, RGS10 m RNA and protein levels were significantly reduced. On the contrary, after MDA-MB-231 cells were transfected with miR-539-5p inhibitor to inhibit the expression of miR-539-5p, RGS10 mRNA and protein levels in MDA-MB-231 cells were significantly increased (Fig. 3.4A-C, Fig. 3.5A-C). This indicates that miR-539-5p can target and regulate RGS10.

(4) To enhance the clarity and interpretability of the Western blot results, it would be advisable to mark the specific kilodalton (kDa) values of the proteins.

Answer: Thank you for your comments and suggestions. We have corrected to mark the specific kilodalton (kDa) values of the proteins in WB.

**Recommendations for the authors:**

**Reviewer #1 (Recommendations For The Authors):**

The function of RGS10 in breast cancer was identified in the paper. However, some major issues in this paper need to be specified:

(1) From reading the introduction section and its references, RGS proteins participate in multiple essential cellular processes and may be tumor initiators or suppressors (Li et al., 2023). This article focuses on the significance of RGS10 in breast cancer, it is recommended to show how the function of RGS10 exhibits therapeutic significance in other types of cancer.

Answer: Thanks for your comments and suggestions on our findings. The dysregulation of RGS protein expression has been observed to be associated with various types of cancer. Especially in ovarian cancer cells. (PMID: 26293348). It has been found that the RGS10 expression is lower than that of normal ovarian cells. (PMID: 21044322). In addition, it has been found that knocking down RGS10 can enhance the vitality of ovarian cancer cells and promote chemoresistance by activating the Rheb GTP/mTOR signaling pathway. (PMID: 26319900). A study suggests that RGS10 mediates inflammation signaling regulation in SKOV-3 ovarian cancer cells with high expression of TNF and COX-2 after RGS10 knockdown. In colorectal tumors, RGS10 transcription is regulated by DNA methylation and histone deacetylation. (PMID: 35810565). RGS10 expression also are associated with poor prognosis in laryngeal cancer, hepatocellular carcinoma, and pediatric acute myeloid leukemia. (PMID: 32776811, PMID: 26516143, PMID: 30538250)

(2) The authors characterize RGS10 protein expression in the breast cancer cell lines MDA-MB-231, MCF7, and SKBR3 in vitro Figure 2A. However, more information would strengthen the data - e.g. information on the expression of RGS10 protein and the survival in public databases, as well as the correlation between RGS10 and Her-2 expression.

Answer: Thanks for your comments. we have checked the correlation of RGS10 expression and survival rate of Her-2 positive breast cancer patients in a public database. Although there is no significant difference in the “p” value, however, RGS10 high-expression patients have a favorable prognosis tendency than RGS10 low-expression patients after the 100th month.

**Author response image 1.**

(3) Regarding the current situation of clinical trials in the RGS family, the potential to develop RGS 10 for clinic translation is a driving factor for EMT.

Answer: Thank you for your comments. The RGS (G protein signal transduction regulator) gene family provides an important "braking" function for the cell receptor family of G-protein coupled receptors (GPCR). GPCR controls hundreds of important functions in systemic cells and is the largest class of drug targets, with over one-third of FDA approved drugs treating diseases by binding to GPCR and altering its activity. When GPCRs are activated by hormones or neurotransmitters, they initiate signaling cascades within host cells through signal-carrying proteins called G proteins. The function of the RGS protein is to inactivate the G protein, thereby shutting down this signaling cascade reaction, which limits G protein signal transduction and allows cells to reset and receive new incoming signals. If it were not for it, the signals triggered by GPCR would inappropriately remain on, and the signal transduction would experience dysfunction (PMID: 33007266). The potential to develop RGS10 as a driving factor of EMT is meaningful for clinic translation.

(4) In Figure 3A, the paper showed that differential gene expression revealed 70 genes were significantly upregulated in RGS10-depleted SKBR3 cells, The authors didn't show any data on the expression of other EMT-related proteins in pathway analysis.

Answer: Thank you for your comments. The enrichment analysis of RNA sequencing in RGS10-depleted SKBR3 cells suggests that high correlation factors that are associated with EMT, such as TAGLN, TNFSF10, NDUFA4L2, CCN5, PHGDH, ST3GAL5, ANG, and LCN2.

(5) In Figure 3B, the paper focuses on LCN2 in pathway analysis, however, the author did not elaborate on the significance of LCN2-related pathways in EMT.

Answer: Thank you for your comments. Some studies have the significance of LCN2-related pathways in EMT. It was confirmed that LCN2 upregulation triggered by PTEN insufficiency induces EMT to promote migration and invasion in MCF7 cells (PMID: 27466505). The activation of STAT3 contributes to an increase in LCN2 expression, which activates ERK pathway-dependent EMT, thus promoting lung metastasis in MDA-MB-231 cells in breast cancer (PMID: 33473115). The silencing of LCN2 reduced the ability of migration and invasion of SUM149 cells and the proportion of tumor stem cells, suggesting that LCN2 may mediate the invasion and metastasis of cancer cells by regulating the stemness of breast cancer cells. The biological effects of LCN2 small molecule inhibitors ZINC00640089 and ZINC00784494 targeting IBC cells have been confirmed. The siRNA-mediated silencing of LCN2 in IBC cells significantly reduces cell proliferation, viability, migration, and invasion. (PMID: 34445288).

(6) Minor: the author did not conduct a semi-quantitative analysis of the immunohistochemical results of RGS10.

Answer: Thank you for your suggestion. We would like to demonstrate the qualitative analysis of RGS10 immunohistochemistry. The semi-quantitative analysis is not required in the paper.

**Reviewer #2 (Recommendations For The Authors):**

The role of RGS10 was well-characterized in this study, However, some minor points need to be modified.

(1) Page 15 line 296, description of cell proliferation was missing, please modify.

Answer: Thank you for your comments. We have corrected the description of cell proliferation on Page 15 highlighted in red.

(2) In Figure 2C, the title of the Y-axis was missing.

Answer: Thank you for your comments. We have corrected the description of the Y-axis title in Figure 2C.

(3) Describe the transfection reagent that was used in this study, and incorporated into the methods section.

Answer: Thank you for your comments. We have added the description of the transfection reagent to the methods section.

(4) The manuscript needs proofreading.

Answer: Thank you for your comments. We have proofread the manuscript.
